# On the accuracy and precision of X-ray and neutron diffraction results as a function of resolution and the electron density model

**DOI:** 10.1107/S2052252520010441

**Published:** 2020-08-25

**Authors:** W. Fabiola Sanjuan-Szklarz, Magdalena Woińska, Sławomir Domagała, Paulina M. Dominiak, Simon Grabowsky, Dylan Jayatilaka, Matthias Gutmann, Krzysztof Woźniak

**Affiliations:** a Biological and Chemical Research Centre, Department of Chemistry, University of Warsaw, Żwirki i Wigury, Warszawa, Poland; b Department of Chemistry and Biochemistry, University of Bern, Freiestrasse 3, 3012 Bern, Switzerland; c School of Molecular Sciences, University of Western Australia, 35 Stirling Highway, Perth WA 6009, Australia; d Rutherford Appleton Laboratory, ISIS Facility, Chilton, Didcot, Oxfordshire OX11 OQX, United Kingdom

**Keywords:** Hirshfeld atom refinement, multipole model, transferable aspherical atom model, aspherical models

## Abstract

X-ray diffraction is the main source of three-dimensional structural information. In total, more than 1.5 million crystal structures have been refined and deposited in structural databanks (PDB, CSD and ICSD) to date. Almost 99.7% of them were obtained by approximating atoms as spheres within the independent atom model (IAM) introduced over a century ago. In this study, X-ray datasets for single crystals of hydrated α-oxalic acid were refined using several alternative electron density models that abandon the crude spherical approximation: the multipole model (MM), the transferable aspherical atom model (TAAM) and the Hirshfeld atom refinement (HAR) model as a function of the resolution of X-ray data. The aspherical models (MM, TAAM, HAR) give far more accurate and precise single-crystal X-ray results than IAM, sometimes identical to results obtained from neutron diffraction and at low resolution. Hence, aspherical approaches open new routes for improving existing structural information collected over the last century.

## Abstract 2

The aspherical models (MM, TAAM, HAR) give far more accurate and precise single-crystal X-ray results than IAM, sometimes identical to results from neutron diffraction and at low resolution. Hence, aspherical approaches open new routes for improving all of the existing structural information collected over the last century.

## 1. Introduction   

Paul Ewald (Ewald, 1948 ▸) first suggested to Max von Laue that X-rays with wavelengths comparable to the interatomic distances in crystals might produce diffraction patterns from them. Max von Laue then encouraged two PhD students (Paul Knipping and Walter Friedrich) to verify this hypothesis. Shortly thereafter, they produced the very first X-ray photograph demonstrating the diffraction phenomenon. Simultaneously, William Henry Bragg built the first X-ray spectrometer, while his son William Lawrence Bragg explained the relationship between the observed X-ray spots and the structure of crystals independently of Max von Laue. This earned Max von Laue the 1914 Nobel Prize in Physics for his discovery of X-ray diffraction by crystals, and the 1915 Nobel Prize in Physics for the Braggs for the analysis of crystal structures using X-rays. Around the time when Max von Laue received the Nobel Prize, William Henry Bragg and Arthur Holly Compton (Compton, 1915 ▸) put forward the hypothesis that each atom/ion could be modelled with a spherical electron density, put into practice in 1925, when the first spherical atomic scattering factors were calculated by Hartree (1925 ▸), which are today still used in more or less the same form.

This model of spherically averaged electron density distributions obtained from theoretical methods developed for isolated atoms in the ground state is called the independent atom model (IAM). IAM does not take into account the changes in the electron density distribution of individual atoms due to chemical bonding, charge transfer, lone electron pairs, *etc*. Nevertheless, IAM is the most commonly used electron density model. Using this model, in total almost 1.5 million structures of inorganic and organic small molecules and macromolecules have been solved and refined so far, including such famous milestones as, for example, the first atomic level structure of transfer RNA in 1973 by Alexander Rich (MIT) (Kim *et al.*, 1973 ▸), the structure of ten base pairs of right-handed DNA by Richard Dickerson in 1980 (Drew *et al.*, 1981 ▸), the first structure of a protein/DNA complex refined by John Rosenberg in 1984 (University of Pittsburgh) (Frederick *et al.*, 1984 ▸), and the structure of the ribosome (Wimberly *et al.*, 2000 ▸) solved by Harry Noller (University California), Venki Ramakrishnan (Cambridge), Thomas Steitz (Yale University) and Ada Yonath (Weizmann Institute of Science), for which these last three scientists were awarded the 2009 Nobel Prize in Chemistry.

Nowadays, single-crystal X-ray and neutron diffraction techniques are the most common experimental methods for obtaining the 3D structure of molecules in the crystalline state. Although the most modern neutron facilities can provide reasonable results even for sub-millimetre-sized single crystals, only *ca* 0.3% of all 1.5 million currently available crystal structures have been determined by neutron diffraction. For the purpose of this work, we collected 14 different neutron diffraction datasets for α-oxalic acid, our test crystal. We will use these neutron diffraction results as ‘gold standard’ reference values.

Structural data from X-ray diffraction are extremely useful in chemistry, pharmacy, crystal engineering, materials science, *etc*. and are stored in crystal structure databases such as the Cambridge Structural Database (Allen, 2002 ▸) or the Inorganic Crystal Structure Database (Bergerhoff & Brown, 1987 ▸; Belsky *et al.*, 2002 ▸), whereas macromolecular/protein single-crystal X-ray structural data are compiled in the Protein Data Bank (Berman *et al.*, 2000 ▸). High-quality structural data are crucial for the further progress of many areas of science, as these data are used to estimate the energies of inter- and intramolecular interactions, for modelling mechanisms of biochemical processes, prediction of new materials with pre-defined properties, and the design of new drugs and materials, *etc*. In single-crystal X-ray diffraction, the quality of the final result depends on several factors. One of the most important factors is the maximum diffraction angle, θ_max_ (or 2θ_max_), *i.e.* the limit at which measured reflections are still taken into consideration in structure refinement. According to commonly accepted guidelines (Spek, 2003 ▸; 2020 ▸), the maximum diffraction angle of the measured reflections (θ_max_) for a single-crystal X-ray diffraction experiment intended for publication in crystallographic journals and crystallographic databases (CSD, ICSD) should be such that sin θ_max_ /λ > 0.6 Å^−1^ (*i.e.* θ_max_ > 25° for Mo *K*α and θ_max_ > 67° for Cu *K*α X-ray radiation).

Another crucial factor is the quality and sophistication of the electron density model used in the refinement procedure. As discussed above, the simplest and most frequently applied model in structural crystallography is IAM. Today, even when one uses the most modern technology such as synchrotrons, free-electron lasers or modern in-house X-ray diffractometers, structural refinements are based on 100 year-old methodology. This was justifiable in the past because errors associated with hardware were much greater than errors associated with electron density models used in the refinements. However, this justification no longer holds and has not done so for many years. Modern hardware and software are very accurate and precise. Errors associated with hardware are by far smaller than those arising from the models of electron density. Almost all crystallographers use estimated standard deviations (e.s.d.s) from the refinement procedures as measures of precision; however, this severely underestimates the real errors present as e.s.d.s only estimate errors in the starting measured quantities transmitted into the errors of the final results. This approach does not take into account all sources of errors, especially systematic errors; therefore, it is far better to estimate errors using sample standard deviations (s.s.d.s) obtained for multiple measurements as will be demonstrated in this work. Hence, our primary aim is to estimate the precision and accuracy of the final structural results obtained by applying more advanced and modern electron density models as compared with IAM. Accuracy and precision are defined in the Statistical analysis[Sec sec4.9] of this work which should be read before the results and discussion.

In more advanced models, the well known asphericity of atomic electron density is explicitly included, while in IAM it is not. These advanced models were first introduced by McWeeny (1952 ▸; 1953 ▸), Dawson (1967 ▸), Kurki-Suonio (1968 ▸), Hirshfeld (1971 ▸; 1977 ▸) and later developed by Stewart (1976 ▸) and Hansen & Coppens (1978 ▸). In the Stewart and Hansen–Coppens models, the total atomic electron density is the sum over the so-called pseudoatoms. Pseudoatoms are the smallest transferable atomic fragments of electron density from which the total electron density distribution can be reconstructed. The electron density of each pseudoatom is centred around an atomic nucleus. This electron density is calculated from the sum of the spherical core electron density, the spherical valence electron density and the valence deformation density. Fourier transform of the pseudoatom electron density produces an aspherical atomic scattering factor which allows for easy modelling of the aspherical concentration and depletion of the electron density in crystals. It has been shown that a molecular geometry very close to the neutron geometry can be obtained after multipole refinement of high-resolution X-ray diffraction data (Hoser *et al.*, 2009 ▸). We will refer to this electron density model as the multipole model (MM). Unfortunately, multipole refinement of experimental electron density can only be achieved for the highest resolution data (in general, up to sin θ_max_ /λ > 1.0 Å^−1^). This is a serious limitation as most crystals do not diffract X-rays to such high resolutions.

Because electronic parameters for the same type of atoms in identical topological environments appear to be grouped close to their average values, databanks of pseudoatom parameters were developed (Brock *et al.*, 1991 ▸). There are three major pseudoatom databanks: UBDB (Koritsanszky *et al.*, 2002 ▸; Volkov *et al.*, 2004 ▸; Dominiak *et al.*, 2007 ▸; Jarzembska & Dominiak, 2012 ▸; Kumar *et al.*, 2019 ▸), Invariom (Dittrich *et al.*, 2004 ▸, 2006 ▸, 2013 ▸; Hübschle *et al.*, 2007 ▸) and ELMAM (Pichon-Pesme *et al.*, 1995 ▸; Domagała & Jelsch, 2008 ▸; Domagała *et al.*, 2012 ▸). ELMAM is based on purely experimental charge densities resulting from multipole refinement against high-resolution X-ray diffraction data, whereas the other two databases are based on theoretical calculations. Using both the transferable aspherical atomic model (TAAM) refinement methodology and high-resolution X-ray data significantly improves the molecular geometries obtained (Kumar *et al.*, 2019 ▸; Dittrich *et al.*, 2004 ▸, 2006 ▸; Volkov *et al.*, 2007 ▸; Jelsch *et al.*, 2005 ▸; Bąk *et al.*, 2011 ▸) with respect to IAM and also leads to atomic displacement parameters (ADPs) closer to those obtained from multipole refinements (Dittrich *et al.*, 2006 ▸; Volkov *et al.*, 2007 ▸; Bąk *et al.*, 2011 ▸, 2009 ▸; Jayatilaka & Dittrich, 2008 ▸; Sanjuan-Szklarz *et al.*, 2016 ▸). In addition, TAAM refinement appears to give molecular geometries that are in excellent agreement with the optimized geometries obtained from periodic DFT (Dovesi *et al.*, 2005 ▸) calculations (Bak *et al.*, 2011 ▸).

For the last few years, a new approach to the refinement of single-crystal X-ray data has become more and more important: Hirshfeld atom refinement (HAR) (Jayatilaka & Dittrich, 2008 ▸; Capelli *et al.*, 2014 ▸), yet another excellent example of so-called quantum crystallography (Grabowsky *et al.*, 2017 ▸), combining electronic wavefunctions from first-principles theoretical calculations and experiments. In HAR, the geometry and ADPs are refined with aspherical atomic scattering factors calculated as Fourier transforms of atomic electron densities (the Hirshfeld atoms) derived from Hirshfeld’s stockholder partitioning of quantum mechanical molecular electron densities (Hirshfeld, 1977 ▸). These molecular electron densities are iteratively updated between each of the refinement steps to produce the best possible electron density model for the particular compound under scrutiny (Capelli *et al.*, 2014 ▸). They are calculated at the Hartree–Fock or DFT level, and the crystal environment is simulated by surrounding with a cluster of atomic Hirshfeld charges and dipoles. HAR has been implemented in the software *Tonto* and *HARt* interfaced to *OLEX2* (Jayatilaka & Grimwood, 2003 ▸; Fugel *et al.*, 2018 ▸). TAAM refinement has also been avalaible for many years now through multipolar model related software programs (Volkov *et al.*, 2004 ▸, 2006 ▸; Hübschle *et al.*, 2007 ▸; Jelsch *et al.*, 2005 ▸; Petricek *et al.*, 2014 ▸). It has recently been implemented in the software library *DiSCaMB* (Chodkiewicz *et al.*, 2018 ▸) and interfaced to *OLEX2* (Jha *et al.*, 2020 ▸).

**Figure 1:**
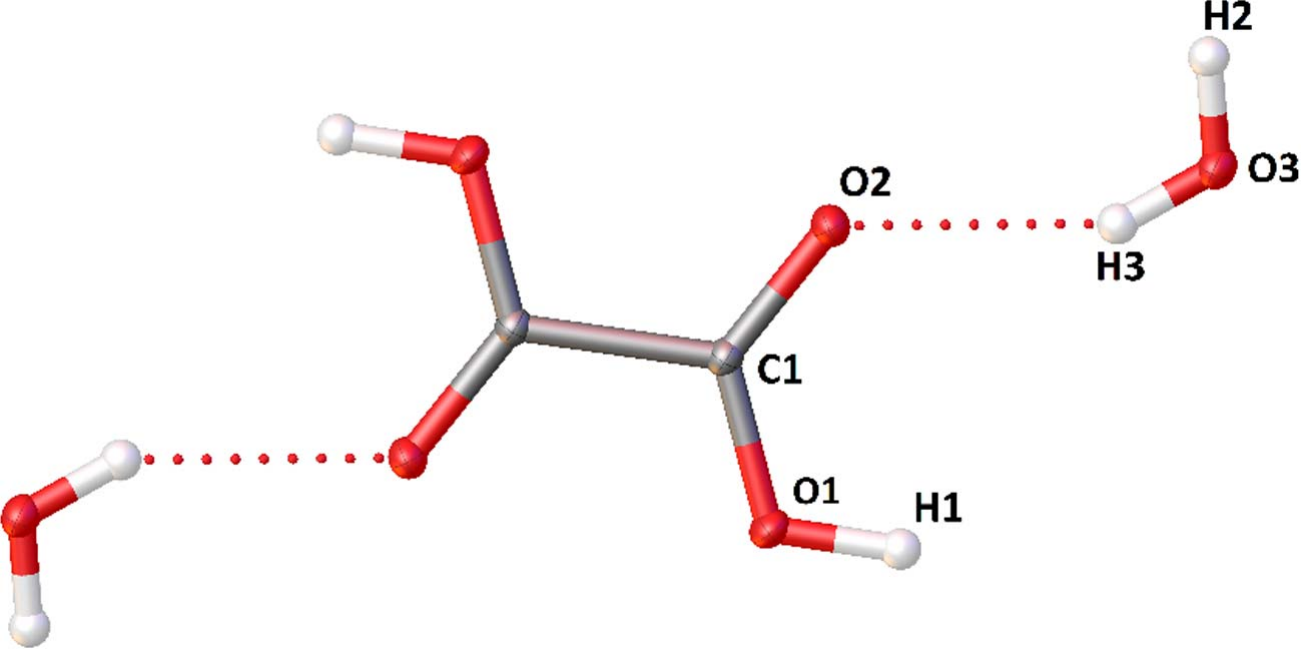
Labelling of atoms in oxalic acid dihydrate. The molecule of oxalic acid is located at a special position (inversion centre located at the midpoint of the C1—C1 bond) and is accompanied by two molecules of H_2_O. The asymmetric part of the unit cell consists of those atoms which are labelled.

The MM, TAAM and HAR electron density models were tested against multiple X-ray diffraction datasets from our test crystals of oxalic acid (C_2_H_2_O_4_·2H_2_O) (Kaminski *et al.*, 2014 ▸) (Fig. 1 ▸) in order to demonstrate the dependence of the final results on the diffraction angle and electron density model. Single crystals of oxalic acid are a well known standard used to fine-tune X-ray diffractometers. The choice of oxalic acid has many advantages including facile crystal growth and high suitability for charge density measurements. Oxalic acid crystals were studied using X-ray diffraction by Stevens *et al.* (1979 ▸) and later by Coppens and many others groups (Stevens & Coppens, 1980 ▸; Dam *et al.*, 1983 ▸; Coppens *et al.*, 1984 ▸; Zobel *et al.*, 1992 ▸). An interesting electron density study was reported by Martin & Pinkerton (1998 ▸) which was the first application of CCD detectors for experimental electron density studies.

### 1.1. Aims of the work   

The aim of this work is to compare the accuracy and precision of single-crystal X-ray and neutron diffraction studies using multiple datasets collected for different single crystals of hydrated α-oxalic acid as a function of the X-ray data resolution. We collected 13 high-resolution X-ray diffraction datasets (further cut to resolution shells of sinθ_max_/λ = 0.63, 0.71, 0.83, 1.00 and 1.14 Å^−1^) which were also previously used to analyse the reliability of multipole refinement results (Kaminski *et al.*, 2014 ▸). The maximal resolution is different for each individual *hkl* dataset within the range from 1.0 up to 1.2 Å^−1^, being on average 1.14 Å^−1^. The X-ray datasets were obtained for 13 different pieces of single crystals of hydrated α-oxalic acid at 100 K, whereas 14 neutron datasets were collected for just one piece of single crystal also at 100 K. All other *hkl* datasets for resolutions different from the maximal were obtained by trimming and reintegrating the original raw *hkl* data from the high-resolution data collection. A comparison of the structural results obtained from the MM/TAAM/HAR models was carried out with respect to the gold standard neutron diffraction (14 datasets, see above) and to IAM – the latter because poor model refinements can spoil even high-quality data. We were also able to compare the X-ray and neutron diffraction data collected with the results of periodic DFT calculations (Dovesi *et al.*, 2005 ▸). Our results will highlight whether the incredible progress in the development of hardware and software within the last few decades has been accompanied by a significant improvement of the quality (accuracy and precision) of the final structural results using modern electron density models.

## 2. Results   

We now discuss some representative examples of the relationship between the geometric and thermal parameters as a function of electron density model and data resolution for hydrated α-oxalic acid. Other examples of relationships and numerical values for all parameters can be found in the supporting information (Figs. S1–S7, Table S1). In each figure, small icons (circles, triangles and squares) denote the average values of a given property for each of the different models of electron density (colour codes are provided below the plots). The average value of the respective property ±3 s.s.d.s based on results from the 13 different single crystals are shown as ranges on the graph. The periodic DFT reference value is drawn as a horizontal pink line and the neutron data as grey lines. Horizontal grey range lines represent the associated ±3 s.s.d.s. Although for a given resolution the average values and vertical confidence intervals are shifted relative to one another, they are always grouped close to a given resolution. Each group of results was calculated for an exact resolution, but the icons are slightly shifted apart for the purpose of visual comparison. The TAAM models are ELMAM, Invariom and UBDB databank applications. HARs were performed with two different DFT methods (BLYP and B3LYP) and the basis set cc-pVTZ. MM was only conducted at each maximal resolution with and without constraints for hydrogen atom positions. For more details, see the Experimental[Sec sec4].

### 2.1. Discrepancy factors   

**Figure 2:**
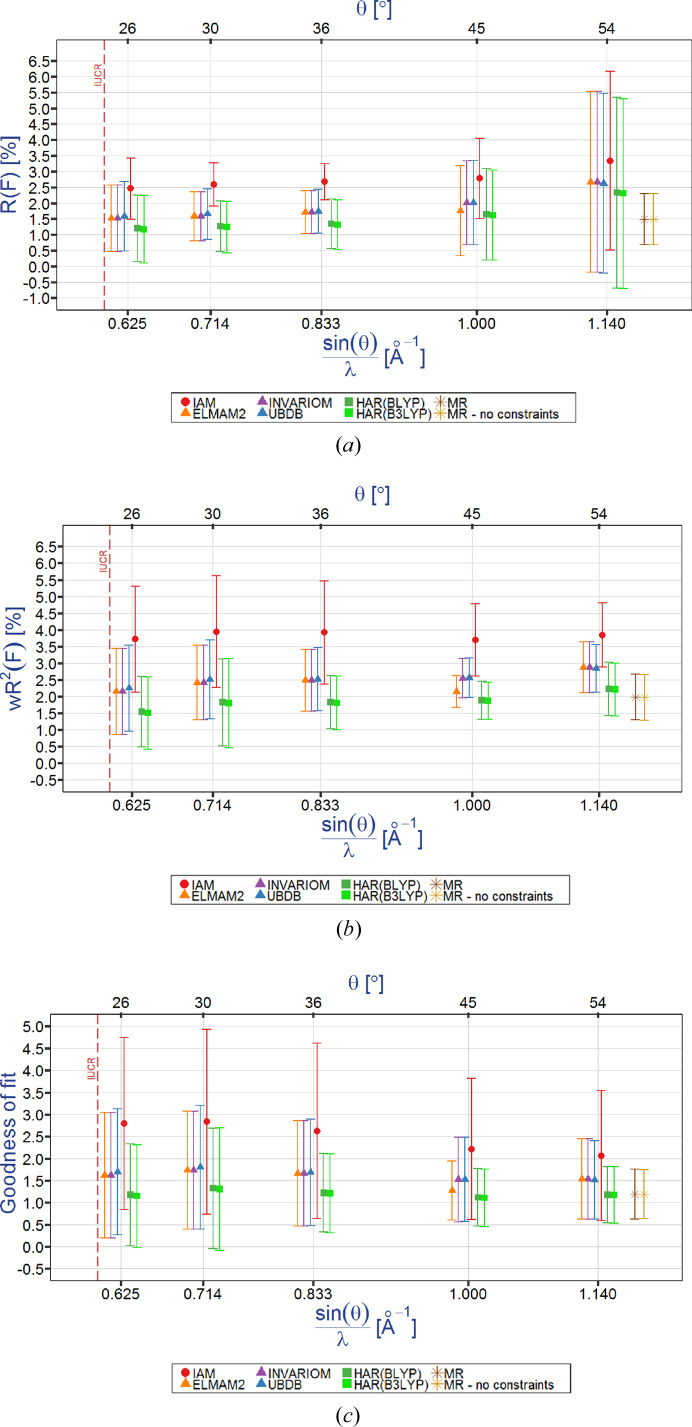
Discrepancy factors and GoF versus resolution and electron density model: (*a*) dependence of *R*(*F*), (*b*) *wR* ^2^(*F*) and (*c*) GoF. MR stands for multipole refinement, no constraints refers to hydrogen atom positions.

In Fig. 2 ▸, dependencies on resolution and electron density model are illustrated for two fitting discrepancy factors [*R*(*F*) and *wR* ^2^(*F*)] and the goodness-of-fit parameter (GoF). For *R*(*F*) [Fig. 2 ▸(*a*)], a significant elongation of precision intervals (decrease of precision) and a significant increase of average values of *R*(*F*) above sin(θ)/λ > 1 is evident for all electron density models with the exception of MM. Notably, for low-resolution data, the IAM discrepancy factor values are significantly larger than the corresponding values obtained for TAAM and HAR refinements. This is partly because of the smaller number of reflections present in the low-resolution data range. However, there seems to be a change of character of the dependence of *R* versus resolution somewhere between 1 and 0.8 Å^−1^. In fact, the precision of the *R*-factor for all models of electron density seems to worsen slightly towards the low-resolution data.

A weighting scheme is used in all the refinements. Since this is not accounted for in the values of *R*(*F*), the precision of *R*(*F*) worsens with resolution [Fig. 2 ▸(*a*)]. However, the reverse is observed for the precision of *wR* ^2^(*F*), where the e.s.d.s of reflections are taken into account *via* the weighting scheme (Fig. 2 ▸ *b*). In this case, precision improves towards higher resolution data. One can clearly see that the use of weights filters out significant parts of the errors introduced by high-order reflections which are usually less precisely determined. There are also systematic differences between values of *wR* ^2^(*F*) of IAM and aspherical refinements, the latter refinements exhibiting lower values, and they are preserved over the whole resolution range.

HAR refinements give GoF values closest to the ideal value of 1 [Fig. 2 ▸(*c*)]. TAAM refinements give GoF values close to 1.5. By far the largest and worst values of GoF are derived from IAM refinement. This is expected, since otherwise it would mean the data do not contain information beyond spherical scattering factors. Generally, GoF improves with increasing resolution, with IAM producing *ca* 2 times worse precision (larger confidence interval values) over the whole range of resolutions. GoF is quite sensitive and its precision clearly depends on resolution.

### 2.2. Bond lengths and valence angles   

**Figure 3:**
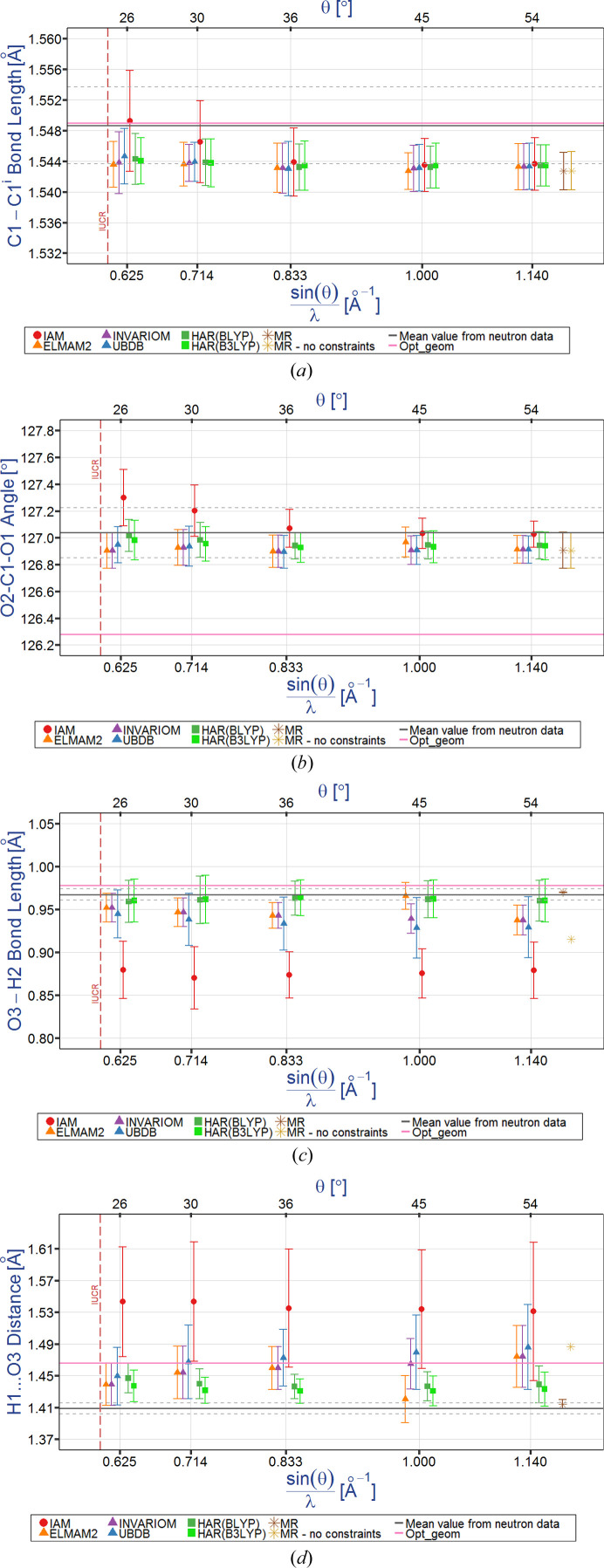
Typical dependencies of geometrical parameters on data resolution and electron density model refined against X-ray and neutron data for: (*a*) the C1—C1 bond length, (*b*) the O2—C1—O1 valence angle, (*c*) the O3—H2 and (*d*) H1—O3 bond lengths. Neutron data and results of periodic DFT calculations are given for the purpose of reference.

Fig. 3 ▸(*a*) shows a typical dependence for bond lengths between non-hydrogen atoms specifically for the central C1—C1 bond of oxalic acid. There is a small (*ca* 0.004 Å) systematic difference between the neutron C1—C1 bond length (1.549 Å) and the C1—C1 bond lengths obtained from the refinements against X-ray data (*ca* 1.544 Å for all X-ray refinements with the exception of low-resolution IAM refinement which, although less precise, coincidently tends to approach the neutron value; see the supporting information). For low-angle data (2θ_max_ = *ca* 50, 60 and 70°), the IAM C1—C1 bond length significantly increases with decreasing resolution while the precision of the IAM C1—C1 bond length simultaneously worsens. Interestingly, it has accuracy and precision comparable to all the other electron density models. Both HAR and TAAM produce the best values for the C1—C1 bond length which seem to be independent of resolution and the aspherical electron density model used. For the CO bonds, the general shape of the dependencies is similar [see Figs. S1(*a*) and S1(*b*) in the supporting information]. The only small difference is in the fact that, when one goes towards lower resolutions, the deviation of a given bond length from the other values obtained for the high-resolution data could either decrease towards the smaller values of the bond length or increase towards the larger values (as this is the case for the C1—C1 bond lengths). The optimized C1—C1 bond length obtained from periodic DFT computations coincides with the IAM 2θ_max_ = 50° C1—C1 bond length value and significantly differs from the other C1—C1 bond lengths. As for the low-resolution IAM bond lengths, the role of the valence electron density is strengthening, and this electron density is partly transferred towards the more electronegative atoms in the bonds, *i.e.* towards the O1 and O2 oxygen atoms for the C—O bonds, the IAM values of the C—C bond length tend to be slightly longer for low-resolution data than for higher resolution data. In all cases, HAR generates the most precise bond lengths between the non-hydrogen atoms. However, both HAR and TAAM exhibit similar accuracy. The supporting information also contains similar relations for the nonbonding O⋯O distances (Fig. S1).

A similar dependence also exists for the valence angles of the non-hydrogen atoms [see Figs. 3 ▸(*b*) and S2]. The IAM O2—C1—O1 valence angles deviate at low resolution (2θ_max_ = 50°) by *ca* 0.4°. However, HAR, TAAM and MM O2—C1—O1 valence angles are very stable and do not change with changing resolution. These values are also very close to the average values of the valence angles based on neutron data or computed using periodic DFT computations. The precision of the neutron time-of-flight Laue method results is slightly worse than that of almost all O1—C1—O2 valence angles resulting from the X-ray refinements and is comparable to the precision of the lowest resolution IAM results.

The above plots and those in the supporting information suggest a path for improvement of the IAM-refined single-crystal data collected in various structural databanks such as the CSD or ICSD. This can be achieved by re-optimization of the IAM-refined data against the measured intensities of scattered reflections using the aspherical electron density models, which means that the measured reflection intensities from the structure factors should also be collected by the above-mentioned databanks going forward. When intensities of reflections are collected, simple refinement of an aspherical electron density model (HAR or TAAM) can give more accurate and more precise structural data than what is currently present in the databanks. This is an important point as better quality structural information will speed up the development of those fields which use these structural data.

A very interesting dependence is obtained for the X—H bond lengths [see Figs. 3 ▸(*c*), S1(*c*) and S1(*d*)]. A systematic difference is immediately obvious between the IAM O2—H2 bond length and the average neutron value of this bond. To account for this discrepancy during routine refinements of crystal structures or electron densities in multipole refinements, the *X*—H bond lengths are normally artificially elongated to the average neutron values while keeping the valence angles *Y*—*X*—H constant, termed standardization/normalization. However, HAR refinements produce results close to those obtained from neutron diffraction for this bond length and the TAAM results are only slightly worse than those obtained for HAR. This means that the systematic difference between the neutron and IAM X-ray *X*—H bond lengths can be entirely attributed to the IAM electron density model. Theoretical values obtained from periodic DFT are also almost identical to the neutron values. Although the precision of the neutron *X*—H values is higher than the precision of the X-ray results, the precision of the HAR results is the best, TAAM is not much worse, but IAM is significantly worse, and multipole model refinement results are the worst. In the case of the O3—H2 bond length, its precision increases slightly with increasing resolution.

A very similar result is obtained for the H⋯*X* hydrogen bonds [Figs. 3 ▸(*d*), S1(*e*) and S1(*f*)] as for the *X*—H bond lengths. As H⋯*X* is a weaker interaction compared with the *X*—H bond, the precision of almost all the parameters is worse than in the case of the *X*—H bond lengths (again with the exception of HAR results). Also, in the case of HAR results, the accuracy of the H⋯*X* bond lengths appears to be independent of the data resolution. HAR results are also the most precise. They have even better precision than that obtained for *X*—H bond lengths. Still, the best precision for H⋯*X* bonds is obtained from neutron diffraction (H1⋯O3 hydrogen-bond lengths specifically). For more hydrogen-bond length dependencies, see Figs. S1(*e*)–S1(*i*).

### 2.3. Errors in geometrical parameters   

**Figure 4:**
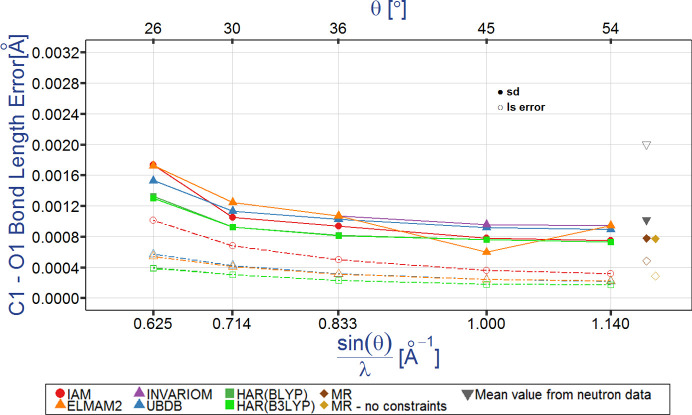
Typical dependencies of the errors of the geometrical parameters (in this case for the C1—O1 bond length) on data resolution and the electron density model refined against X-ray and neutron data; l.s. stands for the least-square e.s.d. values.

Fig. 4 ▸ [and Figs. S3 and S4] illustrates the typical dependencies for the C1—O1 bond length errors as a function of data resolution and electron density model. Two types of errors are considered. These are estimated standard deviation (e.s.d.s, the values obtained from the least-squares refinement against the X-ray or neutron diffraction data) and sample standard deviation (s.s.d.) values calculated on the basis of multiple datasets (13 data collections using X-ray diffraction and 14 for neutron diffraction). Several observations are apparent. First, the s.s.d.s are larger than the e.s.d.s for all the resolutions and for all the electron density models. Intuitively, this is acceptable as e.s.d.s only take into account the errors of the variables defining the refined model, whereas s.s.d.s take into account all the possible random and systematic errors including those which are not accounted for by e.s.d.s. The HAR-derived s.s.d. values for the C1—O1 bond lengths are the smallest among the s.s.d.s as are the corresponding e.s.d. values. All of the errors are dependent on the data resolution and the largest errors are obtained for the lowest resolution data. For IAM, the s.s.d. values are larger than the e.s.d. values and this difference increases with increasing data resolution (up to 3 times larger s.s.d. than e.s.d. for the highest-resolution data). Even routine structural investigations could benefit from error reduction when carefully measured higher resolution data are utilized. The bond length errors present in the neutron diffraction bond lengths are usually higher than the errors from X-ray data for bonds between non-hydrogen atoms and are smaller for the bonds involving hydrogen atoms.

### 2.4. Thermal parameters   

**Figure 5:**
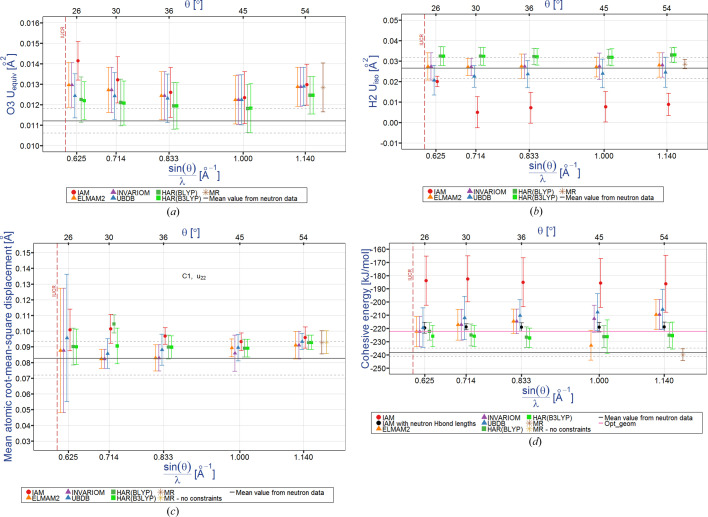
Typical dependencies of the thermal parameters and cohesive energy on the data resolution and the electron density model refined against X-ray and neutron data for: (*a*) *U*_eq_ for the O3 atom, (*b*) *U*_iso_ /*U*_eq_ for the H2 atom and (*c*) *U*_22_ for the C1 atom and (*d*) cohesive energies of crystals. In the case of hydrogen atoms, the plot contains *U*_iso_ values for IAM and UBDB, ELMAM2 and INVARIOM refinements and *U*_eq_ for the other methods. Opt_geom refers to the cohesive energy calculated for the optimized geometry.

Typical dependencies for the equivalent thermal factors (*U*) for heavy atoms are illustrated in Fig. 5 ▸(*a*). It appears that the *U*_equiv_ values are dependent on both the resolution and the electron density model. As far as resolution is concerned, Cruickshank (1956 ▸) stated that high-order diffraction data should contain more information from sharp electron density features and less information from bonding electron density which would be reflected in the quality of the ADPs. More illustrations for the other atoms are shown in Fig. S5. Again, the IAM *U*_equiv_ values increase for low resolution, thus differing from the HAR and TAAM values which remain more or less the same in the entire resolution range; they only increase slightly for the high-resolution data. The HAR and TAAM models seem to produce comparable accuracy and precision for the *U*_equiv_ values. The HAR O3 *U*_equiv_ value is the closest to the neutron value of *U*_equiv_ for O3. A slight increase for the highest resolution could be associated with the errors introduced by core electrons not adequately accounted for, particularly by the pseudoatom model. Apparently, in order to get reliable *U*_equiv_ values one should use at least 0.9 Å^−1^ resolution data with IAM refinement, which means that all routine structural investigations have overestimated temperature factors. This conclusion is also in line with similar results presented for other compounds in the work by Sanjuan-Szklarz *et al.*, 2016 ▸) and should have a huge impact on the crystallographic community.

In the case of the isotropic hydrogen atom temperature factors [Figs. 5 ▸(*b*) and S5(*e*)–S5(*g*)], there are clear and significant differences between the HAR, IAM and TAAM H2 *U*_iso_ values. In general, these are the HAR values which are the closest to the neutron isotropic hydrogen ADPs (with the exception of one ELMAM2 value). Here, the precision of the data is comparable for all of the electron density models with the notable exception of routine IAM data which could not be refined for 2θ_max_ = 50°.

In the case of the components of a thermal motion tensor [see Fig. 5 ▸(*c*)], the scale of changes is larger and the trends are similar to those found for the *U*_equiv_ and *U*_iso_ temperature factors; namely, data precision decreases as resolution decreases. There seems to be a small minimum for the thermal tensor component values somewhere close to a resolution equal to 0.9 Å^−1^, and the HAR values also appear to be the most accurate and precise.

### 2.5. Unit-cell parameters and residual electron density   

We have also analyzed changes of the unit-cell parameters and residual electron density as a function of resolution (see Figs. S6 and S7). It appears that the unit-cell parameters are independent of resolution and, on average, very close to the neutron values. However precision of the X-ray unit-cell parameters is worse than those obtained from neutron diffraction. One can see quite significant differences between the optimized theoretical values (DFT) of the unit-cell parameters and the experimental values. In addition, precision of the volume for low-resolution data is slightly larger than for the higher resolution data.

In the case of the maximal and minimal residual electron density (Fig. S7) there seems to be a small increase of maximal residual electron density with increasing resolution for all methods of refinement. This is accompanied by a small decrease of minimum residual electron density. IAM refinements produce residuals (maximal) which are *ca* 2–3 times higher than the other methods of refinement. In the case of minimum residual electron density these differences are smaller. Interestingly, application of databanks of pseudoatoms leads to residuals which are very close to those from HAR (particularly for low-resolution data). The differences between HAR residuals and other TAAM approaches seem to increase slightly with resolution. For the aspherical approaches, precision of residuals seems to be worst for the extreme values of resolution. No doubt the aspherical refinements are very sensitive to the quality of reflections as, at first, precision increases with increasing resolution as information is accumulated with the increasing number of reflections. However, from *ca* 0.9 Å^−1^, precision worsens when weaker high-resolution reflections are also accounted for.

### 2.6. Small changes in geometry – significant changes in energies   

Are all these small differences in geometrical and thermal parameters obtained using different electron density models and different resolutions really significant and important? One can answer this question by analyzing the cohesive energies (crystal lattice energies) [Fig. 5 ▸(*d*)]. The cohesive energy is the difference between the crystal lattice energy per molecule and the molecular energy of a molecule in the gas phase. The mean cohesive energy values calculated from neutron diffraction results can be treated as an excellent reference value [the black line in Fig. 5 ▸(*d*)]. The cohesive energy for the optimized structure is shown as a pink line. As illustrated in Fig. 5 ▸(*d*), the cohesive energies obtained from the geometries after multipole refinement are effectively the same as the reference values. This is a trivial result as in these refinements the average bond lengths to hydrogen atoms obtained from single-crystal neutron diffraction were used. However, as not all the geometric parameters from neutron diffraction are utilized in multipole refinement (*e.g.* valence angles corresponding to bond-bending terms are not taken from neutron diffraction), the confidence interval for the average cohesive energy calculated on the basis of the geometry from multipole refinement is larger than the confidence interval for cohesive energies calculated for the geometry taken entirely from neutron diffraction.

When other approaches are applied, it appears that the results of HAR refinements are the closest to the reference cohesive energy value. They differ from the reference value by *ca* 10–13 kJ kJ mol^−1^ over the whole range of resolutions. The HAR(B3LYP) results are closer to the reference cohesive energies than those derived from HAR(BLYP) geometries. However, particularly for higher resolutions (2θ_max_ = 90°), the precision of the cohesive energies based on HAR(B3LYP) is slightly worse than the precision of the results based on BLYP. In general, the precision of the HAR results is only *ca* 2–2.5 times worse than the precision of the cohesive energies calculated using only neutron geometries. Interestingly, for low-resolution data (2θ_max_ = 50°), all methods of refinement give comparable accuracy, deviating by *ca* 20 kJ mol^−1^ from the reference neutron cohesive energy, although the precision of the pseudoatom database refinement methods are worse (≥50%) than the precision of the corresponding HAR results.

There is a systematic difference between the precision of the results obtained for geometries derived from UBDB refinements (*ca* ±15 kJ mol^−1^) compared with the results obtained from other databanks (*ca* ±10 kJ mol^−1^). Both the precision and accuracy of the cohesive energies based on the pseudoatom database geometries are dependent on data resolution. Interestingly, they both improve when routine resolutions are used, the only exception being the ELMAM2 results at the 2θ_max_ = 90° resolution. We assume that this is due to the fact that the ELMAM2 parameters are obtained from multipole refinement of oxalic acid data collected for this particular resolution and thus has implicit information from neutron geometry. In general, the precision of all the cohesive energies is *ca* 3 times worse than the reference precision of the cohesive energies based on neutron geometries. Similarly, as in the case of other parameters, both the precision and the accuracy of the cohesive energy calculated using IAM are the worst: the precision is equal to ±20 kJ mol^−1^ whereas the accuracy is *ca* 50 kJ mol^−1^ – a large deviation.

When IAM is used with bonds to hydrogen atoms normalized/standardized to the average neutron bond lengths (Allen & Bruno, 2010 ▸), the accuracy of the cohesive energy is equal to *ca* 15 kJ mol^−1^, being practically independent of resolution. Such an approach also gives excellent precision of ±5 kJ mol^−1^. This discrepancy in the accuracy of such crystal lattice energies results from differences in valence angles defined by hydrogen atoms which are not accounted for.

## 3. Discussion   

A century after the work of Laue, Ewald and the Braggs, more advanced refinements of X-ray diffraction data, which provide significantly more accurate and precise structural and electronic information, are mature now and ready to supplant IAM refinement methods. We have shown this by comparing multiple single-crystal X-ray results with neutron data obtained for multiple measurements of single crystals of hydrated α-oxalic acid and to the periodic DFT optimization outcome.

### 3.1. Accuracy and precision of structural results   

The best accuracy and precision for the X-ray results were obtained when either the HAR or TAAM electron density models were applied. Analysis of the dependencies of structural and thermal parameters obtained by refinement of the different models of the electron density against multiple X-ray and neutron datasets collected for single crystals of oxalic acid showed that IAM gave, in general, significantly worse accuracy and precision than the aspherical models of electron density. When comparing the results against X-ray data resolutions of sinθ_max_/λ = 0.625, 0.714, 0.832, 1.00 and 1.14 Å^−1^, the superiority of the aspherical methods was particularly clear for the lowest resolution data.

### 3.2. HAR results independent of resolution   

For the majority of structural parameters, HAR gives the most accurate and most precise structural results largely independent of the resolution of the input *hkl* data. It is often the most similar to neutron diffraction results. This means that by using HAR, one can get more accurate and more precise results (even for low-resolution data) than for any other electron density model. This is particularly important for those branches of crystallography which are limited in data resolution (*e.g.* high-pressure studies).

### 3.3. Superiority of TAAM and MM over IAM   

The electron density models TAAM and MM also produce results that are clearly superior to those derived from IAM. A century after the introduction of IAM, it is clear that all of the more advanced, aspherical approaches extract the information from X-ray diffraction data measured more successfully using the presently available modern X-ray diffractometers and sources.

### 3.4. Significant improvement of bond lengths (and valence angles) to hydrogen atoms   

Aspherical electron density models improve the experimentally observed bond lengths for hydrogen atoms. Application of HAR gives *X*—H bond lengths that are almost identical to the bond lengths obtained from single-crystal neutron diffraction. The other aspherical electron density models also give far better agreement with neutron data than IAM. This systematic difference between the X-ray *X*—H bond lengths and the neutron values of these parameters arises from the IAM electron density model centring the electron density maximum away from the atomic nucleus, but at the maxima of electron density in the *X*—H bonds. Consequently, when aspherical electron density models are used for the refinement, this discrepancy either disappears (for HAR) or is significantly diminished (for TAAM). No artificial standardization of the *X*—H bond to the neutron bond lengths is needed. It is a myth that X-ray diffraction cannot locate hydrogen atoms accurately or precisely; for the last century almost all crystallographers have been using a model of electron density which is not suited for the refinement of hydrogen atoms.

### 3.5. Significant improvement of geometrical parameters for non-hydrogen atoms   

In the case of structural parameters such as bond lengths and valence angles between the non-hydrogen atoms, the average differences increase at lower resolutions when IAM is used compared with the other models of electron density. In the case of routine structural data collection, the differences are largest up to 2θ_max_ for Mo *K*α = 50°. By employing more advanced electron density models than IAM, one can get better quality structural results.

### 3.6. Dependence of ADPs on resolution   

Also, temperature factors (ADPs) are model- and resolution-dependent. For the non-hydrogen atoms, IAM *U*_equiv_ clearly increases towards low resolution than is found for the aspherical approaches and all models of electron density give a slight increase of the equivalent temperature factors towards the highest resolutions. Apparently, the minimum value of *U*_equiv_ is close to 0.9 Å^−1^. In the case of hydrogen atoms, there is a systematic difference between isotropic temperature factors from IAM and all other models of electron density. In order to get the most reliable temperature factors, one should apply aspherical models of electron density in the refinement against X-ray data of the resolution higher than *ca* 0.9 Å^−1^. The IAM ADP values of hydrogen atoms are systematically underestimated and, in fact, should be corrected to the average neutron diffraction values, similarly as the *X*—H bond lengths are.

### 3.7. Better energies of crystal lattices   

By refining data with more advanced electron density models than IAM, one can get better quality energies of interactions for atoms and molecules in crystals. We observed that the results based on HAR are the closest to the reference cohesive energy value obtained from neutron data. They differ by *ca* 10–13 kJ mol^−1^ over the whole range of resolution. In general, the precision of HAR results is only *ca* 2–2.5 times worse than the precision of the cohesive energies calculated using neutron geometries. Interestingly, for low-resolution data, all aspherical methods of refinement gave comparable accuracy, deviating by *ca* 20 kJ mol^−1^ from the reference neutron cohesive energy, although the precision of the TAAM refinement methods were worse (≥50%) than the precision of the corresponding HAR results. Similar to the case of structural and thermal parameters, both the accuracy and the precision of the cohesive energy calculated using IAM was the worst (50 kJ mol^−1^, whereas the precision error was 35 kJ mol^−1^). However, excellent accuracy (*ca* 15 kJ mol^−1^) and precision (±5 kJ mol^−1^) are obtained when the IAM approach is coupled with the extension of bond lengths to hydrogen atoms to the average neutron *X*—H bond lengths (standardization/normalization procedure). Apparently, this is a low-cost way of obtaining a significant improvement of the final results of refinement. It should be supplemented by correction of X-ray valence angles defined by hydrogen atoms to the average neutron values of such angles which should lead to a further increase of both accuracy and precision of the resulting lattice energies.

### 3.8. Improvement of all structural information acquired so far   

An important consequence of the above results is that there definitely will be a significant improvement in the quality (accuracy and precision) of already measured and refined single-crystal X-ray data present in the CSD and ICSD when the stored X-ray datasets are re-refined with HAR or TAAM. Unfortunately, structure factors are not always present in the databanks, though storage should be mandatory to facilitate future improvements of structural information. In any case, such improved structural information would be especially attractive to all fields of science that start from structural data to determine properties, including crystal engineering and crystal structure prediction, materials science, life science, medicine, pharmaceutical research, *etc*.

## 4. Experimental   

### 4.1. Crystallization, X-ray data collection and reduction   

The details of crystallization, X-ray data collection and data processing are described in the work by Kaminski *et al.* (2014 ▸). High-resolution single-crystal X-ray diffraction experiments (13 in total) were performed on three different experimental setups. In each case a new crystal was grown, mounted on the goniometer head and cooled to 100 K. Depending on the overall quality of the crystal and its orientation, all the datasets were collected using different strategies optimized for each case. Each dataset was then separately reintegrated for this study with the following resolution cut-offs for sinθ_max_/λ = 0.625, 0.714, 0.832, 1.00 and 1.14 Å^−1^, which gave a total of 65 new truncated *hkl* datasets. The maximal resolution of all datasets is on average 1.14 Å^−1^. However, it is different for each individual *hkl* dataset within the range from 1.0 Å^−1^ up to 1.2 Å^−1^. All other resolution values – different from the maximal one – are exactly the same for all *hkl* datasets. Integrations were performed with the respective diffractometer software: *APEX2* (Bruker, 2008 ▸) or *CRYSALIS* (Rigaku Oxford Diffraction, 2012 ▸). Data reduction, correction, and merging were carried out using *SORTAV* (Blessing, 1987 ▸, 1989 ▸, 1995 ▸). The unit-cell parameters were always obtained using data limited to a certain resolution.

### 4.2. IAM X-ray refinement   

The routine IAM X-ray refinements were performed on datasets reintegrated to the desired resolution. The structural determinations and initial refinements were performed using *SHELX* (Sheldrick, 2008 ▸) within *OLEX2* (Dolomanov *et al.*, 2009 ▸). Further refinements based on *F* were performed in the *MoPro* suite (Jelsch *et al.*, 2005 ▸). The refinement scheme was as follows: (i) scale factors (also refined in all other stages); (ii) atomic coordinates and anisotropic ADPs for the non-hydrogen atoms; (iii) atomic coordinates and isotropic ADPs for the hydrogen atoms; (iv) atomic coordinates and ADPs. No restraints or constraints were applied, except at the lowest resolution (0.625 Å^−1^), for which restraints for the ADPs of hydrogen atoms were found to be necessary (scaled to 1.5 of *U*_eq_ computed from anisotropic ADPs of the carrier atom).

### 4.3. Multipole refinement   

All results of the multipole refinements are taken from the work by Kaminski *et al.* (2014 ▸). Multipole refinement of single-crystal X-ray data was only performed for the respective maximal resolution datasets.

### 4.4. TAAM   

The total electron density of a given molecule or macromolecule in a crystal can be reconstructed on the basis of multipole parameters of electron density of pseudoatoms transferable between different molecules (Brock *et al.*, 1991 ▸). Such an electron density model is called a transferable aspherical atom model (TAAM) of electron density and the refinement against X-ray data of this model is called the TAAM refinement (Bak *et al.*, 2011 ▸). In the course of the TAAM refinements, pseudoatom parameters for each atomic species are transferred from a databank and are kept fixed so that only the atomic coordinates and ADPs are refined.

The databank approach allows an easy parameterization, but at the expense of transferability errors. Pseudoatom parameters in the databank are obtained on the basis of some model molecules, not tailor-made for a system under study, unlike in the HAR approach. In our work, we tested the three available and well established databanks of pseudoatoms: the Invariom database (Dittrich *et al.*, 2004 ▸; 2013 ▸), the Experimental Library of Multipolar Atom Models (ELMAM) (Pichon-Pesme *et al.*, 1995 ▸; Domagała *et al.*, 2012 ▸) and the University at Buffalo Pseudoatom Data Bank (UBDB) (Koritsanszky *et al.*, 2002 ▸; Volkov *et al.*, 2004 ▸; Dominiak *et al.*, 2007 ▸; Jarzembska & Dominiak, 2012 ▸; Kumar *et al.*, 2019 ▸). Invariom and UBDB databanks offer pseudoatom parameters bearing information about electron densities of isolated molecules. Only in the case of ELMAM are the pseudoatom parameters obtained in the course of averaging over atomic multipole parameters derived from refinement against experimental structure factors, and hence contain information about interactions in the crystal environment. However, unlike in HAR, the model of electron density used in ELMAM (the multipole model) does not explicitly include information about interactions with the surrounding molecules.

TAAM refinements of the 13 X-ray datasets of hydrated α-oxalic acid at different resolution ranges were carried out with the *MoPro* software (Jelsch *et al.*, 2005 ▸). In each case, the model was refined against structure factor magnitude *F* fulfilling the threshold of *F* ^2^ > 2σ(*F* ^2^). The following software was used to transfer the atomic multipole parameters from each of the databases: Invariom – *InvariomTool* (Hübschle *et al.*, 2007 ▸), *UBDB2011* (Jarzembska & Dominiak, 2012 ▸) – LSDB (Volkov *et al.*, 2004 ▸) and ELMAM2 (Domagała *et al.*, 2012 ▸) – *MoPro* (Jelsch *et al.*, 2005 ▸). Positions and isotropic displacement parameters of hydrogen atoms were refined without any constraints. The weighting scheme used was *w* = 1/σ^2^(*I*_o_). The refinement strategy was as follows: (1) scale factor; (2) scale factor, atomic coordinates and ADPs for the non-hydrogen atoms; (3) scale factor, atomic coordinates and ADPs for the hydrogen atoms (4) scale factor, atomic coordinates and ADPs for all atoms.

### 4.5. HAR   

Hirshfeld atom refinement (HAR) (Jayatilaka & Dittrich, 2008 ▸; Capelli *et al.*, 2014 ▸) uses aspherical atomic scattering factors derived from stockholder partitioning (Hirshfeld, 1977 ▸) of molecular electron densities obtained in an iterative procedure of *ab initio* calculations of the molecular wavefunction of the oxalic acid molecule surrounded by a cluster of the nearest water molecules at the selected level of theory and with the basis set of choice. In HAR, as in TAAM, only coordinates and ADPs are refined, not the electron density parameters, which are calculated. This, in turn, allows us to avoid correlations between the ADPs and electron density parameters, making refinement of ADPs for hydrogen atoms feasible (Capelli *et al.*, 2014 ▸; Woińska *et al.*, 2014 ▸; 2016 ▸). At the same time, it prevents experimental errors from lowering the quality of the reconstructed electron density while retaining good agreement with the experiment (Volkov *et al.*, 2007 ▸). Unlike the TAAM refinement of electron density, HAR does not rely on the assumption that electron density parameters are transferable and, therefore, it is free from transferability errors, which makes this method more flexible and suitable to model subtle effects. It also provides higher flexibility in modelling core density by the choice of the basis set, which in the multipole model is frozen and cannot be changed in the refinement procedure. Moreover, in HAR, the influence of the interactions with the crystal environment can be taken into account during the calculations of the molecular wavefunctions by means of surrounding the central molecule with a cluster of Hirshfeld partition-derived atomic point charges and dipoles placed at the atomic sites, or even with a user-defined explicit cluster of molecules. As a result of this quality, HAR is more suitable for crystal structures with strong intermolecular interactions present in the crystal lattice, such as the studied structure of oxalic acid, compared with TAAM, in which such effects are not explicitly included. The improved electron density model present in HAR is crucial for developing a correct description of the electron density of hydrogen atoms, especially since these electron densities are often biased towards the heavier bonding partner. This allows the precise and accurate identification of the locations of hydrogen positions based on X-ray data refinement (Volkov *et al.*, 2007 ▸), which is particularly vital in the case of strong hydrogen bonds that are difficult to model with positions derived from neutron scattering data (Jelsch *et al.*, 2005 ▸).

HAR against amplitudes of all reflections for the X-ray datasets collected for hydrated α-oxalic acid was performed with the *TONTO* program (Jayatilaka & Grimwood, 2003 ▸). During HAR, the wavefunction of a cluster consisting of the central molecule of oxalic acid interacting via hydrogen bonds with the six nearest water molecules was calculated. The wavefunction was obtained in the course of DFT calculations with the cc-pVTZ basis set (Dunning, 1989 ▸), which was shown to be sufficient to refine hydrogen positions and ADPs with HAR (Capelli *et al.*, 2014 ▸), using two functionals: the purely theoretical BLYP functional and the B3LYP functional (Hohenberg & Kohn, 1964 ▸; Becke, 1993 ▸; Lee *et al.*, 1988 ▸), including semiempirical coefficients providing better fit to experimental data. In order to include interactions with the crystal environment, the central cluster of seven molecules was embedded in a cluster of atomic charges and dipoles for all the surrounding molecules with at least one atom within 8 Å from the central molecular cluster. All atomic positions and ADPs, including hydrogen atoms, were refined without any constraints or restraints. Hydrogen atoms were refined with anisotropic ADP values.

### 4.6. Limitations of pseudoatom databanks and HAR   

Although TAAM is superior to IAM, it has its own limitations. Pseudoatom databanks are built for atoms of light elements common in organic molecules. The databanks are continuously expanded, however they still do not contain all atom types, but this can be solved in the near future. There is no databank currently containing metal atoms or other heavier elements and it is not certain if such a databank can be built in the future. More studies are needed to evaluate the level of transferability (Chimpri & Macchi, 2013 ▸) and quality of multipolar modelling for heavier elements. None of the databanks applied here are able to quantitatively describe effects of intermolecular interactions on electron density. This would require the inclusion of intermolecular atom–atom interactions in the atom typing algorithm (Bojarowski *et al.*, 2017 ▸) and would enlarge the costs of building a databank to cover all known interactions. Finally, the database approach has a built-in error of transferability, which is very low (Hathwar *et al.*, 2011 ▸) but will always be present.

HAR is a method that still needs further development to achieve functionality comparable to IAM. One of its downsides is the long computational time required by repeated quantum mechanical calculations, which makes it impossible for this method to be directly applied to large molecules without combining it with fragmentation or database techniques. Moreover, refinement of structures other than molecular crystals such as network structures and ionic crystals is not yet properly handled. The problem with refinement of disordered structures is also not fully solved. Finally, crystal structures containing heavy elements are challenging due to the choice of basis set, implementation of relativistic methods and achieving convergence during wavefunction calculations.

### 4.7. Neutron diffraction   

Neutron diffraction data were collected as a result of 14 experiments performed on the same crystal of hydrated α-oxalic acid (dimensions 0.6 × 0.15 × 0.15 cm) using the SXD instrument (Wilson, 1990 ▸; 1997 ▸; Keen & Wilson, 1996 ▸; Keen *et al.*, 2006 ▸) at the ISIS spallation neutron source. The Laue time-of-flight diffraction method was applied, with a wavelength range of 0.48–7.0 Å. For peak integration, a local orientation matrix was refined for each frame using approximately 30 reflections from each of the 11 detectors. The dimensions of the unit cell were calculated as a weighted average of all local unit cells. Reflection intensities were extracted and reduced to structure factors using standard SXD procedures, as implemented in the computer program *SXD2001* (Gutmann, 2005 ▸). Refinements of * F* ^2^ against all reflections were performed using *Jana2006* (Petricek *et al.*, 2014 ▸) with ADPs for all atoms, including the hydrogen atoms.

### 4.8. Periodic DFT calculations   

Geometry optimization was carried out at the DFT(B3LYP) level of theory with the cc-pVTZ basis set. During the optimization procedure, atomic coordinates and cell parameters were varied. Initial parameters were taken from the multipolar refinement of the ninth dataset from a previous publication (Kaminski *et al.*, 2014 ▸) which was the best from a statistical point of view. Geometry optimization and theoretical calculations of energies of the crystal structures were performed using the *CRYSTAL09* (Dovesi *et al.*, 2009 ▸) program package at the DFT/B3LYP level of theory (Hohenberg & Kohn, 1964 ▸; Becke, 1993 ▸; Lee *et al.*, 1988 ▸) with the cc-pVTZ basis set (Dunning, 1989 ▸). Grimme D2 dispersion correction was applied (Civalleri *et al.*, 2008 ▸; Grimme, 2006 ▸). Geometry optimization with periodic boundary conditions and optimized unit-cell parameters was carried out in order to form a benchmark that can be used for the experimentally derived geometrical parameters. The cohesive energy for the optimized structure and all the experimental X-ray and neutron structures was calculated as the difference between the crystal lattice energy per molecule and the molecular energy of a molecule in the gas phase, as described in the literature (Civalleri *et al.*, 2008 ▸). BSSE was estimated using the counterpoise method (Boys & Bernardi, 1970 ▸) with ghost atoms selected within a distance of 5 Å from the central molecule.

### 4.9. Statistical analysis   

#### 4.9.1. Accuracy and precision   

**Figure 6:**
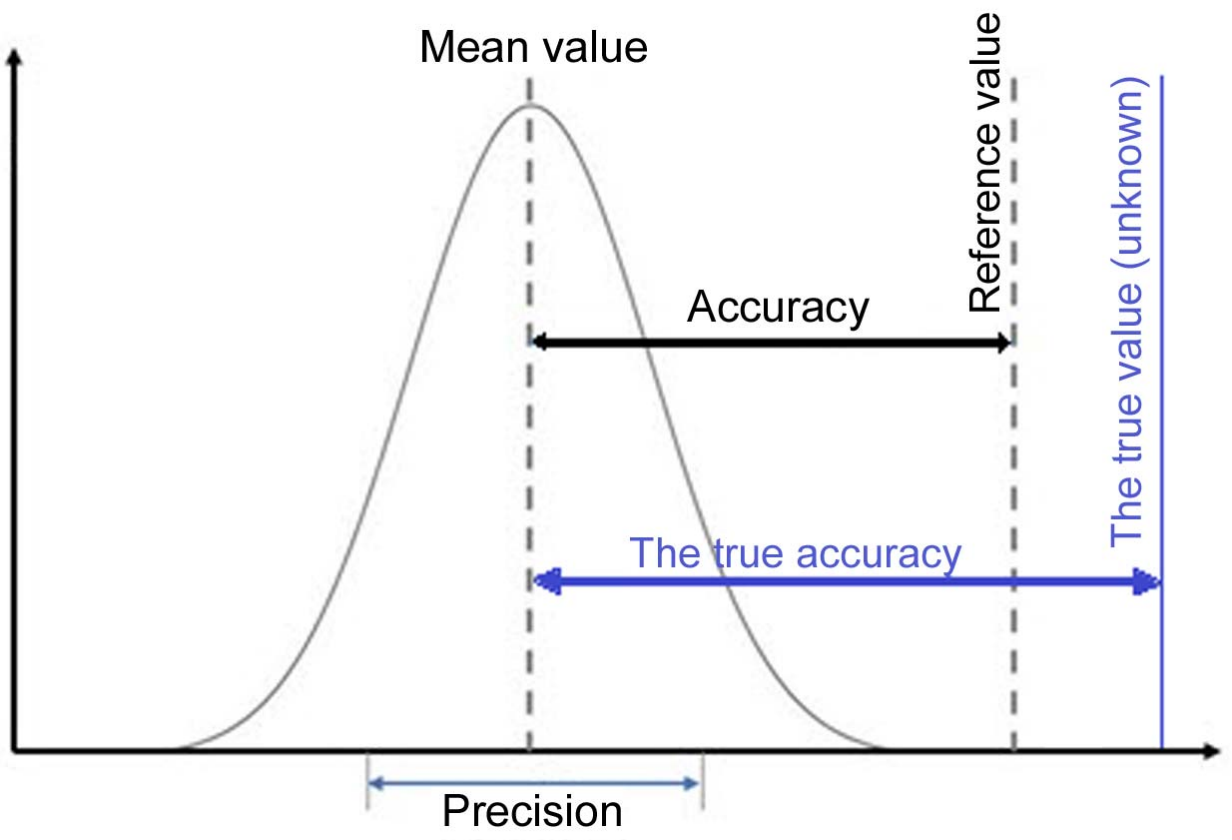
Definition of accuracy and precision.

Accuracy is always measured under the assumption that one knows the true value of a given parameter. In the case of multiple measurements, we estimate accuracy from the difference between the average value (see Fig. 6 ▸)
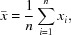
of a given parameter and the ‘true’ value. However, modelling molecular structures has several well known common errors. For example, it is well known that diffraction of neutrons takes place on nuclei and diffraction of X-rays on electron density and thus electron density in *X*—H bonds is shifted towards the non-hydrogen atoms. So it is obvious that when IAM refinement is used, the bond lengths to hydrogen atoms obtained from neutron diffraction are closer to the true values than those obtained from the X-ray *X*—H bond lengths. Therefore, the neutron-derived structural parameters are used as the reference values to estimate accuracy.

In structural studies, precision is commonly measured by the e.s.d., however, in the case of multiple data collection, it can also be estimated by the s.s.d. = *s*_*x*_):
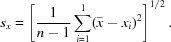
Estimated standard deviations take into account only those errors associated with variables that define a given model used in a given study. But there are also plenty of errors which are not accounted for by this such as the quality of the equipment, the effort expended on crystal production, in addition to various other random and systematic errors. This means that s.s.d.s should give a better estimation of errors than e.s.d.s.

## 5. Conclusions   

A century after the work of Laue, Ewald and the Braggs, more advanced models of aspherical atomic electron density used in refinements of X-ray diffraction data can provide significantly more accurate and precise structural and electronic information than the commonly used IAM. In particular, HAR and TAAM refinements supply the best accuracy and precision for X-ray results. The superiority of the aspherical methods was particularly clear for the lowest resolution data. In fact, HAR results are independent of resolution. This is very important for high-pressure studies and other branches of crystallography which supply data limited in resolution. The most common IAM refinement supplies the worst data and all aspherical approaches extract information from X-ray diffraction data more efficiently. Application of aspherical electron density models improves the experimentally observed bond lengths for hydrogen atoms and also significantly improves geometrical parameters for non-hydrogen atoms. Temperature factors (ADPs) are model and resolution dependent. The IAM ADP values of hydrogen atoms are systematically underestimated and should be corrected to the average neutron diffraction values, in a similar way as done for *X*—H bond lengths. By refining data with more advanced electron density models than IAM, one can get better quality energies of interactions for atoms and molecules in crystals. We observed that the results based on HAR are closest to the reference cohesive energy value obtained from neutron data. By re-refining the stored X-ray datasets with HAR or TAAM, one can improve the quality of all structural information acquired so far.

## Supplementary Material

Supporting information. DOI: 10.1107/S2052252520010441/lt5031sup1.pdf

Final cifs and checkcifs files. DOI: 10.1107/S2052252520010441/lt5031sup2.zip

CCDC references: 2007165, 2007166, 2007167, 2007168, 2007169, 2007170, 2007171, 2007172, 2007173, 2007174, 2007175, 2007176, 2007177, 2007178, 2007179, 2007180, 2007181, 2007182, 2007183, 2007184, 2007185, 2007186, 2007187, 2007188, 2007189, 2007190, 2007191, 2007192, 2007193, 2007194, 2007195, 2007196, 2007197, 2007198, 2007199, 2007200, 2007201, 2007202, 2007203, 2007204, 2007205, 2007206, 2007207, 2007208, 2007209, 2007210, 2007211, 2007212, 2007213, 2007214, 2007215, 2007216, 2007217, 2007218, 2007219, 2007220, 2007221, 2007222, 2007223, 2007224, 2007225, 2007226, 2007227, 2007228, 2007229, 2007230, 2007231, 2007232, 2007233, 2007234, 2007235, 2007236, 2007237, 2007238, 2007239, 2007240, 2007241, 2007242, 2007243, 2007244, 2007245, 2007246, 2007247, 2007248, 2007249, 2007250, 2007251, 2007252, 2007253, 2007254, 2007255, 2007256, 2007257, 2007258, 2007259, 2007260, 2007261, 2007262, 2007263, 2007264, 2007265, 2007266, 2007267, 2007268, 2007269, 2007270, 2007271, 2007272, 2007273, 2007274, 2007275, 2007276, 2007277, 2007278, 2007279, 2007280, 2007281, 2007282, 2007283, 2007284, 2007285, 2007286, 2007287, 2007288, 2007289, 2007290, 2007291, 2007292, 2007293, 2007294, 2007295, 2007296, 2007297, 2007298, 2007299, 2007300, 2007301, 2007302, 2007303, 2007304, 2007305, 2007306, 2007307, 2007308, 2007309, 2007310, 2007311, 2007312, 2007313, 2007314, 2007315, 2007316, 2007317, 2007318, 2007319, 2007320, 2007321, 2007322, 2007323, 2007324, 2007325, 2007326, 2007327, 2007328, 2007329, 2007330, 2007331, 2007332, 2007333, 2007334, 2007335, 2007336, 2007337, 2007338, 2007339, 2007340, 2007341, 2007342, 2007343, 2007344, 2007345, 2007346, 2007347, 2007348, 2007349, 2007350, 2007351, 2007352, 2007353, 2007354, 2007355, 2007356, 2007357, 2007358, 2007359, 2007360, 2007361, 2007362, 2007363, 2007364, 2007365, 2007366, 2007367, 2007368, 2007369, 2007370, 2007371, 2007372, 2007373, 2007374, 2007375, 2007376, 2007377, 2007378, 2007379, 2007380, 2007381, 2007382, 2007383, 2007384, 2007385, 2007386, 2007387, 2007388, 2007389, 2007390, 2007391, 2007392, 2007393, 2007394, 2007395, 2007396, 2007397, 2007398, 2007399, 2007400, 2007401, 2007402, 2007403, 2007404, 2007405, 2007406, 2007407, 2007408, 2007409, 2007410, 2007411, 2007412, 2007413, 2007414, 2007415, 2007416, 2007417, 2007418, 2007419, 2007420, 2007421, 2007422, 2007423, 2007424, 2007425, 2007426, 2007427, 2007428, 2007429, 2007430, 2007431, 2007432, 2007433, 2007434, 2007435, 2007436, 2007437, 2007438, 2007439, 2007440, 2007441, 2007442, 2007443, 2007444, 2007445, 2007446, 2007447, 2007448, 2007449, 2007450, 2007451, 2007452, 2007453, 2007454, 2007455, 2007456, 2007457, 2007458, 2007459, 2007460, 2007461, 2007462, 2007463, 2007464, 2007465, 2007466, 2007467, 2007468, 2007469, 2007470, 2007471, 2007472, 2007473, 2007474, 2007475, 2007476, 2007477, 2007478, 2007479, 2007480, 2007481, 2007482, 2007483, 2007484, 2007485, 2007486, 2007487, 2007488, 2007489, 2007490, 2007491, 2007492, 2007493, 2007494, 2007495, 2007496, 2007497, 2007498, 2007499, 2007500, 2007501, 2007502, 2007503, 2007504, 2007505, 2007506, 2007507, 2007508, 2007509, 2007510, 2007511, 2007512, 2007513, 2007514, 2007515, 2007516, 2007517, 2007518, 2007519, 2007520, 2007521, 2007522, 2007523, 2007524, 2007525, 2007526, 2007527, 2007528, 2007529, 2007530, 2007531, 2007532, 2007533, 2007534, 2007535, 2007536, 2007537, 2007538, 2007539, 2007540, 2007541, 2007542, 2007543, 2007544, 2007545, 2007546, 2007547, 2007548, 2007549, 2007550, 2007551, 2007552, 2007553, 2007554, 2007555, 2007556, 2007557, 2007558, 2007559, 2007560, 2007561, 2007562, 2007563, 2007564, 2007565, 2007566, 2007567, 2007568, 2007569, 2007570, 2007571, 2007572, 2007573, 2007574, 2007575, 2007576, 2007577, 2007578, 2007579, 2007580, 2007581
