# Supplementary material for: On the accuracy and precision of X-ray and neutron diffraction results as a function of resolution and the electron density model
Source: IUCrJ. 2020 Aug 25;7(Pt 5):920–33. doi: 10.1107/S2052252520010441 (PMC7467170; doi:10.1107/S2052252520010441)
Supplement: Supplementary file 1 [file m-07-00920-sup1.pdf]

Supplementary Materials

On Accuracy and Precision of X-Ray and Neutron Diffraction Results as a Function of Resolution and Electron Density Model

W. Fabiola Sanjuan-Szklarz[1], Magdalena Woińska[1], Sławomir Domagała[1], Paulina M. Dominiak[1], Simon Grabowsky[2], Dylan Jayatilaka[3], Matthias Gutmann[4], Krzysztof Woźniak[1*]

***Corresponding author: kwozniak@chem.uw.edu.pl**

Contents

Figure 1S. Dependencies of bond lengths and interatomic distances on data resolution and electron density model refined against X-ray and neutron data for: **(a)** C1-O1 bond length, **(b)** C1-O2, **(c)** O1-H1, **(d)** O(3)-H(3), **(e)** H2…O2 hydrogen bond, **(f)** H3…O2^{iii}, **(g)** O1…O3, **(h)** O3…O2^{ii}, **(i)** O3…O2^{iii} . Neutron data and results of periodic DFT calculations are given for the sake of reference.

Figure 2S. **Dependencies of the valence angles on data resolution and the electron density model refined against X-ray and neutron data for: (a)** C1-O1-H1, **(b)** H2-O3-H3**, (c)** O1-C1-C1, **(d)** O2-C1-C1, **(e) O3-**H2…O2^{ii} **(f) O3-**H3…O2^{iii}. Neutron data and results of periodic DFT calculations are given for the sake of reference..

Figure 3S. **Dependencies of the errors of the geometrical parameters on data resolution and the electron density model refined against X-ray and neutron data for: (a)** C1-O1, **(b)** C1-O2**, (c)** O1-H1, **(d)** O3-H3, **(e)** H2…O2^{ii} **(f)**H3…O2^{iii}**, (g) O1…O3, (h) O3…O2^{ii} (i)** O3…O2^{iii}**, (j)** C1-C1^{i}**, (k)** O3…H2**, (l)** H1…O3**. esd** stands for estimated standard deviation values and **ssd** for the sample standard deviation values.

Figure 4S. **Dependencies of the errors of valence angles on data resolution and the electron density model refined against X-ray and neutron data for: (a) O2-**C1-O1, **(b) O1-H1…O3, (c) C1-**O1-H1, **(d) H2-**O3-H3, **(e)** O1-C1-C1^{i} **(f) O2-C1-C1, (g) O2-C1-O1, (h) O1-H1…O3, (i)** O3-H2…O2^{ii}**, (j)** O3-H3…O2^{iii}. **esd** stands for estimated standard deviation values and **ssd** for the sample standard deviation values.

Figure 5S. Dependencies of the thermal parameters on the data resolution and the electron density model refined against X-ray and neutron data for: **(a)** U_{eq} for C1 atom, **(b)** U_{eq} for O1 atom, **(c)** U_{eq} for C1 atom, **(d)** U_{eq} for O3 atom, **(e)** U_{iso} for H1 atom, **(f)** U_{iso} for H2 atom, **(g)** U_{iso} for H3 atom. In the case of H-atoms, the plot contains Uiso values for IAM and UBDB, ELMAM2 and INVARIOM refinements and Ueq for the other methods.

Figure 6S. Dependencies of the unit cell parameters on data resolution and the electron density model refined against X-ray and neutron data for: **(a)** a, **(b)** b, **(c)** c, **(d)** β, **(e)** unit cell vol.

Figure 7S. Dependencies of residual electron density on data resolution and the electron density model refined against X-ray and neutron data for: **(a)** Max. residual electron density, **(b)** Min. residual electron density.

Table 1S. Numerical values for all parameters studied.

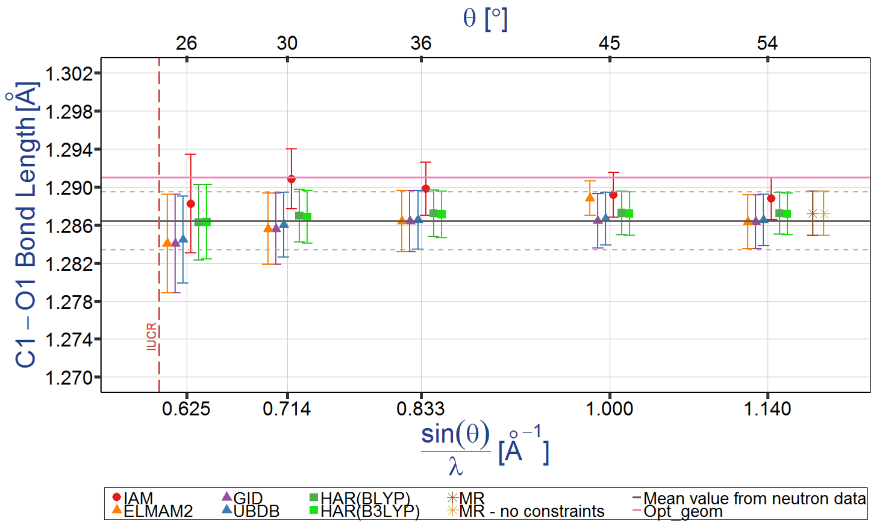
θ [°]
26 30 36 45 54
C1 – O1 Bond Length [Å]
IUCR
0.625 0.714 0.833 1.000 1.140
sin(θ)/λ [Å^-1]
IAM ELMAM2 GID UBDB HAR(BLYP) HAR(B3LYP) MR MR - no constraints Mean value from neutron data Opt_geom

(a)

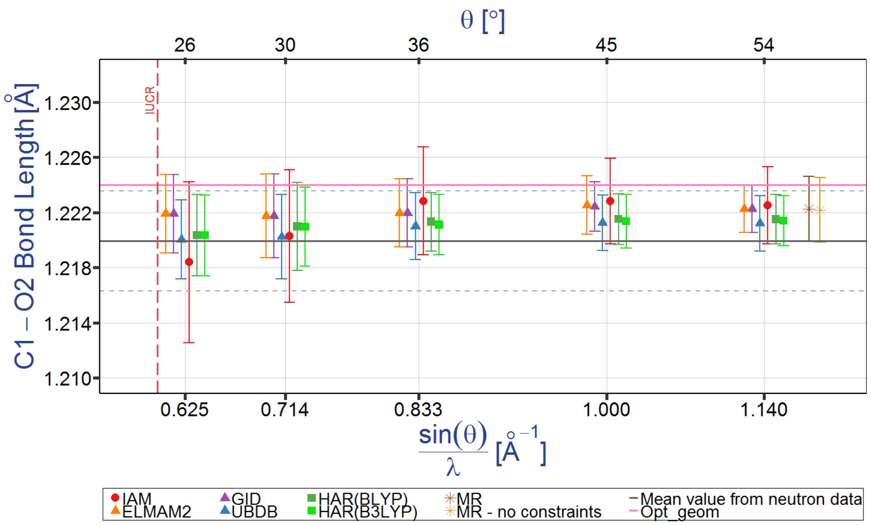
θ [°]
26 30 36 45 54
C1 – O2 Bond Length [Å]
IUCR
0.625 0.714 0.833 1.000 1.140
sin(θ)/λ [Å^-1]
IAM ELMAM2 GID UBDB HAR(BLYP) HAR(B3LYP) MR MR - no constraints Mean value from neutron data Opt_geom

(A

(b)

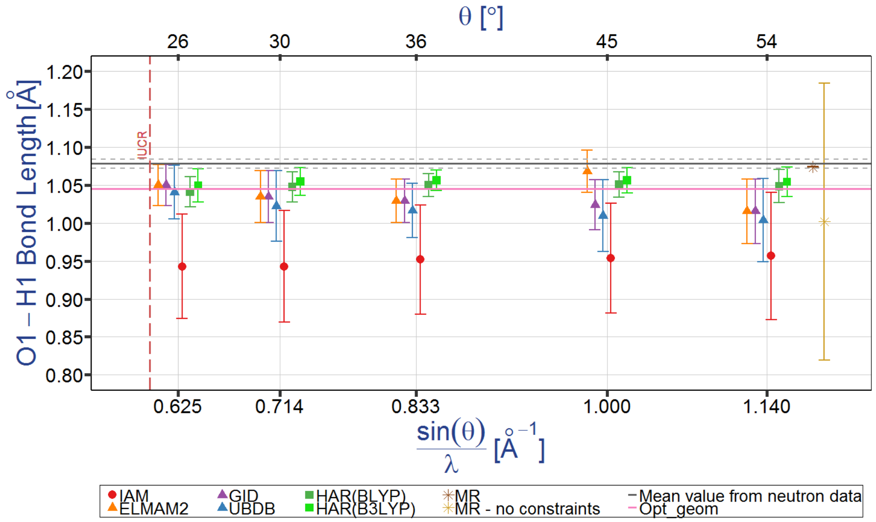
θ [°]
26 30 36 45 54
O1 – H1 Bond Length [Å]
IUCR
0.625 0.714 0.833 1.000 1.140
sin(θ)/λ [Å^-1]
IAM ELMAM2 GID UBDB HAR(BLYP) HAR(B3LYP) MR MR - no constraints Mean value from neutron data Opt_geom

(c)

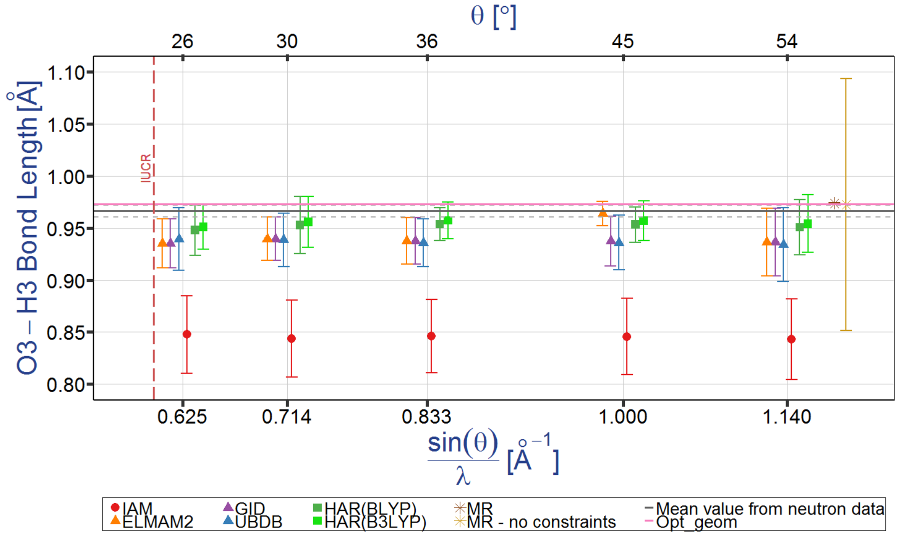
θ [°]
26 30 36 45 54
O3 – H3 Bond Length [Å]
IUCR
0.625 0.714 0.833 1.000 1.140
sin(θ)/λ [Å^-1]
IAM ELMAM2 GID UBDB HAR(BLYP) HAR(B3LYP) MR MR - no constraints Mean value from neutron data Opt_geom

(d)

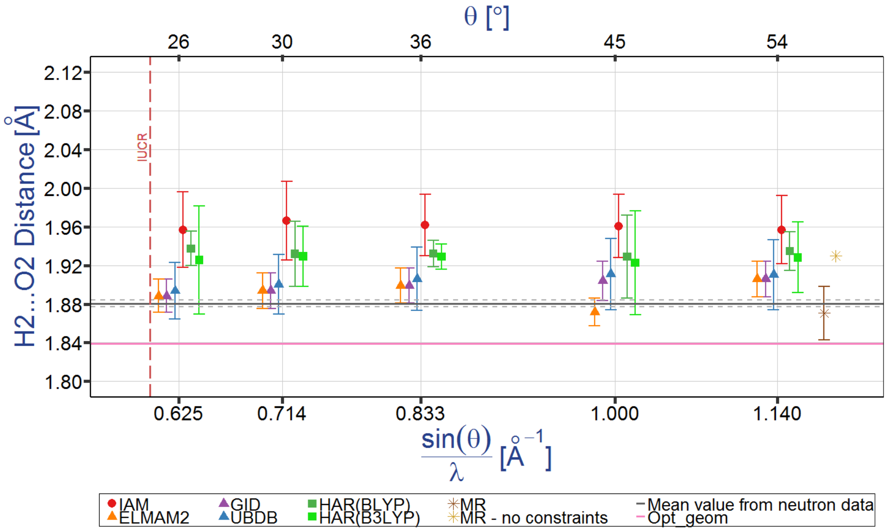
θ [°]
26
30
36
45
54
H2...O2 Distance [Å]
2.12
2.08
2.04
2.00
1.96
1.92
1.88
1.84
1.80
IUCR
0.625
0.714
0.833
1.000
1.140
sin(θ)/λ [Å^-1]
IAM
ELMAM2
GID
UBDB
HAR(BLYP)
HAR(B3LYP)
MR
MR - no constraints
Mean value from neutron data
Opt_geom

(e)

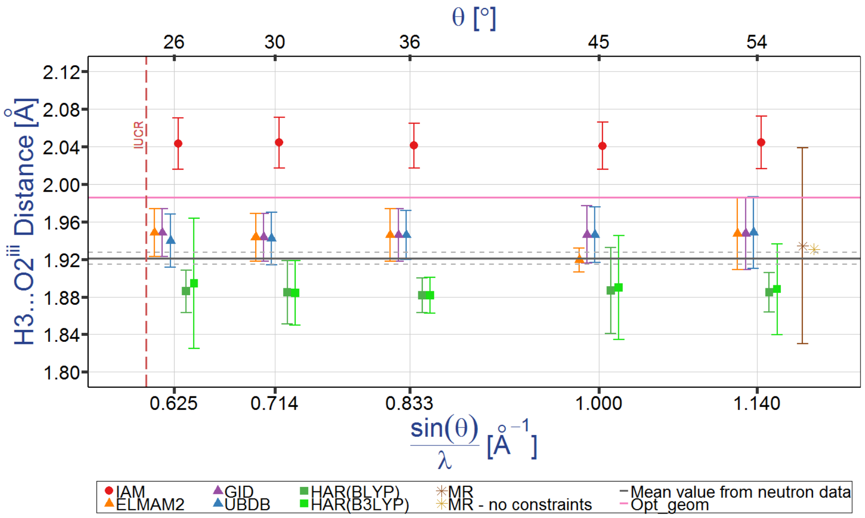
θ [°]
26
30
36
45
54
H3...O2iii Distance [Å]
2.12
2.08
2.04
2.00
1.96
1.92
1.88
1.84
1.80
IUCR
0.625
0.714
0.833
1.000
1.140
sin(θ)/λ [Å^-1]
IAM
ELMAM2
GID
UBDB
HAR(BLYP)
HAR(B3LYP)
MR
MR - no constraints
Mean value from neutron data
Opt_geom

(f)

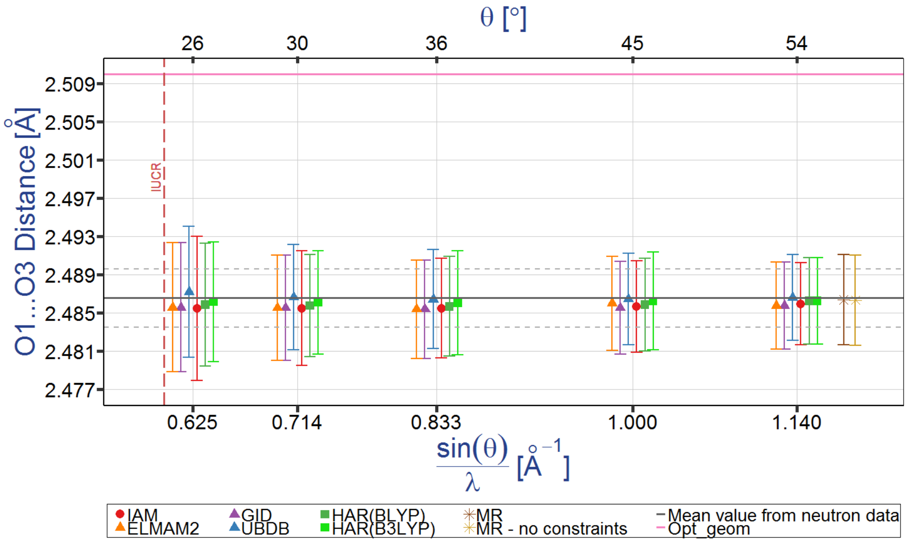
θ [°]
26
30
36
45
54
O1...O3 Distance [Å]
2.509
2.505
2.501
2.497
2.493
2.489
2.485
2.481
2.477
IUCR
0.625
0.714
0.833
1.000
1.140
sin(θ)/λ [Å^-1]
IAM
ELMAM2
GID
UBDB
HAR(BLYP)
HAR(B3LYP)
MR
MR - no constraints
Mean value from neutron data
Opt_geom

(g)

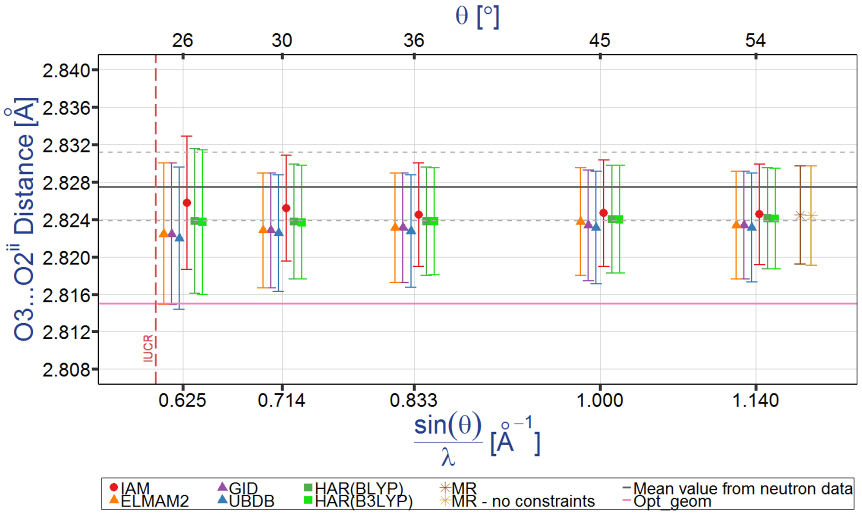
θ [°]
26
30
36
45
54
O3...O2ii Distance [Å]
2.840
2.836
2.832
2.828
2.824
2.820
2.816
2.812
2.808
IUCR
0.625
0.714
0.833
1.000
1.140
sin(θ)/λ [Å^-1]
IAM
ELMAM2
GID
UBDB
HAR(BLYP)
HAR(B3LYP)
MR
MR - no constraints
Mean value from neutron data
Opt_geom

(h)

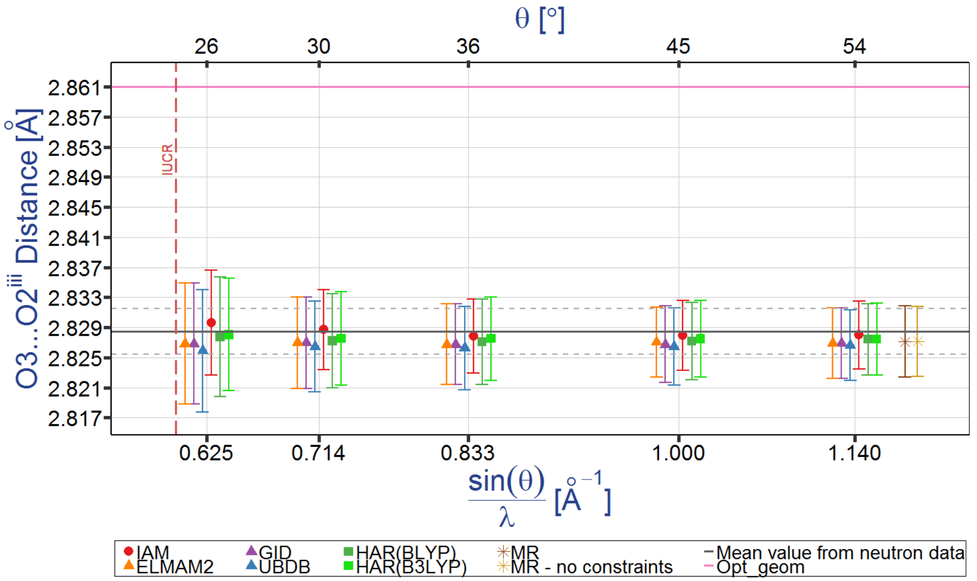

(i)

Figure 1S. Dependencies of bond lengths and interatomic distances on data resolution and electron density model refined against X-ray and neutron data for: **(a)** C1O1 bond length, **(b)** C1-O2, **(c)** O1-H1, **(d)** O(3)-H(3), **(e)** H2…O2 hydrogen bond, **(f)** H3…O2^{iii}, **(g)** O1…O3, **(h)** O3…O2^{ii}, **(i)** O3…O2^{iii} . Neutron data and results of periodic DFT calculations are given for the sake of reference.

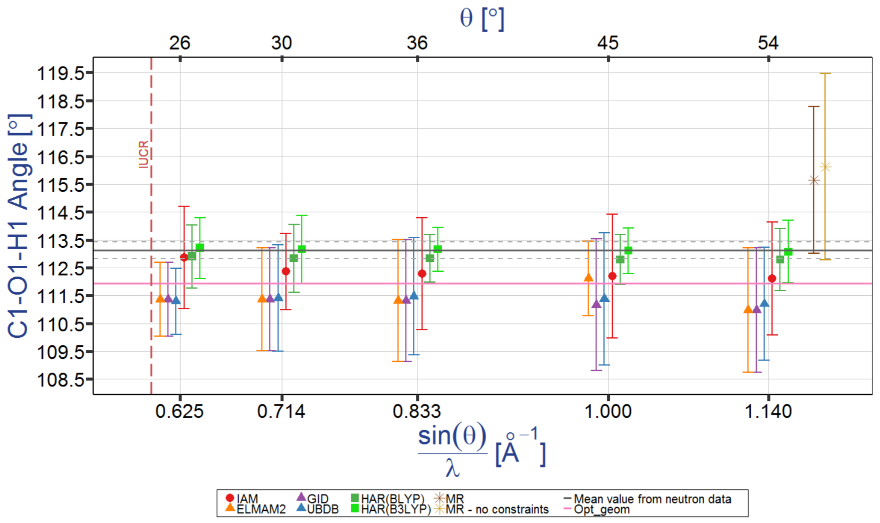
θ [°]
26
30
36
45
54
C1-O1-H1 Angle [°]
IUCR
sin(θ)/λ [Å⁻¹]
0.625
0.714
0.833
1.000
1.140
IAM
ELMAM2
GID
UBDB
HAR(BLYP)
HAR(B3LYP)
MR
MR - no constraints
Mean value from neutron data
Opt_geom

(a)

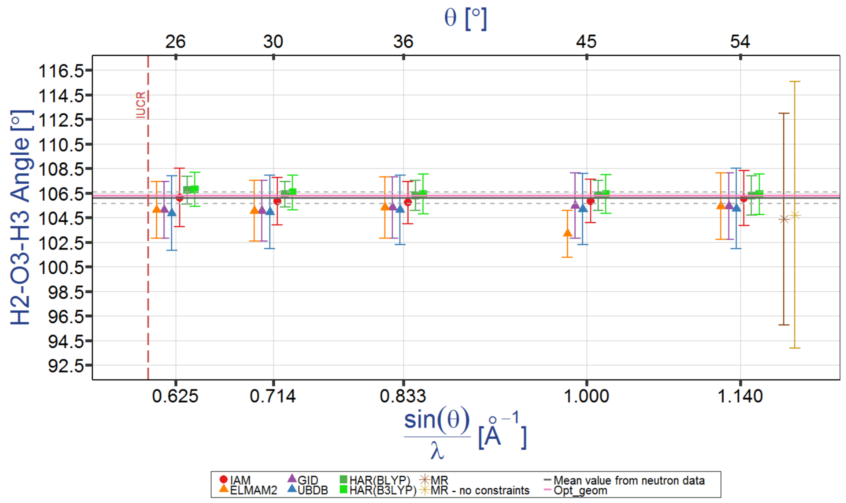
θ [°]
26
30
36
45
54
H2-O3-H3 Angle [°]
IUCR
sin(θ)/λ [Å⁻¹]
0.625
0.714
0.833
1.000
1.140
IAM
ELMAM2
GID
UBDB
HAR(BLYP)
HAR(B3LYP)
MR
MR - no constraints
Mean value from neutron data
Opt_geom

(b)

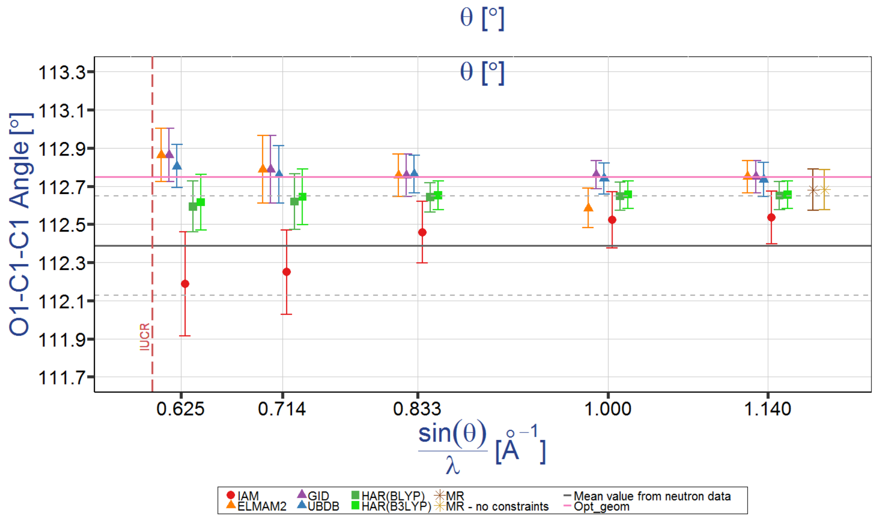
θ [°]
θ [°]
O1-C1-C1 Angle [°]
IUCR
sin(θ)/λ [Å⁻¹]
0.625
0.714
0.833
1.000
1.140
IAM
ELMAM2
GID
UBDB
HAR(BLYP)
HAR(B3LYP)
MR
MR - no constraints
Mean value from neutron data
Opt_geom

(c)

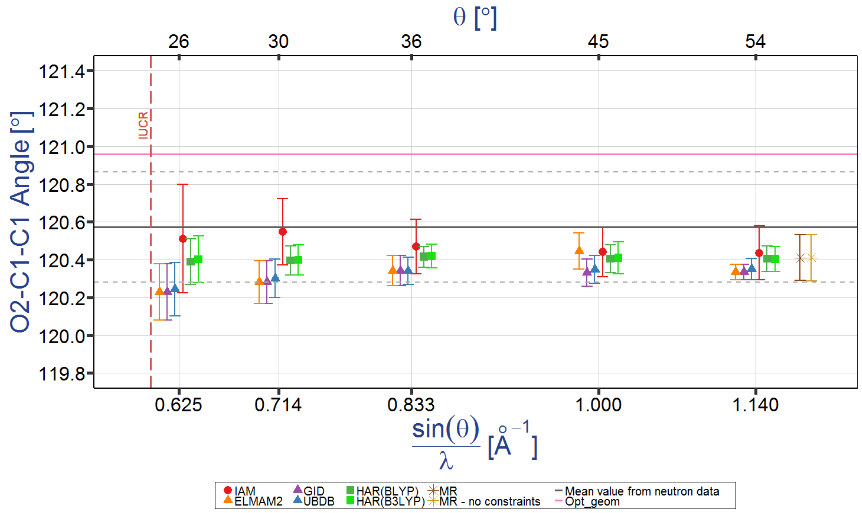
θ [°]
26
30
36
45
54
O2-C1-C1 Angle [°]
IUCR
sin(θ)/λ [Å⁻¹]
0.625
0.714
0.833
1.000
1.140
IAM
ELMAM2
GID
UBDB
HAR(BLYP)
HAR(B3LYP)
MR
MR - no constraints
Mean value from neutron data
Opt_geom

(d)

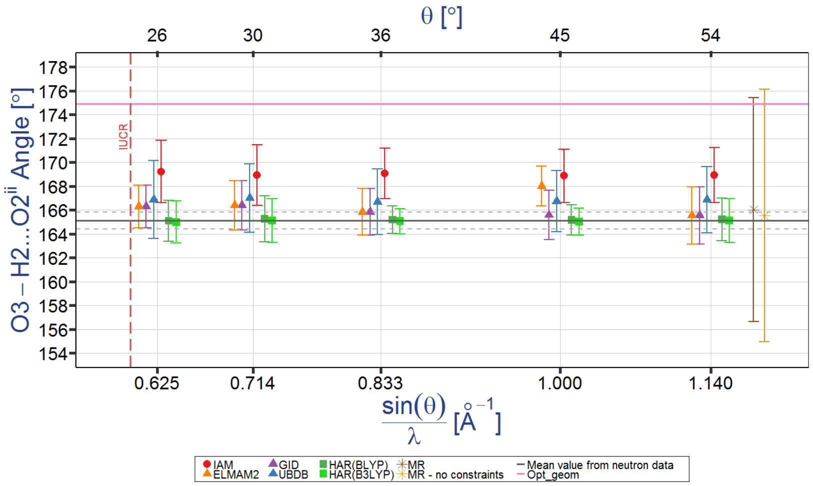

(e)

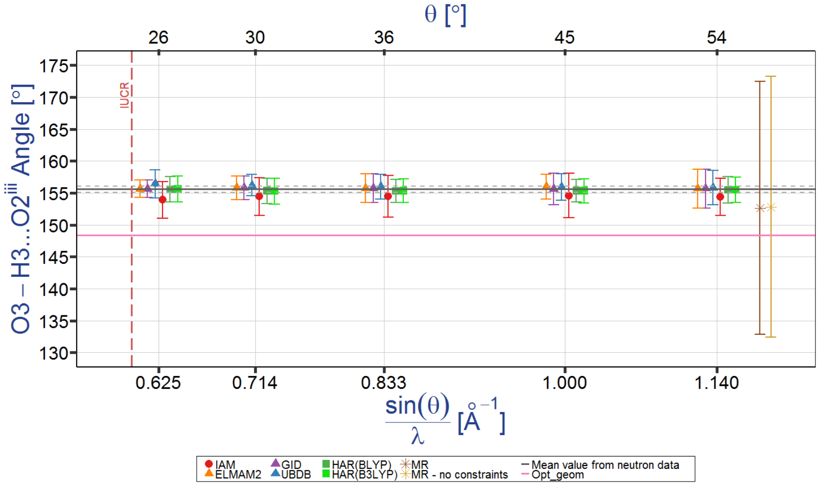

(f)

Figure 2S. Dependencies of the valence angles on data resolution and the electron density model refined against X-ray and neutron data for: (a) C1-O1-H1, **(b)** H2-O3-H3**, (c)** O1-C1-C1, **(d)** O2-C1-C1, **(e) O3**-H2…O2^{ii} **(f) O3**-H3…O2^{iii}. Neutron data and results of periodic DFT calculations are given for the sake of reference.

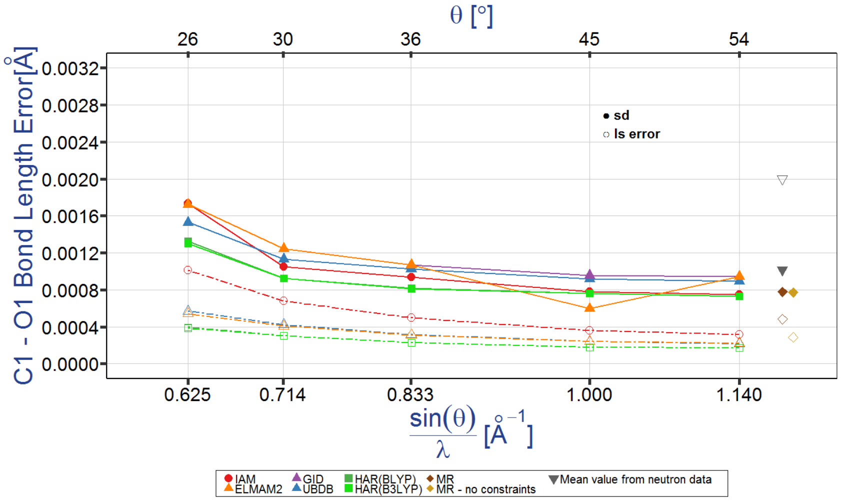
θ [°]
26 30 36 45 54
C1 - O1 Bond Length Error[Å]
0.0032 0.0028 0.0024 0.0020 0.0016 0.0012 0.0008 0.0004 0.0000
sd
ls error
0.625 0.714 0.833 1.000 1.140
sin(θ)/λ [Å^-1]
IAM ELMAM2 GID UBDB HAR(BLYP) HAR(B3LYP) MR MR - no constraints Mean value from neutron data

(a)

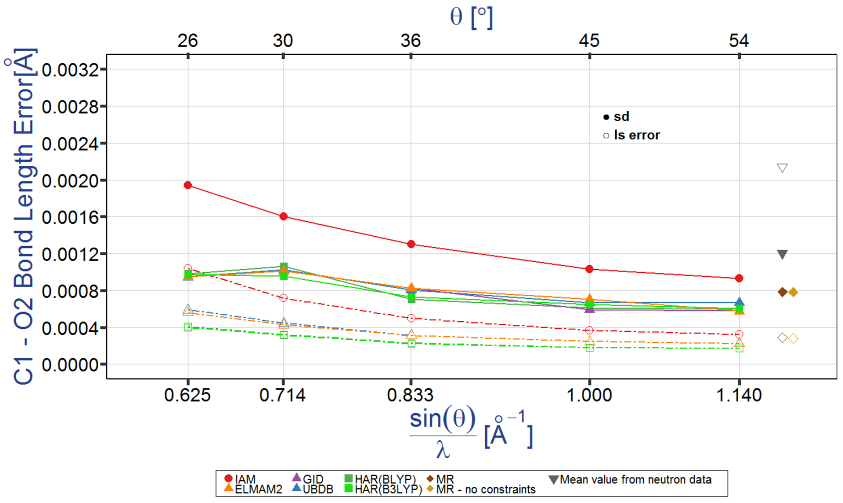
θ [°]
26 30 36 45 54
C1 - O2 Bond Length Error[Å]
0.0032 0.0028 0.0024 0.0020 0.0016 0.0012 0.0008 0.0004 0.0000
sd
ls error
0.625 0.714 0.833 1.000 1.140
sin(θ)/λ [Å^-1]
IAM ELMAM2 GID UBDB HAR(BLYP) HAR(B3LYP) MR MR - no constraints Mean value from neutron data

(b)

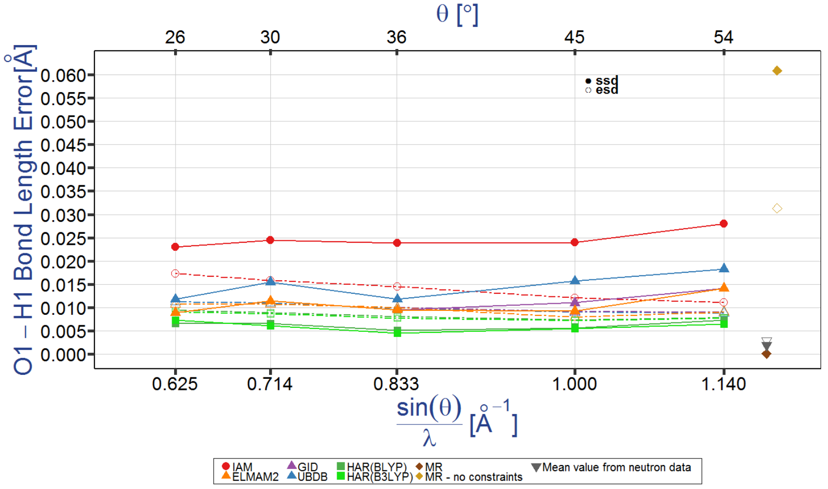
θ [°]
26 30 36 45 54
O1 – H1 Bond Length Error[Å]
0.060 0.055 0.050 0.045 0.040 0.035 0.030 0.025 0.020 0.015 0.010 0.005 0.000
ssd
esd
0.625 0.714 0.833 1.000 1.140
sin(θ)/λ [Å^-1]
IAM ELMAM2 GID UBDB HAR(BLYP) HAR(B3LYP) MR MR - no constraints Mean value from neutron data

(c)

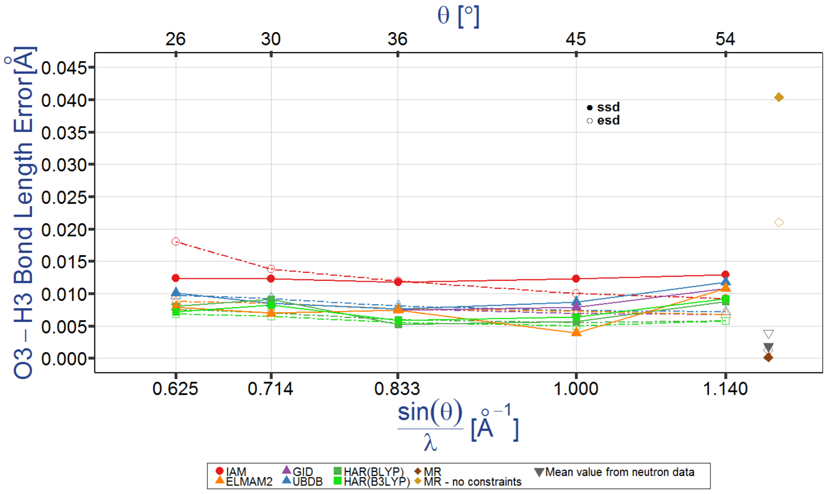
θ [°]
26 30 36 45 54
O3 – H3 Bond Length Error[Å]
0.045 0.040 0.035 0.030 0.025 0.020 0.015 0.010 0.005 0.000
ssd
esd
0.625 0.714 0.833 1.000 1.140
sin(θ)/λ [Å^-1]
IAM ELMAM2 GID UBDB HAR(BLYP) HAR(B3LYP) MR MR - no constraints Mean value from neutron data

(d)

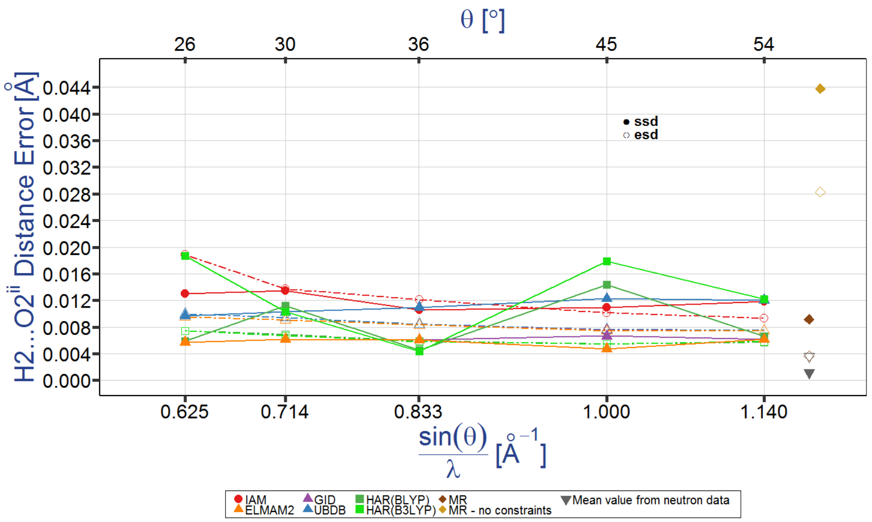
θ [°]
26 30 36 45 54
H2...O2ii Distance Error [Å]
0.000 0.004 0.008 0.012 0.016 0.020 0.024 0.028 0.032 0.036 0.040 0.044
0.625 0.714 0.833 1.000 1.140
sin(θ)/λ [Å⁻¹]
ssd
esd
IAM
ELMAM2
GID
UBDB
HAR(BLYP)
HAR(B3LYP)
MR
MR - no constraints
Mean value from neutron data

(e)

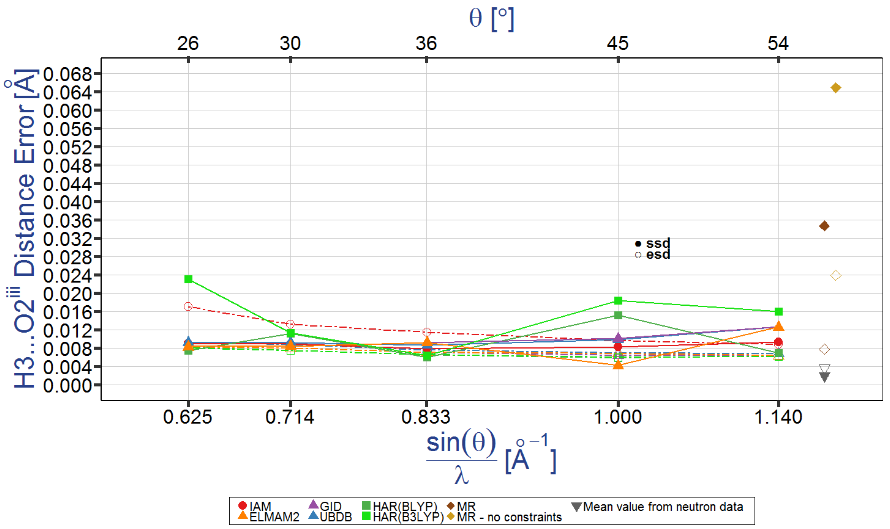
θ [°]
26 30 36 45 54
H3...O2iii Distance Error [Å]
0.000 0.004 0.008 0.012 0.016 0.020 0.024 0.028 0.032 0.036 0.040 0.044 0.048 0.052 0.056 0.060 0.064 0.068
0.625 0.714 0.833 1.000 1.140
sin(θ)/λ [Å⁻¹]
ssd
esd
IAM
ELMAM2
GID
UBDB
HAR(BLYP)
HAR(B3LYP)
MR
MR - no constraints
Mean value from neutron data

(f)

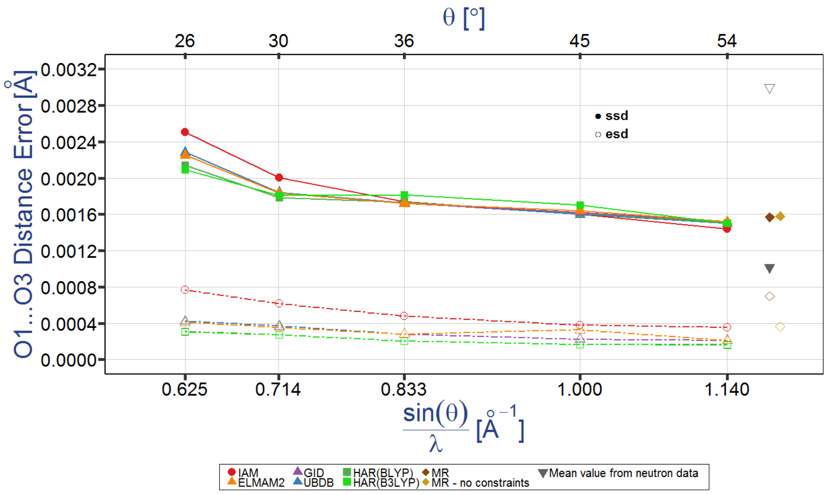
θ [°]
26 30 36 45 54
O1...O3 Distance Error [Å]
0.0000 0.0004 0.0008 0.0012 0.0016 0.0020 0.0024 0.0028 0.0032
0.625 0.714 0.833 1.000 1.140
sin(θ)/λ [Å⁻¹]
ssd
esd
IAM
ELMAM2
GID
UBDB
HAR(BLYP)
HAR(B3LYP)
MR
MR - no constraints
Mean value from neutron data

(g)

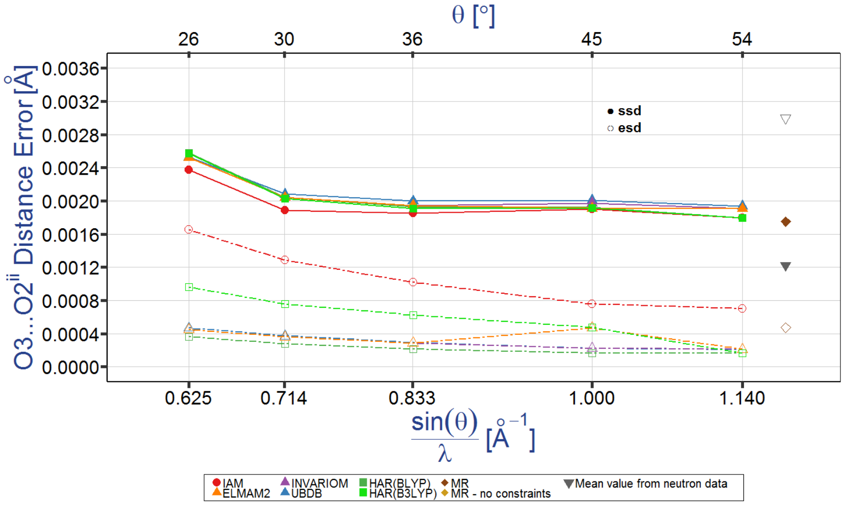
θ [°]
26 30 36 45 54
O3...O2ii Distance Error [Å]
0.0000 0.0004 0.0008 0.0012 0.0016 0.0020 0.0024 0.0028 0.0032 0.0036
0.625 0.714 0.833 1.000 1.140
sin(θ)/λ [Å⁻¹]
ssd
esd
IAM
ELMAM2
INVARIOM
UBDB
HAR(BLYP)
HAR(B3LYP)
MR
MR - no constraints
Mean value from neutron data

(h)

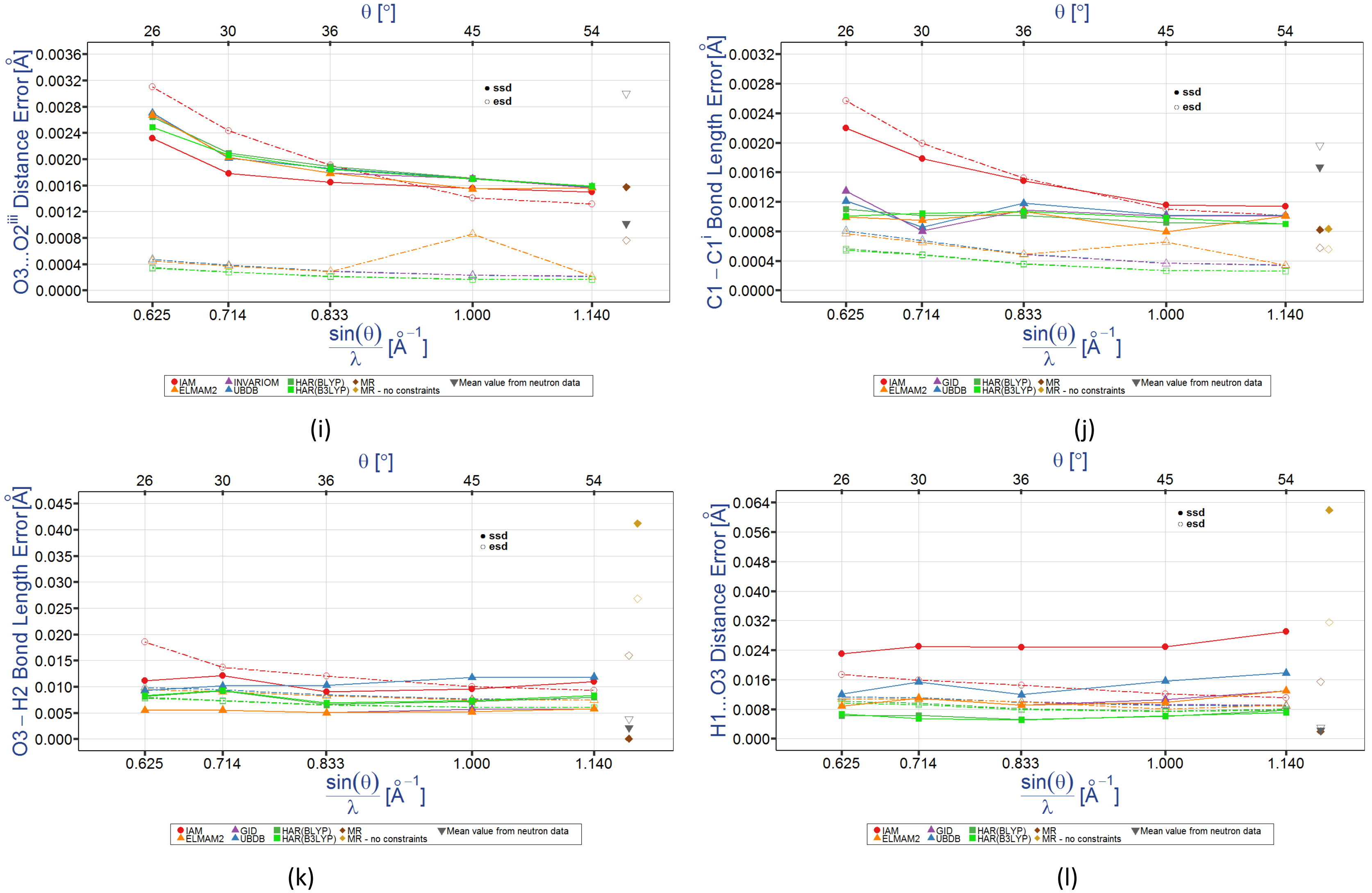

Figure 3S. **Dependencies of the errors of the geometrical parameters on data resolution and the electron density model refined against X-ray and neutron data for: (a)** C1-O1, **(b)** C1-O2, **(c)** O1-H1, **(d)** O3-H3, **(e)** H2…O2^{ii}, **(f)**H3…O2^{iii}, **(g) O1…O3, (h) O3…O2^{ii} (i)** O3…O2^{iii}, **(j)** C1-C1^{i}, **(k)** O3…H2, **(l)** H1…O3. **esd** stands for estimated standard deviation values and **ssd** for the sample standard deviation values.

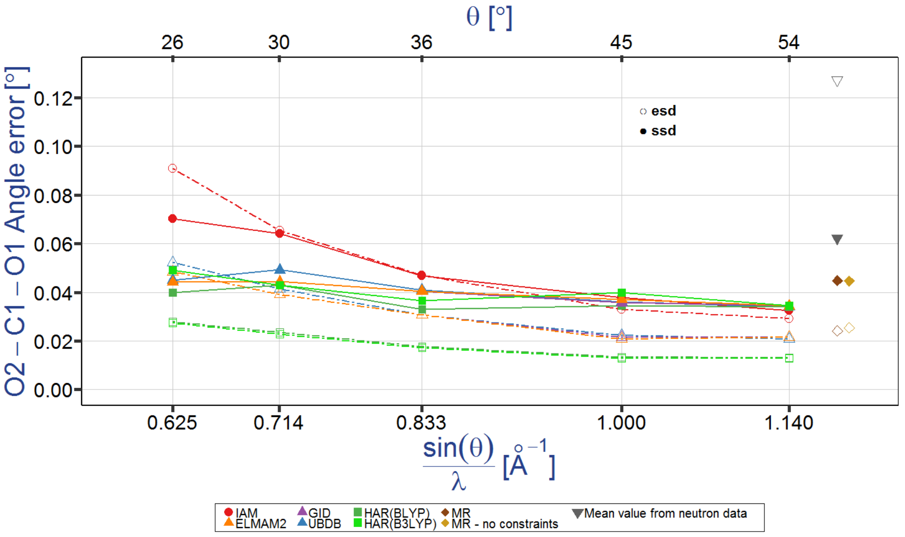
θ [°]
26
30
36
45
54
O2 – C1 – O1 Angle error [°]
0.12
0.10
0.08
0.06
0.04
0.02
0.00
esd
ssd
0.625
0.714
0.833
1.000
1.140
sin(θ)/λ [Å⁻¹]
IAM
ELMAM2
GID
UBDB
HAR(BLYP)
HAR(B3LYP)
MR
MR - no constraints
Mean value from neutron data

(a)

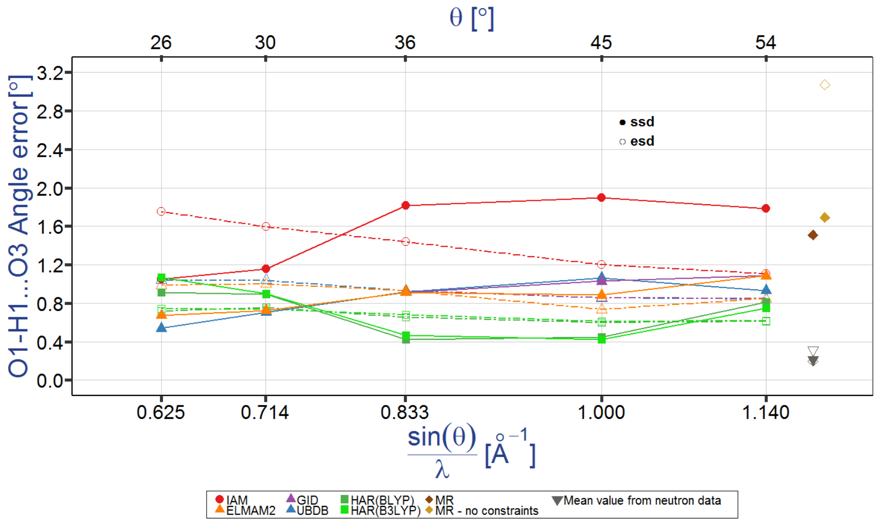
θ [°]
26
30
36
45
54
O1-H1…O3 Angle error [°]
3.2
2.8
2.4
2.0
1.6
1.2
0.8
0.4
0.0
ssd
esd
0.625
0.714
0.833
1.000
1.140
sin(θ)/λ [Å⁻¹]
IAM
ELMAM2
GID
UBDB
HAR(BLYP)
HAR(B3LYP)
MR
MR - no constraints
Mean value from neutron data

(b)

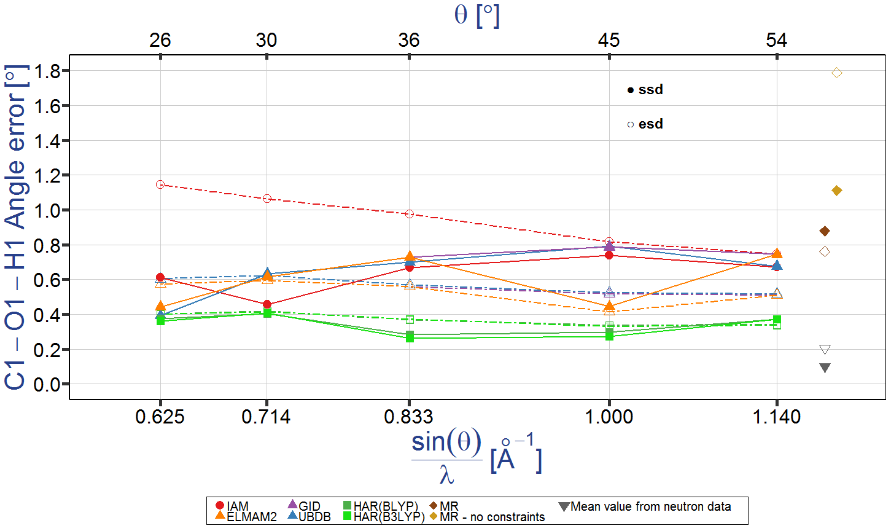
θ [°]
26
30
36
45
54
C1 – O1 – H1 Angle error [°]
1.8
1.6
1.4
1.2
1.0
0.8
0.6
0.4
0.2
0.0
ssd
esd
0.625
0.714
0.833
1.000
1.140
sin(θ)/λ [Å⁻¹]
IAM
ELMAM2
GID
UBDB
HAR(BLYP)
HAR(B3LYP)
MR
MR - no constraints
Mean value from neutron data

(c)

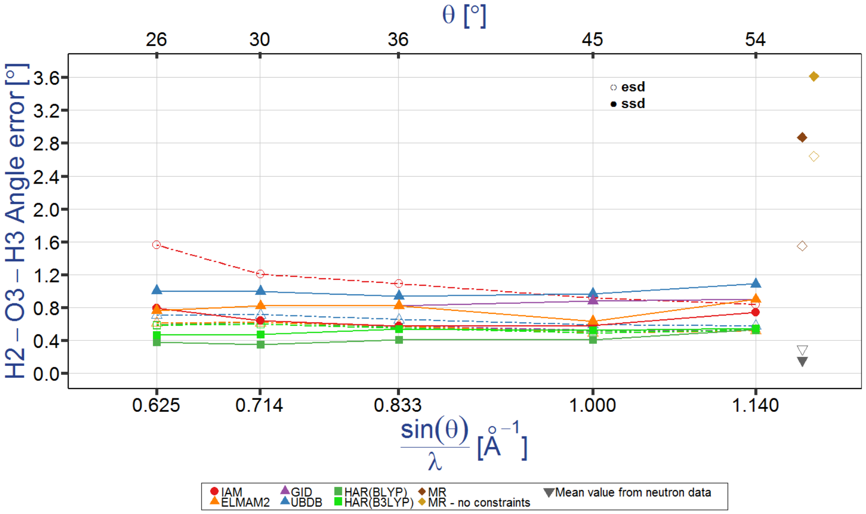
θ [°]
26
30
36
45
54
H2 – O3 – H3 Angle error [°]
3.6
3.2
2.8
2.4
2.0
1.6
1.2
0.8
0.4
0.0
esd
ssd
0.625
0.714
0.833
1.000
1.140
sin(θ)/λ [Å⁻¹]
IAM
ELMAM2
GID
UBDB
HAR(BLYP)
HAR(B3LYP)
MR
MR - no constraints
Mean value from neutron data

(d)

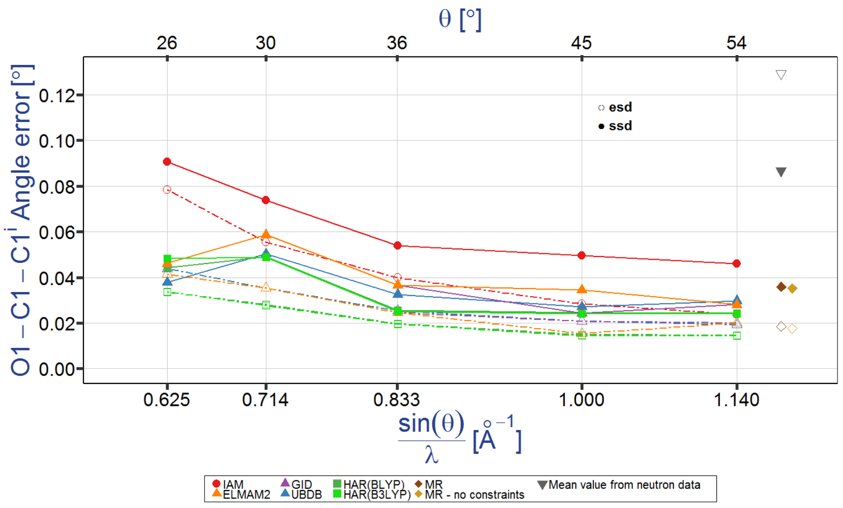
θ [°]
26
30
36
45
54
O1 – C1 – C1ⁱ Angle error [°]
0.12
0.10
0.08
0.06
0.04
0.02
0.00
esd
ssd
0.625
0.714
0.833
1.000
1.140
sin(θ)/λ [Å⁻¹]
IAM
ELMAM2
GID
UBDB
HAR(BLYP)
HAR(B3LYP)
MR
MR - no constraints
Mean value from neutron data

(e)

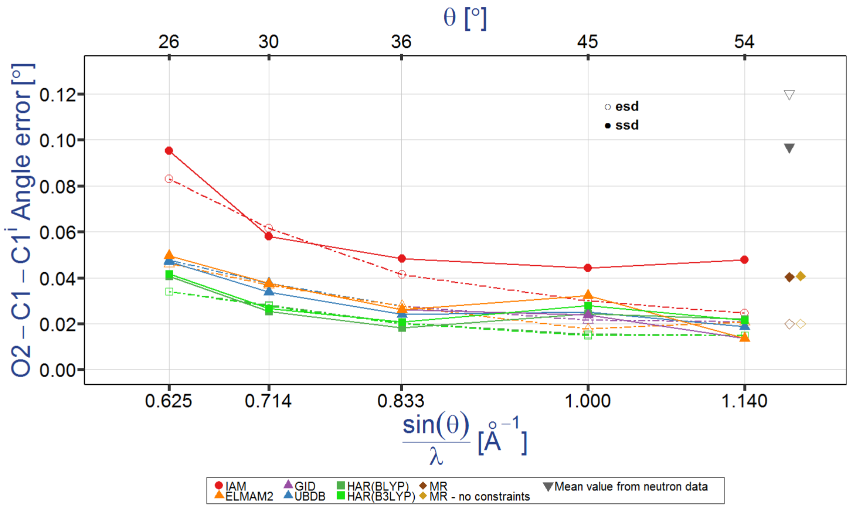
θ [°]
26
30
36
45
54
O2 – C1 – C1ⁱ Angle error [°]
0.12
0.10
0.08
0.06
0.04
0.02
0.00
esd
ssd
0.625
0.714
0.833
1.000
1.140
sin(θ)/λ [Å⁻¹]
IAM
ELMAM2
GID
UBDB
HAR(BLYP)
HAR(B3LYP)
MR
MR - no constraints
Mean value from neutron data

(f)

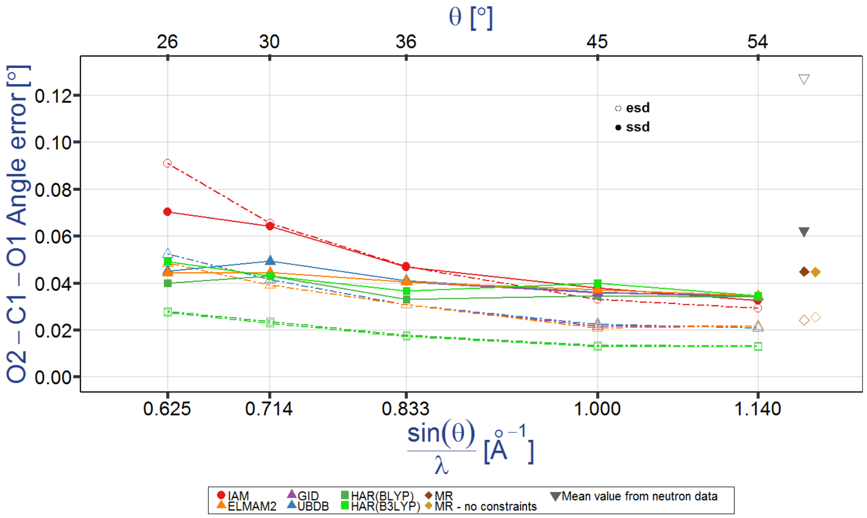
θ [°]
26
30
36
45
54
O2 – C1 – O1 Angle error [°]
0.12
0.10
0.08
0.06
0.04
0.02
0.00
esd
ssd
0.625
0.714
0.833
1.000
1.140
sin(θ)/λ [Å⁻¹]
IAM
ELMAM2
GID
UBDB
HAR(BLYP)
HAR(B3LYP)
MR
MR - no constraints
Mean value from neutron data

(g)

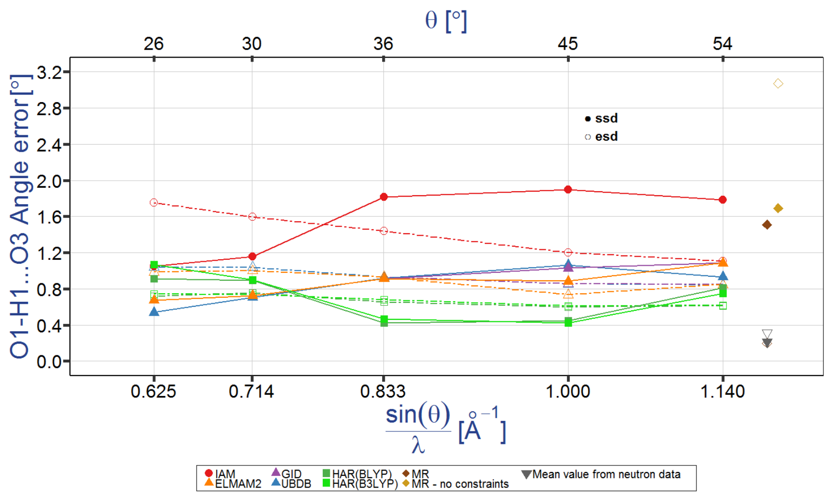
θ [°]
26
30
36
45
54
O1-H1...O3 Angle error [°]
3.2
2.8
2.4
2.0
1.6
1.2
0.8
0.4
0.0
ssd
esd
0.625
0.714
0.833
1.000
1.140
sin(θ)/λ [Å⁻¹]
IAM
ELMAM2
GID
UBDB
HAR(BLYP)
HAR(B3LYP)
MR
MR - no constraints
Mean value from neutron data

(h)

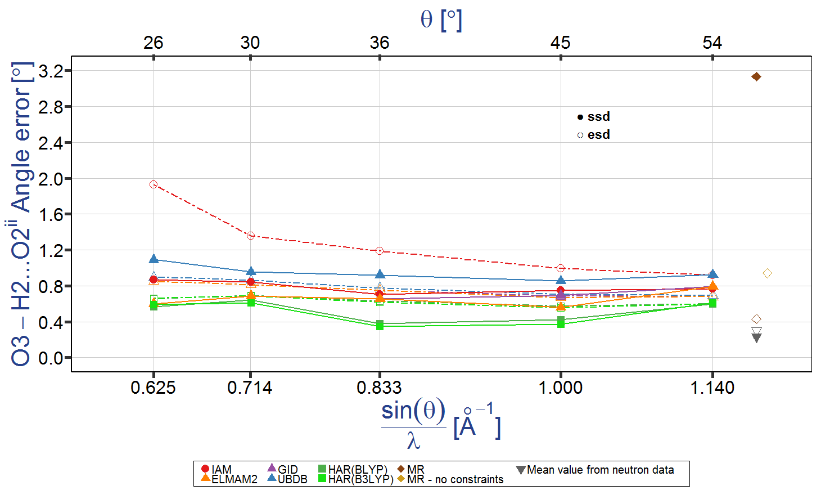

(i)

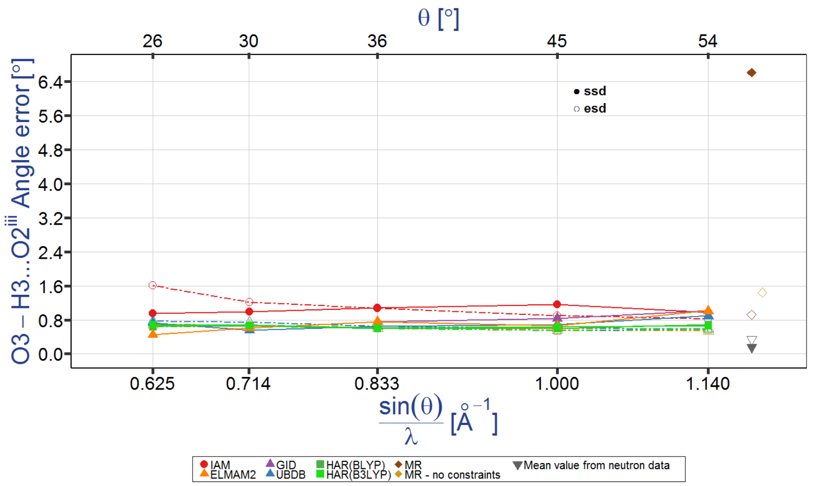

(j)

Figure 4S. **Dependencies of the errors of valence angles on data resolution and the electron density model refined against X-ray and neutron data for: (a) O2-**C1-O1, **(b) O1-H1…O3, (c) C1-**O1-H1, **(d) H2-**O3-H3, **(e)** O1-C1-C1^{i} **(f) O2-C1-C1, (g) O2-C1-O1, (h) O1-H1…O3, (i)** O3-H2…O2^{ii}**, (j)** O3-H3…O2^{iii}. **esd** stands for estimated standard deviation values and **ssd** for the sample standard deviation values.

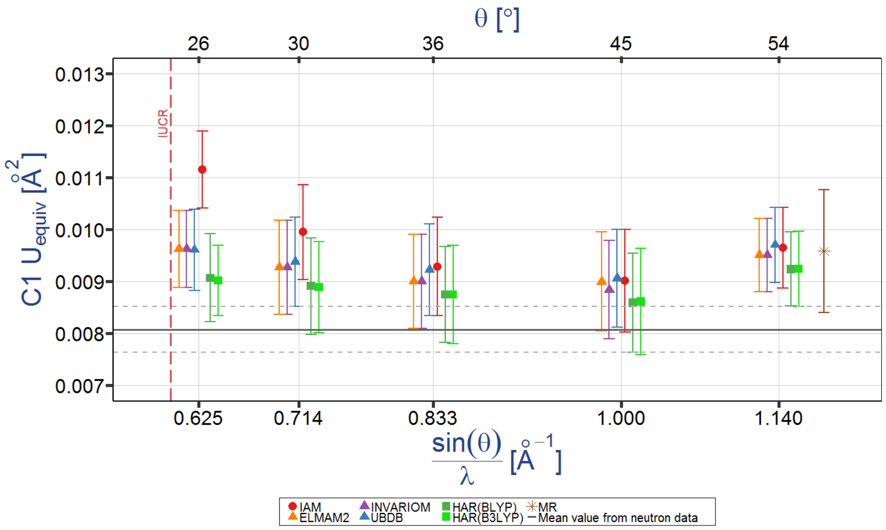
θ [°]
26
30
36
45
54
C1 U_{equiv} [Å^2]
0.013
0.012
0.011
0.010
0.009
0.008
0.007
IUCR
0.625
0.714
0.833
1.000
1.140
$\frac{\sin(\theta)}{\lambda}$ [Å^{-1}]
IAM
ELMAM2
INVARIOM
UBDB
HAR(BLYP)
HAR(B3LYP)
MR
Mean value from neutron data

(a)

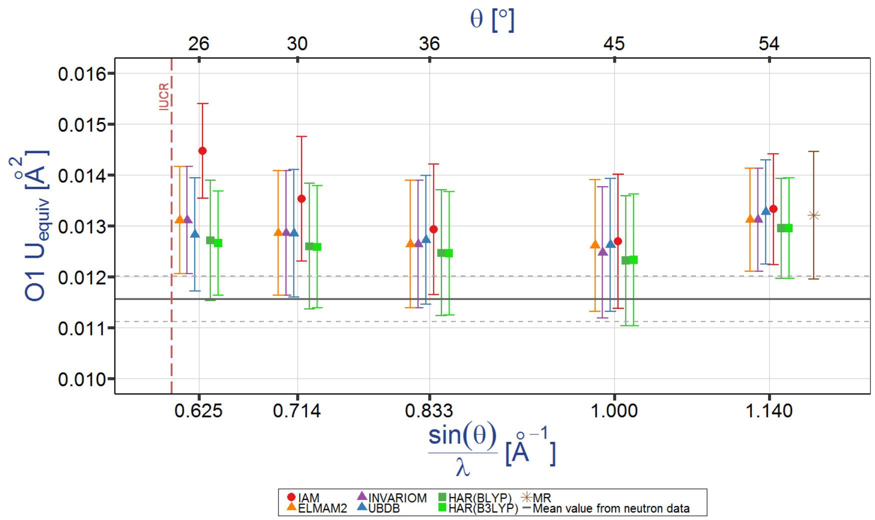
θ [°]
26
30
36
45
54
O1 U_{equiv} [Å^2]
0.016
0.015
0.014
0.013
0.012
0.011
0.010
IUCR
0.625
0.714
0.833
1.000
1.140
$\frac{\sin(\theta)}{\lambda}$ [Å^{-1}]
IAM
ELMAM2
INVARIOM
UBDB
HAR(BLYP)
HAR(B3LYP)
MR
Mean value from neutron data

(b)

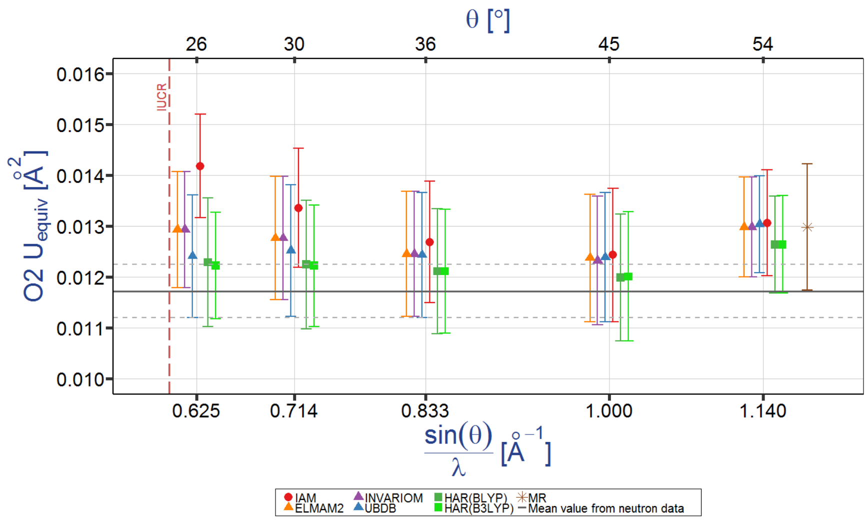
θ [°]
26
30
36
45
54
O2 U_{equiv} [Å^2]
0.016
0.015
0.014
0.013
0.012
0.011
0.010
IUCR
0.625
0.714
0.833
1.000
1.140
$\frac{\sin(\theta)}{\lambda}$ [Å^{-1}]
IAM
ELMAM2
INVARIOM
UBDB
HAR(BLYP)
HAR(B3LYP)
MR
Mean value from neutron data

(c)

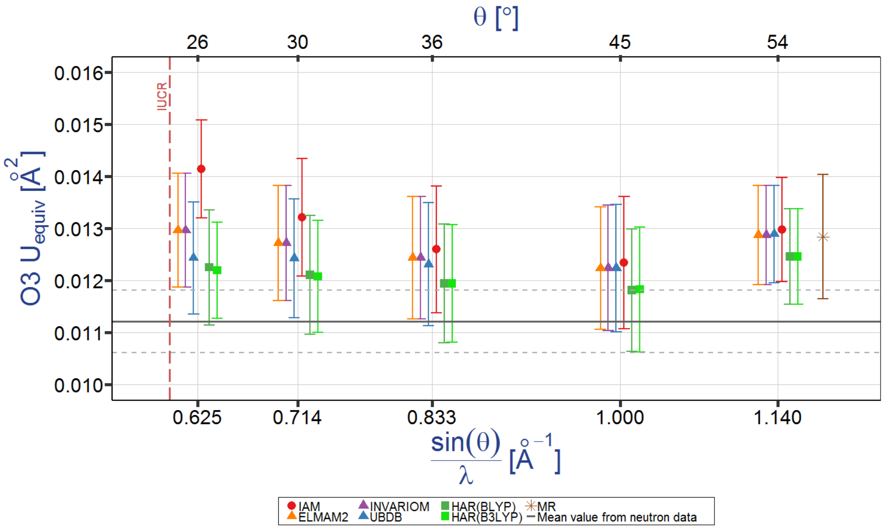
θ [°]
26
30
36
45
54
O3 U_{equiv} [Å^2]
0.016
0.015
0.014
0.013
0.012
0.011
0.010
IUCR
0.625
0.714
0.833
1.000
1.140
$\frac{\sin(\theta)}{\lambda}$ [Å^{-1}]
IAM
ELMAM2
INVARIOM
UBDB
HAR(BLYP)
HAR(B3LYP)
MR
Mean value from neutron data

(d)

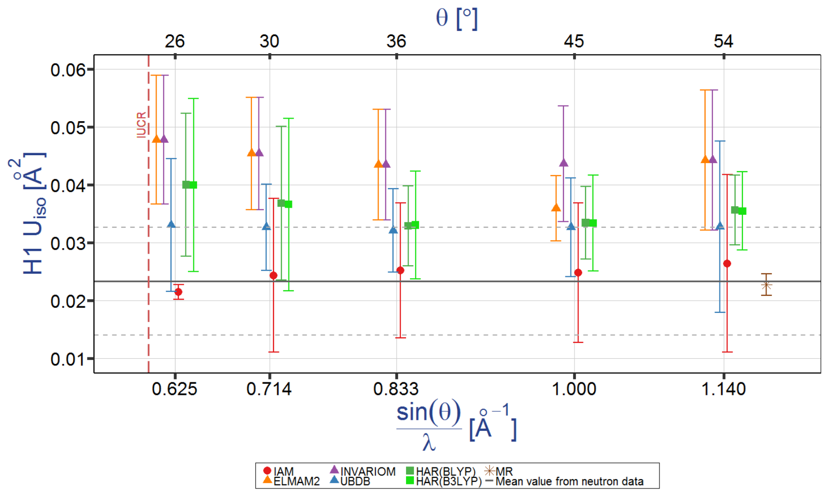

(e)

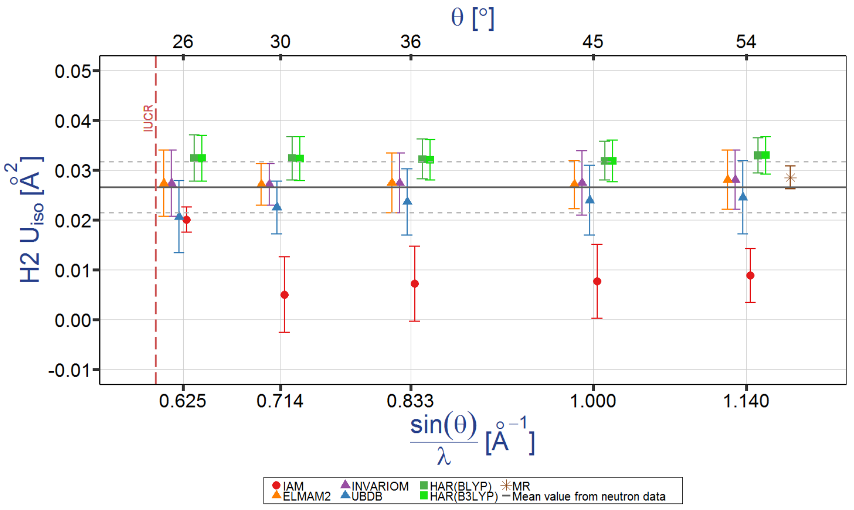

(f)

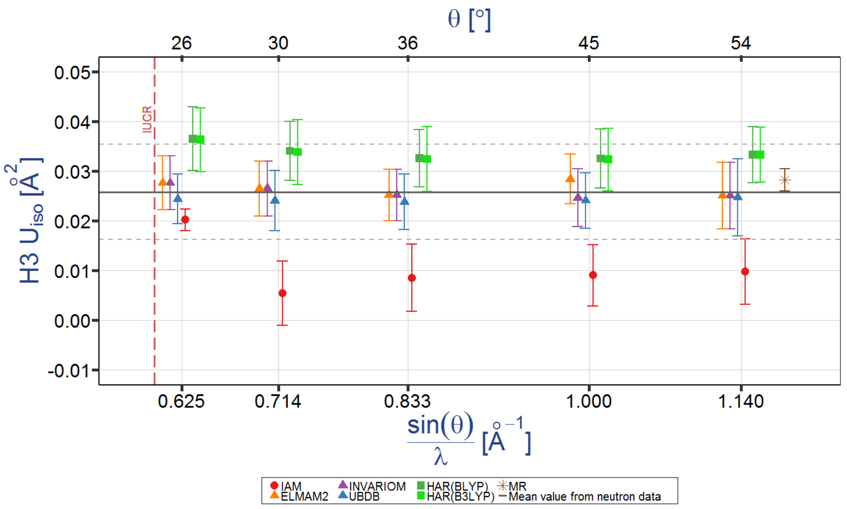

(g)

Figure 5S. Dependencies of the thermal parameters on the data resolution and the electron density model refined against X-ray and neutron data for: **(a)** U_{eq} for C1 atom, **(b)** U_{eq} for O1 atom, **(c)** U_{eq} for C1 atom, **(d)** U_{eq} for O3 atom, **(e)** U_{iso} for H1 atom, **(f)** U_{iso} for H2 atom, **(g)** U_{iso} for H3 atom. In the case of H-atoms, the plot contains Uiso values for IAM and UBDB, ELMAM2 and INVARIOM refinements and Ueq for the other methods.

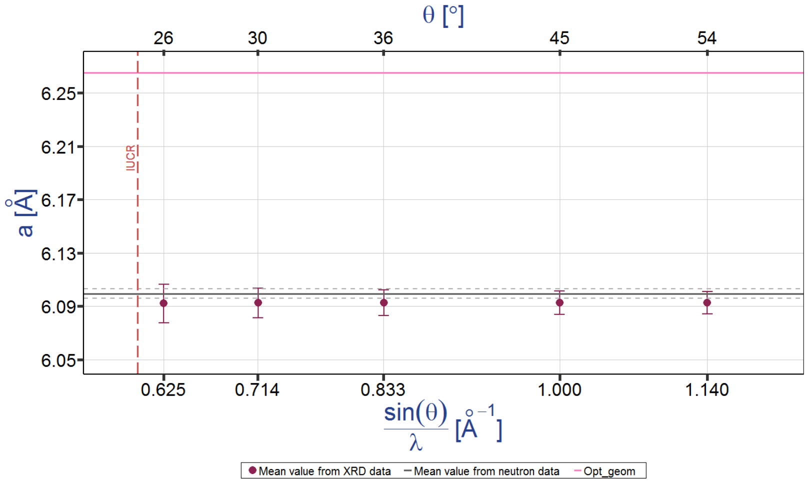
θ [°]
26
30
36
45
54
6.25
6.21
6.17
6.13
6.09
6.05
a [Å]
IUCR
0.625
0.714
0.833
1.000
1.140
sin(θ)/λ [Å^-1]
Mean value from XRD data
Mean value from neutron data
Opt_geom

(a)

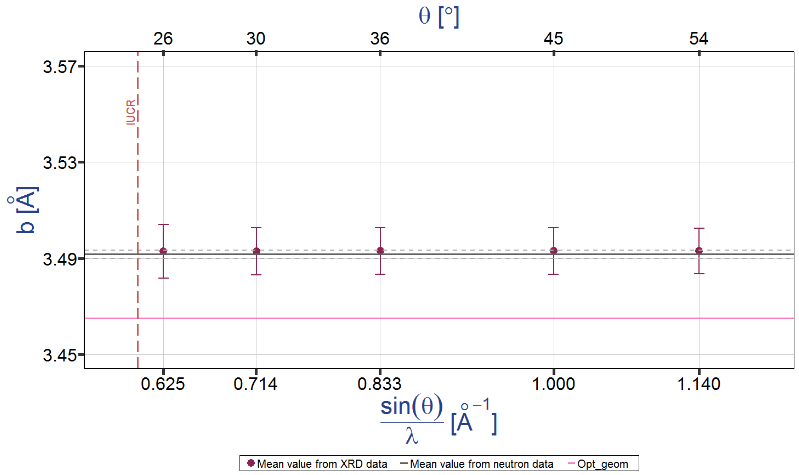
θ [°]
26
30
36
45
54
3.57
3.53
3.49
3.45
b [Å]
IUCR
0.625
0.714
0.833
1.000
1.140
sin(θ)/λ [Å^-1]
Mean value from XRD data
Mean value from neutron data
Opt_geom

(b)

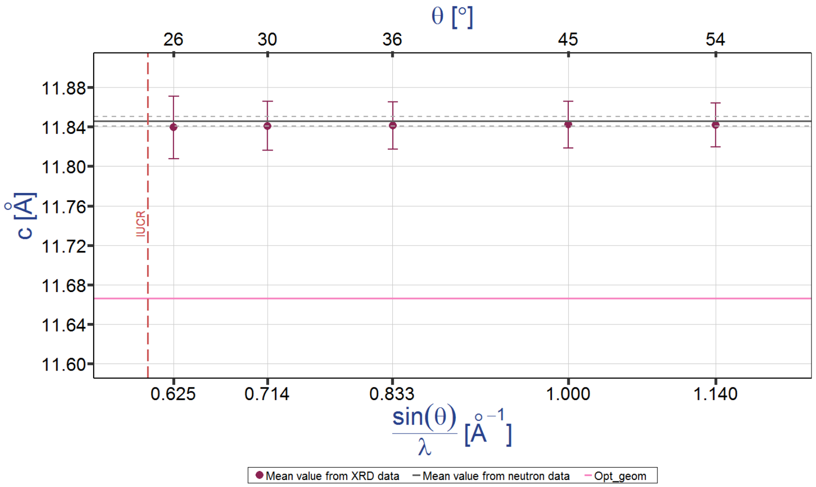
θ [°]
26
30
36
45
54
11.88
11.84
11.80
11.76
11.72
11.68
11.64
11.60
c [Å]
IUCR
0.625
0.714
0.833
1.000
1.140
sin(θ)/λ [Å^-1]
Mean value from XRD data
Mean value from neutron data
Opt_geom

(c)

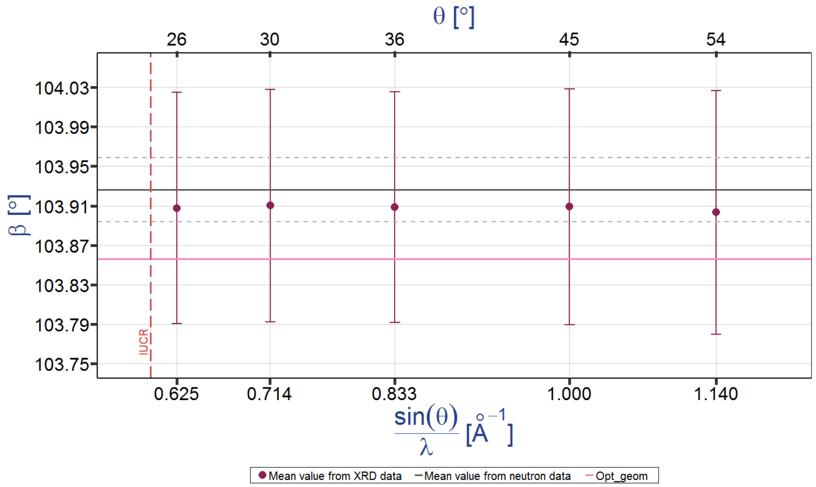
θ [°]
26
30
36
45
54
104.03
103.99
103.95
103.91
103.87
103.83
103.79
103.75
β [°]
IUCR
0.625
0.714
0.833
1.000
1.140
sin(θ)/λ [Å^-1]
Mean value from XRD data
Mean value from neutron data
Opt_geom

(d)

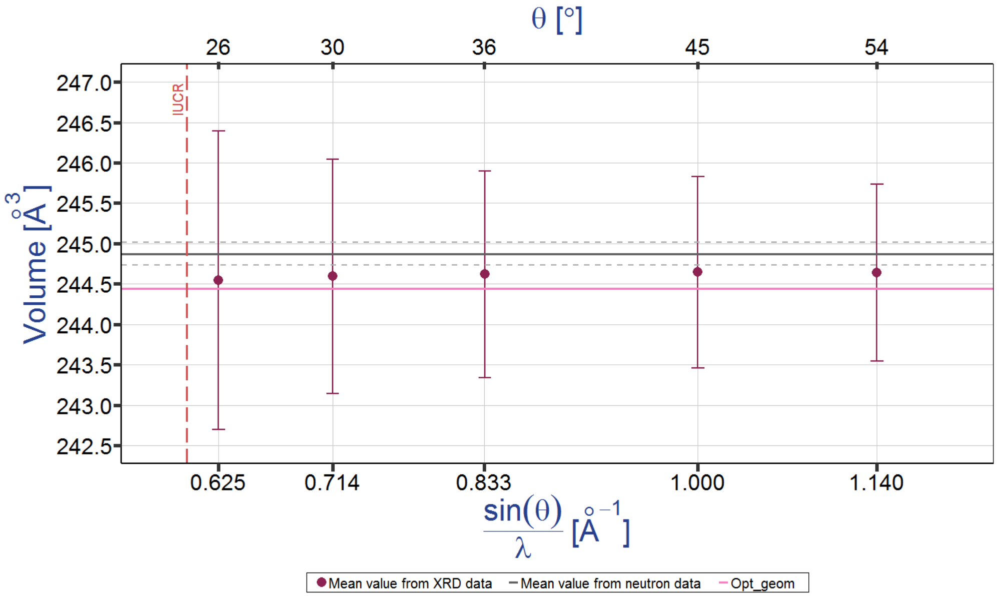

(e)

Figure 6S. Dependencies of the unit cell parameters on data resolution and the electron density model refined against X-ray and neutron data for: **(a)** a, **(b)** b, **(c)** c, **(d)** b, **(e)** unit cell vol.

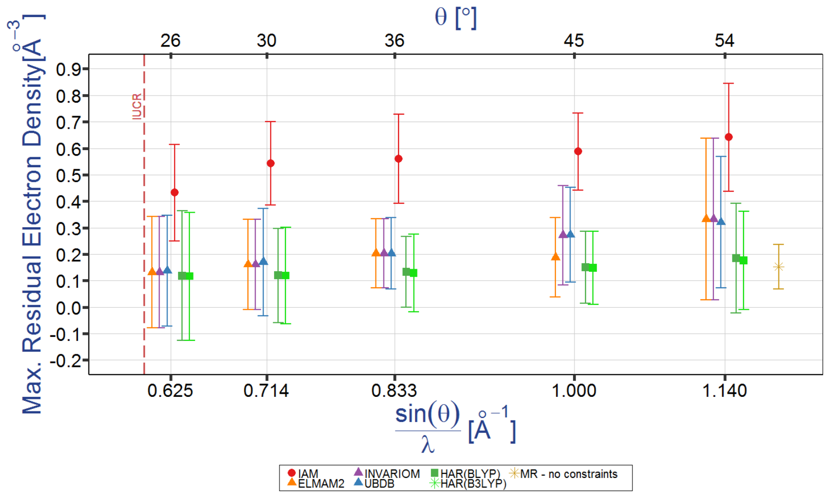

(a)

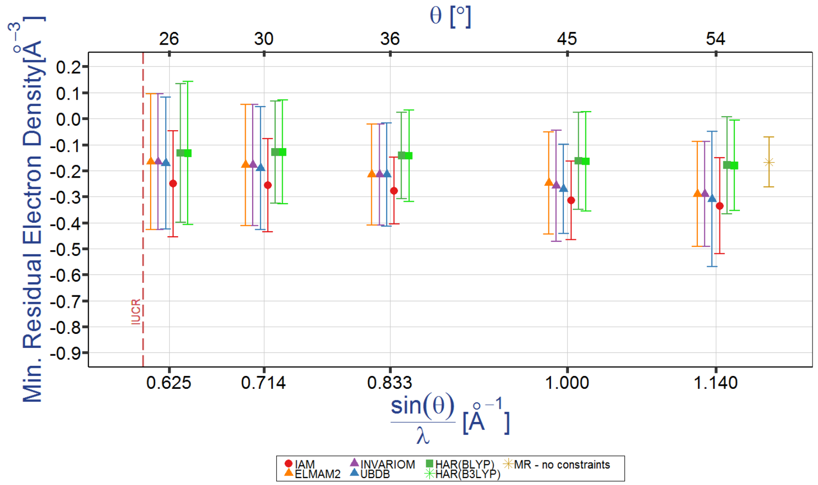

(b)

Figure 7S. Dependencies of residual electron density on data resolution and the electron density model refined against X-ray and neutron data for: **(a)** Max. residual electron density, **(b)** Min. residual electron density.

Table 1S. Numerical values for all parameters studied.

Method	Resolution [Å $^{-1}$]	nr set	C1-C1	C1-O1	C1-O2	O1-H1	O3-H2	O3-H3
IAM	0.625	1	1.55095	1.28769	1.21813	0.9613	0.88555	0.86789
		2	1.54687	1.28667	1.21891	0.9415	0.87327	0.82978
		3	1.55044	1.28603	1.2179	0.90644	0.8693	0.8484
		4	1.54757	1.28603	1.21677	0.94921	0.89161	0.84659
		5	1.5474	1.28844	1.21966	0.95742	0.88446	0.85728
		6	1.54779	1.28807	1.21739	0.92777	0.87637	0.86983
		7	1.55287	1.2894	1.21398	0.94372	0.88115	0.85509
		8	1.55036	1.28684	1.21763	0.95377	0.89417	0.83898
		9	1.55151	1.28859	1.21774	0.95985	0.88375	0.84584
		10	1.54836	1.28773	1.21846	0.93303	0.87251	0.84845
		11	1.54546	1.28889	1.22156	0.97335	0.89468	0.83606
		12	1.55147	1.29106	1.22006	0.89237	0.85646	0.83162
		13	1.54909	1.29163	1.22087	0.95734	0.87059	0.84393
	0.71429	1	1.54805	1.28971	1.22009	0.96392	0.89165	0.86327
		2	1.54389	1.29007	1.22044	0.93128	0.84557	0.83358
		3	1.54722	1.28956	1.21965	0.90905	0.86086	0.842
		4	1.5444	1.28959	1.21953	0.95507	0.88321	0.84333
		5	1.54525	1.29097	1.22119	0.95625	0.87474	0.84655
		6	1.54505	1.29063	1.2192	0.93714	0.8752	0.85842
		7	1.54953	1.29165	1.21795	0.94165	0.8672	0.85137
		8	1.54804	1.29008	1.21923	0.95838	0.88596	0.83371
		9	1.54785	1.29188	1.21938	0.95804	0.86645	0.83987
		10	1.54533	1.29043	1.22027	0.93987	0.86532	0.84615
		11	1.54757	1.29119	1.21983	0.96491	0.86355	0.85267
		12	1.54794	1.29258	1.2229	0.88129	0.86249	0.81452
		13	1.54496	1.2924	1.22394	0.96145	0.87242	0.84135
	0.83333	1	1.54585	1.28932	1.22226	0.97528	0.89192	0.85944
		2	1.54191	1.2891	1.22222	0.94159	0.86718	0.83526
		3	1.54434	1.28851	1.22263	0.92091	0.86726	0.84487
		4	1.54199	1.28864	1.22219	0.9628	0.87965	0.84534
		5	1.54298	1.28996	1.22375	0.96112	0.87527	0.85159

		6	1.54254	1.28965	1.22229	0.95584	0.87885	0.86038
		7	1.54696	1.29049	1.22112	0.94436	0.86966	0.8556
		8	1.54381	1.28851	1.22175	0.95927	0.8891	0.83337
		9	1.54468	1.29061	1.22268	0.9681	0.87014	0.8382
		10	1.54353	1.29012	1.22233	0.94692	0.86732	0.8489
		11	1.54465	1.29024	1.22282	0.97974	0.86585	0.86286
		12	1.54453	1.29116	1.22485	0.89143	0.86241	0.82459
		13	1.54292	1.29114	1.22594	0.96603	0.87536	0.83789
	1	1	1.54524	1.2888	1.22235	0.9805	0.89556	0.86115
		2	1.54214	1.28856	1.22228	0.93895	0.86738	0.83428
		3	1.5436	1.28817	1.22251	0.91632	0.86864	0.84319
		4	1.54213	1.28819	1.22226	0.96441	0.87886	0.84643
		5	1.54217	1.28912	1.2239	0.96484	0.87995	0.84617
		6	1.54251	1.28915	1.22231	0.95369	0.8772	0.85948
		7	1.54591	1.28966	1.22134	0.94706	0.87164	0.85734
		8	1.54345	1.28805	1.22181	0.96526	0.89298	0.833
		9	1.54422	1.28954	1.2227	0.97176	0.87367	0.84305
		10	1.54343	1.28959	1.22289	0.94755	0.86805	0.84712
		11	1.54383	1.28962	1.22282	0.981	0.86787	0.86413
		12	1.54354	1.29033	1.22418	0.89968	0.86475	0.82414
		13	1.54336	1.29039	1.22514	0.96564	0.87491	0.83439
	1.14	1	1.5453	1.28853	1.22214	0.9775	0.89881	0.85632
		2	1.54185	1.28845	1.22213	0.93638	0.8657	0.82105
		3	1.54386	1.28772	1.2221	0.92842	0.88366	0.84312
		4	1.54256	1.28761	1.22224	0.95635	0.87374	0.84021
		5	1.54239	1.28866	1.22345	0.98342	0.88833	0.85351
		6	1.54258	1.2889	1.22229	0.96363	0.88294	0.85257
		7	1.5454	1.28905	1.22153	0.9871	0.88659	0.83558
		8	1.54345	1.28769	1.22152	0.96395	0.89195	0.83436
		9	1.54392	1.28912	1.22264	0.97232	0.87708	0.84521
		10	1.54518	1.28941	1.22167	0.9432	0.87088	0.85144
		11	1.54387	1.28947	1.2227	0.9751	0.87294	0.86615
		12	1.5436	1.28978	1.22359	0.88478	0.85973	0.82554
		13	1.54339	1.28967	1.22477	0.96406	0.87489	0.8337
UBDB	0.625	1	1.54504	1.28351	1.22042	1.04518	0.94605	0.94713

	2	1.54687	1.28279	1.21967	1.02776	0.9543	0.91579
	3	1.54371	1.28328	1.21933	1.03495	0.94227	0.93794
	4	1.54312	1.28272	1.21839	1.03868	0.95328	0.93607
	5	1.54298	1.28513	1.22056	1.04423	0.9401	0.94171
	6	1.54258	1.28401	1.21972	1.04466	0.93851	0.94521
	7	1.54513	1.28431	1.21936	1.03737	0.94183	0.94609
	8	1.54477	1.2831	1.21989	1.06228	0.95039	0.94735
	9	1.54525	1.28465	1.22092	1.05397	0.94814	0.94398
	10	1.54484	1.28395	1.22046	1.03457	0.93781	0.94144
	11	1.54534	1.28627	1.21932	1.0241	0.92355	0.95149
	12	1.54532	1.28729	1.22037	1.02538	0.94667	0.9234
	13	1.54545	1.2869	1.22224	1.05559	0.96051	0.93453
0.71429	1	1.54444	1.28506	1.22058	1.03021	0.94149	0.94881
	2	1.54389	1.28458	1.21973	1.0131	0.95487	0.91891
	3	1.54403	1.28575	1.21867	1.00051	0.92797	0.93819
	4	1.54267	1.28494	1.2192	1.02538	0.94395	0.93474
	5	1.54204	1.28635	1.22127	1.02681	0.93315	0.93898
	6	1.54304	1.2859	1.21902	1.01433	0.92823	0.94388
	7	1.54496	1.28571	1.21995	1.02088	0.93676	0.94589
	8	1.54431	1.2851	1.22063	1.05449	0.95388	0.94428
	9	1.5444	1.28675	1.22063	1.03667	0.9402	0.93795
	10	1.54369	1.28545	1.22091	1.02686	0.9342	0.93709
	11	1.54439	1.28678	1.21975	1.00593	0.92223	0.95011
	12	1.54483	1.28865	1.22028	1.00063	0.93249	0.92863
	13	1.54405	1.28735	1.22249	1.03517	0.94955	0.93521
0.83333	1	1.54436	1.28568	1.22085	1.01728	0.93484	0.94703
	2	1.54191	1.28535	1.22002	1.01179	0.94922	0.91827
	3	1.54201	1.28587	1.22114	1.00122	0.9306	0.9333
	4	1.54181	1.28545	1.22027	1.0226	0.93683	0.93281
	5	1.54201	1.28705	1.22136	1.01581	0.92481	0.93609
	6	1.54154	1.28626	1.22083	1.01617	0.924	0.93775
	7	1.54547	1.28663	1.22006	1.00965	0.92984	0.9458
	8	1.5428	1.28517	1.22067	1.03917	0.95008	0.94139
	9	1.54392	1.28781	1.22088	1.0296	0.93297	0.92992
	10	1.54372	1.28604	1.22113	1.022	0.9276	0.93567

		11	1.54302	1.28722	1.22093	1.00054	0.91518	0.94292
		12	1.54281	1.28837	1.22202	1.00057	0.93538	0.93174
		13	1.54379	1.28755	1.22301	1.02718	0.94674	0.93128
	1	1	1.54443	1.28595	1.22104	1.00879	0.93024	0.94808
		2	1.54214	1.2856	1.2204	1.0056	0.94418	0.916
		3	1.54232	1.28623	1.22126	0.99387	0.92449	0.93353
		4	1.54202	1.28557	1.22073	1.0185	0.92987	0.93288
		5	1.54214	1.28715	1.22164	1.01417	0.91996	0.93433
		6	1.54203	1.28655	1.22117	1.00757	0.91451	0.93538
		7	1.54504	1.28681	1.22043	1.00198	0.92376	0.94849
		8	1.54271	1.28559	1.22086	1.03795	0.94613	0.94208
		9	1.54377	1.28767	1.22147	1.03101	0.93348	0.9322
		10	1.54376	1.28612	1.22144	1.01124	0.92293	0.93244
		11	1.54332	1.28736	1.22109	0.98616	0.90651	0.94457
		12	1.543	1.28834	1.22192	0.98677	0.93434	0.93837
		13	1.54416	1.28772	1.22292	1.02367	0.94341	0.92893
	1.14	1	1.54453	1.28607	1.22112	0.99821	0.9254	0.94458
		2	1.54228	1.28557	1.22038	0.99877	0.94152	0.90287
		3	1.54271	1.28593	1.22109	0.99405	0.93661	0.93432
		4	1.5423	1.28524	1.22089	1.01855	0.92686	0.92564
		5	1.54236	1.28697	1.22175	0.98429	0.93046	0.93017
		6	1.54216	1.28651	1.22135	0.99755	0.91504	0.93462
		7	1.54502	1.28674	1.22064	1.02362	0.91903	0.93908
		8	1.54273	1.28547	1.22084	1.03178	0.94714	0.94153
		9	1.54381	1.28754	1.22156	1.01992	0.93123	0.93284
		10	1.54475	1.28615	1.22046	1.00875	0.92364	0.93905
		11	1.54351	1.2873	1.22101	0.97592	0.90515	0.95095
		12	1.54311	1.28822	1.22182	0.97671	0.93632	0.94136
		13	1.54404	1.28722	1.22284	1.02036	0.94251	0.92701
ELMAM2	0.625	1	1.54414	1.28331	1.222	1.05354	0.95813	0.94433
		2	1.54389	1.28214	1.2215	1.03441	0.94761	0.92024
		3	1.54294	1.28335	1.22106	1.0504	0.9568	0.93564
		4	1.54191	1.28229	1.22035	1.05193	0.955	0.92796
		5	1.54224	1.2848	1.22243	1.04971	0.95163	0.93885
		6	1.54208	1.28382	1.22129	1.04686	0.95107	0.94451

	7	1.54423	1.28342	1.2216	1.04823	0.94707	0.94093
	8	1.54373	1.28199	1.22203	1.06177	0.95095	0.93737
	9	1.54379	1.28393	1.22308	1.06454	0.94977	0.93409
	10	1.54383	1.28348	1.22231	1.04344	0.9436	0.93723
	11	1.54425	1.28619	1.22103	1.03868	0.94702	0.94445
	12	1.54471	1.28752	1.2221	1.04351	0.95563	0.92957
	13	1.54495	1.28645	1.22401	1.0615	0.9636	0.9236
0.71429	1	1.54411	1.28478	1.22193	1.04227	0.95774	0.94895
	2	1.54191	1.28419	1.22182	1.02058	0.93719	0.92855
	3	1.54384	1.2855	1.22029	1.02229	0.94414	0.94013
	4	1.54218	1.2844	1.22085	1.04102	0.94784	0.93222
	5	1.54289	1.2862	1.22178	1.03064	0.94476	0.94309
	6	1.54305	1.28553	1.22047	1.01905	0.94579	0.94667
	7	1.545	1.28502	1.22138	1.03191	0.94275	0.94631
	8	1.54364	1.28409	1.22262	1.05661	0.95432	0.93945
	9	1.54379	1.28602	1.22249	1.05056	0.94429	0.93548
	10	1.54322	1.28492	1.22252	1.0342	0.94339	0.93496
	11	1.54394	1.28662	1.22117	1.02802	0.9463	0.94858
	12	1.54507	1.28849	1.22155	1.031	0.94592	0.93986
	13	1.54427	1.28685	1.22404	1.04302	0.95408	0.92989
0.83333	1	1.54434	1.2857	1.2217	1.02941	0.95216	0.94776
	2	1.54214	1.28522	1.22177	1.01659	0.93642	0.92482
	3	1.54325	1.28637	1.22071	1.01211	0.9437	0.935
	4	1.54194	1.28534	1.22102	1.0313	0.93985	0.93134
	5	1.54219	1.28695	1.22228	1.03256	0.94085	0.9416
	6	1.54176	1.28622	1.22169	1.01792	0.94372	0.94371
	7	1.54543	1.28609	1.22132	1.02872	0.93959	0.94787
	8	1.54249	1.28466	1.22201	1.04176	0.94987	0.94015
	9	1.54367	1.28757	1.22211	1.04185	0.9389	0.93123
	10	1.54341	1.2858	1.22234	1.02767	0.93837	0.93336
	11	1.54291	1.28728	1.22183	1.02345	0.94177	0.94359
	12	1.54307	1.28837	1.22292	1.03985	0.94499	0.93869
	13	1.54411	1.28738	1.22394	1.03407	0.95071	0.92769
1	1	1.54362	1.28846	1.22262	1.07794	0.96823	0.96671
	2	1.54195	1.2883	1.22206	1.06311	0.96668	0.96437

		3	1.54259	1.28827	1.22186	1.05589	0.96292	0.95985
		4	1.54178	1.28791	1.22174	1.07596	0.96582	0.96349
		5	1.54178	1.2888	1.22315	1.06896	0.97107	0.965
		6	1.54212	1.28858	1.22197	1.05728	0.95906	0.96378
		7	1.54411	1.28896	1.2219	1.06031	0.95706	0.9663
		8	1.54189	1.28824	1.22211	1.07981	0.97182	0.96059
		9	1.54306	1.28945	1.2228	1.07525	0.95812	0.9627
		10	1.54272	1.28897	1.22302	1.07485	0.96986	0.96533
		11	1.54297	1.28911	1.22261	1.06476	0.96529	0.96932
		12	1.54292	1.2898	1.22303	1.05376	0.97251	0.96845
		13	1.54382	1.28976	1.2242	1.07734	0.96768	0.95431
	1.14	1	1.54456	1.28592	1.22201	1.00883	0.94658	0.94677
		2	1.5423	1.28535	1.22183	1.00303	0.93523	0.91081
		3	1.54265	1.28597	1.2223	1.01591	0.93464	0.93557
		4	1.54215	1.28503	1.22189	1.0228	0.92929	0.92818
		5	1.54238	1.2868	1.22285	1.0001	0.93869	0.93136
		6	1.54221	1.28629	1.22227	0.99789	0.93374	0.94119
		7	1.54495	1.28636	1.22178	1.0394	0.93704	0.93981
		8	1.54244	1.28506	1.22201	1.03639	0.94618	0.94363
		9	1.54367	1.28742	1.22246	1.02604	0.93652	0.93292
		10	1.54456	1.28582	1.22171	1.01639	0.93273	0.94179
		11	1.54339	1.28718	1.22202	0.99643	0.93188	0.95223
		12	1.54332	1.28816	1.22256	1.0133	0.93795	0.94342
		13	1.54409	1.28697	1.22384	1.02617	0.94723	0.92511
GID	0.625	1	1.54414	1.28331	1.222	1.05354	0.95813	0.94433
		2	1.54687	1.28214	1.2215	1.03441	0.94761	0.92024
		3	1.54294	1.28335	1.22106	1.0504	0.9568	0.93564
		4	1.54191	1.28229	1.22035	1.05193	0.955	0.92796
		5	1.54224	1.2848	1.22243	1.04971	0.95163	0.93885
		6	1.54208	1.28382	1.22129	1.04686	0.95107	0.94451
		7	1.54423	1.28342	1.2216	1.04823	0.94707	0.94093
		8	1.54373	1.28199	1.22203	1.06177	0.95095	0.93737
		9	1.54379	1.28393	1.22308	1.06454	0.94977	0.93409
		10	1.54383	1.28348	1.22231	1.04344	0.9436	0.93723
		11	1.54425	1.28619	1.22103	1.03868	0.94702	0.94445

		12	1.54471	1.28752	1.2221	1.04351	0.95563	0.92957
		13	1.54495	1.28645	1.22401	1.0615	0.9636	0.9236
	0.71429	1	1.54411	1.28478	1.22193	1.04227	0.95774	0.94895
		2	1.54389	1.28419	1.22182	1.02058	0.93719	0.92855
		3	1.54384	1.2855	1.22029	1.02229	0.94414	0.94013
		4	1.54218	1.2844	1.22085	1.04102	0.94784	0.93222
		5	1.54289	1.2862	1.22178	1.03064	0.94476	0.94309
		6	1.54305	1.28553	1.22047	1.01905	0.94579	0.94667
		7	1.545	1.28502	1.22138	1.03191	0.94275	0.94631
		8	1.54364	1.28409	1.22262	1.05661	0.95432	0.93945
		9	1.54379	1.28602	1.22249	1.05056	0.94429	0.93548
		10	1.54322	1.28492	1.22252	1.0342	0.94339	0.93496
		11	1.54394	1.28662	1.22117	1.02802	0.9463	0.94858
		12	1.54507	1.28849	1.22155	1.031	0.94592	0.93986
		13	1.54427	1.28685	1.22404	1.04302	0.95408	0.92989
	0.83333	1	1.54434	1.2857	1.2217	1.02941	0.95216	0.94776
		2	1.54191	1.28522	1.22177	1.01659	0.93642	0.92482
		3	1.54325	1.28637	1.22071	1.01211	0.9437	0.935
		4	1.54194	1.28534	1.22102	1.0313	0.93985	0.93134
		5	1.54219	1.28695	1.22228	1.03256	0.94085	0.9416
		6	1.54176	1.28622	1.22169	1.01792	0.94372	0.94371
		7	1.54543	1.28609	1.22132	1.02872	0.93959	0.94787
		8	1.54249	1.28466	1.22201	1.04176	0.94987	0.94015
		9	1.54367	1.28757	1.22211	1.04185	0.9389	0.93123
		10	1.54341	1.2858	1.22234	1.02767	0.93837	0.93336
		11	1.54291	1.28728	1.22183	1.02345	0.94177	0.94359
		12	1.54307	1.28837	1.22292	1.03985	0.94499	0.93869
		13	1.54411	1.28738	1.22394	1.03407	0.95071	0.92769
	1	1	1.54441	1.28587	1.22197	1.02107	0.95065	0.94901
		2	1.54214	1.28536	1.22203	1.01004	0.93404	0.92298
		3	1.54228	1.2862	1.22228	1.01941	0.94212	0.93781
		4	1.5419	1.28527	1.22184	1.02768	0.93324	0.93291
		5	1.54218	1.28697	1.22267	1.03058	0.93673	0.93988
		6	1.54204	1.28634	1.22222	1.00773	0.93671	0.94175
		7	1.54492	1.28623	1.22182	1.02097	0.93656	0.94835

		8	1.54241	1.28512	1.22217	1.04038	0.9478	0.94206
		9	1.54344	1.2874	1.2228	1.04348	0.93847	0.93291
		10	1.54346	1.28583	1.22271	1.01755	0.93488	0.93019
		11	1.5432	1.28724	1.22213	1.01303	0.93438	0.94273
		12	1.54311	1.2882	1.22294	1.03225	0.93927	0.94028
		13	1.54428	1.28745	1.22398	1.03014	0.94648	0.92658
	1.14	1	1.54456	1.28592	1.22201	1.00883	0.94658	0.94677
		2	1.5423	1.28535	1.22183	1.00303	0.93523	0.91081
		3	1.54265	1.28597	1.2223	1.01591	0.93464	0.93557
		4	1.54215	1.28503	1.22189	1.0228	0.92929	0.92818
		5	1.54238	1.2868	1.22285	1.0001	0.93869	0.93136
		6	1.54221	1.28629	1.22227	0.99789	0.93374	0.94119
		7	1.54495	1.28636	1.22178	1.0394	0.93704	0.93981
		8	1.54244	1.28506	1.22201	1.03639	0.94618	0.94363
		9	1.54367	1.28742	1.22246	1.02604	0.93652	0.93292
		10	1.54456	1.28582	1.22171	1.01639	0.93273	0.94179
		11	1.54339	1.28718	1.22202	0.99643	0.93188	0.95223
		12	1.54332	1.28816	1.22256	1.0133	0.93795	0.94342
		13	1.54409	1.28697	1.22384	1.02617	0.94723	0.92511
HAR(B3LYP)	0.625	1	1.544506	1.285922	1.220121	1.055	0.963	0.952
		2	1.543058	1.284859	1.220025	1.04	0.958	0.947
		3	1.543666	1.285075	1.219717	1.049	0.962	0.951
		4	1.542239	1.284506	1.218824	1.049	0.965	0.95
		5	1.542956	1.28681	1.22065	1.054	0.958	0.953
		6	1.545637	1.288241	1.222538	1.061	0.969	0.946
		7	1.54437	1.286124	1.219856	1.05	0.961	0.947
		8	1.544448	1.286122	1.219812	1.05	0.96	0.95
		9	1.54352	1.285438	1.220287	1.054	0.96	0.944
		10	1.544604	1.28679	1.221146	1.04	0.96	0.941
		11	1.543407	1.285622	1.221056	1.041	0.955	0.957
		12	1.545022	1.288463	1.219227	1.06	0.94	0.97
		13	1.545307	1.288077	1.221237	1.04	0.977	0.955
	0.71429	1	1.544457	1.286407	1.220488	1.058	0.969	0.956
		2	1.542799	1.285856	1.220571	1.04	0.948	0.951
		3	1.542784	1.285789	1.221143	1.05	0.963	0.95

	4	1.542208	1.285678	1.220064	1.058	0.961	0.953
	5	1.542665	1.286945	1.221402	1.06	0.962	0.954
	6	1.544803	1.287833	1.222646	1.061	0.968	0.956
	7	1.544814	1.28646	1.220524	1.055	0.964	0.954
	8	1.544748	1.286547	1.220437	1.05	0.96	0.958
	9	1.543681	1.286527	1.22106	1.061	0.964	0.947
	10	1.543591	1.287757	1.221454	1.054	0.961	0.949
	11	1.542844	1.286322	1.221517	1.053	0.958	0.958
	12	1.545459	1.28844	1.218994	1.06	0.945	0.98
	13	1.544238	1.288107	1.222317	1.05	0.983	0.96
0.83333	1	1.544318	1.286725	1.220677	1.058	0.969	0.958
	2	1.542679	1.286271	1.220716	1.05	0.949	0.951
	3	1.542424	1.286429	1.221142	1.05	0.964	0.954
	4	1.541908	1.286023	1.220468	1.06	0.961	0.955
	5	1.542583	1.287363	1.221369	1.061	0.964	0.958
	6	1.544299	1.287984	1.222878	1.061	0.966	0.955
	7	1.545238	1.287093	1.220385	1.052	0.964	0.959
	8	1.545253	1.28717	1.220273	1.05	0.962	0.966
	9	1.542526	1.286175	1.220863	1.058	0.964	0.948
	10	1.543441	1.288325	1.221291	1.054	0.959	0.952
	11	1.543081	1.286726	1.22161	1.057	0.957	0.959
	12	1.543377	1.287779	1.220816	1.061	0.97	0.962
	13	1.543467	1.288406	1.222036	1.06	0.978	0.969
1	1	1.544294	1.286836	1.22092	1.058	0.968	0.958
	2	1.542568	1.286297	1.220959	1.045	0.948	0.949
	3	1.542526	1.286668	1.221344	1.055	0.963	0.953
	4	1.542096	1.286096	1.220943	1.06	0.956	0.955
	5	1.542491	1.28744	1.22166	1.065	0.963	0.958
	6	1.544426	1.288146	1.222894	1.062	0.965	0.953
	7	1.544939	1.287205	1.220672	1.05	0.962	0.958
	8	1.544948	1.287268	1.220594	1.049	0.961	0.966
	9	1.542502	1.286391	1.221035	1.059	0.962	0.949
	10	1.543593	1.288073	1.221647	1.057	0.96	0.952
	11	1.54318	1.28675	1.221922	1.055	0.956	0.956
	12	1.543505	1.287852	1.221079	1.06	0.969	0.964

		13	1.543347	1.288425	1.222004	1.056	0.979	0.97
	1.14	1	1.544398	1.286907	1.221095	1.057	0.967	0.954
		2	1.543926	1.28698	1.220875	1.043	0.95	0.936
		3	1.542749	1.286541	1.221525	1.053	0.951	0.946
		4	1.542257	1.28592	1.221065	1.057	0.951	0.951
		5	1.542479	1.287436	1.222031	1.065	0.955	0.954
		6	1.544222	1.287717	1.222822	1.061	0.965	0.953
		7	1.542219	1.287036	1.221353	1.05	0.962	0.956
		8	1.544799	1.28724	1.220733	1.059	0.965	0.954
		9	1.542608	1.286276	1.220968	1.059	0.965	0.946
		10	1.543553	1.288052	1.221757	1.05	0.958	0.952
		11	1.544465	1.286638	1.220928	1.055	0.952	0.964
		12	1.54362	1.28782	1.221048	1.053	0.968	0.968
		13	1.543304	1.28846	1.22201	1.043	0.977	0.97
HAR(BLYP)	0.625	1	1.54496	1.285841	1.220142	1.046	0.961	0.95
		2	1.543408	1.284799	1.220118	1.03	0.958	0.941
		3	1.544175	1.285017	1.219729	1.04	0.961	0.947
		4	1.542673	1.28445	1.21887	1.04	0.964	0.944
		5	1.543308	1.286788	1.220734	1.046	0.957	0.95
		6	1.542701	1.285929	1.219668	1.04	0.955	0.953
		7	1.544971	1.286051	1.219754	1.04	0.959	0.944
		8	1.544063	1.285361	1.220194	1.04	0.959	0.939
		9	1.544604	1.28679	1.221146	1.04	0.96	0.941
		10	1.543866	1.2856	1.221048	1.028	0.954	0.952
		11	1.545435	1.288427	1.21926	1.05	0.94	0.97
		12	1.545727	1.288117	1.221327	1.04	0.975	0.95
		13	1.546038	1.288252	1.222498	1.051	0.969	0.942
	0.71429	1	1.544636	1.286475	1.220665	1.051	0.969	0.953
		2	1.542962	1.285976	1.220703	1.03	0.947	0.947
		3	1.542992	1.2859	1.221304	1.05	0.962	0.947
		4	1.542403	1.285822	1.22017	1.051	0.96	0.95
		5	1.542812	1.287073	1.221578	1.054	0.962	0.952
		6	1.543322	1.286807	1.219381	1.04	0.959	0.957
		7	1.545107	1.286604	1.220576	1.046	0.963	0.952
		8	1.543989	1.286619	1.221077	1.052	0.964	0.942

		9	1.543921	1.287868	1.221373	1.045	0.96	0.946
		10	1.543114	1.286445	1.221604	1.044	0.957	0.953
		11	1.545607	1.288535	1.21915	1.05	0.945	0.98
		12	1.544415	1.288289	1.222533	1.05	0.982	0.955
		13	1.544935	1.287987	1.222788	1.053	0.967	0.953
	0.83333	1	1.544375	1.286813	1.2209	1.052	0.969	0.956
		2	1.542697	1.286407	1.220896	1.04	0.949	0.947
		3	1.542494	1.286535	1.221342	1.05	0.964	0.951
		4	1.541943	1.286158	1.220632	1.054	0.96	0.953
		5	1.542619	1.287491	1.221586	1.056	0.964	0.956
		6	1.541875	1.286976	1.220894	1.045	0.963	0.956
		7	1.545358	1.287229	1.220522	1.045	0.962	0.956
		8	1.542647	1.286293	1.220958	1.049	0.964	0.944
		9	1.543539	1.288449	1.221369	1.047	0.959	0.948
		10	1.543169	1.286854	1.22179	1.049	0.956	0.954
		11	1.543388	1.287894	1.22106	1.059	0.97	0.959
		12	1.543525	1.288545	1.222254	1.05	0.976	0.964
		13	1.544302	1.288132	1.223056	1.054	0.966	0.953
	1	1	1.544311	1.286901	1.221094	1.054	0.968	0.955
		2	1.54259	1.286393	1.221107	1.04	0.948	0.945
		3	1.542548	1.286744	1.221485	1.049	0.963	0.95
		4	1.54211	1.286202	1.221065	1.054	0.955	0.952
		5	1.542489	1.287537	1.221808	1.059	0.962	0.956
		6	1.542198	1.287095	1.221277	1.047	0.961	0.955
		7	1.544995	1.287298	1.220776	1.044	0.961	0.956
		8	1.542586	1.286465	1.221105	1.051	0.962	0.945
		9	1.543635	1.288155	1.221715	1.049	0.96	0.95
		10	1.543255	1.286855	1.222067	1.046	0.955	0.953
		11	1.543484	1.287933	1.221241	1.059	0.969	0.961
		12	1.543351	1.288519	1.222153	1.05	0.977	0.965
		13	1.544404	1.288255	1.22302	1.055	0.964	0.95
	1.14	1	1.54441	1.286962	1.221238	1.052	0.967	0.951
		2	1.543942	1.287081	1.221006	1.036	0.95	0.932
		3	1.542772	1.286597	1.221647	1.047	0.951	0.943
		4	1.542284	1.286007	1.221172	1.05	0.951	0.948

		5	1.542484	1.28749	1.222145	1.062	0.955	0.953
		6	1.542203	1.287118	1.221469	1.047	0.962	0.953
		7	1.544824	1.287326	1.220822	1.055	0.966	0.949
		8	1.542695	1.286336	1.221011	1.049	0.964	0.943
		9	1.543583	1.288118	1.2218	1.042	0.958	0.949
		10	1.54454	1.286743	1.221072	1.048	0.951	0.96
		11	1.543589	1.287892	1.221215	1.052	0.968	0.964
		12	1.543323	1.288528	1.222108	1.036	0.974	0.964
		13	1.544206	1.287815	1.222932	1.054	0.965	0.951
MR_no	1.19	1	1.54399	1.28679	1.2219	1.01867	0.90862	0.95749
		2	1.54313	1.28711	1.22115	1.00915	0.89319	1.01229
		3	1.54203	1.28649	1.22196	1.08211	0.89227	0.97483
		4	1.54161	1.28557	1.22174	1.02668	0.89849	1.03589
		5	1.542	1.28722	1.22251	1.04069	0.88471	0.86275
		6	1.54171	1.28688	1.22212	1.03382	0.94199	0.98752
		7	1.5439	1.28714	1.22181	0.82185	0.88318	0.95813
		8	1.5418	1.28655	1.2214	1.00715	0.99896	0.99401
		9	1.54314	1.28809	1.22187	1.00818	0.90879	0.97345
		10	1.54288	1.28785	1.22401	1.02225	0.98977	0.95739
		11	1.54304	1.2878	1.22218	0.96773	0.9312	0.97066
		12	1.54296	1.28845	1.22237	1.0045	0.91266	0.99478
		13	1.54354	1.28761	1.22345	0.98097	0.85588	0.96469
MR	1.18	1	1.54396	1.286809	1.221977	1.073855	0.969973	0.973686
		2	1.543037	1.287116	1.221236	1.073941	0.970076	0.973903
		3	1.542023	1.286559	1.222008	1.074016	0.970031	0.973592
		4	1.541563	1.285563	1.221855	1.07393	0.970097	0.97387
		5	1.542	1.287267	1.222591	1.073945	0.969839	0.973554
		6	1.541691	1.286892	1.222189	1.073882	0.970021	0.973828
		7	1.543792	1.287204	1.221907	1.073591	0.970047	0.973678
		8	1.541736	1.286528	1.221488	1.07392	0.970035	0.974035
		9	1.543118	1.288134	1.221933	1.073924	0.97001	0.973897
		10	1.542836	1.287862	1.22411	1.073949	0.96999	0.974027
		11	1.542965	1.287826	1.222276	1.073548	0.970029	0.973651
		12	1.54293	1.288467	1.22242	1.073796	0.970127	0.973799
		13	1.543517	1.287629	1.223552	1.073804	0.970006	0.973793

Neutron Diffraction	N/A	1	1.549	1.287	1.219	1.077	0.968	0.965
		2	1.55	1.286	1.219	1.079	0.971	0.965
		3	1.5469	1.287	1.22	1.074	0.966	0.965
		4	1.5491	1.287	1.219	1.079	0.968	0.968
		5	1.5447	1.286	1.222	1.076	0.967	0.967
		6	1.55	1.286	1.22	1.08	0.967	0.964
		7	1.549	1.287	1.22	1.078	0.964	0.969
		8	1.55	1.287	1.22	1.078	0.969	0.966
		9	1.5493	1.285	1.223	1.08	0.967	0.966
		10	1.546	1.286	1.22	1.078	0.965	0.964
		11	1.549	1.286	1.22	1.078	0.971	0.964
		12	1.549	1.289	1.219	1.076	0.968	0.968
		13	1.549	1.286	1.219	1.081	0.97	0.968
		14	1.5506	1.285	1.219	1.08	0.965	0.969

Method	Resolution [Å $^{-1}$]	nr set	H1...O3	H2...O2	H3...O2	O1...O3	O3...O2	(ii) x, 1.5-y, 1/2+ O3...O2 ii
IAM	0.625	1	1.5258	1.94869	2.03121	2.48691	2.82568	2.83005
		2	1.54319	1.96192	2.05225	2.48401	2.82336	2.8284
		3	1.57771	1.96625	2.04242	2.48406	2.82418	2.82853
		4	1.53211	1.9401	2.03721	2.4807	2.82074	2.82432
		5	1.52676	1.95276	2.03659	2.48414	2.82826	2.8299
		6	1.55544	1.95918	2.02488	2.48271	2.82556	2.82726
		7	1.54453	1.9536	2.04339	2.48801	2.82519	2.83142
		8	1.53103	1.94132	2.04938	2.48475	2.82494	2.82937
		9	1.52689	1.95397	2.04724	2.48659	2.82793	2.8305
		10	1.55122	1.96375	2.04635	2.48408	2.82676	2.83051
		11	1.51647	1.94187	2.05213	2.48834	2.82458	2.82922
		12	1.59795	1.98601	2.05789	2.48879	2.82862	2.83192
		13	1.53127	1.96938	2.04171	2.4882	2.82918	2.83383
	0.71429	1	1.52365	1.94151	2.03227	2.48708	2.82445	2.82972
		2	1.5538	1.9906	2.04881	2.48381	2.82372	2.82799
		3	1.57577	1.97584	2.04363	2.48456	2.8235	2.82766
		4	1.52735	1.94932	2.03717	2.48151	2.82084	2.82418
		5	1.52855	1.96281	2.04257	2.48465	2.82656	2.82903
		6	1.54742	1.95979	2.03055	2.48337	2.82432	2.82655
		7	1.54732	1.96708	2.04276	2.4887	2.82441	2.83013
		8	1.52744	1.95036	2.05095	2.48546	2.82605	2.82896
		9	1.52912	1.97139	2.0486	2.48651	2.82714	2.82939
		10	1.54522	1.97012	2.04631	2.48461	2.82563	2.82908
		11	1.52286	1.97475	2.04247	2.48654	2.82635	2.82975
		12	1.60892	1.97998	2.06627	2.4876	2.82709	2.82971
		13	1.52699	1.96629	2.04163	2.4873	2.82751	2.83103
	0.83333	1	1.51342	1.94138	2.03395	2.48718	2.82382	2.82947
		2	1.54399	1.96802	2.04981	2.4843	2.82291	2.82736
		3	1.56426	1.96861	2.03983	2.48472	2.823	2.82689
		4	1.52065	1.95239	2.0359	2.48248	2.82045	2.82417
		5	1.52362	1.96126	2.03764	2.48454	2.82575	2.82765

		6	1.52928	1.95491	2.02902	2.48369	2.8238	2.82572
		7	1.54464	1.96483	2.03893	2.48893	2.82436	2.82956
		8	1.52589	1.94429	2.05065	2.48465	2.82355	2.82753
		9	1.51959	1.96744	2.04653	2.48644	2.82657	2.82852
		10	1.53847	1.96723	2.04552	2.48487	2.82522	2.82867
		11	1.50866	1.97009	2.02862	2.48588	2.82546	2.82799
		12	1.60078	1.9781	2.0523	2.4867	2.8264	2.82822
		13	1.52293	1.96285	2.04346	2.4872	2.82717	2.83036
	1	1	1.50871	1.93765	2.03247	2.48735	2.82362	2.82957
		2	1.54653	1.96821	2.05082	2.48422	2.82301	2.82746
		3	1.56935	1.96842	2.03973	2.48503	2.82326	2.82716
		4	1.51995	1.95371	2.03541	2.48317	2.82057	2.82464
		5	1.5202	1.95844	2.04189	2.48454	2.82573	2.82722
		6	1.53204	1.95728	2.02903	2.48416	2.82412	2.82625
		7	1.54193	1.96291	2.03727	2.48887	2.82457	2.8297
		8	1.51994	1.94166	2.05043	2.48465	2.82387	2.82745
		9	1.51611	1.96404	2.04314	2.48654	2.82666	2.82879
		10	1.53853	1.96671	2.04757	2.48543	2.82532	2.8284
		11	1.50741	1.96813	2.02652	2.48607	2.82558	2.82797
		12	1.5938	1.97589	2.05051	2.48638	2.82666	2.82783
		13	1.52369	1.96468	2.04676	2.48739	2.82773	2.83057
	1.14	1	1.51137	1.93505	2.03769	2.48737	2.8238	2.82944
		2	1.54967	1.97028	2.06152	2.48454	2.8231	2.82719
		3	1.55807	1.95339	2.04658	2.48547	2.82328	2.82734
		4	1.52874	1.95791	2.04251	2.48364	2.82046	2.82472
		5	1.50237	1.94876	2.03815	2.48502	2.82578	2.82759
		6	1.52241	1.95107	2.0342	2.48467	2.82411	2.82639
		7	1.50267	1.95149	2.05202	2.48834	2.82455	2.82932
		8	1.52099	1.94233	2.05246	2.48454	2.82422	2.82768
		9	1.51512	1.95997	2.04264	2.48668	2.82661	2.82885
		10	1.54468	1.96231	2.04439	2.48765	2.82393	2.82961
		11	1.5129	1.96289	2.02538	2.48629	2.82576	2.82823
		12	1.6087	1.98026	2.0526	2.4863	2.82669	2.82753
		13	1.52505	1.96375	2.04743	2.48716	2.82681	2.83021
UBDB	0.625	1	1.44711	1.88939	1.9302	2.48875	2.82089	2.82645

	2	1.46133	1.88879	1.95613	2.48583	2.82092	2.82387
	3	1.45325	1.89698	1.94097	2.48576	2.82016	2.8247
	4	1.44768	1.88154	1.93667	2.48264	2.81618	2.82047
	5	1.44443	1.89973	1.93822	2.48642	2.8241	2.82632
	6	1.44316	1.89583	1.9332	2.48466	2.82113	2.82257
	7	1.45633	1.89127	1.93207	2.48983	2.82086	2.82732
	8	1.42683	1.88528	1.93065	2.48631	2.82069	2.82543
	9	1.4374	1.89004	1.93583	2.48829	2.82362	2.82652
	10	1.45393	1.90131	1.93957	2.48651	2.82257	2.8252
	11	1.46728	1.91744	1.93821	2.48845	2.82353	2.82759
	12	1.46793	1.89852	1.9607	2.49006	2.82559	2.82938
	13	1.43778	1.88118	1.94573	2.49024	2.82529	2.83071
0.71429	1	1.46158	1.89405	1.92875	2.4883	2.82106	2.82717
	2	1.47575	1.88832	1.9608	2.48547	2.82114	2.82516
	3	1.48725	1.9104	1.94255	2.48553	2.82135	2.82608
	4	1.46078	1.89264	1.941	2.48307	2.81786	2.82192
	5	1.46084	1.90758	1.94302	2.4856	2.82415	2.8265
	6	1.47323	1.90603	1.9356	2.48454	2.82172	2.82418
	7	1.47303	1.89874	1.93364	2.48941	2.82165	2.8279
	8	1.43523	1.88317	1.93436	2.48652	2.82262	2.82632
	9	1.45384	1.89854	1.94507	2.48729	2.82431	2.82699
	10	1.46093	1.90481	1.94607	2.4861	2.82253	2.82558
	11	1.48431	1.91923	1.93408	2.48745	2.82373	2.82751
	12	1.49069	1.90923	1.95765	2.48856	2.82558	2.82904
	13	1.45717	1.89133	1.94459	2.48872	2.82513	2.82959
0.83333	1	1.47421	1.90097	1.92999	2.48839	2.8214	2.82764
	2	1.47681	1.89361	1.96291	2.48537	2.82156	2.82547
	3	1.4856	1.91102	1.94876	2.48518	2.82153	2.82502
	4	1.46354	1.901	1.94425	2.48351	2.81863	2.82244
	5	1.47125	1.9164	1.94741	2.48551	2.82436	2.8264
	6	1.47086	1.91195	1.94316	2.48452	2.82199	2.82385
	7	1.48448	1.90831	1.93434	2.48953	2.82264	2.82824
	8	1.44969	1.8855	1.93864	2.48562	2.82098	2.82582
	9	1.46076	1.90688	1.9531	2.48702	2.82497	2.82724
	10	1.46597	1.91194	1.94924	2.4864	2.82282	2.82592

		11	1.48792	1.92776	1.9397	2.48681	2.8237	2.82633
		12	1.48954	1.90714	1.95336	2.48742	2.82538	2.82725
		13	1.46459	1.89462	1.94906	2.48853	2.82544	2.82954
	1	1	1.48273	1.90543	1.92869	2.48831	2.82147	2.82791
		2	1.48273	1.89818	1.96649	2.48522	2.82175	2.82569
		3	1.49306	1.9171	1.94918	2.48532	2.82209	2.82555
		4	1.46819	1.9081	1.94531	2.48399	2.81902	2.82296
		5	1.47261	1.92132	1.95056	2.48532	2.82463	2.82644
		6	1.4797	1.92254	1.94795	2.48472	2.82268	2.82457
		7	1.49235	1.91491	1.93247	2.48935	2.82304	2.82831
		8	1.45131	1.89038	1.93813	2.48558	2.82162	2.82578
		9	1.45973	1.90617	1.95206	2.48709	2.82523	2.82749
		10	1.47708	1.91717	1.95232	2.48684	2.82311	2.82603
		11	1.5022	1.93678	1.93811	2.48683	2.82413	2.82667
		12	1.50326	1.90742	1.94449	2.48697	2.82569	2.82694
		13	1.46816	1.89868	1.95174	2.48845	2.82615	2.82963
	1.14	1	1.49338	1.91121	1.93216	2.48813	2.82186	2.8279
		2	1.49009	1.90166	1.97994	2.48552	2.82158	2.82569
		3	1.49463	1.90418	1.95098	2.4857	2.82212	2.82582
		4	1.46881	1.90953	1.95298	2.48421	2.81927	2.82323
		5	1.50392	1.91203	1.95836	2.48549	2.82482	2.82676
		6	1.49006	1.92253	1.94742	2.48498	2.82282	2.82503
		7	1.4701	1.92126	1.9399	2.48878	2.823	2.8282
		8	1.45736	1.8895	1.93941	2.48558	2.82194	2.82583
		9	1.47124	1.90641	1.94902	2.48721	2.8254	2.82776
		10	1.4812	1.91463	1.95028	2.48874	2.82198	2.82727
		11	1.51296	1.93709	1.9302	2.48709	2.82432	2.82692
		12	1.51373	1.9047	1.94451	2.48675	2.8259	2.82686
		13	1.4709	1.8986	1.95419	2.48804	2.82542	2.82935
ELMAM2	0.625	1	1.43779	1.88012	1.93619	2.48707	2.82166	2.82741
		2	1.45286	1.89355	1.9616	2.48406	2.82066	2.82546
		3	1.43667	1.88339	1.94607	2.48417	2.82043	2.82551
		4	1.43347	1.8818	1.949	2.48124	2.81679	2.82125
		5	1.43786	1.8911	1.94423	2.48489	2.82444	2.82717
		6	1.43973	1.88597	1.93638	2.48314	2.82168	2.82338

	7	1.44423	1.89042	1.94263	2.48805	2.82146	2.82828
	8	1.42535	1.88749	1.94629	2.48438	2.82148	2.82653
	9	1.42569	1.89161	1.95159	2.48654	2.8243	2.82763
	10	1.44354	1.89774	1.94978	2.485	2.82296	2.82635
	11	1.45126	1.896	1.94456	2.48694	2.82398	2.82845
	12	1.44739	1.88869	1.95799	2.48847	2.82603	2.82986
	13	1.43052	1.88096	1.96262	2.48875	2.82579	2.83172
0.71429	1	1.44889	1.88135	1.92976	2.48705	2.82156	2.82774
	2	1.46687	1.90531	1.95519	2.48393	2.82143	2.82589
	3	1.46523	1.89541	1.94117	2.48443	2.82152	2.82623
	4	1.44507	1.89044	1.94627	2.48213	2.8182	2.82223
	5	1.4569	1.89833	1.94056	2.48494	2.82444	2.8275
	6	1.46779	1.89141	1.93325	2.48349	2.82198	2.82447
	7	1.46136	1.89576	1.93759	2.48832	2.82201	2.8287
	8	1.43183	1.88562	1.94367	2.48502	2.82319	2.82685
	9	1.43941	1.8966	1.9508	2.48601	2.8246	2.82752
	10	1.45287	1.89792	1.95264	2.48499	2.82278	2.82641
	11	1.46128	1.89733	1.93315	2.48643	2.82401	2.82788
	12	1.45897	1.89522	1.94901	2.48757	2.8258	2.82911
	13	1.44876	1.88894	1.95352	2.48768	2.82525	2.82998
0.83333	1	1.46193	1.88827	1.92935	2.48729	2.82193	2.82811
	2	1.47113	1.90685	1.95924	2.48395	2.82183	2.82607
	3	1.47514	1.8973	1.94627	2.48462	2.82203	2.82628
	4	1.45492	1.90032	1.94819	2.4826	2.81911	2.82306
	5	1.45431	1.90446	1.94322	2.48458	2.82464	2.82673
	6	1.46848	1.89607	1.93657	2.4835	2.82236	2.82416
	7	1.4653	1.90205	1.93595	2.48848	2.82284	2.82876
	8	1.4463	1.88902	1.94452	2.48425	2.8215	2.82626
	9	1.44819	1.90394	1.95392	2.48585	2.82532	2.82764
	10	1.45985	1.9045	1.9558	2.48536	2.82311	2.82663
	11	1.46445	1.90494	1.93615	2.48584	2.82406	2.82666
	12	1.44902	1.89779	1.94917	2.48643	2.82571	2.8274
	13	1.45752	1.89375	1.9564	2.48758	2.82578	2.8299
1	1	1.41287	1.86666	1.91802	2.4878	2.82226	2.82813
	2	1.42283	1.87187	1.91377	2.4843	2.82228	2.82651

		3	1.43293	1.87456	1.92005	2.48537	2.82264	2.82643
		4	1.4112	1.86858	1.91455	2.48353	2.81979	2.82371
		5	1.41818	1.86951	1.92312	2.48514	2.82506	2.8267
		6	1.42973	1.8763	1.91978	2.48461	2.82317	2.82547
		7	1.43021	1.87938	1.92365	2.48896	2.82378	2.8288
		8	1.40649	1.86495	1.91885	2.48447	2.82272	2.82637
		9	1.41416	1.87949	1.92147	2.48652	2.82581	2.82811
		10	1.41295	1.86768	1.9199	2.48595	2.82385	2.82695
		11	1.42518	1.8737	1.91573	2.48666	2.82446	2.82694
		12	1.43763	1.86813	1.91357	2.48697	2.82619	2.82736
		13	1.41299	1.87291	1.92826	2.48808	2.82672	2.82993
	1.14	1	1.48313	1.89555	1.92926	2.48727	2.82217	2.82816
		2	1.48524	1.90957	1.97387	2.48432	2.82203	2.82604
		3	1.47203	1.90877	1.94603	2.4848	2.82239	2.82595
		4	1.46491	1.91044	1.95326	2.48329	2.81948	2.82356
		5	1.48769	1.90919	1.95533	2.48482	2.82506	2.8269
		6	1.48954	1.90787	1.94059	2.48427	2.82301	2.82519
		7	1.45498	1.90838	1.94009	2.48786	2.82332	2.8284
		8	1.45275	1.89485	1.94217	2.48442	2.82238	2.8262
		9	1.46614	1.90526	1.95187	2.48648	2.82562	2.82801
		10	1.47304	1.90836	1.95119	2.48771	2.82219	2.82786
		11	1.49251	1.91518	1.92567	2.4863	2.82454	2.82709
		12	1.47717	1.9052	1.94436	2.48607	2.82612	2.82694
		13	1.46534	1.8976	1.96002	2.48728	2.82561	2.82955
GID	0.625	1	1.43779	1.88012	1.93619	2.48707	2.82166	2.82741
		2	1.45286	1.89355	1.9616	2.48406	2.82066	2.82546
		3	1.43667	1.88339	1.94607	2.48417	2.82043	2.82551
		4	1.43347	1.8818	1.949	2.48124	2.81679	2.82125
		5	1.43786	1.8911	1.94423	2.48489	2.82444	2.82717
		6	1.43973	1.88597	1.93638	2.48314	2.82168	2.82338
		7	1.44423	1.89042	1.94263	2.48805	2.82146	2.82828
		8	1.42535	1.88749	1.94629	2.48438	2.82148	2.82653
		9	1.42569	1.89161	1.95159	2.48654	2.8243	2.82763
		10	1.44354	1.89774	1.94978	2.485	2.82296	2.82635
		11	1.45126	1.896	1.94456	2.48694	2.82398	2.82845

		12	1.44739	1.88869	1.95799	2.48847	2.82603	2.82986
		13	1.43052	1.88096	1.96262	2.48875	2.82579	2.83172
	0.71429	1	1.44889	1.88135	1.92976	2.48705	2.82156	2.82774
		2	1.46687	1.90531	1.95519	2.48393	2.82143	2.82589
		3	1.46523	1.89541	1.94117	2.48443	2.82152	2.82623
		4	1.44507	1.89044	1.94627	2.48213	2.8182	2.82223
		5	1.4569	1.89833	1.94056	2.48494	2.82444	2.8275
		6	1.46779	1.89141	1.93325	2.48349	2.82198	2.82447
		7	1.46136	1.89576	1.93759	2.48832	2.82201	2.8287
		8	1.43183	1.88562	1.94367	2.48502	2.82319	2.82685
		9	1.43941	1.8966	1.9508	2.48601	2.8246	2.82752
		10	1.45287	1.89792	1.95264	2.48499	2.82278	2.82641
		11	1.46128	1.89733	1.93315	2.48643	2.82401	2.82788
		12	1.45897	1.89522	1.94901	2.48757	2.8258	2.82911
		13	1.44876	1.88894	1.95352	2.48768	2.82525	2.82998
	0.83333	1	1.46193	1.88827	1.92935	2.48729	2.82193	2.82811
		2	1.47113	1.90685	1.95924	2.48395	2.82183	2.82607
		3	1.47514	1.8973	1.94627	2.48462	2.82203	2.82628
		4	1.45492	1.90032	1.94819	2.4826	2.81911	2.82306
		5	1.45431	1.90446	1.94322	2.48458	2.82464	2.82673
		6	1.46848	1.89607	1.93657	2.4835	2.82236	2.82416
		7	1.4653	1.90205	1.93595	2.48848	2.82284	2.82876
		8	1.4463	1.88902	1.94452	2.48425	2.8215	2.82626
		9	1.44819	1.90394	1.95392	2.48585	2.82532	2.82764
		10	1.45985	1.9045	1.9558	2.48536	2.82311	2.82663
		11	1.46445	1.90494	1.93615	2.48584	2.82406	2.82666
		12	1.44902	1.89779	1.94917	2.48643	2.82571	2.8274
		13	1.45752	1.89375	1.9564	2.48758	2.82578	2.8299
	1	1	1.47058	1.89043	1.92729	2.48735	2.82185	2.82827
		2	1.47765	1.90917	1.96197	2.48403	2.82198	2.82612
		3	1.46756	1.90171	1.94552	2.48445	2.82226	2.8257
		4	1.45927	1.90777	1.94846	2.4831	2.81929	2.82328
		5	1.45635	1.909	1.94625	2.48456	2.82479	2.82665
		6	1.47908	1.90433	1.94051	2.48392	2.82287	2.82475
		7	1.47331	1.90606	1.9353	2.48846	2.82313	2.82868

		8	1.44852	1.89225	1.94305	2.4844	2.82203	2.82613
		9	1.44779	1.90477	1.95385	2.48618	2.82541	2.82773
		10	1.47046	1.90883	1.95912	2.48583	2.82334	2.82668
		11	1.47515	1.91356	1.93633	2.48604	2.82434	2.82687
		12	1.45681	1.90367	1.9451	2.48617	2.8259	2.82703
		13	1.46188	1.89913	1.95826	2.48763	2.82639	2.82987
	1.14	1	1.48313	1.89555	1.92926	2.48727	2.82217	2.82816
		2	1.48524	1.90957	1.97387	2.48432	2.82203	2.82604
		3	1.47203	1.90877	1.94603	2.4848	2.82239	2.82595
		4	1.46491	1.91044	1.95326	2.48329	2.81948	2.82356
		5	1.48769	1.90919	1.95533	2.48482	2.82506	2.8269
		6	1.48954	1.90787	1.94059	2.48427	2.82301	2.82519
		7	1.45498	1.90838	1.94009	2.48786	2.82332	2.8284
		8	1.45275	1.89485	1.94217	2.48442	2.82238	2.8262
		9	1.46614	1.90526	1.95187	2.48648	2.82562	2.82801
		10	1.47304	1.90836	1.95119	2.48771	2.82219	2.82786
		11	1.49251	1.91518	1.92567	2.4863	2.82454	2.82709
		12	1.47717	1.9052	1.94436	2.48607	2.82612	2.82694
		13	1.46534	1.8976	1.96002	2.48728	2.82561	2.82955
HAR(B3LYP)	0.625	1	1.433173	1.930653	1.881314	2.487245	2.823001	2.828482
		2	1.440829	1.933153	1.886847	2.484305	2.821995	2.826116
		3	1.436898	1.932112	1.88408	2.484508	2.821786	2.825992
		4	1.434554	1.927203	1.876489	2.481683	2.81802	2.822091
		5	1.432038	1.934259	1.889359	2.485242	2.825987	2.828092
		6	1.428492	1.942559	1.881461	2.489083	2.826942	2.832418
		7	1.437683	1.93875	1.883438	2.487863	2.82268	2.829328
		8	1.437895	1.938735	1.883624	2.48788	2.82267	2.82937
		9	1.431582	1.938546	1.885637	2.48441	2.822715	2.827382
		10	1.448539	1.886535	1.943842	2.48661	2.825474	2.828663
		11	1.445783	1.890965	1.93209	2.485755	2.824083	2.827249
		12	1.429098	1.904925	1.924772	2.487043	2.825603	2.829092
		13	1.447165	1.933241	1.872563	2.488513	2.827513	2.830396
	0.71429	1	1.430804	1.927903	1.874446	2.487454	2.822598	2.828454
		2	1.444679	1.929	1.896987	2.484381	2.822296	2.826423
		3	1.432171	1.934246	1.883327	2.484238	2.82226	2.825081

	4	1.425384	1.925434	1.88041	2.482415	2.819107	2.822423
	5	1.426142	1.933512	1.88418	2.485163	2.82527	2.827397
	6	1.427722	1.932122	1.880432	2.488115	2.82617	2.830556
	7	1.434144	1.934029	1.88052	2.488164	2.822774	2.828965
	8	1.436668	1.932129	1.88073	2.488304	2.82275	2.829101
	9	1.42489	1.935919	1.882256	2.485125	2.824183	2.827471
	10	1.433302	1.937266	1.88486	2.486102	2.825377	2.828123
	11	1.432858	1.931358	1.886806	2.485631	2.823638	2.827018
	12	1.427733	1.896575	1.913428	2.487096	2.824827	2.828969
	13	1.434163	1.930635	1.864962	2.487235	2.826597	2.828288
0.83333	1	1.43131	1.925481	1.87426	2.487648	2.822725	2.828719
	2	1.438628	1.92992	1.894743	2.484268	2.822448	2.826618
	3	1.430323	1.930752	1.881997	2.484553	2.822521	2.825619
	4	1.424357	1.924444	1.880891	2.482864	2.819735	2.823227
	5	1.424213	1.931219	1.881664	2.485021	2.825278	2.827195
	6	1.427363	1.931582	1.881653	2.48799	2.826424	2.830364
	7	1.437358	1.930169	1.882225	2.488526	2.823638	2.829315
	8	1.439273	1.926255	1.882616	2.48864	2.823642	2.829432
	9	1.427497	1.934933	1.881147	2.484338	2.822438	2.826733
	10	1.433309	1.935142	1.887169	2.485971	2.825943	2.828275
	11	1.42893	1.932071	1.888688	2.485829	2.82375	2.827316
	12	1.426329	1.922898	1.875538	2.486384	2.824586	2.82721
	13	1.430778	1.921621	1.869308	2.486793	2.826349	2.827818
1	1	1.430409	1.925784	1.87562	2.487619	2.822583	2.828605
	2	1.440154	1.932609	1.895757	2.484322	2.822583	2.826638
	3	1.431028	1.931645	1.883445	2.484941	2.822811	2.826007
	4	1.424178	1.925083	1.885769	2.483446	2.81998	2.823634
	5	1.420804	1.932295	1.882825	2.485012	2.825324	2.827079
	6	1.426643	1.933872	1.884165	2.488056	2.826977	2.83028
	7	1.439341	1.883779	1.930685	2.488615	2.823771	2.829177
	8	1.441183	1.884415	1.926425	2.488708	2.823773	2.829255
	9	1.425826	1.934297	1.883885	2.484512	2.822941	2.826542
	10	1.431037	1.934942	1.886192	2.486349	2.826019	2.828343
	11	1.431422	1.934975	1.890168	2.486328	2.823979	2.827245
	12	1.427255	1.921013	1.876348	2.486604	2.824767	2.827353

		13	1.431261	1.919507	1.868764	2.486606	2.826437	2.827372
	1.14	1	1.43204	1.929749	1.87721	2.487587	2.822805	2.828578
		2	1.445479	1.944872	1.897684	2.487154	2.82439	2.828232
		3	1.432824	1.894471	1.936469	2.485193	2.82282	2.826182
		4	1.427601	1.928194	1.890903	2.483618	2.820131	2.823903
		5	1.420619	1.932313	1.889737	2.485256	2.825445	2.827261
		6	1.427544	1.933864	1.882555	2.487671	2.826178	2.829905
		7	1.435096	1.929992	1.880859	2.48458	2.823537	2.82561
		8	1.430564	1.931877	1.881732	2.488102	2.8241	2.828842
		9	1.426479	1.937794	1.882677	2.484505	2.823186	2.826652
		10	1.438501	1.934993	1.886425	2.486598	2.826096	2.828454
		11	1.43364	1.92822	1.890719	2.488181	2.822802	2.828484
		12	1.43523	1.916666	1.877104	2.48678	2.82496	2.827537
		13	1.445318	1.923247	1.872153	2.486367	2.826497	2.827156
HAR(BLYP)	0.625	1	1.442507	1.932987	1.882793	2.487287	2.823131	2.828562
		2	1.451835	1.939814	1.885733	2.484462	2.821928	2.826229
		3	1.447181	1.935997	1.884384	2.48455	2.821849	2.826116
		4	1.443982	1.933226	1.876675	2.481778	2.818045	2.82215
		5	1.440316	1.937197	1.889596	2.485292	2.826046	2.828122
		6	1.448285	1.930688	1.88765	2.483381	2.823185	2.824417
		7	1.452155	1.942196	1.884678	2.488011	2.822811	2.829345
		8	1.445083	1.943318	1.885624	2.484517	2.822823	2.827571
		9	1.448539	1.943842	1.886535	2.48661	2.825474	2.828663
		10	1.458111	1.936681	1.890953	2.485745	2.824129	2.827514
		11	1.438205	1.92512	1.906148	2.487064	2.82575	2.829117
		12	1.454969	1.93866	1.875344	2.48855	2.82761	2.830429
		13	1.438995	1.946107	1.881069	2.489106	2.827054	2.832561
	0.71429	1	1.437401	1.930475	1.875294	2.487483	2.822664	2.828396
		2	1.452902	1.933838	1.896769	2.484447	2.82235	2.826393
		3	1.439228	1.937093	1.883564	2.484246	2.822307	2.825026
		4	1.433355	1.92816	1.881437	2.482373	2.819177	2.822431
		5	1.432079	1.935085	1.884316	2.485136	2.825316	2.827356
		6	1.449275	1.9271	1.882334	2.483921	2.82276	2.825168
		7	1.443503	1.935511	1.881284	2.488196	2.822832	2.828894
		8	1.434609	1.94086	1.882235	2.485209	2.824294	2.827461

	9	1.442947	1.940738	1.885204	2.48614	2.825571	2.828137
	10	1.442244	1.936196	1.887229	2.485672	2.823698	2.827061
	11	1.434298	1.896957	1.914474	2.487109	2.824898	2.828932
	12	1.440666	1.935428	1.866648	2.487224	2.826645	2.828219
	13	1.435657	1.93457	1.880517	2.488108	2.82626	2.830522
0.83333	1	1.43661	1.928037	1.874622	2.487647	2.822758	2.828648
	2	1.44497	1.934219	1.894401	2.484274	2.822485	2.826572
	3	1.435817	1.933927	1.881952	2.484536	2.822539	2.825563
	4	1.430576	1.926986	1.881858	2.482825	2.819762	2.823208
	5	1.429127	1.933002	1.881532	2.484976	2.825281	2.827134
	6	1.43969	1.929847	1.879047	2.48381	2.823036	2.824692
	7	1.444199	1.932693	1.882885	2.488548	2.823653	2.829222
	8	1.436202	1.939252	1.880856	2.484372	2.822522	2.826682
	9	1.439944	1.939098	1.886842	2.48598	2.826045	2.82821
	10	1.437046	1.936466	1.888901	2.485841	2.823796	2.827296
	11	1.428102	1.926252	1.874885	2.48637	2.824618	2.827117
	12	1.436645	1.926379	1.871846	2.486759	2.826367	2.827764
	13	1.43453	1.933863	1.881521	2.487961	2.826483	2.83032
1	1	1.435294	1.928351	1.875795	2.487618	2.822623	2.828566
	2	1.446821	1.936969	1.895469	2.484328	2.822615	2.826618
	3	1.43659	1.934963	1.883471	2.484934	2.82283	2.825984
	4	1.430635	1.927633	1.886704	2.483422	2.820003	2.82364
	5	1.426291	1.934287	1.882774	2.484988	2.825333	2.827051
	6	1.438754	1.931625	1.881306	2.484282	2.82345	2.825263
	7	1.446293	1.884175	1.933235	2.48864	2.823789	2.82913
	8	1.434229	1.93858	1.88346	2.484527	2.823017	2.82652
	9	1.43898	1.938048	1.885354	2.486339	2.826058	2.828331
	10	1.440955	1.937708	1.890425	2.486303	2.824034	2.827278
	11	1.429047	1.924778	1.875684	2.486599	2.824809	2.82731
	12	1.438073	1.924425	1.871526	2.486584	2.826463	2.827348
	13	1.434162	1.93603	1.884031	2.488042	2.827028	2.830266
1.14	1	1.436787	1.932396	1.877035	2.487596	2.822836	2.828552
	2	1.45263	1.948954	1.897258	2.487166	2.824431	2.828214
	3	1.438732	1.939286	1.894401	2.4852	2.822839	2.826166
	4	1.434997	1.931062	1.890945	2.483618	2.820146	2.823906

		5	1.424317	1.933198	1.889175	2.485262	2.825452	2.827245
		6	1.438998	1.933205	1.880728	2.484588	2.823548	2.825603
		7	1.434577	1.935965	1.881128	2.488117	2.8241	2.828827
		8	1.436686	1.941412	1.882151	2.484509	2.823277	2.826658
		9	1.446695	1.938486	1.886051	2.486608	2.826139	2.828437
		10	1.440954	1.932252	1.890708	2.488214	2.82284	2.828462
		11	1.436258	1.921296	1.876754	2.486782	2.824986	2.827494
		12	1.452799	1.929056	1.875353	2.486359	2.82652	2.82715
		13	1.435035	1.935949	1.882438	2.487662	2.826223	2.829897
MR_no	1.19	1	1.47144	1.93116	2.00347	2.48768	2.82293	2.82830
		2	1.47814	1.96301	1.85706	2.48696	2.82467	2.82782
		3	1.40371	1.96461	1.91482	2.48541	2.82267	2.82615
		4	1.46054	1.93292	1.86609	2.48335	2.82012	2.82376
		5	1.44605	1.95570	2.09864	2.48559	2.82525	2.82724
		6	1.45244	1.89257	1.94475	2.48471	2.82363	2.82547
		7	1.66862	1.96246	1.89406	2.48766	2.82444	2.82842
		8	1.4777	1.85753	1.87971	2.48432	2.82419	2.82620
		9	1.48268	1.92395	1.89289	2.48675	2.82619	2.82861
		10	1.46768	1.84812	1.95291	2.48880	2.82511	2.82720
		11	1.52267	1.91516	1.95839	2.48680	2.82512	2.82711
		12	1.48494	1.93783	1.91431	2.48670	2.82654	2.82720
		13	1.509	2.00282	1.92106	2.48785	2.82641	2.82980
MR	1.18	1	1.415444	1.865558	1.987741	2.487666	2.823053	2.828282
		2	1.413398	1.876677	1.890471	2.487134	2.824983	2.827585
		3	1.411714	1.879194	1.921951	2.485366	2.822799	2.826071
		4	1.412315	1.855904	1.95424	2.483435	2.820232	2.823575
		5	1.412366	1.86779	1.979727	2.485498	2.82534	2.827166
		6	1.411809	1.862458	1.974016	2.484729	2.823663	2.825436
		7	1.414686	1.872665	1.894167	2.487643	2.824509	2.828402
		8	1.410877	1.889826	1.896575	2.484503	2.824087	2.826138
		9	1.41574	1.860642	1.893526	2.486743	2.826248	2.828588
		10	1.415893	1.863204	1.939111	2.488882	2.824993	2.827241
		11	1.41626	1.871864	1.956078	2.486863	2.825132	2.827076
		12	1.415434	1.873862	1.939168	2.486752	2.826568	2.827155
		13	1.415954	1.877456	1.916698	2.487886	2.826479	2.829761

Neutron Diffraction	N/A	1	1.411	1.88	1.924	2.487	2.827	2.83
		2	1.407	1.879	1.922	2.486	2.828	2.83
		3	1.414	1.881	1.922	2.487	2.827	2.829
		4	1.407	1.882	1.918	2.486	2.829	2.828
		5	1.411	1.882	1.92	2.487	2.827	2.828
		6	1.407	1.881	1.924	2.487	2.827	2.829
		7	1.409	1.879	1.922	2.487	2.827	2.827
		8	1.408	1.88	1.922	2.485	2.828	2.829
		9	1.407	1.881	1.919	2.487	2.827	2.828
		10	1.411	1.881	1.923	2.488	2.825	2.83
		11	1.407	1.88	1.921	2.485	2.829	2.828
		12	1.41	1.878	1.919	2.485	2.826	2.828
			1.406	1.881	1.918	2.487	2.829	2.828
		13	1.408	1.881	1.92	2.488	2.829	2.827

z

Method	Resolution [Å $^{-1}$]	nr set	C1 - C1 ls error	C1 - O1 ls error	C1 - O2 ls error	O1 - H1 ls error	O3 - H2 ls error	O3 - H3 ls error
IAM	0.625	1	0.00311	0.00105	0.00101	0.01677	0.0185	0.01906
		2	0.00214	0.0011	0.00111	0.01866	0.02084	0.01961
		3	0.00258	0.00104	0.00104	0.0195	0.0216	0.02017
		4	0.00234	0.00103	0.00104	0.01649	0.01881	0.01834
		5	0.00252	0.00102	0.00102	0.017	0.01973	0.01913
		6	0.00283	0.00102	0.00102	0.01877	0.01951	0.01996
		7	0.00447	0.00111	0.00122	0.0167	0.021	0.0197
		8	0.00179	0.00107	0.00111	0.01486	0.01909	0.0179
		9	0.0018	0.00109	0.00114	0.01549	0.01905	0.01857
		10	0.00189	0.00103	0.00105	0.01746	0.0196	0.01854
		11	0.00224	0.00089	0.00088	0.01832	0.01359	0.01374
		12	0.00295	0.00081	0.00088	0.01935	0.01544	0.01564
		13	0.00272	0.0009	0.00096	0.01525	0.01474	0.01456
	0.71429	1	0.0028	0.00071	0.00073	0.01469	0.01517	0.01602
		2	0.00157	0.00078	0.00083	0.01671	0.01427	0.01473
		3	0.00176	0.00068	0.00069	0.01825	0.01443	0.01463
		4	0.00159	0.00069	0.00073	0.01564	0.01322	0.01333
		5	0.00162	0.00062	0.00066	0.01612	0.0131	0.01297
		6	0.00182	0.00064	0.00067	0.01596	0.01248	0.0133
		7	0.00343	0.00071	0.00079	0.01654	0.01391	0.01309
		8	0.00146	0.00075	0.00079	0.013	0.01289	0.01266
		9	0.00146	0.00073	0.00081	0.0149	0.0134	0.01363
		10	0.00154	0.00073	0.00078	0.0152	0.01281	0.01307
		11	0.00221	0.00066	0.00065	0.01806	0.0125	0.013
		12	0.00261	0.00055	0.00058	0.01744	0.01633	0.01549
		13	0.00199	0.00059	0.00065	0.01394	0.01363	0.01323
	0.83333	1	0.00169	0.00052	0.00051	0.01361	0.01034	0.01067
		2	0.00123	0.00059	0.00059	0.01532	0.01266	0.01264
		3	0.00152	0.0005	0.00047	0.01678	0.01312	0.01324
		4	0.0013	0.00051	0.00051	0.01409	0.01168	0.0116
		5	0.0014	0.00044	0.00044	0.01513	0.01208	0.01192
		6	0.00159	0.00046	0.00046	0.01483	0.01143	0.0119
		7	0.00249	0.00052	0.00054	0.0147	0.01172	0.01148
		8	0.00119	0.00056	0.00055	0.01159	0.01147	0.01105
		9	0.00089	0.00049	0.00052	0.01372	0.0127	0.01224
		10	0.00133	0.0006	0.00062	0.01398	0.01137	0.01187

		11	0.00197	0.00047	0.00045	0.01591	0.01096	0.01155
		12	0.00192	0.00041	0.00039	0.01583	0.01481	0.01409
		13	0.00125	0.00044	0.00043	0.0125	0.01198	0.01167
	1	1	0.00133	0.00037	0.00037	0.01101	0.00826	0.00865
		2	0.00097	0.00045	0.00046	0.01342	0.0109	0.01086
		3	0.00121	0.00035	0.00034	0.01362	0.01062	0.01089
		4	0.0007	0.00037	0.00038	0.01204	0.00984	0.00973
		5	0.00111	0.00031	0.00031	0.01256	0.01005	0.00976
		6	0.00128	0.00033	0.00033	0.01243	0.00946	0.0099
		7	0.00154	0.00038	0.0004	0.0119	0.00956	0.00937
		8	0.00066	0.00039	0.0004	0.00949	0.00954	0.00918
		9	0.00061	0.00033	0.00035	0.01156	0.011	0.01043
		10	0.00112	0.00049	0.00051	0.01228	0.01008	0.01047
		11	0.0016	0.00033	0.00032	0.01303	0.00887	0.00955
		12	0.00115	0.0003	0.0003	0.01301	0.01222	0.0115
		13	0.00101	0.00032	0.00032	0.01053	0.01024	0.00999
	1.14	1	0.00117	0.00031	0.00031	0.00999	0.00739	0.00775
		2	0.0009	0.00043	0.00043	0.01222	0.01015	0.00994
		3	0.00109	0.0003	0.00029	0.01228	0.00985	0.00985
		4	0.00063	0.00033	0.00033	0.01111	0.00941	0.00917
		5	0.00109	0.00027	0.00027	0.01189	0.00906	0.00889
		6	0.00111	0.00027	0.00028	0.01085	0.00807	0.00854
		7	0.0015	0.00032	0.00033	0.0105	0.00868	0.00831
		8	0.00059	0.00034	0.00033	0.00864	0.00879	0.00823
		9	0.00052	0.00026	0.00026	0.0105	0.00978	0.00942
		10	0.00114	0.00049	0.00053	0.01322	0.01072	0.01088
		11	0.00154	0.00031	0.00031	0.01249	0.00873	0.00917
		12	0.00098	0.00025	0.00024	0.01123	0.01067	0.01027
		13	0.00091	0.00027	0.00027	0.00959	0.00926	0.00916
UBDB	0.625	1	0.00077	0.00055	0.00054	0.01012	0.00915	0.00882
		2	0.00093	0.00063	0.00064	0.01191	0.01015	0.01012
		3	0.00073	0.00055	0.00055	0.01141	0.00999	0.00954
		4	0.00081	0.00056	0.00057	0.01032	0.009	0.00911
		5	0.00073	0.00051	0.00052	0.00988	0.00888	0.00866
		6	0.00081	0.00054	0.00055	0.01103	0.00936	0.00942
		7	0.00096	0.00072	0.00079	0.01402	0.01251	0.01132
		8	0.00078	0.0006	0.00062	0.00962	0.00945	0.00912
		9	0.00087	0.00064	0.00066	0.01049	0.00972	0.00991

		10	0.00086	0.00057	0.00058	0.01057	0.00949	0.0094
		11	0.00083	0.0006	0.0006	0.01445	0.00998	0.01124
		12	0.0006	0.00044	0.00049	0.01167	0.01044	0.01017
		13	0.00081	0.00053	0.00056	0.01033	0.00986	0.00943
	0.71429	1	0.00063	0.0004	0.00041	0.00961	0.00812	0.00789
		2	0.00086	0.00052	0.00054	0.01244	0.01016	0.01048
		3	0.00069	0.00045	0.00046	0.01291	0.01066	0.01068
		4	0.0007	0.00043	0.00045	0.01063	0.00877	0.009
		5	0.00058	0.00036	0.00038	0.01001	0.00844	0.00816
		6	0.00067	0.0004	0.00042	0.01114	0.00913	0.00923
		7	0.0007	0.00047	0.00053	0.01274	0.01054	0.00971
		8	0.00067	0.00046	0.00048	0.00894	0.00855	0.00809
		9	0.00073	0.00047	0.00051	0.01036	0.00914	0.00933
		10	0.00073	0.00043	0.00046	0.00987	0.00854	0.00851
		11	0.00064	0.00042	0.00041	0.0114	0.00943	0.00929
		12	0.00055	0.00035	0.00037	0.01368	0.0115	0.01139
		13	0.00062	0.00035	0.00038	0.00977	0.0089	0.0086
	0.83333	1	0.00049	0.00031	0.00031	0.00883	0.00741	0.00713
		2	0.00065	0.00042	0.00042	0.01139	0.00932	0.00932
		3	0.00047	0.00032	0.00029	0.01091	0.00927	0.0088
		4	0.00049	0.00031	0.00031	0.00911	0.00762	0.00772
		5	0.00043	0.00026	0.00026	0.00961	0.00804	0.00774
		6	0.00046	0.00029	0.00028	0.00963	0.00814	0.00784
		7	0.00047	0.00033	0.00034	0.01071	0.00862	0.00814
		8	0.00051	0.00036	0.00035	0.00834	0.00776	0.0073
		9	0.00052	0.00033	0.00035	0.00973	0.0088	0.00858
		10	0.00062	0.00036	0.00038	0.00917	0.00774	0.00775
		11	0.00044	0.00029	0.00027	0.00953	0.00796	0.00754
		12	0.0004	0.00025	0.00024	0.01233	0.01086	0.00988
		13	0.00044	0.00027	0.00026	0.00873	0.00796	0.00752
	1	1	0.00035	0.00023	0.00022	0.0074	0.00612	0.00593
		2	0.00052	0.00035	0.00035	0.01072	0.0087	0.00863
		3	0.00033	0.00023	0.00022	0.00982	0.00807	0.00779
		4	0.00037	0.00025	0.00025	0.00855	0.00703	0.00709
		5	0.0003	0.0002	0.0002	0.00906	0.00738	0.00718
		6	0.00032	0.00021	0.00021	0.00878	0.00721	0.007
		7	0.00033	0.00024	0.00026	0.00935	0.00745	0.00712
		8	0.00039	0.00028	0.00028	0.00751	0.00696	0.00663

		9	0.00036	0.00024	0.00025	0.00897	0.00828	0.00793
		10	0.00055	0.00032	0.00034	0.00878	0.00741	0.00746
		11	0.0003	0.0002	0.0002	0.00841	0.00693	0.00662
		12	0.00029	0.00019	0.00019	0.01152	0.00995	0.00907
		13	0.00033	0.00021	0.00021	0.00816	0.00751	0.00699
	1.14	1	0.0003	0.00019	0.00019	0.00717	0.00582	0.00567
		2	0.00049	0.00033	0.00033	0.01016	0.00855	0.00808
		3	0.00032	0.00021	0.00021	0.00996	0.00826	0.00785
		4	0.00035	0.00023	0.00024	0.0084	0.00714	0.00708
		5	0.0003	0.00019	0.00019	0.00997	0.00757	0.00746
		6	0.00027	0.00017	0.00018	0.00783	0.00638	0.00635
		7	0.00028	0.0002	0.00021	0.00809	0.00682	0.00652
		8	0.00037	0.00026	0.00025	0.00761	0.00692	0.00646
		9	0.0003	0.0002	0.0002	0.00883	0.00788	0.00765
		10	0.00056	0.00033	0.00036	0.00973	0.00803	0.00821
		11	0.00029	0.00019	0.00019	0.00817	0.00689	0.00649
		12	0.00024	0.00015	0.00015	0.01102	0.00955	0.00865
		13	0.0003	0.00019	0.00019	0.00794	0.00727	0.00681
ELMAM2	0.625	1	0.00072	0.00052	0.00051	0.00933	0.00882	0.00798
		2	0.00087	0.00059	0.00059	0.01117	0.00925	0.00886
		3	0.00068	0.00051	0.00051	0.01066	0.00938	0.00856
		4	0.00078	0.00054	0.00055	0.00995	0.00858	0.00821
		5	0.00069	0.00048	0.00049	0.00928	0.00859	0.00785
		6	0.00078	0.00052	0.00052	0.01041	0.00933	0.00856
		7	0.0009	0.00067	0.00074	0.01353	0.01186	0.01034
		8	0.00074	0.00057	0.00059	0.00927	0.00868	0.00813
		9	0.00083	0.0006	0.00062	0.01005	0.00905	0.00871
		10	0.00085	0.00056	0.00057	0.01046	0.00926	0.00878
		11	0.00083	0.0006	0.00059	0.01395	0.0103	0.01079
		12	0.00057	0.00042	0.00047	0.01135	0.01018	0.00944
		13	0.00077	0.0005	0.00054	0.01005	0.00913	0.00837
	0.71429	1	0.0006	0.00038	0.00039	0.00903	0.00798	0.00725
		2	0.00084	0.00051	0.00053	0.01215	0.00962	0.00956
		3	0.00066	0.00043	0.00044	0.01264	0.01022	0.00976
		4	0.00068	0.00042	0.00043	0.01049	0.00845	0.00828
		5	0.00057	0.00035	0.00037	0.00993	0.00836	0.00783
		6	0.00064	0.00039	0.0004	0.01082	0.00914	0.0085
		7	0.00066	0.00044	0.00049	0.01246	0.00994	0.00892

	8	0.00063	0.00044	0.00045	0.00856	0.00785	0.00719
	9	0.00069	0.00044	0.00049	0.01011	0.00865	0.00834
	10	0.00072	0.00043	0.00045	0.00989	0.00838	0.00795
	11	0.00062	0.0004	0.00039	0.0108	0.00951	0.0087
	12	0.00053	0.00034	0.00036	0.01358	0.01079	0.01064
	13	0.00059	0.00034	0.00037	0.00951	0.00838	0.0078
0.83333	1	0.00048	0.0003	0.0003	0.00851	0.00741	0.00657
	2	0.00065	0.00042	0.00042	0.01138	0.00888	0.00861
	3	0.00048	0.00032	0.0003	0.01176	0.00947	0.00887
	4	0.00049	0.00031	0.00031	0.0093	0.00744	0.0073
	5	0.00042	0.00026	0.00026	0.00959	0.00807	0.00729
	6	0.00047	0.00029	0.00028	0.00987	0.00851	0.00752
	7	0.00045	0.00031	0.00033	0.01053	0.00812	0.00742
	8	0.00049	0.00034	0.00034	0.00823	0.00727	0.00658
	9	0.00052	0.00033	0.00034	0.00999	0.00849	0.00793
	10	0.00062	0.00037	0.00038	0.00947	0.00783	0.00739
	11	0.00043	0.00028	0.00027	0.00936	0.0083	0.00713
	12	0.0004	0.00025	0.00024	0.01286	0.01035	0.00947
	13	0.00043	0.00026	0.00026	0.00882	0.00763	0.00696
1	1	0.00069	0.00022	0.00022	0.00616	0.00576	0.0055
	2	0.00069	0.00034	0.00035	0.01036	0.00923	0.0094
	3	0.00073	0.00024	0.00023	0.00909	0.00819	0.0079
	4	0.0006	0.00025	0.00025	0.00794	0.00695	0.00715
	5	0.00064	0.00021	0.00021	0.00802	0.00719	0.00703
	6	0.00071	0.00022	0.00022	0.00784	0.00687	0.00692
	7	0.00062	0.00023	0.00024	0.00741	0.00695	0.00652
	8	0.00055	0.00027	0.00026	0.00668	0.00695	0.00656
	9	0.00057	0.00025	0.00026	0.00873	0.0084	0.00834
	10	0.00064	0.0003	0.00031	0.00792	0.00754	0.00756
	11	0.0008	0.0002	0.0002	0.00677	0.00607	0.00584
	12	0.0007	0.00021	0.00021	0.00966	0.00926	0.00865
	13	0.0006	0.00023	0.00022	0.00727	0.00745	0.00704
1.14	1	0.00031	0.0002	0.0002	0.00729	0.00614	0.00541
	2	0.0005	0.00033	0.00034	0.01031	0.00814	0.00758
	3	0.00033	0.00022	0.00021	0.00974	0.00811	0.00724
	4	0.00036	0.00024	0.00025	0.00883	0.00705	0.00681
	5	0.00031	0.00019	0.0002	0.00978	0.00776	0.0071
	6	0.00027	0.00018	0.00018	0.00817	0.00676	0.00614

		7	0.00027	0.0002	0.0002	0.00778	0.00657	0.00588
		8	0.00036	0.00026	0.00025	0.00774	0.00659	0.00593
		9	0.00031	0.0002	0.00021	0.00935	0.00788	0.00724
		10	0.00057	0.00033	0.00036	0.01019	0.00806	0.00789
		11	0.0003	0.0002	0.00019	0.00827	0.0074	0.00618
		12	0.00024	0.00016	0.00015	0.01207	0.00946	0.0085
		13	0.0003	0.00019	0.00019	0.00815	0.00704	0.00636
GID	0.625	1	0.00072	0.00052	0.00051	0.00933	0.00882	0.00798
		2	0.00087	0.00059	0.00059	0.01117	0.00925	0.00886
		3	0.00068	0.00051	0.00051	0.01066	0.00938	0.00856
		4	0.00078	0.00054	0.00055	0.00995	0.00858	0.00821
		5	0.00069	0.00048	0.00049	0.00928	0.00859	0.00785
		6	0.00078	0.00052	0.00052	0.01041	0.00933	0.00856
		7	0.0009	0.00067	0.00074	0.01353	0.01186	0.01034
		8	0.00074	0.00057	0.00059	0.00927	0.00868	0.00813
		9	0.00083	0.0006	0.00062	0.01005	0.00905	0.00871
		10	0.00085	0.00056	0.00057	0.01046	0.00926	0.00878
		11	0.00083	0.0006	0.00059	0.01395	0.0103	0.01079
		12	0.00057	0.00042	0.00047	0.01135	0.01018	0.00944
		13	0.00077	0.0005	0.00054	0.01005	0.00913	0.00837
	0.71429	1	0.0006	0.00038	0.00039	0.00903	0.00798	0.00725
		2	0.00084	0.00051	0.00053	0.01215	0.00962	0.00956
		3	0.00066	0.00043	0.00044	0.01264	0.01022	0.00976
		4	0.00068	0.00042	0.00043	0.01049	0.00845	0.00828
		5	0.00057	0.00035	0.00037	0.00993	0.00836	0.00783
		6	0.00064	0.00039	0.0004	0.01082	0.00914	0.0085
		7	0.00066	0.00044	0.00049	0.01246	0.00994	0.00892
		8	0.00063	0.00044	0.00045	0.00856	0.00785	0.00719
		9	0.00069	0.00044	0.00049	0.01011	0.00865	0.00834
		10	0.00072	0.00043	0.00045	0.00989	0.00838	0.00795
		11	0.00062	0.0004	0.00039	0.0108	0.00951	0.0087
		12	0.00053	0.00034	0.00036	0.01358	0.01079	0.01064
		13	0.00059	0.00034	0.00037	0.00951	0.00838	0.0078
	0.83333	1	0.00048	0.0003	0.0003	0.00851	0.00741	0.00657
		2	0.00065	0.00042	0.00042	0.01138	0.00888	0.00861
		3	0.00048	0.00032	0.0003	0.01176	0.00947	0.00887
		4	0.00049	0.00031	0.00031	0.0093	0.00744	0.0073
		5	0.00042	0.00026	0.00026	0.00959	0.00807	0.00729

		6	0.00047	0.00029	0.00028	0.00987	0.00851	0.00752
		7	0.00045	0.00031	0.00033	0.01053	0.00812	0.00742
		8	0.00049	0.00034	0.00034	0.00823	0.00727	0.00658
		9	0.00052	0.00033	0.00034	0.00999	0.00849	0.00793
		10	0.00062	0.00037	0.00038	0.00947	0.00783	0.00739
		11	0.00043	0.00028	0.00027	0.00936	0.0083	0.00713
		12	0.0004	0.00025	0.00024	0.01286	0.01035	0.00947
		13	0.00043	0.00026	0.00026	0.00882	0.00763	0.00696
	1	1	0.00035	0.00022	0.00022	0.0073	0.00625	0.00553
		2	0.00051	0.00034	0.00035	0.01069	0.00822	0.00796
		3	0.00033	0.00023	0.00022	0.01011	0.00811	0.00732
		4	0.00038	0.00025	0.00026	0.00878	0.00691	0.00674
		5	0.0003	0.0002	0.0002	0.00909	0.00752	0.00679
		6	0.00033	0.00021	0.00022	0.00909	0.00762	0.00674
		7	0.00032	0.00023	0.00025	0.00927	0.00708	0.00649
		8	0.00038	0.00027	0.00027	0.00745	0.00655	0.006
		9	0.00036	0.00024	0.00025	0.00927	0.00802	0.00731
		10	0.00056	0.00033	0.00034	0.00918	0.00756	0.00713
		11	0.0003	0.0002	0.0002	0.00837	0.00739	0.00625
		12	0.00029	0.00019	0.00019	0.01229	0.00969	0.0088
		13	0.00033	0.00021	0.00021	0.00835	0.00724	0.00652
	1.14	1	0.00031	0.0002	0.0002	0.00729	0.00614	0.00541
		2	0.0005	0.00033	0.00034	0.01031	0.00814	0.00758
		3	0.00033	0.00022	0.00021	0.00974	0.00811	0.00724
		4	0.00036	0.00024	0.00025	0.00883	0.00705	0.00681
		5	0.00031	0.00019	0.0002	0.00978	0.00776	0.0071
		6	0.00027	0.00018	0.00018	0.00817	0.00676	0.00614
		7	0.00027	0.0002	0.0002	0.00778	0.00657	0.00588
		8	0.00036	0.00026	0.00025	0.00774	0.00659	0.00593
		9	0.00031	0.0002	0.00021	0.00935	0.00788	0.00724
		10	0.00057	0.00033	0.00036	0.01019	0.00806	0.00789
		11	0.0003	0.0002	0.00019	0.00827	0.0074	0.00618
		12	0.00024	0.00016	0.00015	0.01207	0.00946	0.0085
		13	0.0003	0.00019	0.00019	0.00815	0.00704	0.00636
HAR(B3LYP)	0.625	1	0.00052	0.000369	0.000373	0.009	0.008	0.007
		2	0.000692	0.000467	0.000469	0.01	0.009	0.008
		3	0.000483	0.000361	0.000368	0.009	0.008	0.007
		4	0.000541	0.000372	0.000378	0.009	0.007	0.007

	5	0.000489	0.00034	0.000348	0.008	0.007	0.006
	6	0.000534	0.000357	0.000367	0.009	0.008	0.007
	7	0.000534	0.000398	0.000441	0.008	0.007	0.000391
	8	0.000468	0.000358	0.000368	0.007	0.006	0.006
	9	0.000642	0.000468	0.000485	0.01	0.008	0.008
	10	0.000533	0.00035	0.000357	0.009	0.007	0.007
	11	0.000662	0.000472	0.00049	0.01	0.01	0.01
	12	0.000424	0.00032	0.000353	0.01	0.009	0.008
	13	0.00054	0.000352	0.000375	0.009	0.007	0.007
0.71429	1	0.000431	0.000272	0.00028	0.008	0.006	0.006
	2	0.000683	0.000413	0.000432	0.01	0.009	0.009
	3	0.00051	0.000334	0.000338	0.01	0.009	0.007
	4	0.000499	0.000306	0.000318	0.009	0.007	0.007
	5	0.000412	0.000253	0.000266	0.008	0.007	0.006
	6	0.000474	0.000284	0.000299	0.009	0.007	0.007
	7	0.000417	0.000279	0.000313	0.007	0.006	0.000262
	8	0.000408	0.00028	0.000293	0.007	0.006	0.006
	9	0.000527	0.000338	0.000375	0.009	0.008	0.007
	10	0.000474	0.00028	0.000297	0.008	0.006	0.006
	11	0.000581	0.000374	0.000373	0.01	0.009	0.01
	12	0.000424	0.00027	0.000281	0.01	0.009	0.007
	13	0.000413	0.000237	0.000257	0.008	0.006	0.006
0.83333	1	0.000346	0.00022	0.000215	0.007	0.006	0.005
	2	0.000525	0.000336	0.000336	0.01	0.008	0.008
	3	0.00036	0.000241	0.000226	0.01	0.008	0.006
	4	0.000357	0.000228	0.000227	0.007	0.006	0.006
	5	0.00031	0.000194	0.000192	0.007	0.006	0.005
	6	0.000331	0.000204	0.000203	0.008	0.006	0.006
	7	0.000293	0.000204	0.000215	0.006	0.005	0.000191
	8	0.000319	0.000225	0.000224	0.006	0.005	0.005
	9	0.0004	0.000253	0.000268	0.008	0.008	0.007
	10	0.000429	0.000252	0.000261	0.008	0.006	0.006
	11	0.000312	0.000204	0.000192	0.007	0.006	0.006
	12	0.000311	0.000198	0.000189	0.01	0.008	0.007
	13	0.000298	0.000184	0.000181	0.007	0.006	0.005
1	1	0.000238	0.000153	0.000153	0.006	0.005	0.004
	2	0.000414	0.000278	0.000284	0.009	0.007	0.007
	3	0.000253	0.000174	0.000168	0.009	0.007	0.005

		4	0.000279	0.000186	0.000191	0.007	0.006	0.005
		5	0.000223	0.000145	0.000148	0.007	0.006	0.005
		6	0.000232	0.000152	0.000154	0.007	0.006	0.005
		7	0.000206	0.000149	0.000161	0.005	0.005	0.000141
		8	0.000261	0.000187	0.000188	0.006	0.005	0.005
		9	0.000285	0.000188	0.000202	0.008	0.007	0.007
		10	0.000404	0.000237	0.000249	0.008	0.006	0.006
		11	0.000206	0.000136	0.000133	0.006	0.005	0.005
		12	0.000227	0.00015	0.000147	0.009	0.007	0.006
		13	0.000238	0.000155	0.000156	0.007	0.006	0.005
	1.14	1	0.000228	0.000145	0.000146	0.006	0.005	0.004
		2	0.0004	0.000267	0.000271	0.009	0.007	0.007
		3	0.00026	0.000173	0.00017	0.009	0.007	0.006
		4	0.000275	0.000183	0.000188	0.008	0.006	0.006
		5	0.000248	0.000157	0.000163	0.009	0.006	0.006
		6	0.000199	0.000129	0.000133	0.007	0.005	0.005
		7	0.000199	0.000142	0.000149	0.006	0.005	0.005
		8	0.000273	0.000194	0.000191	0.007	0.006	0.006
		9	0.000256	0.000167	0.00017	0.008	0.007	0.007
		10	0.000432	0.000253	0.000277	0.009	0.007	0.007
		11	0.000206	0.000135	0.000133	0.006	0.005	0.005
		12	0.000194	0.000125	0.000121	0.009	0.007	0.006
		13	0.000223	0.000142	0.000145	0.007	0.006	0.005
HAR(BLYP)	0.625	1	0.000535	0.000381	0.000384	0.009	0.008	0.007
		2	0.000721	0.000488	0.000488	0.01	0.009	0.009
		3	0.000504	0.000378	0.000384	0.01	0.008	0.007
		4	0.000563	0.000388	0.000393	0.009	0.007	0.007
		5	0.000512	0.000357	0.000365	0.009	0.008	0.007
		6	0.000558	0.000374	0.000384	0.01	0.008	0.007
		7	0.000546	0.00041	0.000452	0.01	0.008	0.007
		8	0.000485	0.000372	0.000381	0.008	0.007	0.007
		9	0.000642	0.000468	0.000485	0.01	0.008	0.008
		10	0.000549	0.000361	0.000368	0.009	0.007	0.007
		11	0.000663	0.000474	0.000492	0.01	0.01	0.01
		12	0.000434	0.00033	0.000363	0.01	0.009	0.008
		13	0.000545	0.000356	0.000379	0.009	0.007	0.007
	0.71429	1	0.000437	0.000276	0.000284	0.008	0.006	0.006
		2	0.000697	0.000422	0.000441	0.01	0.009	0.009

		3	0.00052	0.000341	0.000345	0.01	0.009	0.007
		4	0.000512	0.000314	0.000327	0.009	0.007	0.007
		5	0.00042	0.000258	0.000272	0.008	0.007	0.006
		6	0.000481	0.000289	0.000304	0.01	0.007	0.007
		7	0.000422	0.000283	0.000316	0.009	0.007	0.006
		8	0.000421	0.00029	0.000302	0.007	0.006	0.006
		9	0.000536	0.000344	0.000382	0.009	0.008	0.008
		10	0.000486	0.000288	0.000305	0.008	0.006	0.006
		11	0.000575	0.000371	0.000369	0.01	0.009	0.01
		12	0.000427	0.000273	0.000283	0.01	0.009	0.007
		13	0.000416	0.000239	0.000259	0.008	0.006	0.006
	0.83333	1	0.000348	0.000221	0.000216	0.007	0.006	0.005
		2	0.000534	0.000342	0.000342	0.01	0.008	0.008
		3	0.000366	0.000245	0.000229	0.01	0.008	0.006
		4	0.000366	0.000234	0.000233	0.008	0.006	0.006
		5	0.000314	0.000196	0.000194	0.008	0.006	0.005
		6	0.000336	0.000208	0.000206	0.008	0.006	0.006
		7	0.000294	0.000205	0.000216	0.007	0.006	0.005
		8	0.000326	0.00023	0.000229	0.006	0.005	0.005
		9	0.000404	0.000257	0.000271	0.008	0.008	0.007
		10	0.000439	0.000258	0.000268	0.008	0.006	0.006
		11	0.000314	0.000206	0.000194	0.008	0.006	0.006
		12	0.000313	0.0002	0.000191	0.01	0.008	0.007
		13	0.000301	0.000185	0.000183	0.007	0.006	0.005
	1	1	0.000239	0.000154	0.000153	0.006	0.005	0.004
		2	0.000419	0.000281	0.000287	0.01	0.007	0.007
		3	0.000256	0.000176	0.00017	0.009	0.007	0.006
		4	0.000284	0.000189	0.000194	0.007	0.006	0.006
		5	0.000224	0.000146	0.000149	0.007	0.006	0.005
		6	0.000234	0.000153	0.000155	0.007	0.006	0.005
		7	0.000207	0.000149	0.000161	0.006	0.005	0.005
		8	0.000264	0.000189	0.000191	0.006	0.005	0.005
		9	0.000287	0.00019	0.000204	0.008	0.007	0.007
		10	0.000409	0.000241	0.000252	0.008	0.006	0.006
		11	0.000206	0.000137	0.000134	0.006	0.005	0.005
		12	0.000227	0.00015	0.000147	0.009	0.007	0.006
		13	0.000239	0.000156	0.000156	0.007	0.006	0.005
	1.14	1	0.00023	0.000146	0.000148	0.006	0.005	0.004

		2	0.000405	0.000271	0.000274	0.009	0.007	0.007
		3	0.000262	0.000175	0.000171	0.009	0.007	0.006
		4	0.000281	0.000186	0.000192	0.008	0.006	0.006
		5	0.000248	0.000157	0.000163	0.009	0.006	0.006
		6	0.000201	0.00013	0.000135	0.007	0.005	0.005
		7	0.000199	0.000143	0.000149	0.006	0.005	0.005
		8	0.000275	0.000195	0.000192	0.007	0.006	0.006
		9	0.000258	0.000169	0.000172	0.009	0.007	0.007
		10	0.000437	0.000256	0.000281	0.009	0.007	0.007
		11	0.000206	0.000135	0.000133	0.007	0.005	0.005
		12	0.000194	0.000125	0.000122	0.009	0.007	0.006
		13	0.000223	0.000143	0.000145	0.007	0.006	0.005
MR_no	1.19	1	0.0007	0.00026	0.00025	0.0284	0.02245	0.02089
		2	0.00049	0.00047	0.00046	0.03914	0.04014	0.02369
		3	0.00065	0.00027	0.00026	0.02352	0.02803	0.01925
		4	0.00042	0.00029	0.00029	0.02891	0.03076	0.0163
		5	0.00056	0.00024	0.00024	0.03249	0.02561	0.02891
		6	0.00051	0.00022	0.00021	0.02399	0.0183	0.01674
		7	0.00075	0.00028	0.00027	0.03371	0.02898	0.01808
		8	0.00043	0.00032	0.00029	0.03247	0.02064	0.01967
		9	0.00036	0.00025	0.00024	0.03248	0.0304	0.02411
		10	0.00064	0.00047	0.00052	0.04553	0.02384	0.03237
		11	0.0006	0.00022	0.00021	0.03159	0.01826	0.01891
		12	0.00063	0.00022	0.00021	0.0268	0.02569	0.0158
		13	0.00048	0.00021	0.0002	0.02786	0.03583	0.0188
MR	1.18	1	0.000709	0.000419	0.000258	0.000113	0.01678	0.000102
		2	0.000544	0.000801	0.00047	0.000801	0.015453	0.015453
		3	0.000663	0.000451	0.000269	0.000098	0.019021	0.000088
		4	0.000447	0.000492	0.000299	0.000054	0.014243	0.000049
		5	0.000575	0.000409	0.000248	0.000093	0.018508	0.000084
		6	0.000524	0.00035	0.000219	0.000098	0.015384	0.000088
		7	0.00077	0.000414	0.000277	0.000151	0.016262	0.000137
		8	0.000451	0.000544	0.000298	0.000054	0.013988	0.000049
		9	0.000382	0.000419	0.000244	0.000054	0.014658	0.000049
		10	0.000691	0.000927	0.000546	0.000059	0.014664	0.000053
		11	0.000605	0.000352	0.000217	0.000093	0.018067	0.000084
		12	0.000634	0.000335	0.000213	0.000113	0.016947	0.000102
		13	0.000491	0.000375	0.000213	0.000025	0.014109	0.000023

Neutron Diffraction	N/A	1	0.002	0.002	0.002	0.003	0.004	0.004
		2	0.002	0.002	0.002	0.003	0.004	0.004
		3	0.0019	0.002	0.002	0.003	0.004	0.004
		4	0.0019	0.002	0.002	0.003	0.003	0.004
		5	0.0019	0.002	0.002	0.003	0.004	0.004
		6	0.002	0.002	0.002	0.003	0.004	0.004
		7	0.002	0.002	0.003	0.003	0.004	0.004
		8	0.002	0.002	0.002	0.003	0.004	0.004
		9	0.0019	0.002	0.002	0.003	0.003	0.004
		10	0.002	0.002	0.003	0.003	0.004	0.004
		11	0.002	0.002	0.002	0.003	0.004	0.004
		12	0.002	0.002	0.002	0.003	0.004	0.004
		13	0.002	0.002	0.002	0.003	0.004	0.004
		14	0.0019	0.002	0.002	0.003	0.004	0.003

(ii) x, 1.5-y, 1/2+z

Method	Resolution [Å $^{-1}$]	nr set	H1...O3 ls error	H2...O2 ls error	H3...O2 ls error	O1...O3 ls error	O3...O2 ls error	O3...O2 ^{ii} ls error
IAM	0.625	1	0.01685	0.01887	0.01787	0.00077	0.00207	0.00404
		2	0.01872	0.02135	0.01909	0.00084	0.0013	0.00219
		3	0.01955	0.02197	0.01928	0.00077	0.00168	0.00325
		4	0.01661	0.01924	0.01739	0.00079	0.00146	0.0027
		5	0.01711	0.02001	0.01823	0.00075	0.00161	0.00308
		6	0.01888	0.01982	0.01915	0.00082	0.00181	0.00354
		7	0.01675	0.02112	0.01791	0.00082	0.003	0.00612
		8	0.01498	0.01926	0.0161	0.00074	0.00113	0.00177
		9	0.01562	0.0194	0.01695	0.00078	0.00109	0.00166
		10	0.01759	0.01994	0.01762	0.00082	0.00109	0.00171
		11	0.01839	0.0137	0.01317	0.00067	0.00146	0.0028
		12	0.01935	0.01541	0.0151	0.0007	0.00202	0.00403
		13	0.01531	0.01515	0.01431	0.00072	0.00175	0.00346
	0.71429	1	0.01474	0.01535	0.0152	0.00064	0.00188	0.00377
		2	0.01675	0.01455	0.01445	0.00071	0.00094	0.0015
		3	0.01827	0.01452	0.01421	0.00061	0.00119	0.00216
		4	0.01573	0.01337	0.01282	0.00063	0.00098	0.00174
		5	0.01617	0.01312	0.01257	0.00058	0.00105	0.00195
		6	0.016	0.01252	0.01299	0.00061	0.0012	0.00224
		7	0.01654	0.0138	0.01223	0.00065	0.00232	0.00475
		8	0.01307	0.01287	0.01153	0.00059	0.00092	0.00154
		9	0.01495	0.01344	0.01259	0.00061	0.00089	0.00148
		10	0.01528	0.01297	0.01265	0.00066	0.0009	0.0015
		11	0.01811	0.01255	0.01263	0.00057	0.00148	0.0029
		12	0.01748	0.01615	0.01503	0.00057	0.00176	0.00359
		13	0.01398	0.01377	0.01287	0.00057	0.00127	0.00253
	0.83333	1	0.01361	0.01045	0.01028	0.00051	0.00119	0.00222
		2	0.01535	0.01288	0.01237	0.00053	0.00078	0.0013
		3	0.0168	0.01324	0.01298	0.00048	0.00106	0.00199
		4	0.01416	0.01179	0.01117	0.00047	0.00084	0.00155
		5	0.01515	0.0121	0.01161	0.00045	0.00094	0.00181
		6	0.01484	0.01148	0.01172	0.00048	0.00109	0.00209

		7	0.0147	0.0116	0.0105	0.00052	0.00171	0.00348
		8	0.01164	0.01141	0.01007	0.00046	0.00078	0.00137
		9	0.01375	0.01269	0.01125	0.00045	0.00056	0.00079
		10	0.01404	0.01152	0.01154	0.00057	0.0008	0.00137
		11	0.01594	0.01106	0.01144	0.00045	0.00134	0.00268
		12	0.01591	0.01476	0.01373	0.00046	0.00132	0.00266
		13	0.01254	0.01211	0.01133	0.00042	0.00081	0.00154
	1	1	0.01101	0.00834	0.00834	0.00041	0.00096	0.00181
		2	0.01344	0.01108	0.01065	0.00043	0.00064	0.0011
		3	0.01365	0.01072	0.0107	0.00037	0.00086	0.00164
		4	0.0121	0.00993	0.00933	0.00036	0.00047	0.00073
		5	0.01257	0.01006	0.00959	0.00035	0.00077	0.00151
		6	0.01244	0.00949	0.0098	0.00037	0.0009	0.00175
		7	0.01191	0.00945	0.00845	0.00045	0.00111	0.00216
		8	0.00953	0.00948	0.00832	0.00035	0.00045	0.00065
		9	0.01158	0.01099	0.00955	0.00033	0.0004	0.00062
		10	0.01233	0.0102	0.01018	0.00049	0.00068	0.0012
		11	0.01304	0.00895	0.00954	0.00036	0.00111	0.00224
		12	0.01309	0.0122	0.01116	0.00037	0.00084	0.00159
		13	0.01057	0.01036	0.00967	0.00032	0.00067	0.00132
	1.14	1	0.00997	0.00745	0.00747	0.00037	0.00085	0.00161
		2	0.01224	0.01034	0.00982	0.00045	0.00062	0.00103
		3	0.01232	0.00994	0.00955	0.00034	0.00077	0.00148
		4	0.01117	0.00949	0.00873	0.00032	0.00042	0.00066
		5	0.01191	0.00912	0.0088	0.00032	0.00075	0.0015
		6	0.01085	0.0081	0.00849	0.00033	0.00078	0.00153
		7	0.0105	0.00863	0.00779	0.00042	0.00108	0.00213
		8	0.00866	0.00875	0.00748	0.00032	0.0004	0.00059
		9	0.01051	0.00975	0.00868	0.00027	0.00035	0.00057
		10	0.01327	0.01082	0.01062	0.00053	0.00074	0.00128
		11	0.01251	0.0088	0.00913	0.00035	0.00106	0.00215
		12	0.01134	0.01068	0.01004	0.00033	0.00072	0.00136
		13	0.00963	0.00937	0.00889	0.00028	0.00059	0.00119
UBDB	0.625	1	0.01013	0.0093	0.0084	0.00039	0.00043	0.00043
		2	0.01196	0.01037	0.0099	0.00048	0.00052	0.00053
		3	0.01146	0.01014	0.00919	0.0004	0.00044	0.00045
		4	0.01039	0.0092	0.00872	0.00042	0.00046	0.00045

	5	0.00992	0.00901	0.0084	0.00037	0.0004	0.00041
	6	0.01107	0.00952	0.0091	0.00043	0.00043	0.00046
	7	0.01387	0.01256	0.01026	0.00048	0.0006	0.00056
	8	0.00965	0.0095	0.00834	0.00041	0.00048	0.00047
	9	0.01054	0.00986	0.0092	0.00044	0.0005	0.00051
	10	0.01063	0.00967	0.00906	0.00045	0.00046	0.00047
	11	0.01449	0.01002	0.01067	0.00045	0.00045	0.00049
	12	0.01167	0.01038	0.00955	0.00034	0.00042	0.0004
	13	0.01038	0.0101	0.00912	0.00042	0.00049	0.00047
0.71429	1	0.00962	0.00816	0.00752	0.00034	0.00034	0.00035
	2	0.01249	0.01033	0.01024	0.00047	0.00047	0.00048
	3	0.01296	0.01073	0.0103	0.00039	0.0004	0.0004
	4	0.0107	0.00888	0.00862	0.00039	0.00038	0.00038
	5	0.01003	0.00846	0.00793	0.00032	0.00032	0.00033
	6	0.01115	0.00916	0.00894	0.00037	0.00034	0.00038
	7	0.01255	0.0104	0.00873	0.00038	0.00043	0.00041
	8	0.00896	0.00852	0.00739	0.00036	0.00039	0.00039
	9	0.01039	0.00915	0.00863	0.00038	0.0004	0.00042
	10	0.00992	0.00867	0.00822	0.00039	0.00038	0.00039
	11	0.01145	0.00942	0.00893	0.00035	0.00034	0.00036
	12	0.01369	0.01137	0.01075	0.00033	0.00034	0.00033
	13	0.00979	0.00899	0.00827	0.00034	0.00035	0.00036
0.83333	1	0.00883	0.00743	0.00681	0.00028	0.00028	0.00028
	2	0.01141	0.00947	0.00908	0.00037	0.00038	0.00038
	3	0.01094	0.00934	0.00855	0.00028	0.00029	0.00028
	4	0.00916	0.0077	0.00739	0.00028	0.00028	0.00029
	5	0.00961	0.00806	0.00752	0.00025	0.00025	0.00025
	6	0.00962	0.00816	0.00763	0.00027	0.00026	0.00027
	7	0.01051	0.00846	0.00727	0.00026	0.0003	0.00029
	8	0.00834	0.0077	0.00664	0.00029	0.00032	0.00031
	9	0.00974	0.00877	0.00788	0.00029	0.00031	0.00031
	10	0.00921	0.00786	0.00749	0.00034	0.00033	0.00035
	11	0.00956	0.00798	0.00734	0.00026	0.00025	0.00025
	12	0.01234	0.01079	0.00943	0.00024	0.00026	0.00024
	13	0.00875	0.00804	0.00724	0.00025	0.00027	0.00026
1	1	0.00739	0.00612	0.00566	0.00021	0.0002	0.00021
	2	0.01074	0.00883	0.00842	0.00031	0.00033	0.00033

		3	0.00984	0.00813	0.00757	0.00021	0.00021	0.00021
		4	0.00858	0.0071	0.00679	0.00023	0.00023	0.00024
		5	0.00906	0.00738	0.00698	0.00019	0.00018	0.00019
		6	0.00876	0.00722	0.00682	0.0002	0.00019	0.0002
		7	0.00916	0.0073	0.00635	0.0002	0.00022	0.00022
		8	0.00751	0.00689	0.00602	0.00024	0.00026	0.00025
		9	0.00897	0.00824	0.00724	0.00022	0.00022	0.00024
		10	0.00882	0.00752	0.0072	0.00032	0.0003	0.00032
		11	0.00843	0.00694	0.00646	0.00019	0.00018	0.00018
		12	0.01153	0.0099	0.00861	0.00019	0.00019	0.00018
		13	0.00817	0.00758	0.00671	0.00021	0.00021	0.00021
	1.14	1	0.00714	0.00581	0.0054	0.00019	0.00018	0.00019
		2	0.01018	0.0087	0.00792	0.0003	0.00032	0.00031
		3	0.00999	0.00832	0.00752	0.0002	0.00021	0.0002
		4	0.00844	0.00719	0.00673	0.00021	0.00022	0.00023
		5	0.00997	0.00761	0.00728	0.00019	0.00019	0.00019
		6	0.00781	0.00638	0.00622	0.00017	0.00017	0.00017
		7	0.00803	0.00675	0.00599	0.00017	0.00019	0.00018
		8	0.00758	0.00686	0.00588	0.00023	0.00024	0.00023
		9	0.00881	0.00783	0.00706	0.00019	0.00019	0.0002
		10	0.00977	0.00813	0.00794	0.00033	0.0003	0.00033
		11	0.00817	0.00689	0.00631	0.00018	0.00017	0.00018
		12	0.01106	0.00951	0.00827	0.00016	0.00016	0.00015
		13	0.00796	0.00733	0.00655	0.00019	0.00019	0.00019
ELMAM2	0.625	1	0.00932	0.00896	0.00753	0.00037	0.00041	0.00041
		2	0.01122	0.00946	0.00858	0.00045	0.00049	0.00049
		3	0.0107	0.00954	0.00816	0.00037	0.00042	0.00042
		4	0.01002	0.0088	0.00781	0.00042	0.00045	0.00044
		5	0.00931	0.0087	0.00755	0.00035	0.00039	0.00039
		6	0.01045	0.00947	0.00818	0.00041	0.00042	0.00045
		7	0.01334	0.01185	0.00925	0.00046	0.00057	0.00053
		8	0.00931	0.00873	0.00736	0.0004	0.00046	0.00045
		9	0.01014	0.00921	0.00803	0.00043	0.00048	0.00049
		10	0.01054	0.00946	0.00838	0.00045	0.00045	0.00047
		11	0.01398	0.01034	0.01016	0.00045	0.00045	0.00049
		12	0.01137	0.01017	0.00876	0.00033	0.0004	0.00038
		13	0.01011	0.00937	0.008	0.00041	0.00047	0.00045

0.71429	1	0.00901	0.00799	0.00683	0.00032	0.00032	0.00033
	2	0.01219	0.00979	0.00926	0.00046	0.00046	0.00047
	3	0.01268	0.0103	0.00932	0.00037	0.00038	0.00038
	4	0.01056	0.00858	0.00786	0.00038	0.00037	0.00038
	5	0.00994	0.00837	0.00752	0.00031	0.00031	0.00032
	6	0.01082	0.00915	0.00814	0.00036	0.00033	0.00037
	7	0.01224	0.00979	0.0079	0.00035	0.0004	0.00039
	8	0.00859	0.00783	0.00649	0.00034	0.00037	0.00037
	9	0.01017	0.00868	0.00766	0.00037	0.00038	0.0004
	10	0.00994	0.00852	0.00759	0.00038	0.00037	0.00039
	11	0.01083	0.00948	0.00828	0.00034	0.00033	0.00035
	12	0.01361	0.0107	0.0099	0.00032	0.00033	0.00032
	13	0.00954	0.00846	0.00739	0.00033	0.00034	0.00034
0.83333	1	0.00849	0.00739	0.0062	0.00027	0.00027	0.00028
	2	0.01141	0.00901	0.00832	0.00037	0.00038	0.00038
	3	0.0118	0.00954	0.00851	0.00028	0.0003	0.00029
	4	0.00935	0.00753	0.00691	0.00028	0.00029	0.00029
	5	0.00958	0.00806	0.007	0.00024	0.00025	0.00025
	6	0.00985	0.0085	0.00721	0.00027	0.00026	0.00028
	7	0.0103	0.00794	0.00653	0.00025	0.00029	0.00027
	8	0.00823	0.00719	0.0059	0.00028	0.00031	0.0003
	9	0.01003	0.00846	0.00723	0.00029	0.0003	0.00031
	10	0.00951	0.00797	0.00706	0.00035	0.00033	0.00035
	11	0.00938	0.00827	0.00685	0.00026	0.00025	0.00025
	12	0.01288	0.01031	0.00887	0.00024	0.00025	0.00024
	13	0.00884	0.0077	0.00659	0.00025	0.00026	0.00026
1	1	0.00617	0.00574	0.00521	0.00031	0.0005	0.00094
	2	0.01036	0.00929	0.00909	0.00039	0.00048	0.0008
	3	0.00912	0.00819	0.00761	0.00032	0.00053	0.001
	4	0.00798	0.00697	0.00678	0.00033	0.00043	0.00078
	5	0.00802	0.00715	0.00675	0.00031	0.00046	0.00088
	6	0.00784	0.00684	0.00667	0.00031	0.00052	0.00098
	7	0.00737	0.00683	0.00578	0.00031	0.00045	0.00083
	8	0.00671	0.00685	0.00595	0.00033	0.00039	0.00068
	9	0.00876	0.00832	0.00759	0.00033	0.00041	0.00073
	10	0.00794	0.00759	0.0072	0.00037	0.00041	0.00069
	11	0.0068	0.00606	0.00567	0.0003	0.00059	0.00113

		12	0.00968	0.00917	0.00819	0.00031	0.00051	0.00097
		13	0.00729	0.00746	0.00668	0.00032	0.00043	0.00078
	1.14	1	0.00724	0.0061	0.00509	0.00019	0.00018	0.00019
		2	0.01033	0.00827	0.00736	0.0003	0.00032	0.00032
		3	0.00976	0.00816	0.00685	0.0002	0.00021	0.0002
		4	0.00887	0.00709	0.0064	0.00022	0.00022	0.00023
		5	0.00977	0.00779	0.00686	0.00019	0.00019	0.00019
		6	0.00814	0.00674	0.00592	0.00017	0.00017	0.00017
		7	0.00773	0.00651	0.00535	0.00017	0.00019	0.00018
		8	0.0077	0.00651	0.00532	0.00023	0.00024	0.00023
		9	0.00934	0.00781	0.0066	0.0002	0.0002	0.0002
		10	0.01023	0.00817	0.00754	0.00033	0.00031	0.00033
		11	0.00826	0.00736	0.00593	0.00018	0.00018	0.00018
		12	0.01213	0.00942	0.00794	0.00016	0.00016	0.00016
		13	0.00816	0.0071	0.00602	0.00019	0.00019	0.00019
GID	0.625	1	0.00932	0.00896	0.00753	0.00037	0.00041	0.00041
		2	0.01122	0.00946	0.00858	0.00045	0.00049	0.00049
		3	0.0107	0.00954	0.00816	0.00037	0.00042	0.00042
		4	0.01002	0.0088	0.00781	0.00042	0.00045	0.00044
		5	0.00931	0.0087	0.00755	0.00035	0.00039	0.00039
		6	0.01045	0.00947	0.00818	0.00041	0.00042	0.00045
		7	0.01334	0.01185	0.00925	0.00046	0.00057	0.00053
		8	0.00931	0.00873	0.00736	0.0004	0.00046	0.00045
		9	0.01014	0.00921	0.00803	0.00043	0.00048	0.00049
		10	0.01054	0.00946	0.00838	0.00045	0.00045	0.00047
		11	0.01398	0.01034	0.01016	0.00045	0.00045	0.00049
		12	0.01137	0.01017	0.00876	0.00033	0.0004	0.00038
		13	0.01011	0.00937	0.008	0.00041	0.00047	0.00045
	0.71429	1	0.00901	0.00799	0.00683	0.00032	0.00032	0.00033
		2	0.01219	0.00979	0.00926	0.00046	0.00046	0.00047
		3	0.01268	0.0103	0.00932	0.00037	0.00038	0.00038
		4	0.01056	0.00858	0.00786	0.00038	0.00037	0.00038
		5	0.00994	0.00837	0.00752	0.00031	0.00031	0.00032
		6	0.01082	0.00915	0.00814	0.00036	0.00033	0.00037
		7	0.01224	0.00979	0.0079	0.00035	0.0004	0.00039
		8	0.00859	0.00783	0.00649	0.00034	0.00037	0.00037
		9	0.01017	0.00868	0.00766	0.00037	0.00038	0.0004

		10	0.00994	0.00852	0.00759	0.00038	0.00037	0.00039
		11	0.01083	0.00948	0.00828	0.00034	0.00033	0.00035
		12	0.01361	0.0107	0.0099	0.00032	0.00033	0.00032
		13	0.00954	0.00846	0.00739	0.00033	0.00034	0.00034
	0.83333	1	0.00849	0.00739	0.0062	0.00027	0.00027	0.00028
		2	0.01141	0.00901	0.00832	0.00037	0.00038	0.00038
		3	0.0118	0.00954	0.00851	0.00028	0.0003	0.00029
		4	0.00935	0.00753	0.00691	0.00028	0.00029	0.00029
		5	0.00958	0.00806	0.007	0.00024	0.00025	0.00025
		6	0.00985	0.0085	0.00721	0.00027	0.00026	0.00028
		7	0.0103	0.00794	0.00653	0.00025	0.00029	0.00027
		8	0.00823	0.00719	0.0059	0.00028	0.00031	0.0003
		9	0.01003	0.00846	0.00723	0.00029	0.0003	0.00031
		10	0.00951	0.00797	0.00706	0.00035	0.00033	0.00035
		11	0.00938	0.00827	0.00685	0.00026	0.00025	0.00025
		12	0.01288	0.01031	0.00887	0.00024	0.00025	0.00024
		13	0.00884	0.0077	0.00659	0.00025	0.00026	0.00026
	1	1	0.00727	0.00622	0.00521	0.00021	0.0002	0.00021
		2	0.01071	0.00834	0.00769	0.00031	0.00033	0.00032
		3	0.01014	0.00816	0.00702	0.00021	0.00021	0.00021
		4	0.00882	0.00698	0.00638	0.00023	0.00023	0.00024
		5	0.00908	0.00749	0.00652	0.00019	0.00019	0.00019
		6	0.00907	0.0076	0.00646	0.0002	0.0002	0.00021
		7	0.00905	0.0069	0.0057	0.00019	0.00022	0.00021
		8	0.00745	0.00648	0.00537	0.00023	0.00025	0.00024
		9	0.00931	0.00798	0.00662	0.00022	0.00022	0.00023
		10	0.00922	0.00768	0.00679	0.00032	0.0003	0.00032
		11	0.00838	0.00735	0.00601	0.00019	0.00018	0.00018
		12	0.01231	0.00965	0.00818	0.00019	0.00019	0.00018
		13	0.00836	0.0073	0.00616	0.00021	0.00021	0.00021
	1.14	1	0.00724	0.0061	0.00509	0.00019	0.00018	0.00019
		2	0.01033	0.00827	0.00736	0.0003	0.00032	0.00032
		3	0.00976	0.00816	0.00685	0.0002	0.00021	0.0002
		4	0.00887	0.00709	0.0064	0.00022	0.00022	0.00023
		5	0.00977	0.00779	0.00686	0.00019	0.00019	0.00019
		6	0.00814	0.00674	0.00592	0.00017	0.00017	0.00017
		7	0.00773	0.00651	0.00535	0.00017	0.00019	0.00018

		8	0.0077	0.00651	0.00532	0.00023	0.00024	0.00023
		9	0.00934	0.00781	0.0066	0.0002	0.0002	0.0002
		10	0.01023	0.00817	0.00754	0.00033	0.00031	0.00033
		11	0.00826	0.00736	0.00593	0.00018	0.00018	0.00018
		12	0.01213	0.00942	0.00794	0.00016	0.00016	0.00016
		13	0.00816	0.0071	0.00602	0.00019	0.00019	0.00019
HAR(B3LYP)	0.625	1	0.00875	0.006776	0.007842	0.000282	0.000333	0.000314
		2	0.010441	0.008408	0.009138	0.000387	0.000431	0.000407
		3	0.009551	0.006961	0.007833	0.000283	0.000334	0.000318
		4	0.008822	0.006861	0.007453	0.000306	0.000345	0.000325
		5	0.008293	0.006358	0.007524	0.000264	0.000316	0.00029
		6	0.00914	0.006941	0.00779	0.000306	0.000323	0.000326
		7	0.00989	0.006657	0.01	0.000288	0.008238	0.000335
		8	0.007568	0.006033	0.006567	0.000262	0.000324	0.000302
		9	0.010139	0.008469	0.007788	0.000349	0.00041	0.000396
		10	0.008845	0.007279	0.006506	0.000301	0.000328	0.000312
		11	0.014274	0.01106	0.01011	0.000378	0.000431	0.000426
		12	0.009896	0.007626	0.008772	0.000262	0.00033	0.000303
		13	0.008905	0.007111	0.00743	0.000293	0.000342	0.00032
	0.71429	1	0.007857	0.005758	0.006343	0.000239	0.000242	0.000247
		2	0.01092	0.008588	0.009296	0.000386	0.000385	0.000376
		3	0.011225	0.007266	0.008961	0.000295	0.00031	0.000299
		4	0.008717	0.006547	0.007294	0.000283	0.000277	0.000279
		5	0.00808	0.005867	0.006579	0.000227	0.000232	0.000236
		6	0.00935	0.00725	0.007317	0.000267	0.000254	0.000271
		7	0.008172	0.005465	0.008	0.000226	0.006531	0.000245
		8	0.006582	0.00525	0.005561	0.000227	0.000251	0.000243
		9	0.008637	0.006895	0.007674	0.000285	0.000302	0.000305
		10	0.007933	0.005883	0.006309	0.000265	0.000261	0.000263
		11	0.013871	0.009484	0.009999	0.000324	0.000333	0.000342
		12	0.011155	0.007424	0.008603	0.000256	0.000267	0.000262
		13	0.007782	0.006041	0.006541	0.000231	0.000234	0.000236
	0.83333	1	0.007134	0.00522	0.005775	0.000195	0.000199	0.000201
		2	0.009586	0.007552	0.008226	0.000294	0.000311	0.000305
		3	0.009536	0.00645	0.007778	0.000211	0.000225	0.000215
		4	0.007231	0.005592	0.006202	0.000201	0.000207	0.00021
		5	0.007485	0.005591	0.006138	0.000178	0.000183	0.000183

		6	0.007633	0.005825	0.006365	0.000189	0.000188	0.000193
		7	0.006708	0.004674	0.007	0.000163	0.005538	0.000178
		8	0.006155	0.004774	0.00519	0.000182	0.000205	0.000195
		9	0.008114	0.006493	0.007534	0.00022	0.000234	0.000238
		10	0.007615	0.005728	0.006067	0.000244	0.000236	0.000242
		11	0.007446	0.005824	0.006123	0.000182	0.000184	0.000185
		12	0.009861	0.006756	0.008007	0.00019	0.0002	0.00019
		13	0.006869	0.005319	0.005917	0.000173	0.000182	0.000178
	1	1	0.005833	0.004232	0.004651	0.000143	0.000141	0.000145
		2	0.009161	0.007029	0.007575	0.000252	0.000263	0.000257
		3	0.00858	0.005797	0.006767	0.000158	0.000163	0.000159
		4	0.007041	0.005385	0.005961	0.000169	0.000171	0.000176
		5	0.007167	0.005295	0.005559	0.000138	0.000138	0.000141
		6	0.007158	0.005311	0.005814	0.000144	0.000141	0.000145
		7	0.005888	0.004848	0.006	0.000125	0.004143	0.000134
		8	0.005897	0.004665	0.005032	0.00016	0.000175	0.000167
		9	0.00767	0.006196	0.007264	0.000176	0.000177	0.000184
		10	0.007859	0.005797	0.006148	0.000239	0.000223	0.000235
		11	0.00637	0.004989	0.005106	0.000127	0.000125	0.000128
		12	0.00918	0.006223	0.007371	0.000147	0.000148	0.000146
		13	0.006837	0.005277	0.005902	0.00015	0.000153	0.000152
	1.14	1	0.006312	0.004515	0.00498	0.00014	0.000136	0.000141
		2	0.009111	0.006942	0.00775	0.000244	0.000257	0.000249
		3	0.009214	0.007032	0.006083	0.000164	0.000167	0.000164
		4	0.007723	0.005667	0.006425	0.000168	0.00017	0.000176
		5	0.009076	0.006389	0.00652	0.000156	0.000155	0.000157
		6	0.0067	0.004963	0.00535	0.000124	0.000124	0.000126
		7	0.006077	0.004569	0.005377	0.00012	0.000136	0.000128
		8	0.006602	0.005022	0.005549	0.00017	0.000185	0.000175
		9	0.0084	0.00667	0.007347	0.000161	0.00016	0.000164
		10	0.008972	0.006578	0.006911	0.000258	0.000238	0.000254
		11	0.006408	0.00505	0.005334	0.000127	0.000124	0.000128
		12	0.00933	0.006308	0.00757	0.000127	0.000127	0.000125
		13	0.006871	0.005357	0.005935	0.000141	0.000143	0.000143
HAR(BLYP)	0.625	1	0.009217	0.006943	0.007953	0.00029	0.000342	0.000323
		2	0.011187	0.008788	0.009276	0.000402	0.000448	0.000424
		3	0.010252	0.007273	0.008019	0.000294	0.000348	0.000332

	4	0.009361	0.007119	0.007585	0.000317	0.000358	0.000339
	5	0.008912	0.006622	0.007755	0.000277	0.000331	0.000304
	6	0.009709	0.007293	0.007997	0.00032	0.000337	0.000341
	7	0.010592	0.006792	0.008314	0.000296	0.000401	0.000343
	8	0.008051	0.00629	0.006678	0.000271	0.000335	0.000313
	9	0.010139	0.007788	0.008469	0.000349	0.00041	0.000396
	10	0.00924	0.00679	0.007352	0.000309	0.000338	0.000322
	11	0.014667	0.010061	0.011016	0.000379	0.000432	0.000427
	12	0.010428	0.007768	0.008818	0.000267	0.000338	0.000312
	13	0.009142	0.007227	0.007315	0.000295	0.000345	0.000323
0.71429	1	0.008185	0.005807	0.006387	0.000243	0.000245	0.00025
	2	0.011543	0.008788	0.009392	0.000395	0.000392	0.000385
	3	0.011763	0.00742	0.009073	0.000301	0.000316	0.000305
	4	0.009086	0.006744	0.007435	0.000291	0.000284	0.000286
	5	0.008417	0.005987	0.006683	0.000232	0.000237	0.000241
	6	0.009664	0.007438	0.007326	0.000271	0.000258	0.000275
	7	0.008508	0.00555	0.006555	0.00023	0.000265	0.000248
	8	0.006989	0.005443	0.005665	0.000234	0.000259	0.000251
	9	0.009002	0.007051	0.007759	0.00029	0.000307	0.000311
	10	0.008364	0.006072	0.006403	0.000273	0.000267	0.00027
	11	0.014146	0.009391	0.009849	0.000322	0.00033	0.000339
	12	0.011361	0.007455	0.008673	0.000258	0.00027	0.000264
	13	0.007978	0.006125	0.006516	0.000233	0.000235	0.000238
0.83333	1	0.007317	0.005199	0.005762	0.000196	0.000199	0.000202
	2	0.010079	0.007653	0.008323	0.0003	0.000316	0.000311
	3	0.009926	0.006521	0.007833	0.000214	0.000229	0.000219
	4	0.007552	0.005725	0.006336	0.000206	0.000213	0.000216
	5	0.007713	0.005627	0.00616	0.00018	0.000185	0.000186
	6	0.007881	0.005899	0.006419	0.000193	0.000191	0.000197
	7	0.00693	0.00468	0.005528	0.000164	0.000192	0.000179
	8	0.006433	0.004864	0.005258	0.000186	0.00021	0.000199
	9	0.008421	0.006533	0.007576	0.000223	0.000237	0.000241
	10	0.007995	0.005866	0.006155	0.00025	0.000241	0.000248
	11	0.007659	0.005756	0.006135	0.000184	0.000185	0.000186
	12	0.010052	0.006744	0.007957	0.000192	0.000201	0.000192
	13	0.007028	0.005369	0.005909	0.000174	0.000183	0.00018
1	1	0.005955	0.004216	0.004628	0.000144	0.000141	0.000146

		2	0.009578	0.007082	0.007639	0.000255	0.000266	0.00026
		3	0.00886	0.005834	0.006771	0.00016	0.000165	0.000161
		4	0.007301	0.005469	0.006042	0.000172	0.000174	0.00018
		5	0.007317	0.005282	0.005518	0.000139	0.000138	0.000141
		6	0.007322	0.005334	0.005803	0.000145	0.000142	0.000147
		7	0.006041	0.004809	0.004125	0.000125	0.000141	0.000134
		8	0.006082	0.004706	0.005051	0.000162	0.000177	0.000169
		9	0.007941	0.006231	0.007305	0.000177	0.000178	0.000186
		10	0.008119	0.005918	0.006204	0.000242	0.000225	0.000238
		11	0.00648	0.004891	0.005057	0.000127	0.000126	0.000128
		12	0.009287	0.006183	0.007267	0.000147	0.000148	0.000146
		13	0.006978	0.005312	0.00588	0.000151	0.000153	0.000152
	1.14	1	0.006486	0.004518	0.004967	0.000141	0.000138	0.000142
		2	0.009504	0.007017	0.007817	0.000247	0.00026	0.000253
		3	0.00949	0.006129	0.00704	0.000165	0.000168	0.000165
		4	0.00806	0.00576	0.00651	0.000171	0.000173	0.000179
		5	0.009236	0.006355	0.00647	0.000156	0.000155	0.000157
		6	0.006887	0.004959	0.005326	0.000126	0.000126	0.000127
		7	0.006223	0.00455	0.005346	0.00012	0.000136	0.000128
		8	0.006756	0.005061	0.005544	0.000172	0.000187	0.000176
		9	0.008714	0.006672	0.007364	0.000162	0.000161	0.000166
		10	0.009315	0.006636	0.006957	0.000262	0.000241	0.000257
		11	0.006539	0.004938	0.00527	0.000127	0.000124	0.000128
		12	0.009423	0.006273	0.00743	0.000127	0.000127	0.000125
		13	0.006998	0.005383	0.005892	0.000142	0.000143	0.000144
MR_no	1.19	1	0.02884	0.02282	0.02757	0.00035	0.00030	0.00035
		2	0.03916	0.04423	0.02338	0.00056	0.00061	0.00057
		3	0.02358	0.03208	0.02279	0.00034	0.00032	0.00035
		4	0.0295	0.03227	0.02003	0.00035	0.00033	0.00036
		5	0.03271	0.02673	0.03060	0.00031	0.00028	0.00032
		6	0.02425	0.01789	0.02352	0.00027	0.00025	0.00028
		7	0.0334	0.02930	0.01637	0.00035	0.00032	0.00037
		8	0.03256	0.02329	0.01955	0.00038	0.00038	0.00037
		9	0.03321	0.03076	0.02254	0.00030	0.00028	0.00030
		10	0.04573	0.02384	0.03925	0.00071	0.00058	0.00073
		11	0.03202	0.01925	0.02575	0.00027	0.00024	0.00028
		12	0.0272	0.02721	0.01888	0.00029	0.00026	0.00030

		13	0.02805	0.03814	0.02127	0.00027	0.00026	0.00026
MR	1.18	1	0.016391	0.003663	0.009887	0.000619	0.000533	0.000968
		2	0.015788	0.004279	0.005495	0.001144	0.000637	0.000831
		3	0.016199	0.005045	0.006374	0.000639	0.000518	0.000903
		4	0.014365	0.002705	0.007717	0.000685	0.000369	0.000535
		5	0.017837	0.004521	0.009625	0.000586	0.000445	0.000759
		6	0.015247	0.003267	0.009699	0.000516	0.000407	0.000702
		7	0.016684	0.004012	0.00515	0.000608	0.000592	0.001084
		8	0.013602	0.004006	0.005289	0.000752	0.000413	0.000523
		9	0.016614	0.002664	0.00548	0.000581	0.000306	0.000412
		10	0.014488	0.002644	0.012479	0.001441	0.000619	0.000846
		11	0.016253	0.003951	0.010177	0.000494	0.000453	0.000838
		12	0.015199	0.003768	0.008972	0.000495	0.000485	0.000881
		13	0.012197	0.003359	0.005672	0.000525	0.00037	0.000641
Neutron Diffraction	N/A	1	0.003	0.003	0.004	0.003	0.003	0.003
		2	0.003	0.003	0.004	0.003	0.003	0.003
		3	0.003	0.004	0.004	0.003	0.003	0.003
		4	0.003	0.003	0.003	0.003	0.003	0.003
		5	0.003	0.004	0.004	0.003	0.003	0.003
		6	0.003	0.004	0.004	0.003	0.003	0.003
		7	0.003	0.004	0.004	0.003	0.003	0.003
		8	0.003	0.004	0.004	0.003	0.003	0.003
		9	0.003	0.003	0.003	0.003	0.003	0.003
		10	0.003	0.004	0.004	0.003	0.003	0.003
		11	0.003	0.004	0.004	0.003	0.003	0.003
		12	0.003	0.004	0.004	0.003	0.003	0.003
		13	0.003	0.004	0.004	0.003	0.003	0.003
		14	0.003	0.003	0.003	0.003	0.003	0.003

(i) -x, 1-y, -z

Method	Resolution [Å -1]	nr set	C1-O1-H1	O1 - C1 - C1 i	O2 - C1 - C1 i	O2-C1-O1	H2-O3-H3
IAM	0.625	1	113.223999	112.252998	120.504997	127.241997	105.737
		2	112.381	112.264	120.493	127.242	105.524
		3	113.115997	112.209999	120.406998	127.382004	106.628998
		4	112.417	112.158997	120.526001	127.314003	105.733002
		5	113.93	112.268997	120.509003	127.221001	105.749001
		6	112.668999	112.211998	120.459	127.328003	106.549004
		7	112.923	111.926	120.775	127.297	106.732
		8	113.489998	112.150002	120.467003	127.383003	105.380997
		9	113.269997	112.119003	120.515999	127.363998	105.613998
		10	113.003998	112.238998	120.462997	127.295998	105.908997
		11	112.796997	112.196999	120.498001	127.305	107.862
		12	111.417999	112.210999	120.416	127.371002	107.188004
		13	112.746002	112.230003	120.619003	127.150002	105.139
	0.71429	1	112.738	112.307	120.56	127.132	105.037
		2	111.806999	112.305	120.563004	127.132004	105.787003
		3	112.681	112.224998	120.504997	127.268997	106.636002
		4	112.098999	112.245003	120.573997	127.181	105.556
		5	113.123001	112.269997	120.537003	127.192001	105.915001
		6	111.842003	112.245003	120.524002	127.230003	106.140999
		7	112.832001	112.082001	120.690002	127.227997	106.264999
		8	112.611	112.170998	120.541	127.288002	104.931999
		9	112.207001	112.168999	120.565002	127.264999	105.790001
		10	112.382004	112.273003	120.541	127.183998	105.721001
		11	112.737	112.283997	120.532997	127.181999	106.967003
		12	111.655998	112.309998	120.422997	127.264999	106.482002
		13	112.056	112.358002	120.569	127.071999	104.941002
	0.83333	1	112.343002	112.458	120.502998	127.039001	105.566002
		2	112.047997	112.448997	120.530998	127.018997	106.411003
		3	112.544998	112.464996	120.411003	127.123001	106.263
		4	112.345001	112.448997	120.495003	127.056	105.615997
		5	113.086998	112.472	120.459	127.068001	106.014999
		6	111.735001	112.469002	120.452003	127.078003	106.003998

		7	113.418999	112.367996	120.556	127.074997	106.553001
		8	112.688004	112.398003	120.471001	127.129997	104.869003
		9	111.887001	112.434998	120.440002	127.124001	105.301003
		10	112.443001	112.402	120.500999	127.095001	105.742996
		11	112.620003	112.511002	120.459	127.028999	106.066002
		12	110.732002	112.524002	120.374001	127.100998	105.331001
		13	111.917	112.566002	120.461998	126.971001	104.667999
	1	1	112.217003	112.518997	120.487	126.992996	105.802002
		2	112.177002	112.490997	120.494003	127.014	106.540001
		3	112.442001	112.528999	120.414001	127.056	106.443001
		4	112.239998	112.499001	120.462997	127.037003	105.903999
		5	112.614998	112.564003	120.424004	127.012001	106.361
		6	111.524002	112.517998	120.431	127.050003	106.182999
		7	113.512001	112.473999	120.515999	127.009003	106.533997
		8	112.749001	112.462997	120.446999	127.089996	105.046997
		9	111.988998	112.528	120.389	127.082001	105.238998
		10	112.421997	112.449997	120.468002	127.080002	105.867996
		11	112.5	112.579002	120.433998	126.985001	106.171997
		12	110.292999	112.589996	120.358002	127.050003	105.054001
		13	111.969002	112.607002	120.413002	126.978996	105.029999
	1.14	1	112.223	112.533997	120.474998	126.990997	106.417999
		2	111.762001	112.486	120.514999	126.998001	106.448997
		3	112.084999	112.538002	120.412003	127.049004	107.876999
		4	112.199997	112.504997	120.431	127.063004	105.903999
		5	112.296997	112.57	120.417	127.012001	106.378998
		6	111.667	112.528999	120.429001	127.041	105.717003
		7	111.858002	112.531998	120.475998	126.990997	106.967003
		8	112.936996	112.470001	120.448997	127.081001	105.321999
		9	112.577003	112.559998	120.386002	127.053001	105.485001
		10	112.948997	112.462997	120.503998	127.030998	105.859001
		11	112.580002	112.584	120.425003	126.989998	106.025002
		12	110.332001	112.605003	120.341003	127.053001	105.712997
		13	112.012001	112.594002	120.413002	126.990997	105.064003
UBDB	0.625	1	111.292999	112.900002	120.218002	126.880997	103.682999
		2	110.928001	112.825996	120.213997	126.959999	105.913002
		3	111.816002	112.783997	120.272003	126.943001	105.93

		4	110.875	112.744003	120.245003	127.010002	105.213997
		5	111.925003	112.795998	120.288002	126.915001	104.277
		6	111.049004	112.828003	120.210999	126.959999	104.475998
		7	110.572998	112.802002	120.306	126.890999	103.594002
		8	111.320999	112.791	120.184998	127.023003	104.188004
		9	111.290001	112.82	120.188004	126.990997	103.983002
		10	111.778	112.82	120.185997	126.992996	105.122002
		11	111.178001	112.760002	120.306	126.933998	106.353996
		12	111.512001	112.802002	120.271004	126.925003	106.387001
		13	111.300003	112.789001	120.296997	126.913002	104.167999
	0.71429	1	111.174004	112.853996	120.291	126.855003	103.477997
		2	110.928001	112.786003	120.296997	126.916	106.899002
		3	112.132004	112.665001	120.344002	126.988998	105.642998
		4	111.252998	112.709999	120.314003	126.974998	105.693001
		5	111.863998	112.788002	120.321999	126.889999	104.599998
		6	111.179001	112.713997	120.289001	126.996002	104.272003
		7	110.202003	112.788002	120.327003	126.884003	104.125
		8	111.109001	112.765999	120.237	126.996002	104.060997
		9	111.141998	112.763	120.260002	126.975998	104.595001
		10	111.936996	112.799004	120.254997	126.945	105.445
		11	112.654999	112.779999	120.309998	126.907997	105.764999
		12	111.793999	112.700996	120.332001	126.966003	105.897003
		13	111.028	112.774002	120.337997	126.887001	103.957001
	0.83333	1	111.440002	112.819	120.333	126.847	103.512001
		2	110.959999	112.741997	120.355003	126.902	106.940002
		3	112.067001	112.782997	120.348999	126.866997	106.066002
		4	111.523003	112.736	120.351997	126.911003	105.725998
		5	112.168999	112.737999	120.369003	126.891998	104.974998
		6	111.389999	112.779999	120.333	126.886002	104.611
		7	110.087997	112.752998	120.366997	126.878998	104.699997
		8	110.992996	112.723999	120.308998	126.966003	104.385002
		9	111.045998	112.711998	120.333	126.955002	104.774002
		10	112.015999	112.767998	120.281998	126.948997	105.717003
		11	112.917	112.810997	120.358002	126.830002	105.972
		12	111.429001	112.773003	120.351997	126.874001	105.470001
		13	111.179001	112.782997	120.334	126.882004	104.042

	1	1	111.209	112.789001	120.356003	126.855003	103.426003
		2	110.991997	112.723	120.372002	126.903999	107.031998
		3	112.028	112.748001	120.372002	126.878998	106.088997
		4	111.489998	112.723999	120.360001	126.915001	105.856003
		5	112.165001	112.713997	120.370003	126.915001	105.163002
		6	111.279999	112.742996	120.348999	126.906998	105.124001
		7	109.887001	112.75	120.382004	126.866997	104.841003
		8	110.728996	112.695999	120.337997	126.964996	104.511002
		9	110.919998	112.708	120.332001	126.959999	104.457001
		10	112.061996	112.751999	120.289001	126.958	105.821999
		11	113.046997	112.778	120.355003	126.865997	106.084
		12	111.165001	112.748001	120.336998	126.914001	104.990997
		13	111.051003	112.761002	120.324997	126.913002	104.124001
	1.14	1	111.060997	112.772003	120.358002	126.869003	103.787003
		2	110.821999	112.709999	120.391998	126.897003	107.434998
		3	111.141998	112.741997	120.366997	126.890999	106.459
		4	111.151001	112.723	120.333	126.944	105.625
		5	111.153	112.708	120.367996	126.924004	106.111
		6	111.195	112.737999	120.347	126.914001	104.842003
		7	110.194	112.750999	120.365997	126.882004	105.125999
		8	110.734001	112.693001	120.343002	126.962997	104.596001
		9	110.903	112.697998	120.336998	126.963997	103.634003
		10	112.232002	112.787003	120.342003	126.870003	106.031998
		11	112.903	112.774002	120.346001	126.878998	105.205002
		12	111.056	112.728996	120.325996	126.944	105.287003
		13	111.088997	112.740997	120.332001	126.926003	104.186996
ELMAM2	0.625	1	111.051003	112.959999	120.221001	126.818001	103.544998
		2	111.213997	112.870003	120.196999	126.932999	106.542
		3	111.741997	112.822998	120.271004	126.904999	105.758003
		4	110.963997	112.794998	120.244003	126.959999	105.552002
		5	111.792	112.856003	120.264999	126.878998	104.539001
		6	111.041	112.869003	120.207001	126.922997	104.556999
		7	110.523003	112.903999	120.253998	126.841003	104.371002
		8	111.570999	112.889999	120.148003	126.961998	105.057999
		9	111.400002	112.915001	120.167	126.916	104.920998
		10	112.074997	112.875	120.167999	126.956001	105.696999

	11	111.108002	112.815002	120.307999	126.876999	105.533997
	12	111.933998	112.830002	120.278	126.890999	105.535004
	13	111.364998	112.829002	120.265999	126.903999	105.001999
0.71429	1	110.966003	112.889999	120.281998	126.827003	103.445
	2	111.014	112.820999	120.258003	126.920998	106.695
	3	111.855003	112.697998	120.329002	126.972	105.385002
	4	111.053001	112.738998	120.307999	126.953003	105.960999
	5	111.737	112.742996	120.307999	126.947998	104.853996
	6	111.153	112.736	120.276001	126.987999	104.401001
	7	110.261002	112.825996	120.290001	126.882004	104.691002
	8	111.213997	112.842003	120.209	126.948997	104.845001
	9	111.141998	112.825996	120.237999	126.934998	105.105003
	10	111.989998	112.828003	120.235001	126.936996	105.929001
	11	112.567001	112.808998	120.304001	126.885002	104.802002
	12	111.997002	112.706001	120.324997	126.968002	105.123001
	13	110.880997	112.786003	120.310997	126.902	104.416
0.83333	1	111.141998	112.818001	120.347	126.834	103.612999
	2	110.827003	112.761002	120.324997	126.913002	106.874001
	3	112.069	112.681999	120.374001	126.944	105.581001
	4	111.200996	112.713997	120.362999	126.921997	106.150002
	5	111.822998	112.730003	120.367996	126.902	105.390999
	6	111.342003	112.767998	120.343002	126.888	104.775002
	7	109.82	112.781998	120.349998	126.866997	105.132004
	8	110.897003	112.752998	120.306999	126.940002	105.237999
	9	110.945999	112.727997	120.334999	126.935997	105.334999
	10	111.938004	112.767998	120.285004	126.945999	106.365997
	11	112.760002	112.805	120.372002	126.821999	105.271004
	12	111.598	112.762001	120.353996	126.882004	104.865997
	13	110.890999	112.768997	120.330002	126.900002	104.640999
1	1	112.507004	112.620003	120.482002	126.897003	102.956001
	2	111.990997	112.554001	120.474998	126.970001	103.348
	3	112.143997	112.561996	120.456001	126.982002	103.464996
	4	111.551003	112.546997	120.463997	126.987999	103.245003
	5	112.382004	112.587997	120.432999	126.977997	104.023003
	6	112.079002	112.568001	120.425003	127.005997	103.565002
	7	112.18	112.607002	120.498001	126.894997	104.203003

		8	112.052002	112.537003	120.474998	126.987	102.487
		9	111.822998	112.592003	120.412003	126.996002	102.063004
		10	111.957001	112.545998	120.446999	127.004997	103.042999
		11	113.234001	112.639999	120.425003	126.933998	103.764
		12	111.443001	112.620003	120.392998	126.986	102.961998
		13	112.199997	112.620003	120.408997	126.970001	102.386002
	1.14	1	110.733002	112.780998	120.355003	126.862999	104.014999
		2	110.662003	112.728996	120.362	126.907997	107.412003
		3	111.204002	112.758003	120.344002	126.898003	105.462997
		4	110.732002	112.734001	120.329002	126.935997	106.289001
		5	111.150002	112.723999	120.339996	126.935997	105.626999
		6	111.112	112.748001	120.334	126.917	104.939003
		7	109.875	112.780998	120.343002	126.875	105.260002
		8	110.369003	112.723999	120.331001	126.944	105.623001
		9	110.411003	112.709999	120.327003	126.961998	104.643997
		10	112.084999	112.792999	120.336998	126.869003	106.433998
		11	112.75	112.790001	120.334999	126.874001	104.522003
		12	110.852997	112.731003	120.315002	126.953003	105.438004
		13	110.738998	112.75	120.314003	126.935997	104.875
GID	0.625	1	111.051003	112.959999	120.221001	126.818001	103.544998
		2	111.213997	112.870003	120.196999	126.932999	106.542
		3	111.741997	112.822998	120.271004	126.904999	105.758003
		4	110.963997	112.794998	120.244003	126.959999	105.552002
		5	111.792	112.856003	120.264999	126.878998	104.539001
		6	111.041	112.869003	120.207001	126.922997	104.556999
		7	110.523003	112.903999	120.253998	126.841003	104.371002
		8	111.570999	112.889999	120.148003	126.961998	105.057999
		9	111.400002	112.915001	120.167	126.916	104.920998
		10	112.074997	112.875	120.167999	126.956001	105.696999
		11	111.108002	112.815002	120.307999	126.876999	105.533997
		12	111.933998	112.830002	120.278	126.890999	105.535004
		13	111.364998	112.829002	120.265999	126.903999	105.001999
	0.71429	1	110.966003	112.889999	120.281998	126.827003	103.445
		2	111.014	112.820999	120.258003	126.920998	106.695
		3	111.855003	112.697998	120.329002	126.972	105.385002
		4	111.053001	112.738998	120.307999	126.953003	105.960999

		5	111.737	112.742996	120.307999	126.947998	104.853996
		6	111.153	112.736	120.276001	126.987999	104.401001
		7	110.261002	112.825996	120.290001	126.882004	104.691002
		8	111.213997	112.842003	120.209	126.948997	104.845001
		9	111.141998	112.825996	120.237999	126.934998	105.105003
		10	111.989998	112.828003	120.235001	126.936996	105.929001
		11	112.567001	112.808998	120.304001	126.885002	104.802002
		12	111.997002	112.706001	120.324997	126.968002	105.123001
		13	110.880997	112.786003	120.310997	126.902	104.416
	0.83333	1	111.141998	112.818001	120.347	126.834	103.612999
		2	110.827003	112.761002	120.324997	126.913002	106.874001
		3	112.069	112.681999	120.374001	126.944	105.581001
		4	111.200996	112.713997	120.362999	126.921997	106.150002
		5	111.822998	112.730003	120.367996	126.902	105.390999
		6	111.342003	112.767998	120.343002	126.888	104.775002
		7	109.82	112.781998	120.349998	126.866997	105.132004
		8	110.897003	112.752998	120.306999	126.940002	105.237999
		9	110.945999	112.727997	120.334999	126.935997	105.334999
		10	111.938004	112.767998	120.285004	126.945999	106.365997
		11	112.760002	112.805	120.372002	126.821999	105.271004
		12	111.598	112.762001	120.353996	126.882004	104.865997
		13	110.890999	112.768997	120.330002	126.900002	104.640999
	1	1	110.842003	112.799004	120.351997	126.847	103.629997
		2	110.865997	112.747002	120.336998	126.915001	106.986
		3	111.665001	112.759003	120.364998	126.875999	106.022003
		4	111.077003	112.737999	120.349998	126.912003	106.442001
		5	111.725998	112.724998	120.352997	126.921997	105.600998
		6	111.307999	112.757004	120.334999	126.906998	105.209999
		7	109.686996	112.793999	120.347	126.858002	105.231003
		8	110.540001	112.732002	120.321999	126.945	105.377998
		9	110.634003	112.738998	120.313004	126.946999	105.209
		10	111.918999	112.762001	120.279999	126.957001	106.525002
		11	112.893997	112.793999	120.343002	126.862	105.470001
		12	111.307999	112.761002	120.319	126.918999	104.635002
		13	110.699997	112.771004	120.303001	126.925003	104.813004
	1.14	1	110.733002	112.780998	120.355003	126.862999	104.014999

		2	110.662003	112.728996	120.362	126.907997	107.412003
		3	111.204002	112.758003	120.344002	126.898003	105.462997
		4	110.732002	112.734001	120.329002	126.935997	106.289001
		5	111.150002	112.723999	120.339996	126.935997	105.626999
		6	111.112	112.748001	120.334	126.917	104.939003
		7	109.875	112.780998	120.343002	126.875	105.260002
		8	110.369003	112.723999	120.331001	126.944	105.623001
		9	110.411003	112.709999	120.327003	126.961998	104.643997
		10	112.084999	112.792999	120.336998	126.869003	106.433998
		11	112.75	112.790001	120.334999	126.874001	104.522003
		12	110.852997	112.731003	120.315002	126.953003	105.438004
		13	110.738998	112.75	120.314003	126.935997	104.875
HAR(B3LYP)	0.625	1	113.171564	112.684105	120.391961	126.923305	106.121053
		2	113.171564	112.684105	120.391961	126.923305	106.121053
		3	113.114368	112.588633	120.408866	127.001731	107.515486
		4	112.474017	112.545893	120.398634	127.054853	106.941726
		5	113.535855	112.600554	120.404003	126.994936	106.739263
		6	113.306956	112.604732	120.397818	126.996703	107.114114
		7	113.09688	112.633118	120.453016	126.913147	107.003349
		8	113.08515	112.631411	120.450674	126.917187	106.95315
		9	113.262156	112.591452	120.382928	127.024933	106.845908
		10	113.012885	112.606789	120.363407	127.028608	106.250001
		11	113.364385	112.640118	120.326054	127.033041	107.130711
		12	114.097505	112.522876	120.485611	126.991311	107.425236
		13	113.036056	112.66432	120.371271	126.963415	106.362291
	0.71429	1	113.041674	112.685383	120.405034	126.908959	105.778053
		2	113.041674	112.685383	120.405034	126.908959	105.778053
		3	112.900422	112.673519	120.388053	126.937979	107.113586
		4	112.555279	112.587277	120.405059	127.0072	106.652007
		5	113.253127	112.636479	120.388809	126.974308	106.411375
		6	113.217616	112.636347	120.399873	126.96297	106.633244
		7	112.993858	112.673046	120.423948	126.9024	106.960473
		8	113.24389	112.666722	120.430355	126.902345	106.918011
		9	113.112116	112.610826	120.389493	126.998986	106.628676
		10	113.248471	112.648052	120.374997	126.97618	106.420948
		11	113.417936	112.656304	120.353866	126.988916	107.169021

	12	114.275334	112.518186	120.457425	127.023924	106.628078
	13	112.744921	112.692969	120.3758	126.930508	105.90444
0.83333	1	113.194764	112.679768	120.433212	126.8863	105.705416
	2	113.194764	112.679768	120.433212	126.8863	105.705416
	3	112.911635	112.6645	120.422348	126.912413	106.914649
	4	112.762868	112.620523	120.422392	126.956487	106.603402
	5	113.38352	112.620568	120.421067	126.957714	106.247829
	6	113.233854	112.66469	120.394966	126.939454	106.34009
	7	112.963817	112.66885	120.441604	126.888727	106.986334
	8	113.182269	112.660188	120.447076	126.891965	106.850069
	9	113.052653	112.608044	120.417626	126.973471	106.745535
	10	113.133879	112.630931	120.411056	126.957363	106.397073
	11	113.45871	112.641534	120.36849	126.988902	107.310418
	12	113.744052	112.675539	120.427459	126.89598	105.826988
	13	112.889199	112.670453	120.405956	126.922573	105.805828
1	1	113.055243	112.673088	120.438656	126.887533	105.815777
	2	113.055243	112.673088	120.438656	126.887533	105.815777
	3	112.924688	112.66118	120.424138	126.913875	106.923469
	4	112.792739	112.6326	120.41057	126.956282	106.470217
	5	113.292986	112.625606	120.408783	126.964928	106.278837
	6	113.183328	112.672387	120.374553	126.95221	106.440679
	7	112.896075	112.678712	120.440313	126.880097	106.936481
	8	113.121851	112.672419	120.444322	126.882417	106.780963
	9	112.939327	112.607021	120.420336	126.971891	106.764595
	10	113.120932	112.642371	120.382601	126.974391	106.237412
	11	113.489666	112.639396	120.366488	126.992951	107.371917
	12	113.752724	112.678422	120.40526	126.915269	105.7828
	13	112.811313	112.67732	120.372807	126.948928	105.766666
1.14	1	112.96677	112.667955	120.439251	126.892079	106.105436
	2	112.649597	112.67923	120.425658	126.894344	106.895942
	3	112.93168	112.667289	120.404844	126.92713	106.210081
	4	112.752699	112.631361	120.392735	126.975289	106.546527
	5	113.508581	112.629498	120.396948	126.972882	105.567679
	6	113.212012	112.654204	120.382908	126.962027	106.445144
	7	112.898727	112.652621	120.400575	126.946046	106.109711
	8	112.88144	112.679021	120.42889	126.891209	106.921001

		9	112.976702	112.602194	120.422833	126.974305	107.133111
		10	113.225994	112.636708	120.385987	126.97656	106.000215
		11	113.726802	112.676862	120.409885	126.912091	107.028398
		12	113.669957	112.677711	120.399501	126.921755	105.463039
		13	112.553239	112.669676	120.360687	126.968671	106.818015
HAR(BLYP)	0.625	1	112.807588	112.66927	120.382179	126.947798	106.03005
		2	112.557634	112.620233	120.379343	127.000115	106.837401
		3	112.849443	112.569558	120.398465	127.031128	107.432485
		4	112.224088	112.525306	120.396961	127.077073	106.748535
		5	113.252177	112.586355	120.398502	127.014576	106.556842
		6	112.522121	112.608461	120.353803	127.037074	106.663895
		7	112.761442	112.602724	120.454263	126.942231	106.762704
		8	113.020976	112.56361	120.380888	127.054754	106.727034
		9	113.012885	112.606789	120.363407	127.028608	106.250001
		10	113.201692	112.617267	120.320581	127.061278	106.869869
		11	113.730302	112.508353	120.476934	127.014424	107.350576
		12	112.807057	112.644211	120.363521	126.991212	106.58167
		13	113.024533	112.58426	120.399371	127.015559	106.875617
	0.71429	1	112.692199	112.675962	120.403264	126.920068	105.692269
		2	112.547474	112.628553	120.386816	126.984238	106.502139
		3	112.683702	112.661337	120.386727	126.951459	107.050361
		4	112.332707	112.573546	120.407434	127.018532	106.4166
		5	113.026654	112.627263	120.388091	126.984199	106.272229
		6	112.611958	112.561633	120.37894	127.058627	106.097345
		7	112.650269	112.650948	120.430798	126.917621	106.654072
		8	112.799673	112.588732	120.396152	127.014395	106.474163
		9	112.981696	112.627713	120.381456	126.990012	106.269105
		10	113.217471	112.636009	120.356252	127.006782	106.894974
		11	113.920778	112.509092	120.454897	127.03545	106.502051
		12	112.503447	112.680798	120.373762	126.944702	106.128393
		13	112.900193	112.62475	120.403287	126.97113	106.42044
	0.83333	1	112.828973	112.674702	120.432794	126.891718	105.640897
		2	112.55041	112.630605	120.414577	126.95402	106.415186
		3	112.710795	112.658675	120.421238	126.919323	106.885317
		4	112.556241	112.613158	120.424814	126.961407	106.40182
		5	113.181952	112.615889	120.421133	126.962288	106.142007

		6	112.599469	112.653463	120.405702	126.940058	106.101588
		7	112.593603	112.6563	120.446148	126.896717	106.72446
		8	112.755777	112.595019	120.423259	126.980842	106.570212
		9	112.870898	112.621523	120.415456	126.962354	106.255835
		10	113.258355	112.629531	120.371837	126.997514	107.029491
		11	113.398926	112.671752	120.427899	126.899257	105.820034
		12	112.608658	112.664915	120.404557	126.929493	106.019363
		13	112.913903	112.658678	120.398209	126.942197	106.136631
	1	1	112.662605	112.669832	120.438946	126.890456	105.762815
		2	112.646233	112.631221	120.416682	126.951328	106.446845
		3	112.719307	112.657591	120.423692	126.91789	106.89738
		4	112.588378	112.626925	120.412389	126.960115	106.287942
		5	113.095584	112.622725	120.409239	126.96733	106.192664
		6	112.516539	112.64939	120.399369	126.950501	106.250678
		7	112.532946	112.670842	120.44339	126.884874	106.675872
		8	112.630099	112.59889	120.424072	126.976266	106.611585
		9	112.82981	112.637061	120.384467	126.977819	106.068011
		10	113.303805	112.629719	120.368155	127.000898	107.070679
		11	113.395919	112.676046	120.406108	126.916754	105.787013
		12	112.49364	112.674459	120.372505	126.952082	105.902787
		13	112.871564	112.668399	120.377227	126.953497	106.231062
	1.14	1	112.571601	112.665216	120.439196	126.894839	106.062508
		2	112.479269	112.671698	120.428654	126.89887	106.760118
		3	112.746661	112.664009	120.403915	126.931323	106.090724
		4	112.561539	112.625547	120.39418	126.97963	106.392189
		5	113.213955	112.62728	120.396243	126.975787	105.368219
		6	112.606238	112.649337	120.400749	126.94914	106.100988
		7	112.4976	112.672792	120.431324	126.894972	106.70988
		8	112.664256	112.594393	120.425806	126.979108	106.923392
		9	112.936665	112.632424	120.387669	126.979152	105.856806
		10	113.55781	112.665605	120.412922	126.920252	106.878887
		11	113.30867	112.675742	120.400038	126.923156	105.47009
		12	112.283425	112.666793	120.35992	126.972318	106.956382
		13	112.906744	112.650516	120.384977	126.963624	106.241305
MR_no	1.19	1	117.195999	112.709999	120.432999	126.857002	108.348
		2	115.327003	112.703003	120.480003	126.815002	106.283997

		3	115.213997	112.695	120.411003	126.891998	103.581001
		4	117.93	112.691002	120.382004	126.926003	99.209
		5	116.057999	112.661003	120.389	126.948997	104.325996
		6	116.495003	112.681999	120.393997	126.922997	105.724998
		7	116.803001	112.741997	120.412003	126.845001	100.686996
		8	115.386002	112.641998	120.459	126.898003	106.389999
		9	118.171997	112.663002	120.374001	126.962997	97.390999
		10	115.202003	112.600998	120.480003	126.917	105.404999
		11	115.296997	112.708	120.393997	126.897003	108.997002
		12	115.757004	112.693001	120.351997	126.954002	108.047997
		13	114.716	112.678	120.382	126.938	107.09
MR	1.18	1	116.501709	112.707939	120.432823	126.858574	107.198105
		2	115.021011	112.698875	120.477852	126.821823	103.744469
		3	115.137077	112.695091	120.412483	126.891632	102.169205
		4	117.280228	112.691826	120.379128	126.928268	99.819778
		5	115.853844	112.661072	120.388664	126.949402	104.306221
		6	115.905861	112.681244	120.393784	126.924225	105.934471
		7	115.316711	112.744339	120.41378	126.841034	103.723442
		8	115.032318	112.639229	120.457634	126.902435	107.028458
		9	117.334473	112.661308	120.373009	126.964806	98.738892
		10	114.866798	112.598785	120.47715	126.92234	104.922722
		11	114.804283	112.70607	120.39518	126.897697	108.219101
		12	115.300652	112.691109	120.351601	126.956314	107.211449
		13	115.059326	112.676094	120.38018	126.942871	104.172714
Neutron Diffraction	N/A	1	113.1	112.32	120.61	127.07	106.2
		2	113.2	112.35	120.62	127.03	106.3
		3	113.1	112.37	120.59	127.04	105.9
		4	113.0	112.34	120.69	126.96	106.1
		5	113.3	112.58	120.54	126.88	106.1
		6	113.1	112.38	120.53	127.09	106.1
		7	113.2	112.36	120.62	127.03	106.3
		8	113.0	112.45	120.50	127.05	106.3
		9	113.1	112.51	120.34	127.15	105.9
		10	113.3	112.38	120.61	127.01	105.9
		11	113.2	112.40	120.54	127.06	106.2
		12	113.0	112.21	120.76	127.03	105.9

13	113.1	112.38	120.55	127.07	106.3
14	113.2	112.41	120.53	127.06	106.1

Method	Resolution [Å $^{-1}$]	nr set	O1-H1-O3	(ii) x, 1.5-y, 1/2+z<br>O3 - H2 - O2 ^{ii}	(iii) 1-x, -1/2+y, 1/2-z<br>O3 - H3 -O2 ^{iii}
IAM	0.625	1	178.539993	170.386993	152.597
		2	177.247	168.655	155.516
		3	178.975006	168.867996	153.792999
		4	177.378998	169.139999	154.365005
		5	179.322998	170.167007	153.526001
		6	177.615997	169.587006	152.931
		7	178.391	169.83	152.879
		8	179.257996	169.354996	154.410995
		9	178.658997	169.718994	153.710999
		10	178.619003	169.837997	153.380997
		11	176.725006	167.546997	153.535995
		12	175.809006	167.666	154.654999
		13	177.572006	169.300003	155.598999
	0.71429	1	177.68	170.324	153.201
		2	176.210999	168.257004	155.375
		3	178.259995	167.970001	154.658005
		4	176.813995	168.753998	155.024002
		5	178.727005	169.072006	154.244003
		6	176.341003	169.231995	153.852997
		7	178.279999	169.617004	153.468002
		8	177.996994	169.475006	155.085999
		9	177.320007	169.190002	154.401001
		10	177.662003	169.643997	153.546005
		11	176.304001	168.567001	153.190002
		12	174.503998	167.028	155.970001
		13	176.434998	168.962006	156.005005
	0.83333	1	175.895004	169.903	153.557007
		2	176.209	168.414993	154.653
		3	177.742004	168.145996	154.718002
		4	176.701004	168.789993	154.878006
		5	178.511993	169.179001	153.968994

		6	175.998001	169.615005	153.606003
		7	179.141006	169.492004	153.304001
		8	177.615005	169.701996	154.903
		9	176.289001	169.050003	155.011002
		10	177.576996	169.910004	153.080994
		11	174.720001	169.296997	153.671997
		12	172.044006	167.569	156.630005
		13	175.580002	169.050995	156.197998
	1	1	175.460007	169.854996	153.544998
		2	176.235992	168.278	154.658997
		3	177.311005	167.727005	155.156006
		4	176.371994	168.589005	154.860001
		5	177.656006	168.296997	154.076004
		6	175.815002	169.395004	153.895004
		7	178.802002	169.574005	153.332993
		8	177.516006	169.244003	155.016006
		9	176.164001	169.037994	154.792999
		10	177.306	169.850998	152.953003
		11	174.914001	169.337006	153.852005
		12	170.994995	167.641998	157.072006
		13	175.345993	168.649002	156.264999
	1.14	1	175.923004	169.619995	153.395996
		2	175.878998	168.138	155.039001
		3	176.608994	167.798996	153.725006
		4	175.979996	168.966003	154.630005
		5	177.076004	168.960999	153.468002
		6	176.110001	169.654007	154.229996
		7	176.039993	167.923996	154.533997
		8	177.860992	169.602005	154.335007
		9	177.100998	169.356995	154.464005
		10	178.369995	169.949005	153.009995
		11	175.639999	169.531006	153.740005
		12	170.908997	167.919998	156.175995
		13	175.339996	168.664993	156.181
UBDB	0.625	1	173.817001	167.682999	157.016006

	2	174.041	164.830994	157.459
	3	174.854004	165.878998	156.218002
	4	173.645996	166.080002	156.623001
	5	175.080994	167.175995	156.376999
	6	174.151001	168.235992	155.906998
	7	173.524994	168.686996	157.022003
	8	174.505005	167.518997	156.641006
	9	174.227005	167.690994	156.460007
	10	175.358994	166.837997	155.895996
	11	174.358002	166.429001	154.651993
	12	174.054993	165.716003	156.016006
	13	174.195999	166.981003	157.248001
0.71429	1	173.843002	167.699005	157.139999
	2	173.921005	164.876999	156.014008
	3	175.052994	166.615005	156.125
	4	174.199997	166.014999	156.276001
	5	175.261993	166.806	155.949005
	6	174.251007	168.507996	156.020004
	7	173.003998	167.953003	156.845993
	8	174.123993	167.761993	156.675995
	9	174.095001	167.718994	155.830002
	10	175.707001	166.852997	155.488998
	11	174.481003	166.320007	155.772003
	12	174.501999	166.987	155.509003
	13	173.731003	167.201004	157.100006
0.83333	1	174.190994	167.703995	157.358994
	2	174.061005	165.125	155.755997
	3	175.753006	165.466995	155.574997
	4	174.634995	165.813004	156.089005
	5	175.884995	166.673996	155.585007
	6	174.766006	167.865997	155.600006
	7	172.908005	167.227997	156.783997
	8	174.065994	167.671997	156.242004
	9	173.968994	167.501999	155.826996
	10	175.854996	166.733994	155.181

		11	175.751999	165.723999	155.806
		12	174.563004	166.597	155.395004
		13	174.069	167.121002	156.938995
	1	1	174.072006	167.781998	157.483994
		2	174.162003	165.337006	155.505997
		3	175.792999	165.662994	155.552994
		4	174.576996	165.882996	155.957001
		5	175.998001	166.731995	155.287994
		6	174.705994	167.653	155.225998
		7	172.610001	167.156006	156.634995
		8	173.679001	167.541	156.199997
		9	173.695007	167.701004	155.632004
		10	175.973999	166.610001	155.214005
		11	175.889999	165.714005	155.876007
		12	174.197006	167.022995	155.856003
		13	173.925003	167.102005	156.871002
	1.14	1	173.841995	167.526001	157.468002
		2	173.945007	164.979996	155.339996
		3	174.274002	166.016998	155.070999
		4	174.130005	166.563995	155.873001
		5	174.516006	166.369995	154.567993
		6	174.626999	167.490005	155.591003
		7	172.673004	166.479996	156.994003
		8	173.779007	167.617996	156.048004
		9	173.436996	168.658997	156.212997
		10	176.367996	166.886002	154.574997
		11	175.546997	166.179993	156.302994
		12	173.615005	167.414993	155.218994
		13	174.072006	167.203995	156.673996
ELMAM2	0.625	1	173.222	166.886002	156.505005
		2	174.091995	165.391998	155.626007
		3	174.389999	165.692001	155.772995
		4	173.287994	165.587997	155.817993
		5	174.613998	166.216003	155.858994
		6	173.884003	167.360992	155.548004

	7	173.089996	167.076004	156.011993
	8	174.563004	166.723007	155.587997
	9	173.694	166.669998	155.371002
	10	175.367996	166.149002	154.847
	11	174.296997	165.917999	154.923004
	12	174.858994	166.235992	155.419998
	13	174.052994	166.080994	156.069
0.71429	1	173.322006	166.524994	157.011993
	2	173.802002	165.147003	155.371002
	3	174.201996	166.281006	156.048004
	4	173.444	165.535995	155.729996
	5	174.684006	166.056	155.837997
	6	173.949997	167.404007	156.005997
	7	172.664993	166.873993	156.067001
	8	173.921005	166.819	155.783997
	9	173.453003	166.964996	155.231995
	10	175.240997	166.093994	154.720993
	11	174.412994	165.703995	156.373001
	12	174.897995	167.360001	155.057999
	13	173.335999	166.423004	156.343994
0.83333	1	173.367004	166.139008	157.451996
	2	173.587006	165.011002	155.315002
	3	174.638	165.929993	156.029999
	4	173.720993	165.156006	155.679993
	5	175.009995	165.306	155.412994
	6	174.378006	166.513	155.839996
	7	172.238007	165.975998	156.106995
	8	173.569	166.550003	155.334
	9	173.255997	166.466003	155.468002
	10	175.154999	165.621002	154.434998
	11	175.255997	164.625	156.505005
	12	174.858994	166.658997	154.901993
	13	173.404007	166.067001	156.162994
1	1	174.311996	168.587006	155.897003
	2	175.785004	167.048004	156.955994

		3	173.895996	167.615997	156.522995
		4	173.751007	167.718002	156.352997
		5	175.356003	167.363007	154.882996
		6	174.919998	168.766006	155.557007
		7	175.897003	168.542999	154.947998
		8	175.565002	167.973999	156.615005
		9	174.432007	168.944	155.985001
		10	175.528	168.126007	155.531998
		11	174.050995	167.755005	155.591995
		12	173.093994	167.813995	156.319
		13	175.089005	168.046005	156.679001
	1.14	1	172.830002	165.578995	157.705994
		2	173.417999	164.552994	155.074997
		3	174.154007	165.154999	155.889999
		4	173.061005	165.404999	155.356995
		5	174.283997	164.552994	154.985001
		6	174.115997	166.029999	155.725998
		7	171.595001	164.789001	156.835007
		8	172.837997	166.059006	155.110001
		9	172.125	166.992996	155.632004
		10	175.654999	165.917999	153.979004
		11	174.612	164.630005	157.067001
		12	173.059006	166.651993	154.854996
		13	173.216995	165.854996	155.839996
GID	0.625	1	173.222	166.886002	156.505005
		2	174.091995	165.391998	155.626007
		3	174.389999	165.692001	155.772995
		4	173.287994	165.587997	155.817993
		5	174.613998	166.216003	155.858994
		6	173.884003	167.360992	155.548004
		7	173.089996	167.076004	156.011993
		8	174.563004	166.723007	155.587997
		9	173.694	166.669998	155.371002
		10	175.367996	166.149002	154.847
		11	174.296997	165.917999	154.923004

		12	174.858994	166.235992	155.419998
		13	174.052994	166.080994	156.069
	0.71429	1	173.322006	166.524994	157.011993
		2	173.802002	165.147003	155.371002
		3	174.201996	166.281006	156.048004
		4	173.444	165.535995	155.729996
		5	174.684006	166.056	155.837997
		6	173.949997	167.404007	156.005997
		7	172.664993	166.873993	156.067001
		8	173.921005	166.819	155.783997
		9	173.453003	166.964996	155.231995
		10	175.240997	166.093994	154.720993
		11	174.412994	165.703995	156.373001
		12	174.897995	167.360001	155.057999
		13	173.335999	166.423004	156.343994
	0.83333	1	173.367004	166.139008	157.451996
		2	173.587006	165.011002	155.315002
		3	174.638	165.929993	156.029999
		4	173.720993	165.156006	155.679993
		5	175.009995	165.306	155.412994
		6	174.378006	166.513	155.839996
		7	172.238007	165.975998	156.106995
		8	173.569	166.550003	155.334
		9	173.255997	166.466003	155.468002
		10	175.154999	165.621002	154.434998
		11	175.255997	164.625	156.505005
		12	174.858994	166.658997	154.901993
		13	173.404007	166.067001	156.162994
	1	1	173.158997	165.860992	157.684006
		2	173.667007	165.076004	155.125
		3	174.763	164.994003	155.481995
		4	173.531006	164.897003	155.347
		5	174.921997	165.192993	155.112
		6	174.373001	166.203995	155.533997
		7	172.037003	165.712006	156.126999

		8	173.014008	166.300995	155.231003
		9	172.574997	166.345001	155.158005
		10	175.128006	165.389008	154.393005
		11	175.166	164.274002	156.697998
		12	174.384003	166.654999	155.350006
		13	173.091995	165.854004	155.981995
	1.14	1	172.830002	165.578995	157.705994
		2	173.417999	164.552994	155.074997
		3	174.154007	165.154999	155.889999
		4	173.061005	165.404999	155.356995
		5	174.283997	164.552994	154.985001
		6	174.115997	166.029999	155.725998
		7	171.595001	164.789001	156.835007
		8	172.837997	166.059006	155.110001
		9	172.125	166.992996	155.632004
		10	175.654999	165.917999	153.979004
		11	174.612	164.630005	157.067001
		12	173.059006	166.651993	154.854996
		13	173.216995	165.854996	155.839996
HAR(B3LYP)	0.625	1	176.189549	165.114991	156.245093
		2	176.189549	165.114991	156.245093
		3	176.578408	164.207223	155.672609
		4	175.843657	164.451952	156.109902
		5	176.731475	165.085795	155.261807
		6	177.293168	164.393644	155.869558
		7	177.350118	164.90836	155.752572
		8	177.410981	164.975648	155.728917
		9	177.210355	164.679201	156.142031
		10	176.099868	165.373112	155.772226
		11	177.419019	165.102757	154.782576
		12	180	166.612761	153.786483
		13	176.327982	164.951061	155.645692
	0.71429	1	176.512136	165.167153	156.158882
		2	176.512136	165.167153	156.158882
		3	177.103215	164.244135	155.257009

	4	176.050081	164.660845	155.911509
	5	177.218347	165.141687	155.04339
	6	177.313962	164.924975	155.676036
	7	177.129465	164.848048	155.312857
	8	176.727157	165.00755	154.86149
	9	177.327577	164.821867	156.006821
	10	176.403231	165.477916	155.415728
	11	177.609299	165.058724	154.558686
	12	179.732159	166.884489	153.95817
	13	177.045485	165.260664	154.664208
0.83333	1	176.323149	165.350006	156.182999
	2	176.323149	165.350006	156.182999
	3	177.168993	164.380268	155.232039
	4	176.245326	164.794946	155.792749
	5	177.411961	165.4308	154.768874
	6	177.349386	165.024768	155.807756
	7	177.18297	164.775138	155.225467
	8	176.796081	165.074288	154.550107
	9	177.32633	164.609061	155.759296
	10	176.483556	165.438651	155.42109
	11	177.612186	164.934092	154.333017
	12	176.941408	165.378585	155.592751
	13	177.218179	165.356194	154.658843
1	1	176.616846	165.173143	156.208435
	2	176.616846	165.173143	156.208435
	3	177.168367	164.353039	155.306387
	4	176.376615	164.925404	155.890117
	5	177.449629	165.576866	154.548459
	6	177.299218	164.892591	155.821104
	7	176.939045	164.799296	155.174391
	8	176.527121	165.117827	154.478549
	9	177.226077	164.521916	155.782402
	10	176.253145	165.477773	155.280382
	11	177.60695	164.757877	154.3674
	12	176.750911	165.530376	155.568825

		13	176.884138	165.086794	154.856333
	1.14	1	176.2914	165.025196	156.193469
		2	176.631071	164.468581	156.469362
		3	177.206511	164.702098	155.753968
		4	176.248256	164.853619	156.026296
		5	177.889108	165.785712	155.323557
		6	177.3593	164.937339	155.733502
		7	176.801671	165.790012	155.125223
		8	176.186652	164.53128	155.806398
		9	177.307725	164.195551	155.546988
		10	176.023239	166.086166	155.425735
		11	178.236414	165.654906	154.456024
		12	176.318521	165.698783	155.708207
		13	175.797327	164.818235	154.067376
HAR(BLYP)	0.625	1	176.088401	165.176806	156.276274
		2	176.088716	164.811093	156.238235
		3	176.441096	164.291122	155.670573
		4	175.519409	164.586113	156.118564
		5	176.684282	165.218679	155.290551
		6	176.582927	165.658031	155.277561
		7	176.975572	165.10914	155.75853
		8	176.933888	164.815917	156.140853
		9	176.099868	165.373112	155.772226
		10	177.297006	165.311309	154.882553
		11	179.214453	166.520198	153.924093
		12	175.94695	164.828961	155.505931
		13	176.986819	164.615669	155.942922
	0.71429	1	176.209751	165.198832	156.155933
		2	176.315816	164.85463	156.25794
		3	176.853603	164.294786	155.213393
		4	175.817782	164.839614	155.981847
		5	177.128969	165.239419	155.066077
		6	176.158949	166.165223	155.239748
		7	176.644319	165.079955	155.361784
		8	176.834056	164.952191	155.961402

		9	176.144726	165.672905	155.334426
		10	177.29013	165.266342	154.598978
		11	179.412733	166.832969	154.0834
		12	176.576216	165.065556	154.581411
		13	176.876786	165.067825	155.744139
	0.83333	1	176.060357	165.369147	156.163364
		2	176.461829	165.090866	156.174511
		3	176.892108	164.439348	155.123268
		4	176.000594	164.94865	155.84303
		5	177.351838	165.523458	154.760688
		6	176.681682	165.908386	154.869862
		7	176.606249	164.980647	155.166302
		8	176.817798	164.785227	155.706183
		9	176.127218	165.609491	155.299736
		10	177.289888	165.137801	154.368744
		11	176.746563	165.421925	155.466852
		12	176.585601	165.209947	154.552329
		13	176.886043	165.19239	155.852911
	1	1	176.218266	165.189284	156.1974
		2	176.64818	165.153661	156.122368
		3	176.854454	164.401019	155.223516
		4	176.122239	165.059249	155.941556
		5	177.323671	165.650862	154.548435
		6	176.635827	165.917104	154.741073
		7	176.363159	165.005346	155.115022
		8	176.665168	164.659139	155.726285
		9	175.934241	165.656137	155.224316
		10	177.389034	164.981772	154.461115
		11	176.518909	165.568955	155.456919
		12	176.108067	164.995153	154.83462
		13	176.80844	165.062676	155.890714
	1.14	1	175.881334	165.046601	156.173641
		2	176.316145	164.64698	156.427882
		3	176.947299	164.802306	155.764048
		4	176.002227	164.971239	156.054688

		5	177.537243	165.935569	155.39615
		6	176.407814	165.804231	155.053145
		7	175.679804	164.553811	155.835925
		8	176.794774	164.434252	155.470011
		9	175.735582	166.237211	155.348727
		10	178.028754	165.803326	154.400817
		11	176.041215	165.684751	155.608299
		12	175.080847	164.779608	153.979047
		13	176.869491	165.105984	155.798856
MR_no	1.19	1	174.856003	166.605	143.112
		2	178.112	161.611	159.565
		3	177.886993	160.835	154.526
		4	173.509003	168.998	152.148
		5	176.470993	167.206	141.772
		6	175.899994	169.316	147.131
		7	174.210999	164.868	164.326
		8	177.617996	161.906	158.052
		9	173.289993	171.648	160.337
		10	176.501999	168.583	150.751
		11	173.681	165.068	147.742
		12	174.518005	164.062	151.243
		13	175.163	161.163	156.024
MR	1.18	1	175.810623	167.100906	143.686722
		2	178.513336	163.792206	161.601395
		3	178.02243	162.456589	154.01709
		4	174.500381	170.092331	147.913666
		5	177.040894	167.177765	144.67337
		6	176.782944	168.609787	145.217606
		7	177.388947	165.026886	160.825714
		8	178.222198	159.809402	159.616684
		9	174.394653	170.768875	161.057968
		10	176.786514	168.786423	151.065598
		11	174.37265	165.532608	148.197205
		12	174.837341	165.286118	151.017212
		13	175.512939	164.05719	155.847473

Neutron Diffraction	N/A	1	177.4	165.1	155.4
		2	177.6	164.8	155.7
		3	177.6	165.4	155.7
		4	177.9	165.1	155.6
		5	178.1	164.9	155.5
		6	177.8	165.3	155.5
		7	177.8	165.0	155.5
		8	177.5	165.0	155.4
		9	178.1	165.2	155.8
		10	177.7	165.4	155.6
		11	177.7	164.9	155.9
		12	177.7	165.6	155.4
		13	178.0	164.9	155.5
		14	177.9	165.2	155.7

Method	Resolution [Å $^{-1}$]	nr set	C1 - O1 - H1 ls error	O1 - C1 - C1 ls error	O2 - C1 - C1 ls error	O2 - C1 - O1 ls error	H2 - O3 - H3 ls error
IAM	0.625	1	1.36	0.09	0.09	0.1	1.87
		2	1.36	0.09	0.09	0.1	1.87
		3	1.36	0.09	0.09	0.1	1.87
		4	1.36	0.09	0.09	0.1	1.87
		5	1.36	0.09	0.09	0.1	1.87
		6	1.36	0.09	0.09	0.1	1.87
		7	1.36	0.09	0.09	0.1	1.87
		8	1.36	0.09	0.09	0.1	1.87
		9	1.36	0.09	0.09	0.1	1.87
		10	1.36	0.09	0.09	0.1	1.87
		11	1.36	0.09	0.09	0.1	1.87
		12	1.36	0.09	0.09	0.1	1.87
		13	1.36	0.09	0.09	0.1	1.87
	0.71429	1	1.24	0.07	0.07	0.08	1.41
		2	1.24	0.07	0.07	0.08	1.41
		3	1.24	0.07	0.07	0.08	1.41
		4	1.24	0.07	0.07	0.08	1.41
		5	1.24	0.07	0.07	0.08	1.41
		6	1.24	0.07	0.07	0.08	1.41
		7	1.24	0.07	0.07	0.08	1.41
		8	1.24	0.07	0.07	0.08	1.41
		9	1.24	0.07	0.07	0.08	1.41
		10	1.24	0.07	0.07	0.08	1.41
		11	1.24	0.07	0.07	0.08	1.41
		12	1.24	0.07	0.07	0.08	1.41
		13	1.24	0.07	0.07	0.08	1.41
	0.83333	1	1.16	0.05	0.05	0.06	1.33
		2	1.16	0.05	0.05	0.06	1.33
		3	1.16	0.05	0.05	0.06	1.33
		4	1.16	0.05	0.05	0.06	1.33

	5	1.16	0.05	0.05	0.06	1.33
	6	1.16	0.05	0.05	0.06	1.33
	7	1.16	0.05	0.05	0.06	1.33
	8	1.16	0.05	0.05	0.06	1.33
	9	1.16	0.05	0.05	0.06	1.33
	10	1.16	0.05	0.05	0.06	1.33
	11	1.16	0.05	0.05	0.06	1.33
	12	1.16	0.05	0.05	0.06	1.33
	13	1.16	0.05	0.05	0.06	1.33
1	1	0.95	0.04	0.04	0.05	1.1
	2	0.95	0.04	0.04	0.05	1.1
	3	0.95	0.04	0.04	0.05	1.1
	4	0.95	0.04	0.04	0.05	1.1
	5	0.95	0.04	0.04	0.05	1.1
	6	0.95	0.04	0.04	0.05	1.1
	7	0.95	0.04	0.04	0.05	1.1
	8	0.95	0.04	0.04	0.05	1.1
	9	0.95	0.04	0.04	0.05	1.1
	10	0.95	0.04	0.04	0.05	1.1
	11	0.95	0.04	0.04	0.05	1.1
	12	0.95	0.04	0.04	0.05	1.1
	13	0.95	0.04	0.04	0.05	1.1
1.14	1	0.87	0.04	0.04	0.05	0.99
	2	0.87	0.04	0.04	0.05	0.99
	3	0.87	0.04	0.04	0.05	0.99
	4	0.87	0.04	0.04	0.05	0.99
	5	0.87	0.04	0.04	0.05	0.99
	6	0.87	0.04	0.04	0.05	0.99
	7	0.87	0.04	0.04	0.05	0.99
	8	0.87	0.04	0.04	0.05	0.99
	9	0.87	0.04	0.04	0.05	0.99
	10	0.87	0.04	0.04	0.05	0.99
	11	0.87	0.04	0.04	0.05	0.99
	12	0.87	0.04	0.04	0.05	0.99
	13	0.87	0.04	0.04	0.05	0.99

UBDB	0.625	1	0.78	0.06	0.06	0.07	0.78
		2	0.78	0.06	0.06	0.07	0.78
		3	0.78	0.06	0.06	0.07	0.78
		4	0.78	0.06	0.06	0.07	0.78
		5	0.78	0.06	0.06	0.07	0.78
		6	0.78	0.06	0.06	0.07	0.78
		7	0.78	0.06	0.06	0.07	0.78
		8	0.78	0.06	0.06	0.07	0.78
		9	0.78	0.06	0.06	0.07	0.78
		10	0.78	0.06	0.06	0.07	0.78
		11	0.78	0.06	0.06	0.07	0.78
		12	0.78	0.06	0.06	0.07	0.78
		13	0.78	0.06	0.06	0.07	0.78
	0.71429	1	0.8	0.04	0.05	0.05	0.89
		2	0.8	0.04	0.05	0.05	0.89
		3	0.8	0.04	0.05	0.05	0.89
		4	0.8	0.04	0.05	0.05	0.89
		5	0.8	0.04	0.05	0.05	0.89
		6	0.8	0.04	0.05	0.05	0.89
		7	0.8	0.04	0.05	0.05	0.89
		8	0.8	0.04	0.05	0.05	0.89
		9	0.8	0.04	0.05	0.05	0.89
		10	0.8	0.04	0.05	0.05	0.89
		11	0.8	0.04	0.05	0.05	0.89
		12	0.8	0.04	0.05	0.05	0.89
		13	0.8	0.04	0.05	0.05	0.89
	0.83333	1	0.75	0.03	0.04	0.04	0.83
		2	0.75	0.03	0.04	0.04	0.83
		3	0.75	0.03	0.04	0.04	0.83
		4	0.75	0.03	0.04	0.04	0.83
		5	0.75	0.03	0.04	0.04	0.83
		6	0.75	0.03	0.04	0.04	0.83
		7	0.75	0.03	0.04	0.04	0.83
		8	0.75	0.03	0.04	0.04	0.83
		9	0.75	0.03	0.04	0.04	0.83

		10	0.75	0.03	0.04	0.04	0.83
		11	0.75	0.03	0.04	0.04	0.83
		12	0.75	0.03	0.04	0.04	0.83
		13	0.75	0.03	0.04	0.04	0.83
	1	1	0.73	0.03	0.03	0.03	0.76
		2	0.73	0.03	0.03	0.03	0.76
		3	0.73	0.03	0.03	0.03	0.76
		4	0.73	0.03	0.03	0.03	0.76
		5	0.73	0.03	0.03	0.03	0.76
		6	0.73	0.03	0.03	0.03	0.76
		7	0.73	0.03	0.03	0.03	0.76
		8	0.73	0.03	0.03	0.03	0.76
		9	0.73	0.03	0.03	0.03	0.76
		10	0.73	0.03	0.03	0.03	0.76
		11	0.73	0.03	0.03	0.03	0.76
		12	0.73	0.03	0.03	0.03	0.76
		13	0.73	0.03	0.03	0.03	0.76
	1.14	1	0.7	0.03	0.03	0.03	0.73
		2	0.7	0.03	0.03	0.03	0.73
		3	0.7	0.03	0.03	0.03	0.73
		4	0.7	0.03	0.03	0.03	0.73
		5	0.7	0.03	0.03	0.03	0.73
		6	0.7	0.03	0.03	0.03	0.73
		7	0.7	0.03	0.03	0.03	0.73
		8	0.7	0.03	0.03	0.03	0.73
		9	0.7	0.03	0.03	0.03	0.73
		10	0.7	0.03	0.03	0.03	0.73
		11	0.7	0.03	0.03	0.03	0.73
		12	0.7	0.03	0.03	0.03	0.73
		13	0.7	0.03	0.03	0.03	0.73
ELMAM2	0.625	1	0.76	0.05	0.06	0.06	0.71
		2	0.76	0.05	0.06	0.06	0.71
		3	0.76	0.05	0.06	0.06	0.71
		4	0.76	0.05	0.06	0.06	0.71
		5	0.76	0.05	0.06	0.06	0.71

	6	0.76	0.05	0.06	0.06	0.71
	7	0.76	0.05	0.06	0.06	0.71
	8	0.76	0.05	0.06	0.06	0.71
	9	0.76	0.05	0.06	0.06	0.71
	10	0.76	0.05	0.06	0.06	0.71
	11	0.76	0.05	0.06	0.06	0.71
	12	0.76	0.05	0.06	0.06	0.71
	13	0.76	0.05	0.06	0.06	0.71
0.71429	1	0.8	0.04	0.05	0.05	0.78
	2	0.8	0.04	0.05	0.05	0.78
	3	0.8	0.04	0.05	0.05	0.78
	4	0.8	0.04	0.05	0.05	0.78
	5	0.8	0.04	0.05	0.05	0.78
	6	0.8	0.04	0.05	0.05	0.78
	7	0.8	0.04	0.05	0.05	0.78
	8	0.8	0.04	0.05	0.05	0.78
	9	0.8	0.04	0.05	0.05	0.78
	10	0.8	0.04	0.05	0.05	0.78
	11	0.8	0.04	0.05	0.05	0.78
	12	0.8	0.04	0.05	0.05	0.78
	13	0.8	0.04	0.05	0.05	0.78
0.83333	1	0.77	0.03	0.04	0.04	0.75
	2	0.77	0.03	0.04	0.04	0.75
	3	0.77	0.03	0.04	0.04	0.75
	4	0.77	0.03	0.04	0.04	0.75
	5	0.77	0.03	0.04	0.04	0.75
	6	0.77	0.03	0.04	0.04	0.75
	7	0.77	0.03	0.04	0.04	0.75
	8	0.77	0.03	0.04	0.04	0.75
	9	0.77	0.03	0.04	0.04	0.75
	10	0.77	0.03	0.04	0.04	0.75
	11	0.77	0.03	0.04	0.04	0.75
	12	0.77	0.03	0.04	0.04	0.75
	13	0.77	0.03	0.04	0.04	0.75
1	1	0.53	0.03	0.03	0.03	0.62

		2	0.53	0.03	0.03	0.03	0.62
		3	0.53	0.03	0.03	0.03	0.62
		4	0.53	0.03	0.03	0.03	0.62
		5	0.53	0.03	0.03	0.03	0.62
		6	0.53	0.03	0.03	0.03	0.62
		7	0.53	0.03	0.03	0.03	0.62
		8	0.53	0.03	0.03	0.03	0.62
		9	0.53	0.03	0.03	0.03	0.62
		10	0.53	0.03	0.03	0.03	0.62
		11	0.53	0.03	0.03	0.03	0.62
		12	0.53	0.03	0.03	0.03	0.62
		13	0.53	0.03	0.03	0.03	0.62
	1.14	1	0.74	0.03	0.03	0.03	0.68
		2	0.74	0.03	0.03	0.03	0.68
		3	0.74	0.03	0.03	0.03	0.68
		4	0.74	0.03	0.03	0.03	0.68
		5	0.74	0.03	0.03	0.03	0.68
		6	0.74	0.03	0.03	0.03	0.68
		7	0.74	0.03	0.03	0.03	0.68
		8	0.74	0.03	0.03	0.03	0.68
		9	0.74	0.03	0.03	0.03	0.68
		10	0.74	0.03	0.03	0.03	0.68
		11	0.74	0.03	0.03	0.03	0.68
		12	0.74	0.03	0.03	0.03	0.68
		13	0.74	0.03	0.03	0.03	0.68
GID	0.625	1	0.76	0.05	0.06	0.06	0.71
		2	0.76	0.05	0.06	0.06	0.71
		3	0.76	0.05	0.06	0.06	0.71
		4	0.76	0.05	0.06	0.06	0.71
		5	0.76	0.05	0.06	0.06	0.71
		6	0.76	0.05	0.06	0.06	0.71
		7	0.76	0.05	0.06	0.06	0.71
		8	0.76	0.05	0.06	0.06	0.71
		9	0.76	0.05	0.06	0.06	0.71
		10	0.76	0.05	0.06	0.06	0.71

		11	0.76	0.05	0.06	0.06	0.71
		12	0.76	0.05	0.06	0.06	0.71
		13	0.76	0.05	0.06	0.06	0.71
	0.71429	1	0.8	0.04	0.05	0.05	0.78
		2	0.8	0.04	0.05	0.05	0.78
		3	0.8	0.04	0.05	0.05	0.78
		4	0.8	0.04	0.05	0.05	0.78
		5	0.8	0.04	0.05	0.05	0.78
		6	0.8	0.04	0.05	0.05	0.78
		7	0.8	0.04	0.05	0.05	0.78
		8	0.8	0.04	0.05	0.05	0.78
		9	0.8	0.04	0.05	0.05	0.78
		10	0.8	0.04	0.05	0.05	0.78
		11	0.8	0.04	0.05	0.05	0.78
		12	0.8	0.04	0.05	0.05	0.78
		13	0.8	0.04	0.05	0.05	0.78
	0.83333	1	0.77	0.03	0.04	0.04	0.75
		2	0.77	0.03	0.04	0.04	0.75
		3	0.77	0.03	0.04	0.04	0.75
		4	0.77	0.03	0.04	0.04	0.75
		5	0.77	0.03	0.04	0.04	0.75
		6	0.77	0.03	0.04	0.04	0.75
		7	0.77	0.03	0.04	0.04	0.75
		8	0.77	0.03	0.04	0.04	0.75
		9	0.77	0.03	0.04	0.04	0.75
		10	0.77	0.03	0.04	0.04	0.75
		11	0.77	0.03	0.04	0.04	0.75
		12	0.77	0.03	0.04	0.04	0.75
		13	0.77	0.03	0.04	0.04	0.75
	1	1	0.75	0.03	0.03	0.03	0.7
		2	0.75	0.03	0.03	0.03	0.7
		3	0.75	0.03	0.03	0.03	0.7
		4	0.75	0.03	0.03	0.03	0.7
		5	0.75	0.03	0.03	0.03	0.7
		6	0.75	0.03	0.03	0.03	0.7

		7	0.75	0.03	0.03	0.03	0.7
		8	0.75	0.03	0.03	0.03	0.7
		9	0.75	0.03	0.03	0.03	0.7
		10	0.75	0.03	0.03	0.03	0.7
		11	0.75	0.03	0.03	0.03	0.7
		12	0.75	0.03	0.03	0.03	0.7
		13	0.75	0.03	0.03	0.03	0.7
	1.14	1	0.74	0.03	0.03	0.03	0.68
		2	0.74	0.03	0.03	0.03	0.68
		3	0.74	0.03	0.03	0.03	0.68
		4	0.74	0.03	0.03	0.03	0.68
		5	0.74	0.03	0.03	0.03	0.68
		6	0.74	0.03	0.03	0.03	0.68
		7	0.74	0.03	0.03	0.03	0.68
		8	0.74	0.03	0.03	0.03	0.68
		9	0.74	0.03	0.03	0.03	0.68
		10	0.74	0.03	0.03	0.03	0.68
		11	0.74	0.03	0.03	0.03	0.68
		12	0.74	0.03	0.03	0.03	0.68
		13	0.74	0.03	0.03	0.03	0.68
HAR(B3LYP)	0.625	1	0.645314	0.054771	0.053164	0.039961	0.799657
		2	0.645314	0.054771	0.053164	0.039961	0.799657
		3	0.645314	0.054771	0.053164	0.039961	0.799657
		4	0.645314	0.054771	0.053164	0.039961	0.799657
		5	0.645314	0.054771	0.053164	0.039961	0.799657
		6	0.645314	0.054771	0.053164	0.039961	0.799657
		7	0.645314	0.054771	0.053164	0.039961	0.799657
		8	0.645314	0.054771	0.053164	0.039961	0.799657
		9	0.645314	0.054771	0.053164	0.039961	0.799657
		10	0.645314	0.054771	0.053164	0.039961	0.799657
		11	0.645314	0.054771	0.053164	0.039961	0.799657
		12	0.645314	0.054771	0.053164	0.039961	0.799657
		13	0.645314	0.054771	0.053164	0.039961	0.799657
	0.71429	1	0.623316	0.040474	0.038938	0.029951	0.780164
		2	0.623316	0.040474	0.038938	0.029951	0.780164

	3	0.623316	0.040474	0.038938	0.029951	0.780164
	4	0.623316	0.040474	0.038938	0.029951	0.780164
	5	0.623316	0.040474	0.038938	0.029951	0.780164
	6	0.623316	0.040474	0.038938	0.029951	0.780164
	7	0.623316	0.040474	0.038938	0.029951	0.780164
	8	0.623316	0.040474	0.038938	0.029951	0.780164
	9	0.623316	0.040474	0.038938	0.029951	0.780164
	10	0.623316	0.040474	0.038938	0.029951	0.780164
	11	0.623316	0.040474	0.038938	0.029951	0.780164
	12	0.623316	0.040474	0.038938	0.029951	0.780164
	13	0.623316	0.040474	0.038938	0.029951	0.780164
0.83333	1	0.592792	0.029863	0.028755	0.023533	0.6982
	2	0.592792	0.029863	0.028755	0.023533	0.6982
	3	0.592792	0.029863	0.028755	0.023533	0.6982
	4	0.592792	0.029863	0.028755	0.023533	0.6982
	5	0.592792	0.029863	0.028755	0.023533	0.6982
	6	0.592792	0.029863	0.028755	0.023533	0.6982
	7	0.592792	0.029863	0.028755	0.023533	0.6982
	8	0.592792	0.029863	0.028755	0.023533	0.6982
	9	0.592792	0.029863	0.028755	0.023533	0.6982
	10	0.592792	0.029863	0.028755	0.023533	0.6982
	11	0.592792	0.029863	0.028755	0.023533	0.6982
	12	0.592792	0.029863	0.028755	0.023533	0.6982
	13	0.592792	0.029863	0.028755	0.023533	0.6982
1	1	0.477917	0.020714	0.021137	0.019569	0.627604
	2	0.477917	0.020714	0.021137	0.019569	0.627604
	3	0.477917	0.020714	0.021137	0.019569	0.627604
	4	0.477917	0.020714	0.021137	0.019569	0.627604
	5	0.477917	0.020714	0.021137	0.019569	0.627604
	6	0.477917	0.020714	0.021137	0.019569	0.627604
	7	0.477917	0.020714	0.021137	0.019569	0.627604
	8	0.477917	0.020714	0.021137	0.019569	0.627604
	9	0.477917	0.020714	0.021137	0.019569	0.627604
	10	0.477917	0.020714	0.021137	0.019569	0.627604
	11	0.477917	0.020714	0.021137	0.019569	0.627604

		12	0.477917	0.020714	0.021137	0.019569	0.627604
		13	0.477917	0.020714	0.021137	0.019569	0.627604
	1.14	1	0.423685	0.023024	0.023715	0.02083	0.646293
		2	0.423685	0.023024	0.023715	0.02083	0.646293
		3	0.423685	0.023024	0.023715	0.02083	0.646293
		4	0.423685	0.023024	0.023715	0.02083	0.646293
		5	0.423685	0.023024	0.023715	0.02083	0.646293
		6	0.423685	0.023024	0.023715	0.02083	0.646293
		7	0.423685	0.023024	0.023715	0.02083	0.646293
		8	0.423685	0.023024	0.023715	0.02083	0.646293
		9	0.423685	0.023024	0.023715	0.02083	0.646293
		10	0.423685	0.023024	0.023715	0.02083	0.646293
		11	0.423685	0.023024	0.023715	0.02083	0.646293
		12	0.423685	0.023024	0.023715	0.02083	0.646293
		13	0.423685	0.023024	0.023715	0.02083	0.646293
HAR(BLYP)	0.625	1	0.582375	0.041237	0.042412	0.035601	0.747607
		2	0.582375	0.041237	0.042412	0.035601	0.747607
		3	0.582375	0.041237	0.042412	0.035601	0.747607
		4	0.582375	0.041237	0.042412	0.035601	0.747607
		5	0.582375	0.041237	0.042412	0.035601	0.747607
		6	0.582375	0.041237	0.042412	0.035601	0.747607
		7	0.582375	0.041237	0.042412	0.035601	0.747607
		8	0.582375	0.041237	0.042412	0.035601	0.747607
		9	0.582375	0.041237	0.042412	0.035601	0.747607
		10	0.582375	0.041237	0.042412	0.035601	0.747607
		11	0.582375	0.041237	0.042412	0.035601	0.747607
		12	0.582375	0.041237	0.042412	0.035601	0.747607
		13	0.582375	0.041237	0.042412	0.035601	0.747607
	0.71429	1	0.583371	0.038718	0.039041	0.033617	0.776036
		2	0.583371	0.038718	0.039041	0.033617	0.776036
		3	0.583371	0.038718	0.039041	0.033617	0.776036
		4	0.583371	0.038718	0.039041	0.033617	0.776036
		5	0.583371	0.038718	0.039041	0.033617	0.776036
		6	0.583371	0.038718	0.039041	0.033617	0.776036
		7	0.583371	0.038718	0.039041	0.033617	0.776036

		8	0.583371	0.038718	0.039041	0.033617	0.776036
		9	0.583371	0.038718	0.039041	0.033617	0.776036
		10	0.583371	0.038718	0.039041	0.033617	0.776036
		11	0.583371	0.038718	0.039041	0.033617	0.776036
		12	0.583371	0.038718	0.039041	0.033617	0.776036
		13	0.583371	0.038718	0.039041	0.033617	0.776036
	0.83333	1	0.475303	0.029951	0.030528	0.026155	0.728046
		2	0.475303	0.029951	0.030528	0.026155	0.728046
		3	0.475303	0.029951	0.030528	0.026155	0.728046
		4	0.475303	0.029951	0.030528	0.026155	0.728046
		5	0.475303	0.029951	0.030528	0.026155	0.728046
		6	0.475303	0.029951	0.030528	0.026155	0.728046
		7	0.475303	0.029951	0.030528	0.026155	0.728046
		8	0.475303	0.029951	0.030528	0.026155	0.728046
		9	0.475303	0.029951	0.030528	0.026155	0.728046
		10	0.475303	0.029951	0.030528	0.026155	0.728046
		11	0.475303	0.029951	0.030528	0.026155	0.728046
		12	0.475303	0.029951	0.030528	0.026155	0.728046
		13	0.475303	0.029951	0.030528	0.026155	0.728046
	1	1	0.435613	0.024272	0.024764	0.020848	0.67796
		2	0.435613	0.024272	0.024764	0.020848	0.67796
		3	0.435613	0.024272	0.024764	0.020848	0.67796
		4	0.435613	0.024272	0.024764	0.020848	0.67796
		5	0.435613	0.024272	0.024764	0.020848	0.67796
		6	0.435613	0.024272	0.024764	0.020848	0.67796
		7	0.435613	0.024272	0.024764	0.020848	0.67796
		8	0.435613	0.024272	0.024764	0.020848	0.67796
		9	0.435613	0.024272	0.024764	0.020848	0.67796
		10	0.435613	0.024272	0.024764	0.020848	0.67796
		11	0.435613	0.024272	0.024764	0.020848	0.67796
		12	0.435613	0.024272	0.024764	0.020848	0.67796
		13	0.435613	0.024272	0.024764	0.020848	0.67796
	1.14	1	0.426893	0.02333	0.024021	0.021089	0.657686
		2	0.426893	0.02333	0.024021	0.021089	0.657686
		3	0.426893	0.02333	0.024021	0.021089	0.657686

		4	0.426893	0.02333	0.024021	0.021089	0.657686
		5	0.426893	0.02333	0.024021	0.021089	0.657686
		6	0.426893	0.02333	0.024021	0.021089	0.657686
		7	0.426893	0.02333	0.024021	0.021089	0.657686
		8	0.426893	0.02333	0.024021	0.021089	0.657686
		9	0.426893	0.02333	0.024021	0.021089	0.657686
		10	0.426893	0.02333	0.024021	0.021089	0.657686
		11	0.426893	0.02333	0.024021	0.021089	0.657686
		12	0.426893	0.02333	0.024021	0.021089	0.657686
		13	0.426893	0.02333	0.024021	0.021089	0.657686
MR_no	1.19	1	2.4	0.03	0.04	0.05	3.43
		2	2.4	0.03	0.04	0.05	3.43
		3	2.4	0.03	0.04	0.05	3.43
		4	2.4	0.03	0.04	0.05	3.43
		5	2.4	0.03	0.04	0.05	3.43
		6	2.4	0.03	0.04	0.05	3.43
		7	2.4	0.03	0.04	0.05	3.43
		8	2.4	0.03	0.04	0.05	3.43
		9	2.4	0.03	0.04	0.05	3.43
		10	2.4	0.03	0.04	0.05	3.43
		11	2.4	0.03	0.04	0.05	3.43
		12	2.4	0.03	0.04	0.05	3.43
		13	2.4	0.03	0.04	0.05	3.43
MR	1.18	1	0.908263	0.034685	0.037693	0.045365	2.025433
		2	0.908263	0.034685	0.037693	0.045365	2.025433
		3	0.908263	0.034685	0.037693	0.045365	2.025433
		4	0.908263	0.034685	0.037693	0.045365	2.025433
		5	0.908263	0.034685	0.037693	0.045365	2.025433
		6	0.908263	0.034685	0.037693	0.045365	2.025433
		7	0.908263	0.034685	0.037693	0.045365	2.025433
		8	0.908263	0.034685	0.037693	0.045365	2.025433
		9	0.908263	0.034685	0.037693	0.045365	2.025433
		10	0.908263	0.034685	0.037693	0.045365	2.025433
		11	0.908263	0.034685	0.037693	0.045365	2.025433
		12	0.908263	0.034685	0.037693	0.045365	2.025433

		13	0.908263	0.034685	0.037693	0.045365	2.025433
Neutron Diffraction	N/A	1	0.2	0.13	0.12	0.12	0.3
		2	0.2	0.13	0.12	0.13	0.3
		3	0.2	0.13	0.12	0.12	0.3
		4	0.2	0.12	0.11	0.12	0.3
		5	0.2	0.12	0.11	0.12	0.3
		6	0.2	0.13	0.12	0.13	0.3
		7	0.3	0.14	0.13	0.14	0.3
		8	0.2	0.14	0.13	0.13	0.3
		9	0.2	0.12	0.11	0.12	0.3
		10	0.2	0.14	0.13	0.14	0.3
		11	0.2	0.13	0.13	0.13	0.3
		12	0.2	0.13	0.12	0.13	0.3
		13	0.2	0.13	0.12	0.13	0.3
		14	0.2	0.12	0.11	0.12	0.3

Method	Resolution [Å $^{-1}$]	nr set	O1 - H1 - O3 ls error	(ii) x, 1.5-y, 1/2+z O3 - H2 - O2ii ls error	(iii) 1-x, -1/2+y, 1/2-z O3 - H3 - O2iii ls error
IAM	0.625	1	2.09	2.32	1.91
		2	2.09	2.32	1.91
		3	2.09	2.32	1.91
		4	2.09	2.32	1.91
		5	2.09	2.32	1.91
		6	2.09	2.32	1.91
		7	2.09	2.32	1.91
		8	2.09	2.32	1.91
		9	2.09	2.32	1.91
		10	2.09	2.32	1.91
		11	2.09	2.32	1.91
		12	2.09	2.32	1.91
		13	2.09	2.32	1.91
	0.71429	1	1.88	1.6	1.43
		2	1.88	1.6	1.43
		3	1.88	1.6	1.43
		4	1.88	1.6	1.43
		5	1.88	1.6	1.43
		6	1.88	1.6	1.43
		7	1.88	1.6	1.43
		8	1.88	1.6	1.43
		9	1.88	1.6	1.43
		10	1.88	1.6	1.43
		11	1.88	1.6	1.43
		12	1.88	1.6	1.43
		13	1.88	1.6	1.43
	0.83333	1	1.71	1.46	1.32
		2	1.71	1.46	1.32
		3	1.71	1.46	1.32
		4	1.71	1.46	1.32

	5	1.71	1.46	1.32
	6	1.71	1.46	1.32
	7	1.71	1.46	1.32
	8	1.71	1.46	1.32
	9	1.71	1.46	1.32
	10	1.71	1.46	1.32
	11	1.71	1.46	1.32
	12	1.71	1.46	1.32
	13	1.71	1.46	1.32
1	1	1.39	1.21	1.08
	2	1.39	1.21	1.08
	3	1.39	1.21	1.08
	4	1.39	1.21	1.08
	5	1.39	1.21	1.08
	6	1.39	1.21	1.08
	7	1.39	1.21	1.08
	8	1.39	1.21	1.08
	9	1.39	1.21	1.08
	10	1.39	1.21	1.08
	11	1.39	1.21	1.08
	12	1.39	1.21	1.08
	13	1.39	1.21	1.08
1.14	1	1.33	1.08	0.97
	2	1.33	1.08	0.97
	3	1.33	1.08	0.97
	4	1.33	1.08	0.97
	5	1.33	1.08	0.97
	6	1.33	1.08	0.97
	7	1.33	1.08	0.97
	8	1.33	1.08	0.97
	9	1.33	1.08	0.97
	10	1.33	1.08	0.97
	11	1.33	1.08	0.97
	12	1.33	1.08	0.97
	13	1.33	1.08	0.97

UBDB	0.625	1	1.35	1.14	0.87
		2	1.35	1.14	0.87
		3	1.35	1.14	0.87
		4	1.35	1.14	0.87
		5	1.35	1.14	0.87
		6	1.35	1.14	0.87
		7	1.35	1.14	0.87
		8	1.35	1.14	0.87
		9	1.35	1.14	0.87
		10	1.35	1.14	0.87
		11	1.35	1.14	0.87
		12	1.35	1.14	0.87
		13	1.35	1.14	0.87
	0.71429	1	1.31	1.05	0.92
		2	1.31	1.05	0.92
		3	1.31	1.05	0.92
		4	1.31	1.05	0.92
		5	1.31	1.05	0.92
		6	1.31	1.05	0.92
		7	1.31	1.05	0.92
		8	1.31	1.05	0.92
		9	1.31	1.05	0.92
		10	1.31	1.05	0.92
		11	1.31	1.05	0.92
		12	1.31	1.05	0.92
		13	1.31	1.05	0.92
	0.83333	1	1.18	0.99	0.81
		2	1.18	0.99	0.81
		3	1.18	0.99	0.81
		4	1.18	0.99	0.81
		5	1.18	0.99	0.81
		6	1.18	0.99	0.81
		7	1.18	0.99	0.81
		8	1.18	0.99	0.81
		9	1.18	0.99	0.81

		10	1.18	0.99	0.81
		11	1.18	0.99	0.81
		12	1.18	0.99	0.81
		13	1.18	0.99	0.81
	1	1	1.11	0.91	0.74
		2	1.11	0.91	0.74
		3	1.11	0.91	0.74
		4	1.11	0.91	0.74
		5	1.11	0.91	0.74
		6	1.11	0.91	0.74
		7	1.11	0.91	0.74
		8	1.11	0.91	0.74
		9	1.11	0.91	0.74
		10	1.11	0.91	0.74
		11	1.11	0.91	0.74
		12	1.11	0.91	0.74
		13	1.11	0.91	0.74
	1.14	1	1.07	0.88	0.7
		2	1.07	0.88	0.7
		3	1.07	0.88	0.7
		4	1.07	0.88	0.7
		5	1.07	0.88	0.7
		6	1.07	0.88	0.7
		7	1.07	0.88	0.7
		8	1.07	0.88	0.7
		9	1.07	0.88	0.7
		10	1.07	0.88	0.7
		11	1.07	0.88	0.7
		12	1.07	0.88	0.7
		13	1.07	0.88	0.7
ELMAM2	0.625	1	1.29	1.06	0.83
		2	1.29	1.06	0.83
		3	1.29	1.06	0.83
		4	1.29	1.06	0.83
		5	1.29	1.06	0.83

	6	1.29	1.06	0.83
	7	1.29	1.06	0.83
	8	1.29	1.06	0.83
	9	1.29	1.06	0.83
	10	1.29	1.06	0.83
	11	1.29	1.06	0.83
	12	1.29	1.06	0.83
	13	1.29	1.06	0.83
0.71429	1	1.29	0.98	0.83
	2	1.29	0.98	0.83
	3	1.29	0.98	0.83
	4	1.29	0.98	0.83
	5	1.29	0.98	0.83
	6	1.29	0.98	0.83
	7	1.29	0.98	0.83
	8	1.29	0.98	0.83
	9	1.29	0.98	0.83
	10	1.29	0.98	0.83
	11	1.29	0.98	0.83
	12	1.29	0.98	0.83
	13	1.29	0.98	0.83
0.83333	1	1.22	0.94	0.75
	2	1.22	0.94	0.75
	3	1.22	0.94	0.75
	4	1.22	0.94	0.75
	5	1.22	0.94	0.75
	6	1.22	0.94	0.75
	7	1.22	0.94	0.75
	8	1.22	0.94	0.75
	9	1.22	0.94	0.75
	10	1.22	0.94	0.75
	11	1.22	0.94	0.75
	12	1.22	0.94	0.75
	13	1.22	0.94	0.75
1	1	0.96	0.83	0.74

		2	0.96	0.83	0.74
		3	0.96	0.83	0.74
		4	0.96	0.83	0.74
		5	0.96	0.83	0.74
		6	0.96	0.83	0.74
		7	0.96	0.83	0.74
		8	0.96	0.83	0.74
		9	0.96	0.83	0.74
		10	0.96	0.83	0.74
		11	0.96	0.83	0.74
		12	0.96	0.83	0.74
		13	0.96	0.83	0.74
	1.14	1	1.15	0.86	0.67
		2	1.15	0.86	0.67
		3	1.15	0.86	0.67
		4	1.15	0.86	0.67
		5	1.15	0.86	0.67
		6	1.15	0.86	0.67
		7	1.15	0.86	0.67
		8	1.15	0.86	0.67
		9	1.15	0.86	0.67
		10	1.15	0.86	0.67
		11	1.15	0.86	0.67
		12	1.15	0.86	0.67
		13	1.15	0.86	0.67
GID	0.625	1	1.29	1.06	0.83
		2	1.29	1.06	0.83
		3	1.29	1.06	0.83
		4	1.29	1.06	0.83
		5	1.29	1.06	0.83
		6	1.29	1.06	0.83
		7	1.29	1.06	0.83
		8	1.29	1.06	0.83
		9	1.29	1.06	0.83
		10	1.29	1.06	0.83

		11	1.29	1.06	0.83
		12	1.29	1.06	0.83
		13	1.29	1.06	0.83
	0.71429	1	1.29	0.98	0.83
		2	1.29	0.98	0.83
		3	1.29	0.98	0.83
		4	1.29	0.98	0.83
		5	1.29	0.98	0.83
		6	1.29	0.98	0.83
		7	1.29	0.98	0.83
		8	1.29	0.98	0.83
		9	1.29	0.98	0.83
		10	1.29	0.98	0.83
		11	1.29	0.98	0.83
		12	1.29	0.98	0.83
		13	1.29	0.98	0.83
	0.83333	1	1.22	0.94	0.75
		2	1.22	0.94	0.75
		3	1.22	0.94	0.75
		4	1.22	0.94	0.75
		5	1.22	0.94	0.75
		6	1.22	0.94	0.75
		7	1.22	0.94	0.75
		8	1.22	0.94	0.75
		9	1.22	0.94	0.75
		10	1.22	0.94	0.75
		11	1.22	0.94	0.75
		12	1.22	0.94	0.75
		13	1.22	0.94	0.75
	1	1	1.16	0.88	0.69
		2	1.16	0.88	0.69
		3	1.16	0.88	0.69
		4	1.16	0.88	0.69
		5	1.16	0.88	0.69
		6	1.16	0.88	0.69

		7	1.16	0.88	0.69
		8	1.16	0.88	0.69
		9	1.16	0.88	0.69
		10	1.16	0.88	0.69
		11	1.16	0.88	0.69
		12	1.16	0.88	0.69
		13	1.16	0.88	0.69
	1.14	1	1.15	0.86	0.67
		2	1.15	0.86	0.67
		3	1.15	0.86	0.67
		4	1.15	0.86	0.67
		5	1.15	0.86	0.67
		6	1.15	0.86	0.67
		7	1.15	0.86	0.67
		8	1.15	0.86	0.67
		9	1.15	0.86	0.67
		10	1.15	0.86	0.67
		11	1.15	0.86	0.67
		12	1.15	0.86	0.67
		13	1.15	0.86	0.67
HAR(B3LYP)	0.625	1	1.246538	0.949848	0.902023
		2	1.246538	0.949848	0.902023
		3	1.246538	0.949848	0.902023
		4	1.246538	0.949848	0.902023
		5	1.246538	0.949848	0.902023
		6	1.246538	0.949848	0.902023
		7	1.246538	0.949848	0.902023
		8	1.246538	0.949848	0.902023
		9	1.246538	0.949848	0.902023
		10	1.246538	0.949848	0.902023
		11	1.246538	0.949848	0.902023
		12	1.246538	0.949848	0.902023
		13	1.246538	0.949848	0.902023
	0.71429	1	1.152565	0.890206	0.835184
		2	1.152565	0.890206	0.835184

	3	1.152565	0.890206	0.835184
	4	1.152565	0.890206	0.835184
	5	1.152565	0.890206	0.835184
	6	1.152565	0.890206	0.835184
	7	1.152565	0.890206	0.835184
	8	1.152565	0.890206	0.835184
	9	1.152565	0.890206	0.835184
	10	1.152565	0.890206	0.835184
	11	1.152565	0.890206	0.835184
	12	1.152565	0.890206	0.835184
	13	1.152565	0.890206	0.835184
0.83333	1	1.094619	0.816606	0.795472
	2	1.094619	0.816606	0.795472
	3	1.094619	0.816606	0.795472
	4	1.094619	0.816606	0.795472
	5	1.094619	0.816606	0.795472
	6	1.094619	0.816606	0.795472
	7	1.094619	0.816606	0.795472
	8	1.094619	0.816606	0.795472
	9	1.094619	0.816606	0.795472
	10	1.094619	0.816606	0.795472
	11	1.094619	0.816606	0.795472
	12	1.094619	0.816606	0.795472
	13	1.094619	0.816606	0.795472
1	1	0.884307	0.677616	0.697646
	2	0.884307	0.677616	0.697646
	3	0.884307	0.677616	0.697646
	4	0.884307	0.677616	0.697646
	5	0.884307	0.677616	0.697646
	6	0.884307	0.677616	0.697646
	7	0.884307	0.677616	0.697646
	8	0.884307	0.677616	0.697646
	9	0.884307	0.677616	0.697646
	10	0.884307	0.677616	0.697646
	11	0.884307	0.677616	0.697646

		12	0.884307	0.677616	0.697646
		13	0.884307	0.677616	0.697646
	1.14	1	0.803172	0.734712	0.691706
		2	0.803172	0.734712	0.691706
		3	0.803172	0.734712	0.691706
		4	0.803172	0.734712	0.691706
		5	0.803172	0.734712	0.691706
		6	0.803172	0.734712	0.691706
		7	0.803172	0.734712	0.691706
		8	0.803172	0.734712	0.691706
		9	0.803172	0.734712	0.691706
		10	0.803172	0.734712	0.691706
		11	0.803172	0.734712	0.691706
		12	0.803172	0.734712	0.691706
		13	0.803172	0.734712	0.691706
HAR(BLYP)	0.625	1	1.192464	0.799532	0.805412
		2	1.192464	0.799532	0.805412
		3	1.192464	0.799532	0.805412
		4	1.192464	0.799532	0.805412
		5	1.192464	0.799532	0.805412
		6	1.192464	0.799532	0.805412
		7	1.192464	0.799532	0.805412
		8	1.192464	0.799532	0.805412
		9	1.192464	0.799532	0.805412
		10	1.192464	0.799532	0.805412
		11	1.192464	0.799532	0.805412
		12	1.192464	0.799532	0.805412
		13	1.192464	0.799532	0.805412
	0.71429	1	1.307168	0.872453	0.830748
		2	1.307168	0.872453	0.830748
		3	1.307168	0.872453	0.830748
		4	1.307168	0.872453	0.830748
		5	1.307168	0.872453	0.830748
		6	1.307168	0.872453	0.830748
		7	1.307168	0.872453	0.830748

		8	1.307168	0.872453	0.830748
		9	1.307168	0.872453	0.830748
		10	1.307168	0.872453	0.830748
		11	1.307168	0.872453	0.830748
		12	1.307168	0.872453	0.830748
		13	1.307168	0.872453	0.830748
	0.83333	1	0.836381	0.827571	0.765115
		2	0.836381	0.827571	0.765115
		3	0.836381	0.827571	0.765115
		4	0.836381	0.827571	0.765115
		5	0.836381	0.827571	0.765115
		6	0.836381	0.827571	0.765115
		7	0.836381	0.827571	0.765115
		8	0.836381	0.827571	0.765115
		9	0.836381	0.827571	0.765115
		10	0.836381	0.827571	0.765115
		11	0.836381	0.827571	0.765115
		12	0.836381	0.827571	0.765115
		13	0.836381	0.827571	0.765115
	1	1	0.791819	0.772062	0.707327
		2	0.791819	0.772062	0.707327
		3	0.791819	0.772062	0.707327
		4	0.791819	0.772062	0.707327
		5	0.791819	0.772062	0.707327
		6	0.791819	0.772062	0.707327
		7	0.791819	0.772062	0.707327
		8	0.791819	0.772062	0.707327
		9	0.791819	0.772062	0.707327
		10	0.791819	0.772062	0.707327
		11	0.791819	0.772062	0.707327
		12	0.791819	0.772062	0.707327
		13	0.791819	0.772062	0.707327
	1.14	1	0.80153	0.748866	0.691806
		2	0.80153	0.748866	0.691806
		3	0.80153	0.748866	0.691806

		4	0.80153	0.748866	0.691806
		5	0.80153	0.748866	0.691806
		6	0.80153	0.748866	0.691806
		7	0.80153	0.748866	0.691806
		8	0.80153	0.748866	0.691806
		9	0.80153	0.748866	0.691806
		10	0.80153	0.748866	0.691806
		11	0.80153	0.748866	0.691806
		12	0.80153	0.748866	0.691806
		13	0.80153	0.748866	0.691806
MR_no	1.19	1	4.4	1.58	2.18
		2	4.4	1.58	2.18
		3	4.4	1.58	2.18
		4	4.4	1.58	2.18
		5	4.4	1.58	2.18
		6	4.4	1.58	2.18
		7	4.4	1.58	2.18
		8	4.4	1.58	2.18
		9	4.4	1.58	2.18
		10	4.4	1.58	2.18
		11	4.4	1.58	2.18
		12	4.4	1.58	2.18
		13	4.4	1.58	2.18
MR	1.18	1	0.508791	0.59813	1.425084
		2	0.508791	0.59813	1.425084
		3	0.508791	0.59813	1.425084
		4	0.508791	0.59813	1.425084
		5	0.508791	0.59813	1.425084
		6	0.508791	0.59813	1.425084
		7	0.508791	0.59813	1.425084
		8	0.508791	0.59813	1.425084
		9	0.508791	0.59813	1.425084
		10	0.508791	0.59813	1.425084
		11	0.508791	0.59813	1.425084
		12	0.508791	0.59813	1.425084

		13	0.508791	0.59813	1.425084
Neutron Diffraction	N/A	1	0.3	0.3	0.3
		2	0.3	0.3	0.3
		3	0.3	0.3	0.3
		4	0.3	0.3	0.3
		5	0.3	0.3	0.3
		6	0.3	0.3	0.3
		7	0.4	0.3	0.4
		8	0.3	0.3	0.4
		9	0.3	0.3	0.3
		10	0.4	0.3	0.4
		11	0.3	0.3	0.4
		12	0.3	0.3	0.4
		13	0.3	0.3	0.4
		14	0.3	0.3	0.3

Method	Resolution [Å $^{-1}$]	nr set	a [Å]	b [Å]	c [Å]	β [°]	Volume [Å ^{3}]
NEUT	N/A	1	6.100	3.4916	11.845	103.91	244.87
		2	6.100	3.4921	11.845	103.91	244.94
		3	6.098	3.4929	11.845	103.93	244.85
		4	6.099	3.4910	11.850	103.94	244.89
		5	6.098	3.4914	11.846	103.92	244.82
		6	6.100	3.4916	11.847	103.92	244.90
		7	6.099	3.4924	11.845	103.94	244.88
		8	6.101	3.4913	11.846	103.92	244.90
		9	6.102	3.4916	11.847	103.94	244.96
		10	6.100	3.4919	11.843	103.93	244.85
		11	6.100	3.4915	11.845	103.93	244.84
		12	6.100	3.4923	11.845	103.92	244.91
		13	6.098	3.4917	11.846	103.94	244.79
		14	6.099	3.4907	11.847	103.92	244.83
XRD	0.625	1	6.0908	3.4917	11.8397	103.845001	244.481995
		2	6.0868	3.4961	11.8283	103.879997	244.356995
		3	6.0899	3.4919	11.8298	103.908997	244.188004
		4	6.0836	3.4849	11.8194	103.942001	243.197998
		5	6.0941	3.4959	11.8428	103.949997	244.862
		6	6.0881	3.4889	11.8351	103.956001	243.966003
		7	6.0929	3.4897	11.8383	103.828003	244.414993
		8	6.0903	3.4944	11.8336	103.913002	244.453003
		9	6.0954	3.4953	11.8467	103.935997	244.968002
		10	6.0927	3.4927	11.8399	103.934998	244.537994
		11	6.0934	3.4916	11.8468	103.893997	244.675003
		12	6.0958	3.4955	11.8559	103.894997	245.231995
		13	6.1029	3.4992	11.8556	103.916	245.748993
	0.71429	1	6.0913	3.4919	11.8405	103.838997	244.539001
		2	6.0882	3.4964	11.8304	103.880997	244.477005
		3	6.09	3.4924	11.8337	103.910004	244.307007
		4	6.0861	3.4863	11.8252	103.940002	243.516998
		5	6.0938	3.496	11.8433	103.946999	244.869995
		6	6.0885	3.4897	11.8366	103.946999	244.078003
		7	6.0941	3.4894	11.8415	103.828003	244.509003

	8	6.0941	3.4931	11.841	103.932999	244.647003
	9	6.0947	3.495	11.848	103.936996	244.944
	10	6.0934	3.4929	11.8402	103.935997	244.585007
	11	6.0929	3.4915	11.8467	103.897003	244.641998
	12	6.0957	3.4947	11.8543	103.919998	245.112
	13	6.1002	3.4982	11.852	103.916	245.494995
0.83333	1	6.0924	3.4919	11.8409	103.835999	244.595001
	2	6.0891	3.4965	11.8309	103.884003	244.526993
	3	6.0904	3.4925	11.8356	103.906998	244.371994
	4	6.0878	3.4868	11.828	103.936996	243.681
	5	6.0941	3.4962	11.8432	103.948997	244.891998
	6	6.0894	3.4905	11.8381	103.939003	244.210007
	7	6.0952	3.4886	11.8449	103.825996	244.569
	8	6.0904	3.4954	11.8337	103.916	244.526001
	9	6.0947	3.4949	11.8491	103.938004	244.959
	10	6.0941	3.4931	11.841	103.932999	244.647003
	11	6.0922	3.4915	11.8466	103.898003	244.610992
	12	6.0951	3.4939	11.853	103.931999	244.992004
	13	6.0997	3.498	11.8525	103.917	245.470001
1	1	6.0926	3.4921	11.8406	103.832001	244.615005
	2	6.0894	3.4966	11.831	103.885002	244.546997
	3	6.0909	3.4927	11.8369	103.903999	244.436005
	4	6.0887	3.4869	11.8295	103.931	243.761002
	5	6.0936	3.4963	11.8436	103.949997	244.886002
	6	6.0904	3.4911	11.8397	103.938004	244.326004
	7	6.0953	3.4882	11.8459	103.828003	244.563004
	8	6.0902	3.4959	11.835	103.917	244.578995
	9	6.0951	3.4947	11.8498	103.939003	244.973999
	10	6.0947	3.4934	11.8417	103.933998	244.705002
	11	6.0918	3.4917	11.8476	103.897003	244.630997
	12	6.0942	3.493	11.8532	103.941002	244.886993
	13	6.0991	3.4979	11.8545	103.921997	245.475006
1.14	1	6.0931	3.4921	11.8409	103.842003	244.630997
	2	6.0893	3.4965	11.8311	103.884003	244.539001
	3	6.091	3.4928	11.8372	103.901001	244.457001
	4	6.0889	3.487	11.8299	103.93	243.785995
	5	6.0939	3.4965	11.8441	103.950996	244.921997
	6	6.0909	3.4916	11.8401	103.934998	244.393005
	7	6.095	3.4881	11.8462	103.841003	244.537003

8	6.0903	3.4959	11.8348	103.917	244.578995
9	6.0954	3.4948	11.8499	103.938004	244.996994
10	6.0924	3.4919	11.8409	103.835999	244.595001
11	6.092	3.4919	11.8481	103.898003	244.662003
12	6.0937	3.4929	11.8533	103.947998	244.854996
13	6.0988	3.4974	11.8505	103.920998	245.345993

Support Material - Oxalic Acid

Method	Resolution [Å $^{-1}$]	nr set	RF	RI	wR2F	wR2I	GoF
IAM	0.625	1	2.383	4.079	4.891	9.811	4.131
		2	2.353	3.339	3.067	6.127	2.105
		3	2.413	4.31	4.197	8.37	3.281
		4	2.211	3.4	3.544	7.117	2.69
		5	2.922	6.425	4.371	8.784	3.1
		6	2.721	6.474	4.467	8.938	3.575
		7	3.316	5.507	4.33	9.647	3.189
		8	2.417	4.4	3.419	6.963	2.45
		9	2.607	4.892	3.897	7.889	2.194
		10	2.304	3.715	3.465	7.074	2.319
		11	2.428	4.405	4.788	9.667	4.234
		12	2.425	3.613	3.809	7.641	2.706
		13	2.33	4.538	3.535	7.204	2.795
	0.71429	1	2.412	3.936	4.574	9.226	3.661
		2	2.578	3.476	3.222	6.253	2.066
		3	2.48	4.269	4.211	8.309	3.21
		4	2.413	3.65	3.541	7.072	2.556
		5	2.846	6.133	4.156	8.292	2.876
		6	2.653	5.786	4.245	8.488	3.335
		7	3.177	5.275	4.101	9.127	2.968
		8	2.486	4.085	3.355	6.845	2.199
		9	2.777	4.94	3.941	7.958	2.082
		10	2.511	3.981	3.458	7.048	2.108
		11	2.504	4.226	5.192	10.46	4.425
		12	2.442	3.614	3.869	7.636	2.785
		13	2.336	4.294	3.422	6.965	2.58
	0.83333	1	2.593	4.588	4.589	9.235	3.332
		2	3.01	4.052	3.269	6.355	1.855
		3	2.533	4.401	4.136	8.015	2.994
		4	2.589	4.011	3.539	7.06	2.32
		5	2.741	5.817	4.095	8.021	2.718
		6	2.671	5.75	4.192	8.249	3.146

		7	2.895	4.945	3.958	8.56	2.707
		8	2.604	4.036	3.401	6.92	2.014
		9	2.877	5.04	3.9	7.886	1.927
		10	2.851	4.249	3.656	7.431	1.951
		11	2.495	4.177	5.063	10.129	4.12
		12	2.477	3.902	3.852	7.453	2.726
		13	2.391	4.158	3.306	6.743	2.328
	1	1	2.574	4.25	4.253	8.476	2.726
		2	3.882	4.427	3.417	6.607	1.599
		3	2.494	4.282	3.784	7.289	2.494
		4	2.84	4.341	3.442	6.819	1.964
		5	2.633	5.61	3.742	7.331	2.286
		6	2.573	5.598	3.83	7.469	2.651
		7	2.877	4.842	3.628	7.589	2.207
		8	2.821	4.077	3.308	6.692	1.683
		9	2.895	4.99	3.62	7.266	1.625
		10	3.335	4.592	3.774	7.65	1.721
		11	2.31	3.976	4.499	8.97	3.439
		12	2.409	4.067	3.578	6.911	2.39
		13	2.513	4.145	3.186	6.515	2.008
	1.14	1	3.177	5.09	4.319	8.507	2.431
		2	6.15	5.527	3.912	7.489	1.468
		3	2.948	4.454	3.955	7.476	2.258
		4	3.591	5.474	3.708	7.297	1.794
		5	3.078	5.838	4.065	7.842	2.284
		6	2.968	5.861	3.856	7.489	2.325
		7	2.836	4.631	3.728	7.427	2.179
		8	3.863	4.551	3.641	7.24	1.541
		9	3.473	5.248	3.721	7.422	1.576
		10	3.491	4.924	3.918	7.949	1.826
		11	2.349	3.961	4.425	8.824	3.304
		12	2.61	4.408	3.415	6.604	2.051
		13	2.9	4.253	3.27	6.66	1.823
UBDB	0.625	1	1.33	2.787	2.588	5.06	2.193
		2	1.303	1.721	1.764	3.439	1.214
		3	1.373	2.892	2.239	4.342	1.756

		4	1.22	2.057	1.922	3.744	1.464
		5	2.01	5.43	2.215	4.347	1.576
		6	1.76	5.361	2.397	4.639	1.925
		7	2.563	4.89	2.853	7.037	2.108
		8	1.444	3.153	1.904	3.824	1.369
		9	1.676	3.706	2.263	4.538	1.278
		10	1.386	2.458	1.914	3.854	1.286
		11	1.599	3.533	3.262	6.141	2.885
		12	1.457	2.579	2.018	3.943	1.439
		13	1.465	3.616	1.983	3.963	1.573
	0.71429	1	1.392	2.646	2.775	5.384	2.221
		2	1.614	1.819	2.152	3.981	1.38
		3	1.469	2.856	2.847	5.087	2.17
		4	1.384	2.08	2.206	4.149	1.592
		5	1.962	5.141	2.438	4.647	1.687
		6	1.681	4.759	2.731	5.018	2.145
		7	2.354	4.414	2.852	6.803	2.064
		8	1.614	3.125	2.057	4.109	1.348
		9	1.866	3.793	2.531	5.012	1.337
		10	1.568	2.603	2.075	4.154	1.265
		11	1.569	3.455	3.352	6.061	2.857
		12	1.514	2.567	2.569	4.568	1.849
		13	1.538	3.716	2.071	4.119	1.562
	0.83333	1	1.54	2.994	2.889	5.671	2.097
		2	2.061	1.991	2.32	4.285	1.316
		3	1.476	2.679	2.701	4.944	1.955
		4	1.534	2.11	2.196	4.185	1.44
		5	1.869	4.715	2.528	4.766	1.678
		6	1.64	4.42	2.66	4.952	1.996
		7	2.103	4.033	2.684	6.174	1.836
		8	1.789	3.131	2.188	4.376	1.296
		9	2.04	3.917	2.631	5.225	1.3
		10	1.874	2.714	2.237	4.47	1.193
		11	1.526	3.258	3.15	5.979	2.564
		12	1.524	2.472	2.532	4.618	1.792
		13	1.589	3.567	2.034	4.083	1.432

	1	1	1.684	2.681	2.767	5.398	1.774
		2	3.136	2.389	2.646	4.901	1.238
		3	1.642	2.542	2.633	4.884	1.735
		4	1.953	2.235	2.36	4.482	1.347
		5	1.912	4.424	2.537	4.806	1.55
		6	1.719	4.19	2.594	4.871	1.795
		7	2.248	3.989	2.62	5.635	1.594
		8	2.18	3.268	2.351	4.676	1.197
		9	2.215	3.925	2.668	5.282	1.198
		10	2.474	2.925	2.533	5.05	1.155
		11	1.541	3.101	2.932	5.722	2.241
		12	1.6	2.425	2.504	4.648	1.673
		13	1.85	3.498	2.144	4.345	1.351
	1.14	1	2.32	2.742	3.022	5.78	1.701
		2	5.445	3.317	3.171	5.863	1.19
		3	2.206	2.591	3.07	5.431	1.753
		4	2.718	2.811	2.703	5.126	1.308
		5	2.453	4.505	3.154	5.803	1.772
		6	2.15	3.801	2.694	5.089	1.625
		7	2.202	3.484	2.783	5.402	1.626
		8	3.249	3.26	2.813	5.472	1.191
		9	2.832	3.726	2.959	5.833	1.253
		10	2.666	3.331	2.742	5.475	1.279
		11	1.597	3.044	2.926	5.679	2.185
		12	1.924	2.543	2.566	4.808	1.542
		13	2.309	3.603	2.346	4.779	1.308
ELMAM2	0.625	1	1.263	2.603	2.436	4.723	2.064
		2	1.261	1.751	1.641	3.195	1.13
		3	1.288	2.755	2.092	4.058	1.641
		4	1.204	2.049	1.861	3.629	1.417
		5	1.952	5.397	2.103	4.14	1.496
		6	1.728	5.314	2.297	4.478	1.845
		7	2.44	4.646	2.676	6.766	1.977
		8	1.379	2.94	1.803	3.606	1.296
		9	1.599	3.498	2.138	4.286	1.208
		10	1.333	2.479	1.882	3.791	1.264

	11	1.553	3.318	3.242	5.93	2.867
	12	1.368	2.349	1.942	3.818	1.384
	13	1.398	3.39	1.895	3.827	1.503
0.71429	1	1.31	2.417	2.647	5.084	2.119
	2	1.572	1.858	2.113	3.795	1.355
	3	1.378	2.657	2.726	4.875	2.078
	4	1.374	2.082	2.148	4.058	1.55
	5	1.889	5.055	2.39	4.472	1.653
	6	1.625	4.625	2.632	4.899	2.068
	7	2.25	4.193	2.681	6.524	1.94
	8	1.538	2.85	1.936	3.858	1.269
	9	1.801	3.586	2.403	4.765	1.27
	10	1.533	2.636	2.032	4.079	1.239
	11	1.495	3.181	3.237	5.818	2.759
	12	1.416	2.246	2.473	4.444	1.78
	13	1.446	3.353	1.99	3.973	1.5
0.83333	1	1.488	2.742	2.798	5.442	2.031
	2	2.055	2.122	2.309	4.21	1.31
	3	1.472	2.556	2.751	4.902	1.991
	4	1.552	2.147	2.196	4.184	1.44
	5	1.836	4.631	2.499	4.715	1.658
	6	1.657	4.347	2.675	4.999	2.007
	7	2.021	3.794	2.549	5.982	1.744
	8	1.723	2.802	2.106	4.202	1.247
	9	2.011	3.723	2.592	5.162	1.28
	10	1.881	2.792	2.249	4.509	1.2
	11	1.501	2.979	3.111	5.822	2.532
	12	1.498	2.271	2.509	4.583	1.776
	13	1.52	3.134	2.011	4.064	1.416
1	1	1.368	2.299	2.203	4.152	1.412
	2	2.928	2.104	2.424	4.382	1.134
	3	1.36	2.163	2.268	3.961	1.495
	4	1.698	1.902	2.002	3.7	1.143
	5	1.713	4.314	2.145	3.933	1.31
	6	1.415	3.789	2.122	3.77	1.469
	7	1.92	3.578	2.059	4.6	1.252

		8	1.96	2.984	1.954	3.821	0.994
		9	2.021	3.602	2.361	4.642	1.06
		10	2.243	2.671	2.13	4.219	0.971
		11	1.179	2.508	2.241	4.161	1.713
		12	1.344	2.085	2.175	3.929	1.453
		13	1.612	3.112	1.844	3.789	1.162
	1.14	1	2.421	3.012	3.074	5.873	1.731
		2	5.508	3.638	3.201	5.918	1.201
		3	2.285	2.786	3.109	5.504	1.775
		4	2.815	3.251	2.779	5.279	1.344
		5	2.529	4.638	3.247	5.988	1.824
		6	2.229	3.988	2.755	5.238	1.662
		7	2.205	3.33	2.718	5.254	1.588
		8	3.246	3.04	2.793	5.427	1.182
		9	2.892	3.666	3.024	5.966	1.281
		10	2.734	3.535	2.778	5.557	1.295
		11	1.607	2.839	2.967	5.694	2.215
		12	1.991	2.772	2.615	4.917	1.571
		13	2.273	3.197	2.355	4.809	1.313
GID	0.625	1	1.263	2.603	2.436	4.723	2.064
		2	1.261	1.751	1.641	3.195	1.13
		3	1.288	2.755	2.092	4.058	1.641
		4	1.204	2.049	1.861	3.629	1.417
		5	1.952	5.397	2.103	4.14	1.496
		6	1.728	5.314	2.297	4.478	1.845
		7	2.44	4.646	2.676	6.766	1.977
		8	1.379	2.94	1.803	3.606	1.296
		9	1.599	3.498	2.138	4.286	1.208
		10	1.333	2.479	1.882	3.791	1.264
		11	1.553	3.318	3.242	5.93	2.867
		12	1.368	2.349	1.942	3.818	1.384
		13	1.398	3.39	1.895	3.827	1.503
	0.71429	1	1.31	2.417	2.647	5.084	2.119
		2	1.572	1.858	2.113	3.795	1.355
		3	1.378	2.657	2.726	4.875	2.078
		4	1.374	2.082	2.148	4.058	1.55

		5	1.889	5.055	2.39	4.472	1.653
		6	1.625	4.625	2.632	4.899	2.068
		7	2.25	4.193	2.681	6.524	1.94
		8	1.538	2.85	1.936	3.858	1.269
		9	1.801	3.586	2.403	4.765	1.27
		10	1.533	2.636	2.032	4.079	1.239
		11	1.495	3.181	3.237	5.818	2.759
		12	1.416	2.246	2.473	4.444	1.78
		13	1.446	3.353	1.99	3.973	1.5
	0.83333	1	1.488	2.742	2.798	5.442	2.031
		2	2.055	2.122	2.309	4.21	1.31
		3	1.472	2.556	2.751	4.902	1.991
		4	1.552	2.147	2.196	4.184	1.44
		5	1.836	4.631	2.499	4.715	1.658
		6	1.657	4.347	2.675	4.999	2.007
		7	2.021	3.794	2.549	5.982	1.744
		8	1.723	2.802	2.106	4.202	1.247
		9	2.011	3.723	2.592	5.162	1.28
		10	1.881	2.792	2.249	4.509	1.2
		11	1.501	2.979	3.111	5.822	2.532
		12	1.498	2.271	2.509	4.583	1.776
		13	1.52	3.134	2.011	4.064	1.416
	1	1	1.672	2.46	2.738	5.301	1.755
		2	3.133	2.53	2.631	4.848	1.231
		3	1.66	2.546	2.624	4.856	1.73
		4	1.998	2.344	2.389	4.536	1.363
		5	1.915	4.396	2.542	4.83	1.553
		6	1.754	4.179	2.631	4.967	1.821
		7	2.19	3.771	2.52	5.487	1.533
		8	2.13	2.91	2.293	4.544	1.167
		9	2.202	3.685	2.646	5.241	1.188
		10	2.512	3.027	2.572	5.128	1.173
		11	1.53	2.821	2.937	5.655	2.245
		12	1.622	2.349	2.522	4.708	1.685
		13	1.807	3.079	2.149	4.371	1.354
	1.14	1	2.421	3.012	3.074	5.873	1.731

		2	5.508	3.638	3.201	5.918	1.201
		3	2.285	2.786	3.109	5.504	1.775
		4	2.815	3.251	2.779	5.279	1.344
		5	2.529	4.638	3.247	5.988	1.824
		6	2.229	3.988	2.755	5.238	1.662
		7	2.205	3.33	2.718	5.254	1.588
		8	3.246	3.04	2.793	5.427	1.182
		9	2.892	3.666	3.024	5.966	1.281
		10	2.734	3.535	2.778	5.557	1.295
		11	1.607	2.839	2.967	5.694	2.215
		12	1.991	2.772	2.615	4.917	1.571
		13	2.273	3.197	2.355	4.809	1.313
HAR(B3LYP)	0.625	1	0.8554	2.1277	1.7305	2.9071	1.482268
		2	0.9984	1.2234	1.2949	1.8905	0.900208
		3	0.8752	2.1384	1.4704	2.2034	1.165199
		4	0.808	1.3923	1.2772	1.95	0.982728
		5	1.5861	4.8813	1.468	2.2146	1.054893
		6	1.3015	4.5883	1.566	2.4734	1.270661
		7	2.0708	4.1941	1.5781	2.4855	1.178094
		8	0.9901	2.4631	1.1237	1.844	0.815872
		9	1.4031	3.2323	1.6418	2.9068	0.93464
		10	0.9293	1.7881	1.1659	1.9036	0.790374
		11	1.2341	2.939	2.548	4.6927	2.2781
		12	1.1016	2.0108	1.413	2.4428	1.017081
		13	1.0626	2.943	1.3114	2.2664	1.051262
	0.71429	1	0.8685	1.9535	1.8969	3.661	1.525824
		2	1.2986	1.3211	1.7108	2.0813	1.101296
		3	0.9878	2.0759	2.1149	2.7055	1.619345
		4	0.9991	1.5205	1.5778	2.1278	1.143807
		5	1.5521	4.6301	1.7198	2.3321	1.194681
		6	1.2062	3.9403	1.9401	2.5608	1.531204
		7	1.8606	3.7193	1.6894	2.4006	1.228233
		8	1.1487	2.4259	1.2496	1.9382	0.822087
		9	1.5365	3.272	1.8304	3.1825	0.968986
		10	1.1644	1.9782	1.3357	2.0648	0.816857
		11	1.2408	2.8631	3.0215	4.5149	2.588115

	12	1.1659	2.005	1.9507	2.776	1.409956
	13	1.0949	2.9354	1.3866	2.1815	1.050358
0.83333	1	1.0182	2.2464	2.0266	5.2382	1.471373
	2	1.7262	1.4989	1.8676	2.2386	1.058503
	3	1.0309	1.9395	2.0669	2.6081	1.495974
	4	1.1696	1.5589	1.5984	2.1856	1.047664
	5	1.474	4.217	1.8429	2.4782	1.222559
	6	1.1975	3.6825	1.906	2.4392	1.430484
	7	1.5939	3.2615	1.6685	2.2869	1.141073
	8	1.3414	2.4235	1.3783	2.0388	0.815679
	9	1.704	3.3634	2.0079	3.4689	0.989268
	10	1.504	2.1388	1.5432	2.2664	0.822378
	11	1.0052	2.3956	2.2359	3.4652	1.820834
	12	1.1661	1.9317	1.9602	2.7031	1.387425
	13	1.158	2.752	1.3971	2.1209	0.983839
1	1	1.1968	1.9287	1.8923	4.0851	1.208754
	2	2.8141	1.8651	2.1316	2.4232	0.992539
	3	1.2341	1.8153	2.0144	2.5503	1.323564
	4	1.6054	1.7	1.7664	2.3308	1.004487
	5	1.5777	4.022	1.8961	2.6446	1.153804
	6	1.3156	3.4925	1.8902	2.4846	1.304434
	7	1.7642	3.1768	1.6413	2.2493	0.994712
	8	1.7729	2.5508	1.5896	2.2209	0.805397
	9	1.9091	3.3457	2.1155	3.5071	0.943795
	10	2.134	2.3595	1.8507	2.4747	0.840387
	11	1.0465	2.2302	2.0107	3.1873	1.53316
	12	1.2758	1.984	1.9815	2.7165	1.31934
	13	1.4489	2.6753	1.5638	2.2283	0.982184
1.14	1	1.9834	2.4971	2.2788	3.4919	1.276459
	2	5.2547	3.2182	2.5669	2.8561	0.956516
	3	1.8828	2.0254	2.5064	3.059	1.424138
	4	2.4451	2.5921	2.1195	2.9605	1.019821
	5	2.2199	4.2686	2.6493	3.7725	1.480637
	6	1.7875	3.1778	2.029	2.6886	1.217921
	7	1.7614	2.6843	1.9932	3.0951	1.159501
	8	2.943	2.7771	2.1031	2.7962	0.884493

		9	2.5979	3.3345	2.4959	4.0093	1.049264
		10	2.3493	2.8705	2.1109	3.1376	0.979792
		11	1.138	2.2604	2.0627	3.2789	1.535802
		12	1.6532	2.271	2.0884	2.8987	1.248436
		13	1.9203	2.7719	1.7682	2.3228	0.980994
HAR(BLYP)	0.625	1	0.8956	2.1306	1.7794	2.9787	1.52419
		2	1.0523	1.3516	1.3483	1.9859	0.937302
		3	0.9178	2.1353	1.5319	2.3277	1.213926
		4	0.8531	1.4209	1.3284	2.0632	1.022152
		5	1.6402	4.9382	1.5376	2.337	1.104872
		6	1.3328	4.5383	1.6352	2.6167	1.326879
		7	2.0887	4.1468	1.6146	2.5476	1.205322
		8	1.0246	2.4886	1.1629	1.9487	0.844355
		9	1.4031	3.2323	1.6418	2.9068	0.93464
		10	0.9629	1.8308	1.2002	1.9983	0.813628
		11	1.2545	2.9343	2.5512	4.6553	2.280888
		12	1.132	2.0563	1.449	2.4679	1.042941
		13	1.0777	2.8999	1.3224	2.2888	1.060057
	0.71429	1	0.8975	1.9575	1.9223	3.657	1.546275
		2	1.3538	1.5026	1.7469	2.1713	1.124572
		3	1.0302	2.0736	2.1572	2.8089	1.651763
		4	1.0434	1.5512	1.6175	2.2478	1.17257
		5	1.5763	4.6319	1.7557	2.4061	1.219607
		6	1.2282	3.9059	1.9684	2.6663	1.553569
		7	1.875	3.6681	1.709	2.448	1.242411
		8	1.1819	2.4283	1.2902	2.0516	0.84876
		9	1.5692	3.2585	1.8632	3.2712	0.986332
		10	1.2022	2.0581	1.3705	2.1667	0.838138
		11	1.2523	2.8226	2.9931	4.4527	2.563806
		12	1.198	2.0557	1.9668	2.7641	1.421579
		13	1.1006	2.8511	1.3967	2.2071	1.057973
	0.83333	1	1.0425	2.2542	2.0355	5.1107	1.477835
		2	1.7844	1.7309	1.9003	2.3398	1.077054
		3	1.0764	1.9824	2.1006	2.706	1.520348
		4	1.2194	1.6359	1.6394	2.316	1.074519
		5	1.4892	4.2096	1.8638	2.5289	1.236467

		6	1.2366	3.7119	1.9382	2.5378	1.454671
		7	1.5943	3.1854	1.6805	2.3223	1.14932
		8	1.3676	2.3964	1.4087	2.133	0.833641
		9	1.7304	3.3389	2.0336	3.5362	1.001917
		10	1.5445	2.2396	1.579	2.3759	0.841463
		11	1.0307	2.3718	2.2532	3.444	1.834961
		12	1.1954	1.9887	1.972	2.7026	1.395755
		13	1.1653	2.6486	1.408	2.151	0.991508
	1	1	1.216	1.9485	1.9016	4.0008	1.214743
		2	2.8598	2.1055	2.1557	2.5185	1.003738
		3	1.2703	1.9089	2.0364	2.6269	1.33803
		4	1.6544	1.8417	1.7978	2.4502	1.022331
		5	1.5864	4.0284	1.9022	2.6669	1.157466
		6	1.3502	3.5617	1.9088	2.5508	1.317217
		7	1.7641	3.1137	1.645	2.2692	0.996998
		8	1.7866	2.4951	1.608	2.2917	0.814721
		9	1.9267	3.3056	2.1324	3.558	0.951326
		10	2.1683	2.4685	1.8754	2.5736	0.851598
		11	1.058	2.1965	2.0152	3.1497	1.536625
		12	1.2979	2.0586	1.9851	2.7145	1.321794
		13	1.4594	2.5866	1.5711	2.2562	0.986803
	1.14	1	2.0199	2.6814	2.3016	3.5321	1.289211
		2	5.3019	3.4431	2.6004	3.008	0.969026
		3	1.9218	2.1627	2.5287	3.1301	1.436799
		4	2.5039	2.8832	2.1607	3.1131	1.039624
		5	2.2332	4.3228	2.655	3.79	1.483818
		6	1.8299	3.3655	2.0487	2.7735	1.229747
		7	1.7806	2.7035	1.9999	3.1252	1.163385
		8	2.964	2.7849	2.1176	2.8647	0.890572
		9	2.6277	3.3791	2.5173	4.072	1.058259
		10	2.3873	2.9854	2.1353	3.2097	0.99113
		11	1.1529	2.2503	2.069	3.2571	1.540487
		12	1.6773	2.411	2.0916	2.9158	1.250329
		13	1.9281	2.6866	1.7729	2.3442	0.983575
MR_no	1.19	1	1.413	2.228	2.09	3.919	1.318
		2	1.987	2.057	2.16	4.145	1.059

		3	1.388	2.324	2.274	4.034	1.449
		4	1.5	2.084	1.888	3.578	1.061
		5	1.638	3.393	2.369	4.35	1.461
		6	1.283	3.11	1.835	3.409	1.222
		7	1.372	2.737	1.83	3.526	1.193
		8	1.663	2.742	1.836	3.684	0.943
		9	1.921	3.533	2.249	4.503	1.089
		10	1.605	2.485	1.843	3.723	1.002
		11	1.031	2.513	1.843	3.404	1.462
		12	1.222	2.035	1.912	3.506	1.25
		13	1.369	2.979	1.585	3.191	0.985
MR	1.18	1	1.415	2.232	2.094	3.931	1.319
		2	1.99	2.037	2.165	4.154	1.061
		3	1.386	2.294	2.278	4.039	1.45
		4	1.504	2.079	1.895	3.598	1.065
		5	1.651	3.426	2.379	4.37	1.467
		6	1.283	3.089	1.835	3.414	1.222
		7	1.363	2.704	1.852	3.572	1.206
		8	1.656	2.713	1.835	3.681	0.941
		9	1.924	3.56	2.252	4.512	1.09
		10	1.602	2.453	1.841	3.718	1.000
		11	1.034	2.556	1.848	3.417	1.465
		12	1.226	2.05	1.916	3.514	1.252
		13	1.378	3.032	1.592	3.207	0.988

Method	Resolution [Å $^{-1}$]	nr set	C1 - U equiv	O1 - U equiv	O2 - U equiv	O3 - U equiv	H1 - U iso	H2 - U iso	H3 - U iso
IAM	0.625	1	0.0111	0.0143	0.014	0.0139	0.0215	0.0191	0.0193
		2	0.0113	0.0149	0.0146	0.0146	0.022	0.0213	0.0214
		3	0.0111	0.0144	0.0141	0.014	0.0215	0.0198	0.0201
		4	0.0109	0.0138	0.0136	0.0136	0.0205	0.0195	0.0197
		5	0.0112	0.0146	0.0144	0.0143	0.0217	0.0203	0.0204
		6	0.0117	0.0149	0.0147	0.0146	0.0221	0.0204	0.0207
		7	0.0113	0.0145	0.0142	0.0142	0.0218	0.0203	0.0204
		8	0.0109	0.0143	0.0139	0.0141	0.0211	0.0203	0.0205
		9	0.0108	0.0144	0.014	0.014	0.0212	0.0202	0.0203
		10	0.011	0.0144	0.014	0.0142	0.0213	0.0205	0.0206
		11	0.011	0.0142	0.0139	0.0137	0.0217	0.018	0.0186
		12	0.0113	0.0147	0.0143	0.0142	0.0217	0.0205	0.0206
		13	0.0114	0.0148	0.0147	0.0145	0.0219	0.0209	0.021
	0.71429	1	0.0098	0.0133	0.0131	0.0129	0.027	0.003	0.0016
		2	0.0104	0.0141	0.0138	0.0137	0.02	0.003	0.006
		3	0.0098	0.0133	0.0132	0.013	0.024	0.005	0.007
		4	0.0095	0.0127	0.0126	0.0126	0.024	0.005	0.005
		5	0.0099	0.0137	0.0136	0.0133	0.026	0.006	0.005
		6	0.0102	0.0137	0.0136	0.0134	0.022	0.005	0.005
		7	0.0099	0.0136	0.0134	0.0132	0.033	0.005	0.003
		8	0.0099	0.0135	0.0133	0.0133	0.023	0.003	0.007
		9	0.0098	0.0135	0.0133	0.0132	0.026	0.005	0.004
		10	0.0099	0.0135	0.0132	0.0132	0.022	0.0022	0.005
		11	0.0096	0.0131	0.0129	0.0127	0.032	0.003	0.004
		12	0.0101	0.0137	0.0136	0.0133	0.019	0.011	0.009
		13	0.0106	0.0143	0.0141	0.014	0.019	0.009	0.009
	0.83333	1	0.0091	0.0126	0.0124	0.0122	0.027	0.005	0.005
		2	0.0097	0.0135	0.0132	0.0131	0.024	0.006	0.008
		3	0.0091	0.0127	0.0125	0.0123	0.026	0.008	0.011
		4	0.0088	0.0121	0.0119	0.0119	0.025	0.006	0.007
		5	0.0093	0.0131	0.0129	0.0128	0.027	0.008	0.009

		6	0.0094	0.013	0.0128	0.0127	0.024	0.007	0.007
		7	0.0092	0.013	0.0127	0.0126	0.034	0.006	0.007
		8	0.0092	0.0129	0.0127	0.0127	0.023	0.006	0.009
		9	0.0093	0.013	0.0127	0.0127	0.026	0.01	0.008
		10	0.0094	0.0129	0.0126	0.0126	0.022	0.003	0.007
		11	0.0089	0.0125	0.0123	0.0121	0.03	0.006	0.008
		12	0.0094	0.0131	0.0128	0.0127	0.02	0.012	0.013
		13	0.01	0.0138	0.0135	0.0134	0.02	0.011	0.012
	1	1	0.0088	0.0124	0.0121	0.012	0.027	0.006	0.006
		2	0.0094	0.0133	0.013	0.0129	0.025	0.007	0.008
		3	0.00881	0.01244	0.01221	0.01211	0.024	0.007	0.011
		4	0.0084	0.0118	0.0115	0.0115	0.026	0.007	0.007
		5	0.00913	0.01296	0.01268	0.01255	0.026	0.009	0.009
		6	0.00902	0.01266	0.01241	0.01234	0.023	0.007	0.008
		7	0.0091	0.0128	0.0126	0.0125	0.034	0.007	0.008
		8	0.00912	0.0129	0.01259	0.0125	0.023	0.007	0.01
		9	0.00892	0.0127	0.0124	0.0123	0.026	0.012	0.01
		10	0.009	0.0126	0.0124	0.0123	0.022	0.003	0.007
		11	0.00872	0.01232	0.01206	0.01197	0.029	0.006	0.009
		12	0.00906	0.0127	0.01246	0.01236	0.019	0.011	0.012
		13	0.00976	0.01354	0.01326	0.01319	0.019	0.011	0.013
	1.14	1	0.00948	0.01313	0.01285	0.01277	0.029	0.007	0.006
		2	0.0101	0.014	0.0137	0.0136	0.024	0.008	0.008
		3	0.00944	0.0131	0.01285	0.01277	0.028	0.01	0.013
		4	0.0093	0.0126	0.0124	0.0124	0.027	0.008	0.008
		5	0.00983	0.01365	0.01335	0.01323	0.029	0.009	0.01
		6	0.00954	0.01321	0.01296	0.01286	0.025	0.008	0.009
		7	0.00984	0.0136	0.01331	0.01323	0.039	0.009	0.01
		8	0.00969	0.01344	0.01318	0.01313	0.024	0.01	0.01
		9	0.00938	0.01313	0.01285	0.01273	0.028	0.012	0.011
		10	0.0101	0.0137	0.0135	0.0134	0.024	0.006	0.008
		11	0.0095	0.01311	0.01285	0.01275	0.029	0.007	0.009
		12	0.00951	0.01313	0.01289	0.01279	0.019	0.011	0.013
		13	0.00973	0.01351	0.01322	0.01316	0.019	0.011	0.013
UBDB	0.625	1	0.0094	0.0125	0.0121	0.0121	0.031	0.021	0.022

	2	0.0099	0.0133	0.0128	0.0129	0.031	0.02	0.023
	3	0.0096	0.0128	0.0124	0.0124	0.033	0.021	0.024
	4	0.0094	0.0122	0.0118	0.0119	0.032	0.02	0.025
	5	0.0096	0.013	0.0126	0.0126	0.032	0.022	0.025
	6	0.0101	0.0133	0.0129	0.0129	0.033	0.021	0.024
	7	0.0097	0.0129	0.0125	0.0126	0.039	0.022	0.021
	8	0.0093	0.0126	0.0122	0.0123	0.033	0.02	0.026
	9	0.0094	0.0127	0.0122	0.0122	0.031	0.02	0.025
	10	0.0094	0.0126	0.0121	0.0123	0.029	0.021	0.025
	11	0.0094	0.0124	0.0119	0.0119	0.043	0.014	0.026
	12	0.0099	0.0132	0.0129	0.0127	0.033	0.022	0.025
	13	0.0099	0.0133	0.013	0.0129	0.03	0.025	0.027
0.71429	1	0.0092	0.0126	0.0122	0.0121	0.031	0.022	0.021
	2	0.0096	0.0134	0.0129	0.0128	0.031	0.021	0.024
	3	0.0093	0.0127	0.0124	0.0123	0.032	0.022	0.026
	4	0.0089	0.012	0.0117	0.0117	0.032	0.021	0.024
	5	0.0096	0.0132	0.0128	0.0127	0.033	0.023	0.024
	6	0.0097	0.0131	0.0129	0.0128	0.032	0.023	0.023
	7	0.0095	0.013	0.0127	0.0125	0.04	0.023	0.021
	8	0.0092	0.0127	0.0123	0.0123	0.033	0.021	0.024
	9	0.0092	0.0128	0.0123	0.0123	0.032	0.021	0.023
	10	0.0092	0.0126	0.0122	0.0122	0.03	0.021	0.024
	11	0.0091	0.0124	0.0122	0.0121	0.032	0.023	0.024
	12	0.0096	0.0131	0.0129	0.0127	0.035	0.026	0.028
	13	0.0099	0.0135	0.0133	0.0131	0.032	0.026	0.027
0.83333	1	0.009	0.0124	0.0121	0.012	0.031	0.023	0.022
	2	0.0095	0.0131	0.0128	0.0127	0.031	0.021	0.023
	3	0.0091	0.0126	0.0123	0.0121	0.031	0.024	0.024
	4	0.0087	0.0118	0.0116	0.0115	0.031	0.022	0.024
	5	0.0094	0.0131	0.0128	0.0126	0.032	0.024	0.025
	6	0.0094	0.0129	0.0126	0.0124	0.031	0.024	0.022
	7	0.0093	0.0128	0.0126	0.0125	0.039	0.023	0.022
	8	0.0091	0.0126	0.0123	0.0123	0.033	0.022	0.023
	9	0.0091	0.0128	0.0124	0.0123	0.031	0.024	0.023
	10	0.0091	0.0125	0.0121	0.0121	0.03	0.021	0.024

		11	0.009	0.0124	0.0122	0.012	0.031	0.024	0.023
		12	0.00944	0.01297	0.01268	0.01248	0.035	0.028	0.028
		13	0.00987	0.01351	0.01322	0.0131	0.032	0.028	0.027
	1	1	0.00886	0.01235	0.01211	0.01197	0.031	0.023	0.022
		2	0.00944	0.01313	0.01285	0.01266	0.031	0.022	0.022
		3	0.0089	0.01244	0.01221	0.01202	0.032	0.023	0.025
		4	0.00851	0.01165	0.01148	0.01137	0.031	0.022	0.023
		5	0.00923	0.01294	0.01273	0.01251	0.034	0.024	0.026
		6	0.00911	0.01267	0.01244	0.01229	0.032	0.024	0.023
		7	0.00922	0.01283	0.01262	0.01246	0.039	0.024	0.023
		8	0.00909	0.01274	0.01246	0.01235	0.033	0.022	0.024
		9	0.0089	0.01257	0.01229	0.01214	0.031	0.025	0.023
		10	0.00891	0.01246	0.01212	0.01208	0.029	0.021	0.024
		11	0.00882	0.01232	0.01212	0.01195	0.032	0.025	0.024
		12	0.00911	0.01267	0.01246	0.01227	0.038	0.029	0.028
		13	0.00977	0.01345	0.0132	0.01307	0.032	0.028	0.027
	1.14	1	0.00957	0.01308	0.01286	0.01269	0.0205	0.0191	0.019
		2	0.01015	0.01381	0.01358	0.01339	0.03	0.023	0.021
		3	0.00954	0.0131	0.01286	0.0127	0.035	0.025	0.027
		4	0.00936	0.0125	0.01236	0.01225	0.033	0.023	0.025
		5	0.00993	0.01364	0.01342	0.01322	0.036	0.025	0.026
		6	0.00962	0.01318	0.01298	0.01281	0.032	0.025	0.024
		7	0.00997	0.01358	0.01336	0.0132	0.041	0.024	0.025
		8	0.00969	0.01338	0.01307	0.01301	0.034	0.024	0.024
		9	0.00942	0.01308	0.01282	0.01266	0.032	0.025	0.024
		10	0.01001	0.01357	0.01324	0.01317	0.03	0.023	0.026
		11	0.00962	0.01312	0.01293	0.01275	0.032	0.026	0.025
		12	0.00957	0.01312	0.01291	0.01275	0.039	0.029	0.029
		13	0.00976	0.01343	0.0132	0.01307	0.032	0.028	0.027
ELMAM	0.625	1	0.0094	0.0128	0.0126	0.0127	0.043	0.028	0.025
		2	0.0099	0.0136	0.0134	0.0134	0.048	0.027	0.028
		3	0.0096	0.013	0.0129	0.0129	0.046	0.027	0.028
		4	0.0094	0.0125	0.0124	0.0124	0.048	0.027	0.027
		5	0.0096	0.0132	0.0131	0.0131	0.044	0.028	0.028
		6	0.0101	0.0135	0.0134	0.0134	0.045	0.029	0.027

	7	0.0097	0.0132	0.013	0.0131	0.055	0.028	0.024
	8	0.0094	0.0129	0.0127	0.0128	0.05	0.027	0.029
	9	0.0094	0.013	0.0128	0.0128	0.049	0.027	0.028
	10	0.0094	0.0129	0.0127	0.0128	0.046	0.028	0.029
	11	0.0095	0.0128	0.0124	0.0124	0.055	0.021	0.031
	12	0.0099	0.0135	0.0133	0.0133	0.046	0.028	0.027
	13	0.0099	0.0136	0.0135	0.0135	0.047	0.031	0.029
0.71429	1	0.0091	0.0126	0.0125	0.0125	0.042	0.028	0.024
	2	0.0097	0.0133	0.0132	0.013	0.043	0.024	0.026
	3	0.0091	0.0127	0.0126	0.0126	0.044	0.026	0.027
	4	0.0088	0.012	0.012	0.012	0.046	0.026	0.026
	5	0.0094	0.0132	0.0131	0.013	0.044	0.028	0.027
	6	0.0096	0.0131	0.0131	0.0131	0.044	0.028	0.026
	7	0.0093	0.0129	0.0129	0.0128	0.054	0.027	0.023
	8	0.0091	0.0127	0.0126	0.0125	0.049	0.027	0.027
	9	0.0091	0.0128	0.0126	0.0126	0.047	0.027	0.026
	10	0.0091	0.0127	0.0125	0.0125	0.045	0.027	0.028
	11	0.009	0.0125	0.0124	0.0124	0.042	0.028	0.026
	12	0.0095	0.0131	0.013	0.013	0.046	0.027	0.03
	13	0.0098	0.0136	0.0135	0.0134	0.045	0.03	0.029
0.83333	1	0.0088	0.0124	0.0122	0.0122	0.041	0.028	0.023
	2	0.0093	0.013	0.0129	0.0128	0.042	0.024	0.024
	3	0.0088	0.0124	0.0123	0.0122	0.042	0.027	0.027
	4	0.0085	0.0117	0.0116	0.0116	0.043	0.025	0.026
	5	0.00919	0.01298	0.01281	0.01272	0.041	0.028	0.026
	6	0.0092	0.0128	0.0126	0.0126	0.041	0.029	0.024
	7	0.009	0.0127	0.0126	0.0126	0.051	0.026	0.023
	8	0.00885	0.0126	0.01237	0.01239	0.047	0.026	0.025
	9	0.009	0.0127	0.0124	0.0124	0.045	0.027	0.024
	10	0.0088	0.0125	0.0122	0.0123	0.044	0.027	0.026
	11	0.0088	0.0123	0.0121	0.0121	0.039	0.03	0.024
	12	0.00922	0.01287	0.01264	0.0126	0.046	0.029	0.028
	13	0.00967	0.01343	0.01323	0.01322	0.044	0.031	0.028
1	1	0.00881	0.01236	0.01211	0.01198	0.035	0.0275	0.0254
	2	0.00939	0.01308	0.01286	0.0126	0.033	0.024	0.027

		3	0.00881	0.01239	0.01219	0.01204	0.036	0.027	0.029
		4	0.00843	0.01166	0.01147	0.01139	0.038	0.025	0.029
		5	0.00917	0.01295	0.0127	0.01251	0.036	0.027	0.029
		6	0.00903	0.01264	0.01243	0.01231	0.037	0.027	0.028
		7	0.00918	0.01286	0.01262	0.01246	0.04	0.028	0.026
		8	0.00902	0.01272	0.01246	0.01233	0.037	0.027	0.029
		9	0.00884	0.01255	0.01226	0.01214	0.037	0.027	0.029
		10	0.00884	0.01243	0.01215	0.01208	0.034	0.027	0.031
		11	0.00876	0.01233	0.0121	0.01197	0.036	0.0273	0.0273
		12	0.00905	0.01265	0.01242	0.01227	0.035	0.028	0.029
		13	0.0097	0.01344	0.01316	0.01304	0.034	0.031	0.031
	1.14	1	0.00938	0.01293	0.01281	0.01269	0.042	0.029	0.022
		2	0.00997	0.01364	0.0136	0.01339	0.041	0.025	0.022
		3	0.00937	0.01293	0.0128	0.01264	0.041	0.026	0.025
		4	0.00916	0.01236	0.01234	0.01226	0.045	0.026	0.026
		5	0.00976	0.01348	0.01334	0.01317	0.041	0.027	0.025
		6	0.00943	0.01303	0.01289	0.01277	0.041	0.03	0.024
		7	0.00978	0.01343	0.01331	0.01319	0.051	0.028	0.025
		8	0.0095	0.01325	0.01303	0.01299	0.049	0.027	0.024
		9	0.00925	0.01295	0.01275	0.01263	0.045	0.028	0.025
		10	0.00975	0.01342	0.01322	0.01323	0.044	0.027	0.028
		11	0.0094	0.01296	0.01282	0.01272	0.04	0.031	0.024
		12	0.00939	0.01297	0.01281	0.01269	0.052	0.03	0.03
		13	0.00956	0.01328	0.01313	0.01304	0.044	0.031	0.027
GID	0.625	1	0.0094	0.0128	0.0126	0.0127	0.043	0.028	0.025
		2	0.0099	0.0136	0.0134	0.0134	0.048	0.027	0.028
		3	0.0096	0.013	0.0129	0.0129	0.046	0.027	0.028
		4	0.0094	0.0125	0.0124	0.0124	0.048	0.027	0.027
		5	0.0096	0.0132	0.0131	0.0131	0.044	0.028	0.028
		6	0.0101	0.0135	0.0134	0.0134	0.045	0.029	0.027
		7	0.0097	0.0132	0.013	0.0131	0.055	0.028	0.024
		8	0.0094	0.0129	0.0127	0.0128	0.05	0.027	0.029
		9	0.0094	0.013	0.0128	0.0128	0.049	0.027	0.028
		10	0.0094	0.0129	0.0127	0.0128	0.046	0.028	0.029
		11	0.0095	0.0128	0.0124	0.0124	0.055	0.021	0.031

	12	0.0099	0.0135	0.0133	0.0133	0.046	0.028	0.027
	13	0.0099	0.0136	0.0135	0.0135	0.047	0.031	0.029
0.71429	1	0.0091	0.0126	0.0125	0.0125	0.042	0.028	0.024
	2	0.0097	0.0133	0.0132	0.013	0.043	0.024	0.026
	3	0.0091	0.0127	0.0126	0.0126	0.044	0.026	0.027
	4	0.0088	0.012	0.012	0.012	0.046	0.026	0.026
	5	0.0094	0.0132	0.0131	0.013	0.044	0.028	0.027
	6	0.0096	0.0131	0.0131	0.0131	0.044	0.028	0.026
	7	0.0093	0.0129	0.0129	0.0128	0.054	0.027	0.023
	8	0.0091	0.0127	0.0126	0.0125	0.049	0.027	0.027
	9	0.0091	0.0128	0.0126	0.0126	0.047	0.027	0.026
	10	0.0091	0.0127	0.0125	0.0125	0.045	0.027	0.028
	11	0.009	0.0125	0.0124	0.0124	0.042	0.028	0.026
	12	0.0095	0.0131	0.013	0.013	0.046	0.027	0.03
	13	0.0098	0.0136	0.0135	0.0134	0.045	0.03	0.029
0.83333	1	0.0088	0.0124	0.0122	0.0122	0.041	0.028	0.023
	2	0.0093	0.013	0.0129	0.0128	0.042	0.024	0.024
	3	0.0088	0.0124	0.0123	0.0122	0.042	0.027	0.027
	4	0.0085	0.0117	0.0116	0.0116	0.043	0.025	0.026
	5	0.00919	0.01298	0.01281	0.01272	0.041	0.028	0.026
	6	0.0092	0.0128	0.0126	0.0126	0.041	0.029	0.024
	7	0.009	0.0127	0.0126	0.0126	0.051	0.026	0.023
	8	0.00885	0.0126	0.01237	0.01239	0.047	0.026	0.025
	9	0.009	0.0127	0.0124	0.0124	0.045	0.027	0.024
	10	0.0088	0.0125	0.0122	0.0123	0.044	0.027	0.026
	11	0.0088	0.0123	0.0121	0.0121	0.039	0.03	0.024
	12	0.00922	0.01287	0.01264	0.0126	0.046	0.029	0.028
	13	0.00967	0.01343	0.01323	0.01322	0.044	0.031	0.028
1	1	0.00865	0.01221	0.01207	0.012	0.04	0.028	0.022
	2	0.00926	0.01295	0.01288	0.01267	0.042	0.024	0.023
	3	0.00868	0.01228	0.01211	0.012	0.042	0.027	0.025
	4	0.0083	0.01151	0.01146	0.01141	0.043	0.025	0.025
	5	0.009	0.01278	0.01263	0.0125	0.042	0.028	0.026
	6	0.00888	0.0125	0.01234	0.01226	0.041	0.029	0.023
	7	0.009	0.01268	0.01256	0.01247	0.051	0.026	0.023

		8	0.00888	0.01261	0.01242	0.01236	0.046	0.025	0.024
		9	0.0087	0.01243	0.01222	0.01214	0.045	0.029	0.024
		10	0.00866	0.01233	0.01213	0.01216	0.043	0.026	0.026
		11	0.00859	0.01217	0.01201	0.01193	0.04	0.03	0.024
		12	0.00888	0.01251	0.01233	0.01225	0.049	0.029	0.029
		13	0.00955	0.0133	0.01313	0.01307	0.044	0.031	0.027
	1.14	1	0.00938	0.01293	0.01281	0.01269	0.042	0.029	0.022
		2	0.00997	0.01364	0.0136	0.01339	0.041	0.025	0.022
		3	0.00937	0.01293	0.0128	0.01264	0.041	0.026	0.025
		4	0.00916	0.01236	0.01234	0.01226	0.045	0.026	0.026
		5	0.00976	0.01348	0.01334	0.01317	0.041	0.027	0.025
		6	0.00943	0.01303	0.01289	0.01277	0.041	0.03	0.024
		7	0.00978	0.01343	0.01331	0.01319	0.051	0.028	0.025
		8	0.0095	0.01325	0.01303	0.01299	0.049	0.027	0.024
		9	0.00925	0.01295	0.01275	0.01263	0.045	0.028	0.025
		10	0.00975	0.01342	0.01322	0.01323	0.044	0.027	0.028
		11	0.0094	0.01296	0.01282	0.01272	0.04	0.031	0.024
		12	0.00939	0.01297	0.01281	0.01269	0.052	0.03	0.03
		13	0.00956	0.01328	0.01313	0.01304	0.044	0.031	0.027
HAR(B3LYP)	0.625	1	0.00883333	0.0123667	0.0119333	0.0119667	0.0366667	0.0326667	0.0333333
		2	0.0094	0.0132	0.0127	0.0126	0.034	0.033	0.037
		3	0.0091	0.0127667	0.0123333	0.0123	0.04	0.0323333	0.036
		4	0.0088	0.0121	0.0117333	0.0117	0.04	0.0323333	0.036
		5	0.009	0.0128	0.0124333	0.0123667	0.037	0.0343333	0.0363333
		6	0.00936667	0.0132	0.0127667	0.0126667	0.0343333	0.0343333	0.0406667
		7	0.009	0.0126	0.0122	0.0122	0.043	0.033	0.034
		8	0.00916667	0.0128	0.0124	0.0123667	0.049	0.0326667	0.0336667
		9	0.00886667	0.0124667	0.012	0.0120667	0.039	0.0316667	0.0396667
		10	0.00886667	0.0125333	0.0120333	0.0119667	0.0386667	0.033	0.0363333
		11	0.00886667	0.0125	0.012	0.0121	0.041	0.033	0.0373333
		12	0.00873333	0.0122667	0.0117667	0.0117667	0.0503333	0.0286667	0.0373333
		13	0.0093	0.0130333	0.0126667	0.0125333	0.037	0.0303333	0.0353333
	0.71429	1	0.0087	0.0123333	0.0119333	0.0118	0.0346667	0.0323333	0.0313333
		2	0.0093	0.0131	0.0127	0.0125	0.031	0.032	0.034
		3	0.0088	0.0125333	0.0120667	0.0119667	0.035	0.033	0.0326667

	4	0.0085	0.0118	0.0115	0.0114	0.0356667	0.032	0.0333333
	5	0.00903333	0.0129333	0.0125667	0.0123667	0.034	0.034	0.0333333
	6	0.00949667	0.0132733	0.01297	0.0127533	0.033	0.035	0.0376667
	7	0.009	0.0127	0.0124	0.0122	0.039	0.032	0.032
	8	0.00893333	0.0127	0.0123667	0.0121667	0.0436667	0.0323333	0.0303333
	9	0.00866667	0.0123633	0.0120367	0.0118833	0.036	0.0316667	0.0373333
	10	0.00863333	0.0124333	0.0120333	0.0119333	0.0356667	0.0333333	0.0353333
	11	0.00873333	0.0124	0.0119667	0.0119333	0.038	0.033	0.0353333
	12	0.00866667	0.0122	0.0119	0.0118	0.049	0.0293333	0.035
	13	0.00916667	0.0129	0.0125	0.0123667	0.0316667	0.0303333	0.033
0.83333	1	0.00850333	0.0121733	0.0118167	0.01167	0.0333333	0.0323333	0.0306667
	2	0.0091	0.01285	0.01256	0.01229	0.029	0.032	0.032
	3	0.00863667	0.0123367	0.0119567	0.0117633	0.0316667	0.0313333	0.0323333
	4	0.00824	0.01157	0.0112867	0.01117	0.0316667	0.0306667	0.0323333
	5	0.00891	0.0127933	0.01244	0.0122267	0.0313333	0.033	0.0326667
	6	0.00946	0.0132333	0.0128833	0.0127067	0.0313333	0.035	0.036
	7	0.0089	0.01261	0.0123	0.0121	0.036	0.031	0.031
	8	0.0088	0.0125667	0.0122667	0.0120667	0.0403333	0.0313333	0.0283333
	9	0.00859333	0.01235	0.0120067	0.01188	0.035	0.0313333	0.036
	10	0.00863667	0.0124633	0.0120333	0.0118833	0.0323333	0.0336667	0.0333333
	11	0.00863667	0.0122867	0.0118867	0.0118133	0.0366667	0.033	0.0346667
	12	0.00842333	0.0121333	0.01178	0.0116067	0.031	0.0326667	0.031
	13	0.00893333	0.0126567	0.0122933	0.0121033	0.0306667	0.03	0.0313333
1	1	0.00839	0.01207	0.0117333	0.0115733	0.0333333	0.032	0.0303333
	2	0.00905	0.01282	0.01252	0.01224	0.03	0.032	0.032
	3	0.00844667	0.0121367	0.0118	0.0116067	0.033	0.031	0.0326667
	4	0.00804333	0.0113767	0.01112	0.01099	0.033	0.0313333	0.0316667
	5	0.00872667	0.0126033	0.0122733	0.01206	0.0313333	0.0316667	0.0323333
	6	0.00937333	0.0131467	0.0128033	0.01264	0.0313333	0.035	0.0356667
	7	0.00885	0.01257	0.01226	0.01206	0.036	0.031	0.031
	8	0.00873667	0.0125	0.0122367	0.01202	0.0396667	0.0316667	0.0286667
	9	0.00862	0.0124	0.01206	0.0119	0.0343333	0.0313333	0.036
	10	0.00841	0.0122333	0.0118567	0.0116833	0.0316667	0.0333333	0.0333333
	11	0.00845667	0.0121667	0.0117933	0.01171	0.0373333	0.0326667	0.0346667
	12	0.00832333	0.0120033	0.0117	0.01153	0.032	0.0323333	0.0313333

		13	0.00862333	0.0123233	0.0120067	0.0118167	0.0313333	0.029	0.0313333
	1.14	1	0.00910333	0.01279	0.01246	0.0122867	0.0356667	0.032	0.0306667
		2	0.00966667	0.01348	0.0131967	0.0129367	0.0333333	0.034	0.0323333
		3	0.00909667	0.01278	0.0124667	0.01227	0.036	0.033	0.0343333
		4	0.00888333	0.0122233	0.0119733	0.0118367	0.038	0.0326667	0.0333333
		5	0.00945333	0.0132833	0.01297	0.01275	0.0343333	0.0326667	0.0326667
		6	0.00937	0.0131367	0.0128067	0.0126333	0.0316667	0.035	0.0363333
		7	0.00917333	0.0128933	0.0125767	0.01238	0.034	0.0323333	0.032
		8	0.00950333	0.01327	0.01296	0.0127633	0.04	0.0326667	0.0333333
		9	0.00922667	0.0130433	0.0126867	0.0125667	0.0373333	0.033	0.0363333
		10	0.00895333	0.01276	0.0124	0.0122133	0.036	0.0336667	0.0346667
		11	0.00954	0.0132367	0.0128767	0.0127833	0.037	0.0343333	0.0346667
		12	0.00912667	0.0127967	0.0124967	0.0123267	0.0333333	0.034	0.031
		13	0.00910333	0.01278	0.01247	0.0122867	0.0346667	0.03	0.0316667
HAR(BLYP)	0.625	1	0.008833333	0.012366667	0.011933333	0.011966667	.036666667	032666667	033333333
		2	0.0095	0.0133	0.0129	0.0128	0.037	0.033	0.037
		3	0.0091	0.012766667	0.012333333	0.0123	0.04	032333333	0.036
		4	0.0088	0.0121	0.011733333	0.0117	0.04	032333333	0.036
		5	0.009	0.0128	0.012433333	0.012366667	.037333333	034333333	036333333
		6	0.009533333	0.013133333	0.012766667	0.012666667	0.039	0.034	0.035
		7	0.0091	0.0127	0.0123	0.0123	0.047	0.033	0.034
		8	0.008866667	0.012466667	0.012	0.012066667	0.039	031666667	0.04
		9	0.008866667	0.012533333	0.012033333	0.011966667	.038666667	0.033	036333333
		10	0.008866667	0.0125	0.012	0.0121	0.041	0.033	037333333
		11	0.008733333	0.012266667	0.011766667	0.011766667	.050333333	028666667	037333333
		12	0.0093	0.013033333	0.012666667	0.012533333	0.037	030333333	035333333
		13	0.009433333	0.013333333	0.012933333	0.0128	.037333333	0.034	0.041
	0.71429	1	0.0087	0.012333333	0.011933333	0.0118	.034666667	032333333	031333333
		2	0.0093	0.0131	0.0127	0.0125	0.034	0.032	0.034
		3	0.0088	0.012533333	0.012066667	0.011966667	0.035	0.033	032666667
		4	0.0085	0.0118	0.0115	0.0114	.035666667	0.032	033333333
		5	0.009033333	0.012933333	0.012566667	0.012366667	0.034	0.034	033333333
		6	0.009166667	0.0128	0.0126	0.012433333	.038333333	0.034	0.033
		7	0.009	0.0127	0.0124	0.0122	0.042	0.032	0.032
		8	0.008666667	0.012363333	0.012036667	0.011883333	0.036	031666667	037333333

	9	0.008633333	0.012433333	0.012033333	0.011933333	.035666667	033333333	035333333
	10	0.008733333	0.0124	0.011966667	0.011933333	0.038	0.033	035333333
	11	0.008666667	0.0122	0.0119	0.0118	0.049	029333333	0.035
	12	0.009166667	0.0129	0.0125	0.012366667	.031666667	030666667	0.033
	13	0.009536667	0.01333	0.013046667	0.01283	.035333333	034666667	0.038
0.83333	1	0.008503333	0.012173333	0.011816667	0.01167	.033333333	032333333	030666667
	2	0.0091	0.0129	0.01258	0.01232	0.031	0.032	0.032
	3	0.008636667	0.012336667	0.011956667	0.011763333	.031666667	031333333	032333333
	4	0.00824	0.01157	0.011286667	0.01117	.031666667	030666667	032333333
	5	0.00891	0.012793333	0.01244	0.012226667	.031333333	0.033	032666667
	6	0.008906667	0.01264	0.01227	0.012086667	0.032	033333333	030666667
	7	0.00882	0.0126	0.01229	0.01209	0.038	0.031	0.031
	8	0.008593333	0.01235	0.012006667	0.01188	0.035	031333333	0.036
	9	0.008636667	0.012463333	0.012033333	0.011883333	.032333333	033666667	033333333
	10	0.008636667	0.012286667	0.011886667	0.011813333	.036666667	0.033	034666667
	11	0.008423333	0.012133333	0.01178	0.011606667	0.031	0.033	0.031
	12	0.008933333	0.012656667	0.012293333	0.012103333	.030666667	0.03	031333333
	13	0.00941	0.01324	0.012896667	0.01272	.033666667	034666667	036333333
1	1	0.00839	0.01207	0.011733333	0.011573333	.033333333	0.032	030333333
	2	0.00899	0.01282	0.01251	0.01224	0.033	0.032	0.032
	3	0.008446667	0.012136667	0.0118	0.011606667	0.033	0.031	032666667
	4	0.008043333	0.011376667	0.01112	0.01099	0.033	031333333	031666667
	5	0.008726667	0.012603333	0.012273333	0.01206	.031333333	031666667	032333333
	6	0.00864	0.012356667	0.012023333	0.01185	0.033	032666667	030666667
	7	0.00877	0.01254	0.01223	0.01203	0.038	0.031	0.031
	8	0.00862	0.0124	0.01206	0.0119	.034333333	031333333	036333333
	9	0.00841	0.012233333	0.011856667	0.011683333	.031666667	033333333	033333333
	10	0.008456667	0.012166667	0.011793333	0.01171	.037333333	032666667	034666667
	11	0.008323333	0.012003333	0.0117	0.01153	0.032	032333333	031333333
	12	0.008623333	0.012323333	0.012006667	0.011816667	.031333333	0.029	031333333
	13	0.009303333	0.013133333	0.012793333	0.012623333	0.034	034333333	0.036
1.14	1	0.009103333	0.01279	0.01246	0.012286667	.035666667	0.032	030666667
	2	0.009666667	0.01348	0.013196667	0.012936667	.033333333	0.034	032333333
	3	0.009096667	0.01278	0.012466667	0.01227	0.036	0.033	034333333
	4	0.008883333	0.012223333	0.011973333	0.011836667	0.038	032666667	033333333

		5	0.009453333	0.013283333	0.01297	0.01275	.034333333	032666667	032666667
		6	0.009173333	0.012893333	0.012576667	0.01238	0.034	032333333	0.032
		7	0.009503333	0.01327	0.01296	0.012763333	0.04	032666667	033333333
		8	0.009226667	0.013043333	0.012686667	0.012566667	.037333333	0.033	036333333
		9	0.008953333	0.01276	0.0124	0.012213333	0.036	033666667	034666667
		10	0.00954	0.013236667	0.012876667	0.012783333	0.037	034333333	034666667
		11	0.009126667	0.012796667	0.012496667	0.012326667	.033333333	0.034	0.031
		12	0.009103333	0.01278	0.01247	0.012286667	.034666667	0.03	031666667
		13	0.009306667	0.013116667	0.01279	0.012613333	0.034	034333333	036666667
MR	1.18	1	0.00925	0.0129	0.01267	0.01254	0.02242	0.0282	0.02793
		2	0.0106	0.0141	0.0139	0.0137	0.02374	0.02957	0.02929
		3	0.00955	0.01308	0.01286	0.01271	0.02254	0.02827	0.028
		4	0.00913	0.0124	0.0122	0.0121	0.02121	0.02651	0.02626
		5	0.00982	0.01356	0.01332	0.01316	0.02332	0.02926	0.02898
		6	0.00942	0.0131	0.01287	0.01272	0.02275	0.02862	0.02834
		7	0.00981	0.0135	0.01331	0.01316	0.02334	0.02931	0.02902
		8	0.00937	0.01318	0.01293	0.01282	0.02292	0.02886	0.02858
		9	0.00934	0.01301	0.01278	0.01261	0.02256	0.02837	0.02809
		10	0.01	0.0136	0.0133	0.0132	0.02297	0.02866	0.02839
		11	0.00928	0.01293	0.01271	0.01257	0.02256	0.02839	0.02811
		12	0.00947	0.01305	0.01285	0.01271	0.02253	0.02827	0.028
		13	0.00961	0.01335	0.01311	0.01298	0.02316	0.02911	0.02883

Method	Resolution [Å $^{-1}$]	nr set	Maximum Residual Electron Density [Å $^{-3}$]	Mininum Residual Electron Density [Å $^{-3}$]
IAM	0.625	1	4.635003E-01	-0.2189433
		2	4.342294E-01	-0.1777725
		3	4.494653E-01	-0.2159741
		4	4.255269E-01	-0.1902115
		5	3.672872E-01	-0.2977395
		6	3.033256E-01	-0.3131455
		7	5.666734E-01	-0.3826663
		8	4.515636E-01	-0.2923381
		9	4.259988E-01	-0.3400299
		10	3.879580E-01	-0.2225367
		11	4.508210E-01	-0.2342311
		12	4.673788E-01	-0.1625391
		13	4.296700E-01	-0.2071036
	0.71429	1	6.004492E-01	-0.213548
		2	5.850706E-01	-0.1989521
		3	5.657084E-01	-0.2088289
		4	5.638103E-01	-0.1981631
		5	4.709963E-01	-0.3350922
		6	4.240453E-01	-0.321723
		7	5.239522E-01	-0.3443988
		8	5.795595E-01	-0.2776778
		9	5.278539E-01	-0.3304747
		10	5.031858E-01	-0.2527877
		11	6.051826E-01	-0.2549478
		12	5.362330E-01	-0.1809468
		13	5.624085E-01	-0.2138779
	0.83333	1	5.975403E-01	-0.2669321
		2	6.565808E-01	-0.2683085
		3	5.830488E-01	-0.2405192
		4	5.759761E-01	-0.2186458
		5	4.675473E-01	-0.3488629
		6	4.531468E-01	-0.3104072
		7	5.331596E-01	-0.2757879
		8	6.162524E-01	-0.2790989
		9	5.453510E-01	-0.3649919

		10	5.289429E-01	-0.2741719
		11	5.835759E-01	-0.2683765
		12	5.732175E-01	-0.2273009
		13	5.713756E-01	-0.2587243
	1	1	6.035663E-01	-0.2620036
		2	6.144785E-01	-0.3423606
		3	5.905430E-01	-0.2469354
		4	6.271300E-01	-0.2909745
		5	5.793192E-01	-0.3859554
		6	4.836550E-01	-0.3568949
		7	6.900983E-01	-0.3559835
		8	6.006437E-01	-0.3128662
		9	5.510580E-01	-0.4000303
		10	5.368560E-01	-0.2855674
		11	5.816926E-01	-0.2971268
		12	6.009031E-01	-0.2504581
		13	5.744149E-01	-0.2961091
	1.14	1	7.126641E-01	-0.2800131
		2	7.793164E-01	-0.4967541
		3	6.054880E-01	-0.2864814
		4	7.139458E-01	-0.292154
		5	6.810491E-01	-0.3879512
		6	5.979439E-01	-0.351775
		7	6.607729E-01	-0.3086563
		8	5.935635E-01	-0.3538684
		9	5.725738E-01	-0.3861657
		10	5.502949E-01	-0.286954
		11	5.871785E-01	-0.3100474
		12	6.773674E-01	-0.2866766
		13	6.056646E-01	-0.3356582
UBDB	0.625	1	8.851739E-02	-0.1492737
		2	9.832448E-02	-0.0879844
		3	1.072063E-01	-0.1244659
		4	9.583871E-02	-0.0979634
		5	2.246190E-01	-0.2558207
		6	2.052842E-01	-0.2615591
		7	3.191344E-01	-0.3947905
		8	9.622374E-02	-0.1248174
		9	1.082966E-01	-0.1401736

		10	1.117046E-01	-0.1415248
		11	1.344899E-01	-0.1621848
		12	9.732523E-02	-0.1305097
		13	9.822941E-02	-0.1531301
	0.71429	1	1.275685E-01	-0.1354226
		2	1.361035E-01	-0.1190569
		3	1.720489E-01	-0.159549
		4	1.795503E-01	-0.1103988
		5	2.369703E-01	-0.2880618
		6	2.012047E-01	-0.2479386
		7	3.565517E-01	-0.3947833
		8	1.136172E-01	-0.1385313
		9	1.276712E-01	-0.1819883
		10	1.487941E-01	-0.1539334
		11	1.783854E-01	-0.1879066
		12	1.297223E-01	-0.1733596
		13	1.004457E-01	-0.1912339
	0.83333	1	1.645023E-01	-0.2148343
		2	2.110857E-01	-0.1523854
		3	1.782423E-01	-0.1722126
		4	2.359103E-01	-0.1386656
		5	2.188040E-01	-0.2911643
		6	2.142635E-01	-0.2788952
		7	2.892956E-01	-0.3665781
		8	1.777818E-01	-0.1744774
		9	2.333678E-01	-0.2423108
		10	2.564350E-01	-0.1659503
		11	1.758039E-01	-0.2067306
		12	1.605451E-01	-0.1597685
		13	1.240640E-01	-0.2266533
	1	1	2.495895E-01	-0.2275894
		2	3.758761E-01	-0.2862761
		3	2.746957E-01	-0.2416922
		4	3.317496E-01	-0.1952715
		5	2.200130E-01	-0.3089628
		6	2.403992E-01	-0.3238901
		7	3.375896E-01	-0.3829303
		8	2.252119E-01	-0.2298243
		9	2.925423E-01	-0.3370341

		10	3.441010E-01	-0.2237421
		11	2.212015E-01	-0.2554166
		12	2.611575E-01	-0.2059256
		13	1.753865E-01	-0.3001013
	1.14	1	3.558890E-01	-0.2491102
		2	4.936604E-01	-0.5237992
		3	3.793369E-01	-0.3082709
		4	3.992267E-01	-0.1907211
		5	2.635684E-01	-0.3496397
		6	2.419544E-01	-0.3124002
		7	2.886681E-01	-0.2914921
		8	2.764054E-01	-0.2897789
		9	3.618753E-01	-0.3902523
		10	3.843986E-01	-0.2250121
		11	2.089331E-01	-0.2498828
		12	2.834780E-01	-0.262015
		13	2.269212E-01	-0.3747973
ELMAM2	0.625	1	8.123275E-02	-0.1025917
		2	1.021821E-01	-0.1099702
		3	9.865632E-02	-0.134705
		4	9.301916E-02	-0.1231808
		5	2.200693E-01	-0.2702528
		6	2.032732E-01	-0.2461207
		7	3.123888E-01	-0.3969628
		8	8.336345E-02	-0.1121809
		9	1.048257E-01	-0.1336406
		10	1.032165E-01	-0.1474621
		11	1.313628E-01	-0.1528027
		12	8.789120E-02	-0.1006149
		13	9.321090E-02	-0.1244398
	0.71429	1	1.101731E-01	-0.100821
		2	1.493292E-01	-0.1438032
		3	1.619079E-01	-0.1448487
		4	1.742841E-01	-0.1230687
		5	2.128776E-01	-0.2936123
		6	2.025965E-01	-0.2517414
		7	3.096944E-01	-0.3641676
		8	1.225354E-01	-0.1248098
		9	1.313259E-01	-0.1799363

	10	1.474795E-01	-0.1636699
	11	1.656005E-01	-0.1453229
	12	1.065439E-01	-0.1235192
	13	9.958573E-02	-0.1574301
0.83333	1	1.671317E-01	-0.1705074
	2	2.156795E-01	-0.2122206
	3	1.838262E-01	-0.1730252
	4	2.397314E-01	-0.1551733
	5	2.202134E-01	-0.3063002
	6	2.152316E-01	-0.282721
	7	2.625734E-01	-0.3639512
	8	1.810688E-01	-0.185013
	9	2.370498E-01	-0.2270407
	10	2.723177E-01	-0.1959015
	11	1.621216E-01	-0.1636082
	12	1.617345E-01	-0.1559693
	13	1.282903E-01	-0.2054244
1	1	1.613330E-01	-0.2096169
	2	2.437405E-01	-0.2450366
	3	1.476123E-01	-0.2040854
	4	1.942511E-01	-0.1625495
	5	2.028442E-01	-0.2767046
	6	2.289495E-01	-0.3042909
	7	3.125353E-01	-0.4097961
	8	1.657997E-01	-0.2477903
	9	1.747557E-01	-0.2874571
	10	1.665497E-01	-0.2048197
	11	1.268697E-01	-0.2272716
	12	1.698658E-01	-0.1697105
	13	1.435206E-01	-0.2637039
1.14	1	3.530800E-01	-0.225845
	2	6.071377E-01	-0.4428617
	3	3.579452E-01	-0.2494822
	4	3.910544E-01	-0.2381566
	5	2.600878E-01	-0.3453432
	6	2.596002E-01	-0.3149075
	7	2.721982E-01	-0.2690795
	8	2.707389E-01	-0.3015094
	9	3.691977E-01	-0.3224299

		10	3.971074E-01	-0.2511698
		11	2.120730E-01	-0.2256834
		12	3.101909E-01	-0.2145703
		13	2.568480E-01	-0.3657759
GID	0.625	1	8.123275E-02	-1.025917E-01
		2	1.021821E-01	-1.099702E-01
		3	9.865632E-02	-1.347050E-01
		4	9.301916E-02	-1.231808E-01
		5	2.200693E-01	-2.702528E-01
		6	2.032732E-01	-2.461207E-01
		7	3.123888E-01	-3.969628E-01
		8	8.336345E-02	-1.121809E-01
		9	1.048257E-01	-1.336406E-01
		10	1.032165E-01	-1.474621E-01
		11	1.313628E-01	-1.528027E-01
		12	8.789120E-02	-1.006149E-01
		13	9.321090E-02	-1.244398E-01
	0.71429	1	1.101731E-01	-1.008210E-01
		2	1.493292E-01	-1.438032E-01
		3	1.619079E-01	-1.448487E-01
		4	1.742841E-01	-1.230687E-01
		5	2.128776E-01	-2.936123E-01
		6	2.025965E-01	-2.517414E-01
		7	3.096944E-01	-3.641676E-01
		8	1.225354E-01	-1.248098E-01
		9	1.313259E-01	-1.799363E-01
		10	1.474795E-01	-1.636699E-01
		11	1.656005E-01	-1.453229E-01
		12	1.065439E-01	-1.235192E-01
		13	9.958573E-02	-1.574301E-01
	0.83333	1	1.671317E-01	-1.705074E-01
		2	2.156795E-01	-2.122206E-01
		3	1.838262E-01	-1.730252E-01
		4	2.397314E-01	-1.551733E-01
		5	2.202134E-01	-3.063002E-01
		6	2.152316E-01	-2.827210E-01
		7	2.625734E-01	-3.639512E-01
		8	1.810688E-01	-1.850130E-01
		9	2.370498E-01	-2.270407E-01

		10	2.723177E-01	-1.959015E-01
		11	1.621216E-01	-1.636082E-01
		12	1.617345E-01	-1.559693E-01
		13	1.282903E-01	-2.054244E-01
	1	1	2.435007E-01	-1.616983E-01
		2	4.129556E-01	-2.582873E-01
		3	2.699091E-01	-2.052647E-01
		4	3.183504E-01	-1.861335E-01
		5	2.198978E-01	-3.354448E-01
		6	2.237606E-01	-3.244394E-01
		7	3.157961E-01	-4.014413E-01
		8	2.347743E-01	-2.610110E-01
		9	3.000157E-01	-3.214679E-01
		10	3.338178E-01	-2.324066E-01
		11	2.149273E-01	-2.052075E-01
		12	2.518525E-01	-1.893403E-01
		13	1.871870E-01	-2.704211E-01
	1.14	1	3.530800E-01	-2.258450E-01
		2	6.071377E-01	-4.428617E-01
		3	3.579452E-01	-2.494822E-01
		4	3.910544E-01	-2.381566E-01
		5	2.600878E-01	-3.453432E-01
		6	2.596002E-01	-3.149075E-01
		7	2.721982E-01	-2.690795E-01
		8	2.707389E-01	-3.015094E-01
		9	3.691977E-01	-3.224299E-01
		10	3.971074E-01	-2.511698E-01
		11	2.120730E-01	-2.256834E-01
		12	3.101909E-01	-2.145703E-01
		13	2.568480E-01	-3.657759E-01
HAR(B3LYP)	0.625	1	0.072118	-0.070891
		2	0.137683	-0.142461
		3	0.074591	-0.077798
		4	0.054061	-0.052778
		5	0.165170	-0.210088
		6	0.163333	-0.186088
		7	0.352102	-0.367146
		8	0.074740	-0.072401
		9	0.120000	-0.220000

	10	0.054619	-0.054322
	11	0.098103	-0.110655
	12	0.065499	-0.068497
	13	0.084360	-0.093562
0.71429	1	0.093028	-0.079573
	2	0.151278	-0.158026
	3	0.074068	-0.090003
	4	0.065825	-0.069024
	5	0.183599	-0.213789
	6	0.161821	-0.173611
	7	0.275260	-0.294929
	8	0.081123	-0.094288
	9	0.118357	-0.130314
	10	0.069095	-0.074865
	11	0.118075	-0.106708
	12	0.076729	-0.075476
	13	0.083285	-0.112341
0.83333	1	0.099783	-0.120387
	2	0.184537	-0.194507
	3	0.091551	-0.094791
	4	0.082052	-0.077519
	5	0.182545	-0.219131
	6	0.162146	-0.186051
	7	0.237349	-0.268995
	8	0.095753	-0.123760
	9	0.126938	-0.149290
	10	0.109870	-0.096574
	11	0.125218	-0.110787
	12	0.080915	-0.078234
	13	0.091038	-0.139956
1	1	0.105746	-0.119441
	2	0.238825	-0.241401
	3	0.098937	-0.095523
	4	0.125731	-0.119971
	5	0.179317	-0.218727
	6	0.178807	-0.195650
	7	0.232587	-0.299196
	8	0.126618	-0.146166
	9	0.127447	-0.199203

		10	0.146643	-0.123311
		11	0.119412	-0.123301
		12	0.125101	-0.082918
		13	0.117180	-0.173778
	1.14	1	0.143003	-0.150339
		2	0.364148	-0.326987
		3	0.157294	-0.134835
		4	0.175164	-0.147584
		5	0.202041	-0.206650
		6	0.157151	-0.171079
		7	0.131917	-0.201416
		8	0.178953	-0.195392
		9	0.161044	-0.217762
		10	0.194369	-0.145854
		11	0.112492	-0.118581
		12	0.184440	-0.108124
		13	0.136850	-0.218870
HAR(BLYP)	0.625	1	0.078882	-0.085340
		2	0.139247	-0.142659
		3	0.077533	-0.076381
		4	0.053835	-0.047880
		5	0.165612	-0.207187
		6	0.159788	-0.177411
		7	0.360133	-0.361279
		8	0.077816	-0.092131
		9	0.120000	-0.220000
		10	0.057611	-0.054622
		11	0.098001	-0.104639
		12	0.065487	-0.067720
		13	0.086225	-0.087957
	0.71429	1	0.089706	-0.081301
		2	0.152941	-0.166308
		3	0.087848	-0.079788
		4	0.064910	-0.070733
		5	0.181736	-0.210596
		6	0.160432	-0.163026
		7	0.274000	-0.291878
		8	0.082877	-0.117122
		9	0.119806	-0.131185

		10	0.077205	-0.073054
		11	0.112344	-0.118461
		12	0.072165	-0.070735
		13	0.081977	-0.101351
	0.83333	1	0.126026	-0.117774
		2	0.184881	-0.202673
		3	0.103204	-0.094900
		4	0.089079	-0.073961
		5	0.180511	-0.213078
		6	0.164192	-0.173209
		7	0.231490	-0.257799
		8	0.113960	-0.120228
		9	0.118976	-0.160128
		10	0.123685	-0.095091
		11	0.119631	-0.113272
		12	0.079530	-0.091984
		13	0.091069	-0.130073
	1	1	0.105896	-0.110421
		2	0.242383	-0.247691
		3	0.105738	-0.093042
		4	0.132650	-0.123966
		5	0.177668	-0.213490
		6	0.179266	-0.178642
		7	0.232334	-0.296319
		8	0.125950	-0.146067
		9	0.140283	-0.196425
		10	0.151271	-0.136987
		11	0.115718	-0.113667
		12	0.127432	-0.092470
		13	0.116347	-0.162022
	1.14	1	0.143347	-0.154720
		2	0.397352	-0.356662
		3	0.168013	-0.139299
		4	0.187149	-0.154467
		5	0.205002	-0.196406
		6	0.162774	-0.171356
		7	0.142982	-0.191030
		8	0.176263	-0.188860
		9	0.168714	-0.207539

		10	0.201391	-0.145180
		11	0.108236	-0.109660
		12	0.184865	-0.112981
		13	0.151198	-0.201299
MR_no	1.19	1	1.272758E-01	-1.301553E-01
		2	1.710372E-01	-1.845611E-01
		3	1.405702E-01	-1.460192E-01
		4	1.444869E-01	-1.282589E-01
		5	1.907813E-01	-2.003284E-01
		6	2.036778E-01	-2.217741E-01
		7	1.469912E-01	-1.541599E-01
		8	1.379022E-01	-1.804870E-01
		9	1.997217E-01	-1.997565E-01
		10	1.320825E-01	-1.693530E-01
		11	1.348663E-01	-1.660045E-01
		12	1.230037E-01	-1.123192E-01
		13	1.431347E-01	-1.802086E-01

Method	Resolution [Å $^{-1}$]	nr set	Cohesive Energy [kJ/mol]
IAM_Hbonds_const	0.625	1	-219.3542063
		2	-219.8535185
		3	-220.5731528
		4	-220.0326282
		5	-221.4048769
		6	-219.2617653
		7	-217.4991492
		8	-221.1508853
		9	-220.1125858
		10	-220.1672448
		11	-218.8540698
		12	-216.8877884
		13	-218.9625273
	0.71429	1	-217.5731417
		2	-218.6899051
		3	-219.5276753
		4	-219.1578702
		5	-220.026651
		6	-219.8012827
		7	-217.8594265
		8	-219.3397421
		9	-218.1364223
		10	-218.9053269
		11	-217.9673432
		12	-217.7546317
		13	-218.5424617
	0.83333	1	-217.7889931
		2	-219.0950168
		3	-177.2641671
		4	-188.7841904
		5	-189.1817278
		6	-185.672107
		7	-219.4132057
		8	-187.8277972
		9	-188.5254566
		10	-183.3924827

		11	-192.1241263
		12	-169.7285677
		13	-186.3116179
	1	1	-217.7438041
		2	-219.4466811
		3	-219.9363205
		4	-219.8236031
		5	-220.3302124
		6	-217.8489903
		7	-219.8514912
		8	-220.5532193
		9	-219.0780219
		10	-219.5582848
		11	-218.127936
		12	-216.1996428
		13	-219.0502778
	1.14	1	-217.87878
		2	-218.8288935
		3	-218.8235243
		4	-219.709797
		5	-219.7325258
		6	-218.2031161
		7	-217.8190594
		8	-220.7257076
		9	-219.7657488
		10	-219.4826339
		11	-218.5253581
		12	-216.1005151
		13	-219.2919069
IAM	0.625	1	-189.81486722
		2	-181.17517276
		3	-176.25841883
		4	-185.38280596
		5	-189.35152555
		6	-180.63224961
		7	-182.17418297
		8	-187.06320508
		9	-189.10751173
		10	-182.46583669

		11	-187.64755677
		12	-169.20903622
		13	-190.42903729
	0.71429	1	-190.1902252
		2	-177.2338384
		3	-175.7640704
		4	-185.5590925
		5	-185.9584886
		6	-181.2807263
		7	-181.6630539
		8	-186.5221219
		9	-185.9568878
		10	-182.5764521
		11	-187.1328657
		12	-168.6852712
		13	-185.2562694
	0.83333	1	-191.6821967
		2	-181.9889628
		3	-177.2641671
		4	-188.7841905
		5	-189.1817278
		6	-185.672107
		7	-184.3714633
		8	-187.8277972
		9	-188.5254566
		10	-183.3924827
		11	-192.1241263
		12	-169.7285677
		13	-186.3116179
	1	1	-192.2809946
		2	-181.1776137
		3	-177.4654344
		4	-189.1974643
		5	-188.8186114
		6	-185.6085665
		7	-185.0594767
		8	-189.8598409
		9	-189.242601
		10	-184.1434254

		11	-192.4440003
		12	-170.8318702
		13	-187.8154442
	1.14	1	-192.0438884
		2	-180.6788579
		3	-178.701328
		4	-186.1968066
		5	-193.2669369
		6	-187.7452399
		7	-192.305047
		8	-190.1423528
		9	-190.6531121
		10	-183.9218108
		11	-190.2936331
		12	-167.71379
		13	-187.8277668
UBDB	0.625	1	-220.7198966
		2	-213.6302483
		3	-216.8629769
		4	-218.3941166
		5	-220.8857929
		6	-218.7932618
		7	-226.3500987
		8	-227.5467212
		9	-223.4802791
		10	-217.5884464
		11	-212.9521139
		12	-211.8478369
		13	-223.470075
	0.71429	1	-215.4955209
		2	-209.6371063
		3	-206.1233405
		4	-213.5810998
		5	-214.4065759
		6	-208.9709482
		7	-208.025555
		8	-223.7491762
		9	-215.9114295
		10	-214.2013558

	11	-208.2283717
	12	-202.7391632
	13	-215.848982
0.83333	1	-211.1667975
	2	-208.1091762
	3	-208.1077851
	4	-212.1117505
	5	-210.6263571
	6	-209.6069671
	7	-205.2820381
	8	-218.7520961
	9	-212.1636757
	10	-211.9976639
	11	-206.4116754
	12	-204.0432188
	13	-214.7090722
1	1	-208.0540659
	2	-205.3715284
	3	-204.2083264
	4	-210.1362906
	5	-209.071413
	6	-205.7074051
	7	-202.8379079
	8	-216.9585326
	9	-213.3081883
	10	-208.8107359
	11	-202.2344085
	12	-201.1640121
	13	-211.3766485
1.14	1	-204.4948735
	2	-202.1530741
	3	-202.7509325
	4	-209.5933934
	5	-200.1970783
	6	-202.9072787
	7	-208.7046635
	8	-215.1418771
	9	-209.7371431
	10	-207.9925814

		11	-200.3824653
		12	-198.3384578
		13	-210.8726981
INVARIOM	0.625	1	-224.5205915
		2	-216.0670164
		3	-223.9250399
		4	-224.8670664
		5	-223.2002126
		6	-222.2122958
		7	-219.1978677
		8	-227.3080394
		9	-226.8808111
		10	-221.2947951
		11	-215.9024975
		12	-219.5186617
		13	-225.8725455
	0.71429	1	-220.4186704
		2	-210.5315274
		3	-214.0378995
		4	-218.7878127
		5	-216.7000157
		6	-212.5266886
		7	-213.9702549
		8	-225.0954866
		9	-221.2555734
		10	-217.8905931
		11	-217.1804193
		12	-216.7432588
		13	-218.7446511
	0.83333	1	-215.7032544
		2	-208.6553463
		3	-210.524149
		4	-214.5647928
		5	-217.2091669
		6	-212.4091787
		7	-211.9253955
		8	-218.8922336
		9	-217.1218473
		10	-214.6571912

		11	-215.0898523
		12	-218.7513133
		13	-215.2157448
	1	1	-212.7887265
		2	-206.5511579
		3	-211.9396564
		4	-213.1890208
		5	-215.973194
		6	-208.3509616
		7	-209.433488
		8	-218.3747273
		9	-217.3946352
		10	-211.1377308
		11	-211.7788407
		12	-214.3973393
		13	-213.3131262
	1.14	1	-208.6019671
		2	-204.1073831
		3	-209.6014087
		4	-210.5337693
		5	-204.9914595
		6	-205.1320404
		7	-214.8621893
		8	-216.422119
		9	-211.3527961
		10	-211.0639189
		11	-206.874968
		12	-207.7251249
		13	-212.6411757
ELMAM2	0.625	1	-224.5205915
		2	-216.0670164
		3	-223.9250399
		4	-224.8670663
		5	-223.2002126
		6	-222.2122958
		7	-219.1978678
		8	-227.3080392
		9	-226.8808111
		10	-221.2947951

	11	-215.9024975
	12	-219.5186617
	13	-225.8725455
0.71429	1	-220.4186704
	2	-210.5315274
	3	-214.0378995
	4	-218.7878127
	5	-216.7000157
	6	-212.5266886
	7	-213.9702549
	8	-225.0954866
	9	-221.2555734
	10	-217.8905931
	11	-217.1804193
	12	-216.7432588
	13	-218.7446511
0.83333	1	-215.7032544
	2	-208.6553463
	3	-210.524149
	4	-214.5647928
	5	-217.2091669
	6	-212.4091787
	7	-211.9253955
	8	-218.8922336
	9	-217.1218473
	10	-214.6571912
	11	-215.0898523
	12	-218.7513133
	13	-215.2157448
1	1	-236.9896645
	2	-230.8362853
	3	-228.0932885
	4	-235.5724947
	5	-234.4689518
	6	-228.3489729
	7	-228.6559989
	8	-237.7665703
	9	-233.9673082
	10	-235.4260714

		11	-233.3890134
		12	-226.7497365
		13	-235.6764903
	1.14	1	-208.6019671
		2	-204.1073831
		3	-209.6014087
		4	-210.5337693
		5	-204.9914595
		6	-205.1320405
		7	-214.8621893
		8	-216.422119
		9	-211.3527961
		10	-211.0639189
		11	-206.8749671
		12	-207.7251249
		13	-212.6411758
HAR(B3LYP)	0.625	1	-227.3070127
		2	-222.0253081
		3	-224.2298851
		4	-225.8091757
		5	-228.2371459
		6	-221.4058882
		7	-228.1158957
		8	-226.1784949
		9	-224.0771095
		10	-224.2982609
		11	-226.2438014
		12	-227.1039165
		13	-231.1263708
	0.71429	1	-227.3070127
		2	-222.0253081
		3	-224.2298851
		4	-225.8091757
		5	-228.2371459
		6	-221.4058882
		7	-228.1158957
		8	-226.1784949
		9	-224.0771095
		10	-224.2982609

		11	-226.2438014
		12	-227.1039165
		13	-231.1263708
	0.83333	1	-227.878566
		2	-223.5939313
		3	-226.4027884
		4	-227.006211
		5	-229.8672693
		6	-224.3873138
		7	-227.030096
		8	-226.1225964
		9	-224.4663594
		10	-226.4185449
		11	-231.4926777
		12	-228.1276231
		13	-230.9659262
	1	1	-227.3336733
		2	-223.3852241
		3	-224.9134889
		4	-229.3872755
		5	-229.4963768
		6	-224.5829612
		7	-225.6520286
		8	-227.0601407
		9	-214.95997
		10	-224.3961624
		11	-231.2571871
		12	-227.3235081
		13	-231.041217
	1.14	1	-226.2641325
		2	-217.8037064
		3	-223.423727
		4	-225.0858183
		5	-230.0886289
		6	-225.7020687
		7	-227.0313108
		8	-225.5259123
		9	-222.7704177
		10	-225.6126509

		11	-228.2539362
		12	-222.2634799
		13	-230.6221462
HAR(BLYP)	0.625	1	-224.8474201
		2	-219.5558501
		3	-222.3655903
		4	-221.7934431
		5	-225.180598
		6	-220.5305176
		7	-220.1807788
		8	-221.7473397
		9	-221.5150935
		10	-218.9473879
		11	-224.7563376
		12	-221.7945181
		13	-225.3784177
	0.71429	1	-227.3070127
		2	-218.3652968
		3	-224.2298851
		4	-225.6636175
		5	-228.2371459
		6	-221.4058882
		7	-223.9233808
		8	-226.1784949
		9	-224.0771095
		10	-224.2982609
		11	-226.336687
		12	-227.1039165
		13	-227.5134278
	0.83333	1	-227.878566
		2	-222.135483
		3	-226.4027884
		4	-227.006211
		5	-229.8257352
		6	-224.3873142
		7	-223.6772314
		8	-226.1225966
		9	-224.4663594
		10	-226.4185449

		11	-231.4926777
		12	-228.0907304
		13	-227.9895182
	1	1	-227.3336733
		2	-221.0055078
		3	-226.1990471
		4	-226.8414074
		5	-229.645657
		6	-224.4421998
		7	-222.9362218
		8	-225.7987068
		9	-225.7518855
		10	-224.3961624
		11	-231.257187
		12	-228.4821463
		13	-227.6514789
	1.14	1	-226.2641325
		2	-217.8037064
		3	-223.423727
		4	-225.2131534
		5	-230.0886289
		6	-225.2327246
		7	-227.1312511
		8	-225.5259123
		9	-222.7704176
		10	-225.6126509
		11	-228.2539362
		12	-222.26348
		13	-227.4318597
MR	1.18	1	-238.900
		2	-239.800
		3	-239.700
		4	-242.300
		5	-237.400
		6	-239.200
		7	-240.200
		8	-240.700
		9	-242.900
		10	-239.200

	11	-238.100
	12	-239.800
	13	-240.600
